# Nd─Nd Bond in I_h_ and D_5h_ Cage Isomers of Nd_2_@C_80_ Stabilized by Electrophilic CF_3_ Addition

**DOI:** 10.1002/advs.202305190

**Published:** 2023-11-09

**Authors:** Wei Yang, Georgios Velkos, Marco Rosenkranz, Sandra Schiemenz, Fupin Liu, Alexey A. Popov

**Affiliations:** ^1^ Leibniz Institute for Solid State and Materials Research Helmholtzstraße 20 01069 Dresden Germany

**Keywords:** electrophilic trifluoromethylation, lanthanide–lanthanide bond, magnetic properties, metallofullerene, single‐crystal X‐ray diffraction

## Abstract

Synthesis of molecular compounds with metal–metal bonds between 4f elements is recognized as one of the fascinating milestones in lanthanide metallochemistry. The main focus of such studies is on heavy lanthanides due to the interest in their magnetism, while bonding between light lanthanides remains unexplored. In this work, the Nd─Nd bonding in Nd‐dimetallofullerenes as a case study of metal–metal bonding between early lanthanides is demonstrated. Combined experimental and computational study proves that pristine Nd_2_@C_80_ has an open shell structure with a single electron occupying the Nd─Nd bonding orbital. Nd_2_@C_80_ is stabilized by a one‐electron reduction and further by the electrophilic CF_3_ addition to [Nd_2_@C_80_]^−^. Single‐crystal X‐ray diffraction reveals the formation of two Nd_2_@C_80_(CF_3_) isomers with D_5h_‐C_80_ and I_h_‐C_80_ carbon cages, both featuring a single‐electron Nd─Nd bond with the length of 3.78–3.79 Å. The mutual influence of the exohedral CF_3_ group and endohedral metal dimer in determining the molecular structure of the adducts is analyzed. Unlike Tb or Dy analogs, which are strong single‐molecule magnets with high blocking temperature of magnetization, the slow relaxation of magnetization in Nd_2_@I_h_‐C_80_(CF_3_) is detectable via out‐of‐phase magnetic susceptibility only below 3 K and in the presence of magnetic field.

## Graphical abstract

Two isomers of Nd‐dimetallofullerene derivative Nd_2_@C_80_(CF_3_) with D_5h_‐C_80_ and I_h_‐C_80_ carbon cages featuring the 3.78–3.79 Å long single‐electron Nd─Nd bond are synthesized by electrophilic trifluoromethylation of Nd_2_@C_80_ anions. The mutual influence of the exohedral CF_3_ group and endohedral metal dimer in determining the molecular structure of the adducts is analyzed.

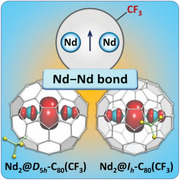

## 1 Introduction

Stabilization of unconventional metallic species and thereby a broadening of the metallochemistry frontiers is one of the unique features of endohedral metallofullerenes (EMFs).^[^ ^1^ ^]^ A spectacular example of this sort is the realization of the lanthanide–lanthanide bonding within the fullerene cage,^[^ ^2^ ^]^ which for a long time deemed not possible in conventional lanthanide chemistry. M_2_@C_79_N (M = Y, Tb, Gd) azafullerenes with single‐electron metal–metal bonds were discovered in 2008–2011.^[^ ^3^ ^]^ In parallel, an understanding that two‐electron metal–metal bonds are quite ubiquitous for dimetallofullerenes (di‐EMFs) of Lu, Er, and Y was developed in a series of experimental and computational studies.^[^ ^2^, ^4^ ^]^ Single‐electron lanthanide–lanthanide bonds were also stabilized in a series of M_2_@C_80_R derivatives (R = CH_2_Ph, CF_3_).^[^ ^5^ ^]^ Three‐center metal–metal bonding was suggested first in trimetallofullerenes M_3_@C_2n_ based mainly on computational studies,^[^ ^6^ ^]^ and was then found experimentally in carbide clusterfullerenes M_3_C_2_@C_80_ (M = Dy, Er).^[^ ^7^ ^]^ Single‐electron bonds in M_2_@C_79_N and M_2_@C_80_R are especially interesting as the way to create very strong magnetic interactions between 4f magnetic moments,^[^ ^3^, ^5^, ^8^, ^9^ ^]^ leading to high‐performance single molecule magnets.^[^ ^5^, ^10^ ^]^ The first non‐fullerene complexes with two and three‐center single electron bonds between lanthanides were also obtained recently.^[^ ^11^ ^]^

**Figure 1:**
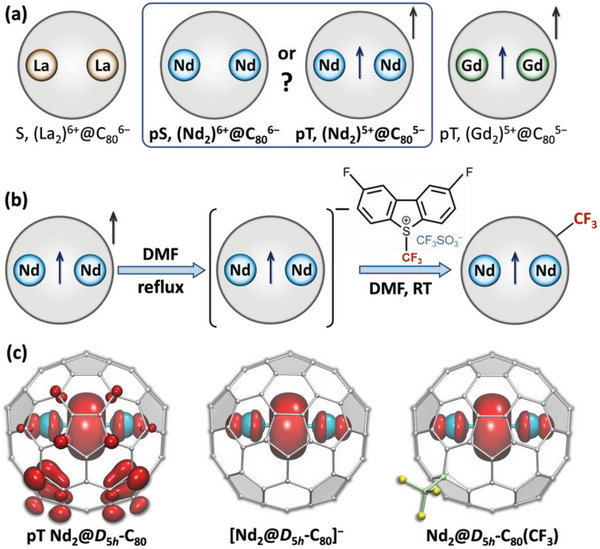
a) Schematic description of the ground‐state electronic structure of dimetallofullerenes M_2_@C_80_: singlet (S) La_2_@C_80_ with trivalent La, pseudo‐triplet (pT) Gd_2_@C_80_ with (Gd_2_)^5+^ dimer and an open‐shell structure of the fullerene, and the two corresponding possibilities for Nd_2_@C_80_. b) Schematic description of the extraction of Nd_2_@C_80_ (along with other EMFs) by hot DMF in the form of [Nd_2_@C_80_]^−^ anion, followed by the reaction with Umemoto reagent II with formation of the Nd_2_@C_80_(CF_3_) adduct. c) DFT‐computed valence spin density distribution in Nd_2_@D_5h_‐C_80_ (pseudo‐triplet), [Nd_2_@D_5h_‐C_80_]^−^ anion, and Nd_2_@D_5h_‐C_80_(CF_3_). Similar spin density distributions are obtained for the I_h_‐C_80_ cage isomer (Figure S2, Supporting Information).

Formation of M─M bonds in di‐EMFs is achieved by the guest‐to‐host electron transfer as schematically shown in **Figure** **1**a. Some fullerene cages, such as I_h_‐C_80_, are efficient acceptors of six electrons, and when dimers of early lanthanides La and Ce are encapsulated within, they readily transfer all six valence electrons to the fullerene host. The La_2_@C_80_ molecule has the singlet (S) ground state, and no La─La bond is present. For heavier lanthanides, such as Gd and beyond, the metal dimer orbitals have lower energies, and only five electrons are transferred to the fullerene. M_2_@C_80_ molecules with such metals feature a single‐electron M─M bond, while the carbon cage has an open‐shell electronic structure with one “missing” electron. For Y and heavy lanthanides, this pseudo‐triplet electronic state (denoted as pT hereafter) is lower in energy than the pseudo‐singlet (pS) with six‐electron transfer (we use prefix “pseudo” to underline that spins of 4f electrons are not considered and thus the true spin multiplicity of the molecule can be different).^[^ ^5^, ^12^ ^]^ The switch between pS and pT ground states occurs somewhere between Ce and Gd, but di‐EMFs of light lanthanides other than La and Ce are not well studied. Sm and Eu prefer divalent state in EMFs, and reported Sm di‐EMFs do not feature Sm─Sm bonds.^[^ ^13^ ^]^ Pr and Nd adopt the Ln^III^ state in mono‐EMFs, but not much is known about their di‐EMFs so far. An early report showed that Pr_2_@C_80_ can be extracted from the EMF soot and proved the Pr^III^ state by XPS technique,^[^ ^14^ ^]^ whereas more recent studies addressed the structure and properties of Pr_2_@C_72_.^[^ ^15^ ^]^ To the best of our knowledge, there are no reports on Nd‐based di‐EMFs at all, in contrast to the relatively well studied mono‐EMF Nd@C_82_.^[^ ^16^ ^]^

In this work, we aim at the understanding of the metal–metal bonding in light lanthanides and focus on Nd as the lanthanide with the least studied di‐EMFs. We demonstrate that while neutral Nd_2_@C_80_ is non‐accessible for the analysis in the pristine state, it can be stabilized as an anion and in the form of CF_3_ mono‐adduct. Two isomers of Nd_2_@C_80_(CF_3_) with D_5h_ and I_h_ cage symmetry are characterized by single‐crystal X‐ray diffraction, proving the ability of Nd to form single‐electron Nd─Nd bonds. The factors affecting regioselectivity of electrophilic CF_3_ addition to EMF anions are analyzed. Magnetic properties of Nd_2_@C_80_(CF_3_) are studied by SQUID (superconducting quantum interference device) magnetometry to understand if the single‐electron Nd─Nd bond leads to the single‐molecule magnetism.

## 2 Results and Discussion

### 2.1 Electronic Structure of M_2_@C_80_

Metallofullerenes are normally obtained by arc‐discharge evaporation of graphite followed by solvent extraction from the soot, but their solubility depends on the electronic state. Those with the closed‐shell electronic structure are usually soluble in CS_2_ and aromatic solvents. Open‐shell EMFs show low to negligible solubility in CS_2_ and mainly remain in the soot after CS_2_ extraction because of polymerization or interaction with soot particles. However, low‐gap and open‐shell fullerenes can be dissolved in boiling dimethylformamide (DMF), which is a poor solvent for empty fullerenes.^[^ ^17^ ^]^ During the DMF extraction, such fullerenes are reduced and extracted in the form of anions, which are well‐soluble in polar DMF (Figure 1b),^[^ ^18^ ^]^ unlike the closed‐shell fullerenes with large HOMO–LUMO gap, which remain neutral and hence poorly soluble in DMF (HOMO and LUMO refer to highest‐occupied and lowest‐unoccupied molecular orbitals). Particularly for M_2_@C_80_ di‐EMFs, the extraction with CS_2_ and DMF gives the first indication on the electronic structure. While singlet La and Ce di‐EMFs are soluble in CS_2_, pseudo‐triplet M_2_@C_80_ di‐EMFs of heavy lanthanides starting from Gd can be only extracted by DMF.^[^ ^5^, ^19^ ^]^

**Figure 2:**
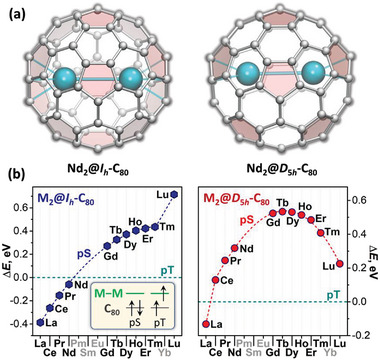
a) Molecular structure of Nd_2_@C_80_ isomers with I_h_ and D_5h_ cage symmetry. Both molecules are shown along the *C*_5_ axis of the carbon cage; pentagons are shaded to highlight different relative orientations of upper and lower hemispheres in I_h_ and D_5h_ isomers. b) Relative energies of pseudo‐singlet (pS) state of M_2_@C_80_ molecules versus pseudo‐triplet (pT) states as predicted by DFT calculations at the PBE0//PBE level (I_h_‐C_80_ isomer—left, D_5h_‐C_80_ isomer—right). Four lanthanides were not considered in calculations, because Pm is not available for experimental work, while Sm, Eu, and Yb adopt 2+ oxidation state in EMFs.

To identify a transition point between the two types, we first perform a systematic DFT (density functional theory) study of M_2_@C_80_ molecules in pS and pT states for the whole lanthanide row using two fullerene isomers with I_h_ and D_5h_ cage symmetry (**Figure** **2**). Calculations showed that in the M_2_@I_h_‐C_80_ series, the pT state is gradually stabilized with the growth of the atomic number (Figure 2b and Table S1a, Supporting Information). The pS state is more stable for La–Nd, whereas the pT state is more preferable from Gd to Lu. In the M_2_@D_5h_‐C_80_ series, the fullerene‐based HOMO has higher energy than in I_h_ isomers, and the pT state is predicted to be more stable for all lanthanides except for La. However, as the M─M bonding MO gets stabilized along the lanthanide row, its energy gap to the fullerene‐based HOMO becomes very small, and the orbitals start mixing (Figure S1, Supporting Information). For pS‐Lu_2_@C_80_, the occupied orbital becomes of the dominant Lu─Lu bonding nature, whereas the LUMO acquires a fullerene‐based character. This orbital quasi‐degeneracy implies a multiconfigurational electronic state and may be problematic for DFT. Nevertheless, the general trend of the gradual stabilization of pT along the lanthanide row holds and qualitatively agrees with experimental observations, although the numerical values may be not very accurate.

In the M_2_@I_h_‐C_80_ series, Nd is particular close to the transition between the two types of behavior and therefore appears to be an interesting target for the synthesis. DFT PBE0//PBE calculations predict that pS and pT states of Nd_2_@I_h_‐C_80_ are nearly degenerate, the former being 0.06 eV more stable (see Table S1b, Supporting Information, for calculations with other DFT functionals). In the closed‐shell pS state, the HOMO is localized on the fullerene whereas the LUMO is the Nd─Nd bonding orbital. In the pT state, one electron from the fullerene orbital is promoted to the Nd─Nd bonding orbital (see inset in Figure 2b). The valence spin density in pT‐Nd_2_@C_80_ is thus localized both on the Nd─Nd bonding MO and on the fullerene cage (Figure 1c and Figure S2, Supporting Information).

### 2.2 Synthesis of Nd_2_@C_80_

The soot containing Nd‐EMFs was obtained by arc‐discharge evaporation of graphite rods filled with a mixture of Nd_2_O_3_ and graphite powders under helium atmosphere (180 mbar). Extraction behavior of Nd di‐EMFs appeared similar to those of heavy lanthanides. We could not detect Nd_2_@C_80_ in mass‐spectra of CS_2_ extracts, but its signal was readily observed in mass‐spectra after extraction with DMF (Figures S3 and S4, Supporting Information). Furthermore, our attempts to obtain neutral Nd_2_@C_80_ by a treatment of the anionic Nd‐EMF extract with CHCl_2_COOH in acetone could only recover neutral Nd@C_82_. For comparison, an analogous reaction with La‐EMF anions produced both La@C_82_ and La_2_@C_80_.^[^ ^20^ ^]^ Thus, we conclude that Nd_2_@C_80_ is not stable in the neutral form, suggesting its pT ground state. This open‐shell electronic structure is responsible for the low solubility of neutral Nd_2_@C_80_ and its facile reduction to a monoanionic state. In the [Nd_2_@C_80_]^−^ anion, the fullerene cage attains a closed‐shell electronic configuration, while the Nd─Nd bonding orbital still hosts an unpaired electron (Figure 1c and Figure S2, Supporting Information). Interestingly, CS_2_ extraction of Pr‐EMFs obtained in a similar arc‐discharge procedure did produce Pr_2_@C_2n_ species detectable in mass‐spectra (Figure S3, Supporting Information), in line with the earlier report on the isolation of Pr_2_@C_80_.^[^ ^14^ ^]^ These results demonstrate that the transition from the pS to the pT ground state of M_2_@C_80_ occurs between Pr and Nd.

### 2.3 Electrophilic Trifluoromethylation of [Nd_2_@C_80_]^−^

**Figure 3:**
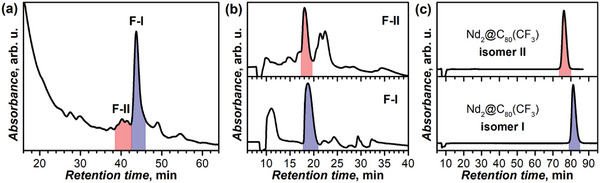
a) HPLC chromatogram of the crude product mixture after reaction of Nd‐metallofullerene DMF extract with Umemoto reagent II (linear combination of two Buckyprep columns, toluene, 5 mL min^−1^); the fractions highlighted by color and denoted as F‐I and F‐II contain isomers of Nd_2_@C_80_(CF_3_) and are separated further; b) The second step separation of fractions F‐I and F‐II on a Buckyprep‐D column (toluene, 2 mL min^−1^), fractions with Nd_2_@C_80_(CF_3_) isomers are highlighted by color. c) HPLC chromatograms of purified Nd_2_@C_80_(CF_3_) isomers (Buckyprep column, toluene, 2 mL min^−1^). Note that the separation of isomer II required an additional HPLC step to remove minor impurity as described in Figure S6, Supporting Information.

**Figure 4:**
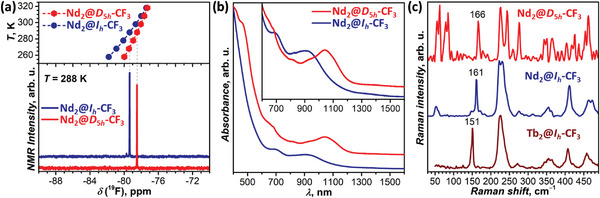
a) ^19^F NMR spectra of two Nd_2_@C_80_(CF_3_) isomers in CS_2_ solution at 288 K, and the temperature dependence of ^19^F chemical shifts between 258 and 318 K. b) Vis‐NIR absorption spectra of two Nd_2_@C_80_(CF_3_) isomers in toluene solution. c) Raman spectra of two Nd_2_@C_80_(CF_3_) isomers compared to the spectrum of Tb_2_@I_h_‐C_80_(CF_3_) (*T* = 78 K, excitation at 532 nm).

To stabilize Nd_2_@C_80_ in the form of a neutral compound, electrophilic trifluoromethylation with 2,8‐difluoro‐S‐(trifluoromethyl)dibenzothiophenium trifluoromethanesulfonate (Umemoto reagent II,^[^ ^21^ ^]^ URII) was performed. The reaction can be formally described as a [CF_3_]^+^ cation addition to [EMF]^−^ anions, giving neutral EMF(CF_3_) adducts (Figure 1b). We recently found that at the first stage, the reaction of Tb‐EMFs with URII exhibited enhanced selectivity to [Tb_2_@I_h_‐C_80_]^−^.^[^ ^5^ ^]^ Likewise, optimization of the URII amount in the reaction with Nd‐EMF extract in DMF allowed to obtain Nd_2_@C_80_(CF_3_) in this work (Figure S5, Supporting Information). When optimized reagent ratio is used, HPLC (high‐performance liquid chromatography) profile of the crude reaction product mixture has a dominant fraction in the retention time range of EMF(CF_3_) monoadducts (F‐I in **Figure** **3**a), which mainly contains Nd_2_@C_80_(CF_3_). The presence of Nd_2_@C_80_(CF_3_), identified as a different isomer, was also found by mass‐spectrometry in the preceding fraction (F‐II in Figure 3a). Both fractions were then subjected to the second HPLC step with a different column, which gave pure isomer I and almost pure isomer II (Figure 3b). For the latter, an additional step was required to remove some minor admixtures (Figure S6, Supporting Information). The isolated isomers of Nd_2_@C_80_(CF_3_) were then characterized by matrix‐assisted laser desorption/ionization mass‐spectrometry (Figure S7, Supporting Information) and a variety of spectroscopic techniques (**Figure** **4**; Figures S8 and S9, Supporting Information). To accumulate sufficient amount of material, 40 arc‐discharge evaporations were performed, consuming 68 g of Nd_2_O_3_ and yielding eventually 2.5 mg of the major Nd_2_@C_80_(CF_3_) isomer. Perfluoroalkylfullerenes usually bear even number of addends,^[^ ^22^ ^]^ and the odd number of CF_3_ groups in Nd_2_@C_80_(CF_3_) indicates that these adducts have metal‐based open‐shell character. Indeed, DFT calculations discussed in detail below demonstrate the presence of a single‐electron Nd─Nd bond in Nd_2_@C_80_(CF_3_) (Figure 1c and Figure S2, Supporting Information).

**Figure 5:**
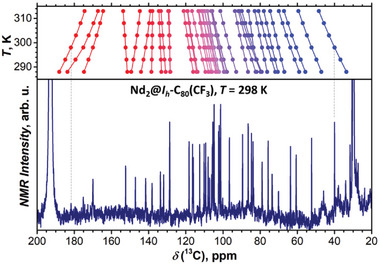
^13^C NMR spectrum of Nd_2_@I_h_‐C_80_(CF_3_) in CS_2_ measured at 298 K (lower panel) and temperature dependence of chemical shifts assigned to the fullerene carbons (upper panel).

^19^F NMR (nuclear magnetic resonance) spectra of each Nd_2_@C_80_(CF_3_) isomer at 298 K have only one signal at −78.65 (I) and −78.05 ppm (II), proving their isomeric purity (Figure 4a). Both isomers exhibit considerable temperature dependence of their ^19^F chemical shifts caused by the magnetic influence of endohedral Nd_2_ dimers on the ^19^F nuclear spins of CF_3_ groups. However, the paramagnetic shift of Nd_2_@C_80_(CF_3_) is much weaker than in Tb_2_@I_h_‐C_80_(CF_3_), which exhibits *δ*(^19^F) of −251 ppm at 298 K.^[^ ^5^ ^]^ Note that a conservative estimation of the diamagnetic shift based on the *T* ^−2^ dependence of *δ*(^19^F) gives the values near −70 ppm. Carbon chemical shifts in ^13^C NMR spectra of isomer I (**Figure** **5**) were found in the range of 30–190 ppm, some of them with considerable temperature dependence. The number of detectable signals, around 40, is consistent with *C*_s_ effective symmetry of the adduct.

Vis‐NIR (visible‐near infrared) absorption spectra of two isomers are distinctly different (Figure 4b). That of the main isomer I resembles the spectra of Tb_2_@I_h_‐C_80_(CF_3_)^[^ ^5^ ^]^ and M_2_@I_h_‐C_80_(CH_2_Ph),^[^ ^5^ ^]^ whereas the absorption pattern of isomer II is similar to the spectrum of La_2_@D_5h_‐C_80_(C_3_N_3_‐Ph_2_).^[^ ^5^ ^]^ Given that absorption spectra of EMFs and their derivatives are usually weakly influenced by their endohedral species and exohedral groups but are chiefly determined by the fullerene π‐system topology, the fullerene cage isomers I_h_‐C_80_ and D_5h_‐C_80_ can be assigned to Nd_2_@C_80_(CF_3_) isomers I and II, respectively.

The structural conjecture is further corroborated by vibrational spectroscopy. Infrared spectrum of Nd_2_@C_80_(CF_3_)‐I is virtually identical to the spectrum of Tb_2_@I_h_‐C_80_(CF_3_) from ref. [5e] (Figure S8, Supporting Information), both in the position and relative intensity of strong CF_3_‐related modes at 946, 1174–1196, and 1222 cm^−1^, and in the absorption pattern of weaker cage‐based vibration, which suggests that the two compounds are isostructural. Strong CF_3_ absorption features in the spectrum of Nd_2_@C_80_(CF_3_)‐II are found at similar positions (943, 1174–1196, and 1237 cm^−1^) as in Nd_2_@C_80_(CF_3_)‐I, but the spectra are quite different in the range of the fullerene cage modes. Likewise, Raman spectra of Nd_2_@C_80_(CF_3_)‐I and Tb_2_@I_h_‐C_80_(CF_3_) are very similar above 200 cm^−1^ (Figure S9, Supporting Information), but are distinct at lower frequencies, where metal‐based modes occur (Figure 4c). Particular characteristic is the metal‐cage stretching mode, which in lanthanide dimetallofullerenes usually occurs at 140–170 cm^−1^, has medium‐strong Raman intensity and a narrow linewidth. For instance, in La_2_@C_80_ it occurs at 163 cm^−1^,^[^ ^23^ ^]^ in Gd_2_@C_79_N it was found at 156 cm^−1^,^[^ ^3^ ^]^ while in the M_2_@C_80_(CH_2_Ph) series the position varies from 151 cm^−1^ for Gd to 144 cm^−1^ for Er.^[^ ^5^ ^]^ in Nd_2_@C_80_(CF_3_)‐I this mode occurs at 161 cm^−1^, comparable to La_2_@C_80_, as Nd is relatively light. In Tb_2_@I_h_‐C_80_(CF_3_) this vibration is shifted to 151 cm^−1^ owing to the larger mass of Tb. The metal‐based mode of Nd_2_@C_80_(CF_3_)‐II occurs at somewhat higher frequency (166 cm^−1^) than in the isomer I, while vibrational pattern of the fullerene cage is again quite different.

### 2.4 Crystallographic Analysis

**Figure 6:**
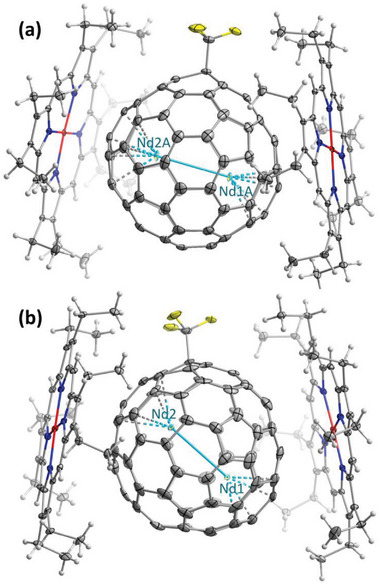
Molecular structures of a) isomer I, Nd_2_@I_h_‐C_80_(CF_3_), and b) isomer II, Nd_2_@D_5h_‐C_80_(CF_3_), in co‐crystals with NiOEP. Only single Nd_2_@C_80_(CF_3_) moiety with the main Nd sites and two clamping NiOEP molecules are shown for each co‐crystal, see Supporting Information for further details. Nd site occupancies are 0.72/0.72 for Nd1A and Nd2A in (a), and 0.22/0.26 (out of 0.5) for Nd1/Nd2 in (b). The displacement parameters are shown at the 30% probability level. Color code: grey for carbon, cyan for Nd, yellow for F, blue for N, white for H, and red for Ni.

An unambiguous elucidation of molecular structures was obtained by single‐crystal X‐ray diffraction (SC‐XRD) (**Figure** **6**). Nd_2_@C_80_(CF_3_) in toluene (isomer I) or CS_2_ (isomer II) was co‐crystallized with nickel octaethylporphyrin (NiOEP) in benzene. X‐ray diffraction data collection was carried out at 100 K at the BESSY storage ring (BL14.2, Berlin‐Adlershof, Germany).^[^ ^24^ ^]^ XDSAPP2.0 suite was employed for data processing.^[^ ^25^ ^]^ The structure was solved by direct methods and refined by SHELXL‐2018.^[^ ^26^ ^]^ Hydrogen atoms were added geometrically and refined with a riding model. The crystal data are presented in Table S2 (Supporting Information).

**Figure 7:**
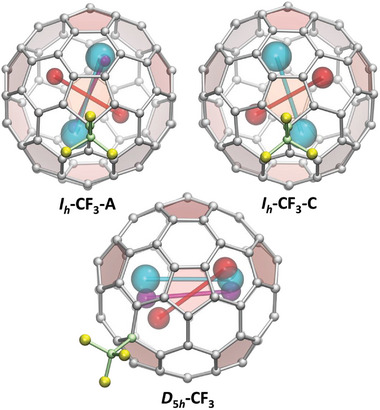
SC‐XRD molecular structures of Nd_2_@C_80_(CF_3_) isomers showing disorder of Nd positions. I_h_‐CF_3_‐A: molecule A of Nd_2_@I_h_‐C_80_(CF_3_), Nd_2_ sites are cyan (Nd_1_A/Nd_2_A, 2 × 0.72), red (Nd_3_A/Nd_2_A, 2 × 0.20), and purple (Nd5A/Nd6A, 2 × 0.07). I_h_‐CF_3_‐C: molecule C of Nd_2_@I_h_‐C_80_(CF_3_), Nd_2_ sites are cyan (Nd1C/Nd2C, 2 × 0.36) and red (Nd3C/Nd4C, 2 × 0.14), overlapping fullerene molecule and symmetry related Nd sites are not shown. D_5h_‐CF_3_: Nd_2_@D_5h_‐C_80_(CF_3_), Nd_2_ sites are cyan (Nd1/Nd2, 0.21/0.26), red (Nd3/Nd4, 0.17/0.15), and purple (Nd5/Nd6, 0.11/0.09), overlapping fullerene molecule and symmetry related Nd sites are not shown. The volume of spheres representing metal atoms scales as their relative occupancy.

The isomer Nd_2_@C_80_(CF_3_)‐I features I_h_(7)‐C_80_ carbon cage and forms monoclinic crystals (C2/c space group) with the composition of 2Nd_2_@I_h_‐C_80_(CF_3_)·4NiOEP·1.63C_7_H_8_·0.37C_6_H_6_, closely resembling the crystal structure of Tb_2_@I_h_‐C_80_(CF_3_).^[^ ^5e^ ^]^ The asymmetric unit contains four intact NiOEP molecules exhibiting some degree of translational disorder, one ordered Nd_2_@I_h_‐C_80_(CF_3_) molecule (site A), four halves of fullerene molecules statistically disordered over two orientations with half occupancies (sites B and C), and disordered benzene/toluene molecules. Four halves of disordered fullerene molecules are correlated by the *C*_2_ axis of the crystal passing through the molecule (roughly parallel to the C_80_─CF_3_ bond). The Nd_2_ dimer is disordered over three positions with occupancies of 0.72, 0.20, and 0.07 in the site A, and over two positions with occupancies of 0.36 and 0.14 in each of the sites B and C (**Figure** **7** and Figure S10, Supporting Information). The Nd─Nd bond lengths determined for the major Nd_2_ sites are 3.792(2), 3.791(2), and 3.778(2) Å in molecules A, B, and C, respectively. In all the major Nd_2_ sites, Nd atoms coordinate fullerene hexagons in η^6^‐manner, with the shortest Nd─C distances of 2.39–2.44 Å.

The isomer Nd_2_@C_80_(CF_3_)‐II has D_5h_(6)‐C_80_ carbon cage and forms co‐crystals with the Nd_2_@D_5h_‐C_80_(CF_3_)·2NiOEP·C_6_H_6_ composition and C2/c space group. The asymmetric unit contains one intact NiOEP molecule, two halves of fullerene molecules with half occupancy correlated by the crystallographic *C*_2_ axis passing through the fullerene molecules, and one intact benzene molecule. The Nd_2_ dimer is disordered over three positions (Figure 7) and their symmetry replica, the Nd─Nd bond lengths being 3.788(2) Å for Nd1–Nd2 (occupancy 0.21/0.26), 3.780(3) Å for Nd3–Nd4 (0.15/0.17), and 3.790(4) Å for Nd5–Nd6 (0.11/0.09). In the major Nd_2_ site, Nd atoms also tend to coordinate with fullerene hexagons but are displaced from the center, so that hapticity is better described as η^4^.

In both crystal structures, NiOEP molecules show translational disorder over two sites with the major site occupancies of 0.92–0.95 for Nd_2_@I_h_‐C_80_(CF_3_) and 0.85 for Nd_2_@D_5h_‐C_80_(CF_3_) (Figure S12, Supporting Information). Similar phenomenon was described in the NiOEP co‐crystals of Sc_3_N@*C*_2v_(7854)‐C_70_^[^ ^27^ ^]^ and Tb_2_@I_h_(7)‐C_80_(CF_3_).^[^ ^5e^ ^]^ Besides, in both crystals the Nd_2_@C_80_(CF_3_) moiety is clamped by two NiOEP molecules (Figure 6), which is a rather rare motif in fullerene·NiOEP co‐crystals^[^ ^5^, ^28^ ^]^ as fullerenes usually prefer 1:1 complexation with octaethylporphyrin.

Comparison of Nd_2_@C_80_(CF_3_) to other M_2_@C_80_ di‐EMFs with single‐electron lanthanide–lanthanide bonds characterized by SC‐XRD (Table S3, Supporting Information) shows that the Nd─Nd bond length of 3.78–3.79 Å is very similar to La─La bonds (3.78–3.79 Å),^[^ ^5b^ ^]^ but shorter than Gd─Gd (3.83 Å),^[^ ^9a^ ^]^ Tb─Tb (3.84–3.90 Å),^[^ ^3^, ^5^ ^]^ and Dy─Dy bonds (3.89–3.90 Å).^[^ ^5^, ^10^ ^]^ A systematic increase of the M─M bond distance in the lanthanide row despite the lanthanide contraction is caused by the rigid size of the C_80_ fullerene cage and shortening of optimal M─C bond lengths, which inevitably leads to the increase of the M─M distance (see Table S4, Supporting Information, for selected M─C bond lengths from DFT calculations).

### 2.5 CF_3_ Addition Sites and Endohedral Positions of Nd_2_ Dimer

Because of its high symmetry, the I_h_‐C_80_ cage has only two types of carbons, 20 located on triple‐hexagon junctions (THJ) and 60 on pentagon–hexagon–hexagon junctions (PHHJ). Addition of radical groups to THJ carbons is usually strongly energetically unfavorable in comparison to the PHHJ counterparts.^[^ ^22^ ^]^ Thus, in accordance with the THJ‐avoidance rule, the CF_3_ group in Nd_2_@I_h_‐C_80_(CF_3_) is attached to a PHHJ carbon as in the isostructural Tb_2_@I_h_‐C_80_(CF_3_).^[^ ^5e^ ^]^ DFT calculations for different orientations of the Nd_2_ dimer inside the I_h_‐C_80_(CF_3_) moiety revealed the pattern similar to the observed in M_2_@C_80_(R) adducts of heavy lanthanides (R = CF_3_, CH_2_Ph).^[^ ^5c–e^ ^]^ Namely, the Nd_2_ dimer tends to avoid the C‐sp^3^ carbon at the CF_3_ addition site and adopts positions along the belt of hexagons near the plane, located roughly perpendicular to the C_80_–CF_3_ bond (Figure S14, Supporting Information). Several conformers of this sort found in our calculations are isoenergetic within few kJ mol^−1^ (Figure S14, Supporting Information), indicating that the Nd_2_ dimer is likely to experience a quasi‐2D rotation. The Nd_2_ sites in the SC‐XRD structure of Nd_2_@I_h_‐C_80_(CF_3_) correspond to some of these conformers (Figure 7; Figures S15 and S16, Supporting Information), and their particular positions are determined by interactions with NiOEP molecules since all Nd sites in the experimental structure are found close to Ni─N bonds of nearby porphyrins (Figures S11,  S15, and S16, Supporting Information).

As the high symmetry of I_h_‐C_80_ leaves essentially no alternatives for CF_3_ addition site in Nd_2_@I_h_‐CF_3_, it cannot be concluded for certain whether the orientation of the Nd_2_ dimer in [Nd_2_@I_h_‐C_80_]^−^ determines which of 60 PHHJ carbons acts as the addition site of the CF_3_ group, or the CF_3_ group first adds to a random PHHJ position, and then the Nd_2_ dimer adjusts its orientation. The lower symmetry of the D_5h_‐C_80_ cage gives more room for such an analysis. Notably, D_5h_‐C_80_ isomers of lanthanide di‐EMFs are less abundant than I_h_‐C_80_, and information about their chemical properties is very scarce.^[^ ^5^, ^29^ ^]^ Nd_2_@D_5h_‐C_80_(CF_3_) obtained in this work is only the second example of the di‐EMF derivatives with this cage.

**Figure 8:**
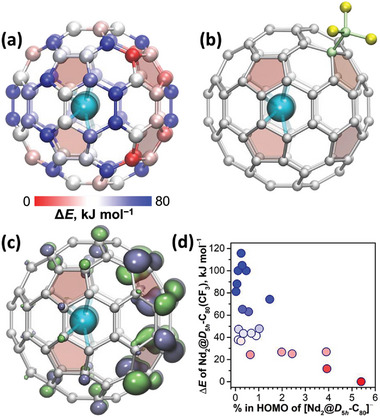
a) Nd_2_@D_5h_‐C_80_ with carbon atoms colored according to the stability of corresponding Nd_2_@C_80_(CF_3_) isomers (positions with Δ*E* > 80 kJ mol^−1^ are all blue). b) The most stable isomer of Nd_2_@D_5h_‐C_80_(CF_3_) according to DFT calculations. c) Isosurface of the α‐HOMO of [Nd_2_@D_5h_‐C_80_]^−^; note that spin‐up (α) and spin‐down (β) counterparts of the HOMO are identical (hence only α part is plotted), whereas the singly‐occupied Nd─Nd bonding orbital is α‐HOMO–1 and has lower energy than the fullerene‐based α‐HOMO. d) Correlation between contributions of carbons to the α‐HOMO of [Nd_2_@D_5h_‐C_80_]^−^ and relative energies of corresponding Nd_2_@C_80_(CF_3_) isomers.

D_5h_‐C_80_ has six types of carbon atoms (four PHHJ types and two THJ types), but the endohedral metal dimer will reduce the symmetry and increase the number of non‐equivalent carbons. Therefore, we first performed DFT calculations for [Nd_2_@D_5h_‐C_80_]^−^ anion with various orientations of the Nd_2_ dimer to find the most preferable metal positions. The structure of the lowest‐energy conformer is shown in Figure 1c and **8**a, while five other conformers appeared less stable by 22–57 kJ mol^−1^ (Figure S17, Supporting Information). A similar position of Ce atoms was found earlier in the most stable conformer of Ce_2_@D_5h_‐C_80_.^[^ ^29^, ^30^ ^]^ Interestingly, despite the *C*_2v_ point symmetry of this structure, the ^13^C NMR study of Ce_2_@D_5h_‐C_80_ demonstrated the effective D_5h_ cage symmetry in solution at 280–300 K, proving the rotation of the metal dimer inside the fullerene near room temperature on the NMR timescale.^[^ ^29^ ^]^

Having established the most stable structure of [Nd_2_@D_5h_‐C_80_]^−^, we optimized all 22 regioisomers of Nd_2_@D_5h_‐C_80_(CF_3_) obtained by addition of a CF_3_ group to this conformer (Figure 8a). This survey showed that the isomer corresponding to the experimental SC‐XRD structure has the lowest energy (Figure 8b). It has CF_3_ group in the same position, to which the radical group was added in La_2_@D_5h_‐C_80_(C_3_N_3_‐Ph_2_).^[^ ^5b^ ^]^ The second most stable isomer is at 12 kJ mol^−1^, and all the others span the relative energy range of 24–120 kJ mol^−1^ (Figure 8a and Figure S18, Supporting Information). To find the rationale for the enhanced stability of the experimental Nd_2_@D_5h_‐C_80_(CF_3_) regioisomer, we analyzed structural and electronic properties of [Nd_2_@D_5h_‐C_80_]^−^. Neither atomic charges nor pyramidalization of carbons in pristine [Nd_2_@D_5h_‐C_80_]^−^ exhibited discernible correlation with the relative energy of Nd_2_@D_5h_‐C_80_(CF_3_) regioisomers. More revealing appeared the MO analysis, which showed that the most stable regioisomers have CF_3_ group on the carbons with the largest contribution to the HOMO of [Nd_2_@D_5h_‐C_80_]^−^ (Figure 8c,d). The same atoms also have the largest spin populations in the pseudo‐triplet state of the neutral Nd_2_@D_5h_‐C_80_ (Figure 1c).

Calculations also showed that the CF_3_ addition to positions equivalent in the empty D_5h_‐C_80_ cage, but inequivalent when the metal dimer is considered in Nd_2_@D_5h_‐C_80_, leads to structures with substantially different stability and the relative energy spread of 48 kJ mol^−1^ (Figure S18, Supporting Information). As the Nd_2_ dimer did not undergo noticeable rearrangements in the course of optimization, another series of calculations was performed, in which the CF_3_ position was fixed to the experimental one, while the orientation of the metal dimer was varied. These calculations localized 11 conformers of Nd_2_@D_5h_‐C_80_(CF_3_) (Figure S19, Supporting Information), twice more than for the bare [Nd_2_@D_5h_‐C_80_]^−^ anion. Nonetheless, position of the Nd_2_ dimer in the lowest‐energy conformer of Nd_2_@D_5h_‐C_80_(CF_3_) (Figure 8b) remained the same as in the most stable conformer of [Nd_2_@D_5h_‐C_80_]^−^, whereas other Nd_2_ orientations increase the energy by 24–70 kJ mol^−1^. Thus, rotation of Nd_2_ in Nd_2_@D_5h_‐C_80_(CF_3_) is considerably hindered in comparison to Nd_2_@I_h_‐C_80_(CF_3_). As electrostatic stabilization of metal cations inside negatively charged carbon cages is one of the factors determining stability of metallofullerenes, we also analyzed electrostatic potential (ESP) distribution inside [D_5h_‐C_80_]^6−^ and [D_5h_‐C_80_(CF_3_)]^5−^ (Figure S20, Supporting Information). While ESP distribution in the non‐functionalized fullerene is quite uniform and has nearly spherical shape without well‐localized minimum, addition of CF_3_ group results in considerable inhomogeneity of ESP in [D_5h_‐C_80_(CF_3_)]^5−^. Importantly, the minimum of ESP in the latter is close to the position of one of Nd atoms in the lowest‐energy conformer of Nd_2_@D_5h_‐C_80_(CF_3_).

Comparison of the experimental positions of Nd atoms in the SC‐XRD structure of Nd_2_@D_5h_‐C_80_(CF_3_) is complicated by the overlap of two fullerene orientations, which doubles the number of possible orientations for each Nd_2_ site and precludes their ascribing to the particular cage orientation solely on the base of SC‐XRD data (Figure S21, Supporting Information). Careful analysis augmented with DFT calculations showed that Nd1–Nd2 and Nd5–Nd6 sites correspond to the lowest‐energy conformer of Nd_2_@D_5h_‐C_80_(CF_3_) (Figure S21, Supporting Information). The situation is more ambiguous for the Nd3–Nd4 site, but in one of the cage orientations metal atoms are also not strongly displaced from the most stable conformer (Figure 7 and Figure S22, Supporting Information). Based on these findings, we conclude that the Nd_2_ dimer in the experimental structure of Nd_2_@D_5h_‐C_80_(CF_3_) is located near the DFT‐predicted energy minimum, while the disorder of Nd sites reflects a large‐amplitude librational and translational motion of the Nd_2_ dimer around its optimal position. Such motions of metal atoms are quite common for metallofullerenes because, when metal atoms are encapsulated inside the fullerene cage, their translational degrees of freedom transform into vibrations with librational and translational character. Lateral vibrational modes, in which metal atoms are displaced parallel to the fullerene surface, occur at low frequencies (<100 cm^−1^), in part because metal atoms are heavy, and in part because potential energy surface for such displacements is often rather flat. The low frequencies imply the ease of thermal excitation and the large amplitude of such motions.

Extended DFT calculations described in this section prove that the experimental structure of Nd_2_@D_5h_‐C_80_(CF_3_) corresponds to the most thermodynamically stable isomer. However, it would be too hasty to conclude that the electrophilic CF_3_ addition to [M_2_@C_80_]^−^ proceeds under thermodynamic control. The latter implies that CF_3_ groups bonded to a fullerene can rearrange on its surface via detachment/attachment steps until the most thermodynamically favorable position is achieved. But CF_3_ derivatives of fullerenes are extremely thermally stable,^[^ ^22^ ^]^ and detachment of CF_3_ groups occurs only at high temperatures (*T* > 300°C).^[^ ^28^, ^31^ ^]^ It is therefore very unlikely that a CF_3_ group, once it formed a bond with a certain carbon atom of Nd_2_@C_80_, can rearrange to a different site at room temperature in solution. The correlation between the isomer stability and the shape of the HOMO suggests that thermodynamically favorable products of the electrophilic trifluoromethylation of [M_2_@C_80_]^−^ and presumably other EMF anions are also favorable kinetically. The spatial distribution of the HOMO, in its turn, is strongly related to the position of the metal dimer (Figure 8c), and it would be reasonable to conclude that the CF_3_ addition site is to a large extent controlled by the metal dimer. Even though the latter can often rotate inside a fullerene, its momentary position at the time of the attack will determine where the HOMO is localized and which carbons will act as CF_3_ addition sites.

### 2.6 Magnetic Properties of Nd_2_@I_h_‐C_80_(CF_3_)

**Figure 9:**
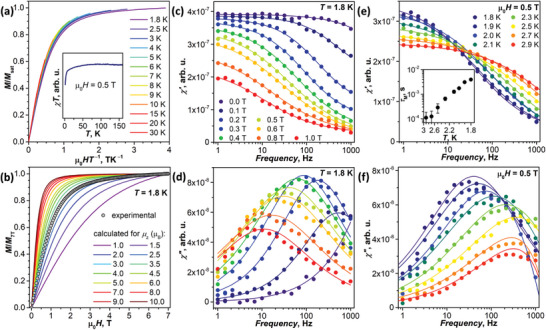
Magnetic properties of Nd_2_@I_h_‐C_80_(CF_3_). a) *MT* ^−1^ product at different temperatures, the inset shows *χT* curve measured in the field of 0.5 T. b) Calculated normalized magnetization curves for axially‐anisotropic molecules with different µ_z_ compared to the normalized experimental magnetization curve of Nd_2_@I_h_‐C_80_(CF_3_). c,d) In‐phase (*χ′*) and out‐of‐phase (*χ″*) magnetic susceptibility measured in different fields at 1.8 K. e,f) In‐phase (*χ′*) and out‐of‐phase (*χ″*) magnetic susceptibility measured at different temperatures in the field of 0.5 T; the inset in (e) shows the temperature dependence of relaxation times.

Single‐electron metal–metal bonds lead to strong exchange interactions in endohedral lanthanide dimers, which earlier resulted in dramatic improvement of the single‐molecule magnetism (SMM) in Dy_2_ and Tb_2_ compounds.^[^ ^5^, ^10^, ^11^ ^]^ To find if similar phenomena take place in Nd_2_, we studied magnetic properties of Nd_2_@I_h_‐C_80_(CF_3_) with SQUID magnetometry. *M* versus *HT* ^−1^ plots do not superimpose, indicating magnetic anisotropy and/or thermally accessible excited magnetic states (**Figure** **9**a). Unfortunately, the low available amount of the sample (≈2.5 mg) and its small magnetization prevent accurate determination of the magnetic moment from the saturated magnetization, and we had to rely on the shapes of magnetization curves for a conservative estimation. Figure 9b compares normalized magnetization curves (*M*/*M*_7T_) simulated for powder samples of axially anisotropic magnetic moments with *µ*_z_ ranging from 1 to 10 µ_B_. If, similar to Tb and Dy analogs,^[^ ^5^ ^]^ Nd ions in Nd_2_@I_h_‐C_80_(CF_3_) were having axial single‐ion magnetic anisotropy with the largest *J*_z_ projection of 9/2 and ferromagnetic coupling of collinear Nd and unpaired electron magnetic moments, the total moment of the molecule would be 7.545 µ_B_. However, experimental magnetization curve of Nd_2_@I_h_‐C_80_(CF_3_) closely follows the simulated one for *µ*_z_ of only 3 µ_B_ (Figure 9b). Several factors may contribute to such a small magnetic moment, including a weak exchange coupling, a small single‐ion anisotropy of Nd ions, a non‐collinear arrangement of the moments, or a combination of several of these factors. Deeper understanding of how magnetic moments of Nd ions interact in Nd_2_@I_h_‐C_80_(CF_3_) will require advanced computational ab initio modeling, which is beyond the scope of this work.

Nd_2_@I_h_‐C_80_(CF_3_) did not show magnetic hysteresis down to 1.8 K, and we performed AC measurement to understand if the relaxation of magnetization may still happen on a sub‐second time scale. At 1.8 K in zero static magnetic field, only a tail of the *χ*″ peak could be detected (Figure 9c,d), which means that the relaxation is too fast in the frequency range of 1000 Hz. However, an increase of the static field decelerated the process and shifted the *χ*″ peak to lower frequencies (Figure 9d). The temperature dependence measured in the static field of 0.5 T revealed a fast increase of the relaxation rate with temperature, giving the upper temperature limit of only 3 K (Figure 9e,f). Thus, Nd_2_@I_h_‐C_80_(CF_3_) exhibits properties of a weak field‐induced SMM akin to many other Nd‐SMMs,^[^ ^32^ ^]^ but the very limited temperature range does not allow us to analyze its relaxation mechanisms.

## 3 Conclusions

The ability of fullerenes to encapsulate metal dimers with M─M bonds is utilized to obtain the first instance of covalent Nd─Nd bond. First, we performed a systematic DFT study of lanthanide M_2_@C_80_ with different lanthanides and found that Nd_2_@C_80_ is on the border between two types of di‐EMFs. One type forms M_2_@C_80_ molecules with pseudo‐singlet ground state without M─M bonding, whereas another type features a single‐electron M─M bond in the pseudo‐triplet ground state. The synthesis of Nd EMFs with subsequent DMF extraction and reaction of the anionic extract with electrophilic “CF_3_^+^” reagent allowed isolation of two Nd_2_@C_80_(CF_3_) adducts. Crystallographic analysis determined that these compounds are based on I_h_‐C_80_ and D_5h_‐C_80_ cage isomers and both feature a single‐electron Nd─Nd bond with the length of ≈3.8 Å. Relatively low symmetry of Nd_2_@D_5h_‐C_80_ cage enabled analysis of the factors affecting regioselectivity of electrophilic trifluoromethylation of EMF anions and the mutual influence of CF_3_ and metal dimer. The influence of the Nd─Nd bond on magnetization dynamics was studied by SQUID magnetometry, which revealed that Nd_2_@I_h_‐C_80_(CF_3_) is a weak SMM. Its slow relaxation of magnetization could be detectable only below 3 K in the presence of magnetic field, while no magnetic hysteresis was observed. This behavior is quite different from Dy and Tb dimetallofullerenes with single‐electron metal–metal bond, showing that M─M bonds in light lanthanides may lead to different properties and require further studies.

## Conflict of Interest

The authors declare no conflict of interest.

## Supporting information

Supporting Information

Supporting Information

## Data Availability

The data that support the findings of this study are available in the supplementary material of this article.
