# Supplementary material for: Nd─Nd Bond in Ih and D5h Cage Isomers of Nd2@C80 Stabilized by Electrophilic CF3 Addition
Source: Adv Sci (Weinh). 2023 Nov 9;11(1):2305190. doi: 10.1002/advs.202305190 (PMC10767449; doi:10.1002/advs.202305190)
Supplement: Supplementary file 1 — Supporting Information [file ADVS-11-2305190-s001.pdf]

ADVANCED SCIENCE
Open Access

Supporting Information

for *Adv. Sci.*, DOI 10.1002/advs.202305190

Nd—Nd Bond in I_h and D_{5h} Cage Isomers of $Nd_2@C_{80}$ Stabilized by Electrophilic CF_3 Addition

Wei Yang, Georgios Velkos, Marco Rosenkranz, Sandra Schiemenz, Fupin Liu and Alexey A. Popov**

Supporting Information

Nd–Nd bond in I_h and D_{5h} cage isomers of $Nd_2@C_{80}$ stabilized by electrophilic CF_3 addition

Wei Yang, Georgios Velkos, Marco Rosenkranz, Sandra Schiemenz, Fupin Liu,* Alexey A. Popov*

Table of contents

Experimental details	S2
DFT calculations of pS and pT-$M_2@C_{80}$	S3
Spin density in $Nd_2@C_{80}$, $[Nd_2@C_{80}]^-$ and $Nd_2@C_{80}(CF_3)$	S6
Mass-spectra of Nd-EMF and Pr-EMF extracts	S7
Synthesis and separation of $Nd_2@C_{80}(CF_3)$ isomers	S9
Spectroscopic characterization of $Nd_2@C_{80}(CF_3)$ isomers	S12
Single-crystal XRD of $Nd_2@C_{80}(CF_3)$ isomers	S14
Metal-metal distances in lanthanide $M_2@C_{80}$ di-EMFs	S18
DFT calculations of $Nd_2@I_h$-$C_{80}(CF_3)$	S19
DFT calculations of $[Nd_2@D_{5h}$-$C_{80}]^-$ and $Nd_2@D_{5h}$-$C_{80}(CF_3)$	S21
References	S26

Experimental details

HPLC: HPLC analysis and separation were performed for toluene solutions of fullerene and with toluene as an eluent, employing semipreparative COSMOSIL Buckyprep and Buckyprep-D chromatographic columns (Nacalai Tesque) and Agilent 1260 Infinity II LC System. Recycling HPLC separation was performed using Sunflow 100 system (SunChrome).

Mass spectrometry: Matrix-assisted laser desorption/ionization time-of-flight (MALDI-TOF) mass-spectra were measured with a Bruker autoflex mass-spectrometer with 1,1,4,4-tetraphenyl-1,3-butadiene as a matrix.

NMR spectrometry: ^{19}F and ^{13}C NMR spectra were measured with 500 MHz Avance II spectrometer (Bruker) in CS_2 solution.

UV-Vis spectrometry: UV-vis-NIR absorption spectra were measured in CS_2 solution at room temperature with Shimadzu 3100 spectrophotometer.

Vibrational spectroscopy. FT-IR spectra were recorded at room temperature on a Vertex 80 FT-IR spectrometer (Bruker) equipped with Hyperion microscope. The samples were drop-casted from toluene solution onto KBr substrate and measured in transmission mode. The same samples but cooled down to 78 K were used in Raman measurements performed with T64000 spectrometer (Horiba) and laser excitation at 532 nm (Torus laser by Laser Quantum).

X-ray diffraction. Single crystal X-ray diffraction data collection was carried out at 100 K at the BESSY storage ring (BL14.2, Berlin-Adlershof, Germany).[1] XDSAPP2.0 suite was employed for data processing.[2, 3] The structure was solved by direct methods and refined by SHELXL-2018.[4] Hydrogen atoms were added geometrically and refined with a riding model.

Magnetic measurements. DC and AC magnetic measurements of powder samples were performed using a Quantum Design VSM MPMS3 magnetometer. The sample was drop-casted from solution into standard propylene sample capsule. Magnetic simulations were performed using PHI code.[5] In AC magnetometry studies, the in-phase and out-of-phase susceptibility, $\chi'(\nu)$ and $\chi''(\nu)$, were measured as a function of oscillation frequency ν and then fitted with the generalized Debye model:

$$\chi'(\nu) = \chi_S + (\chi_T - \chi_S)\frac{1 + (\nu\tau_{\mathrm{M}})^{1-\alpha}\sin\left(\frac{\pi\alpha}{2}\right)}{1 + 2(\nu\tau_{\mathrm{M}})^{1-\alpha}\sin\left(\frac{\pi\alpha}{2}\right) + (\nu\tau_{\mathrm{M}})^{2-2\alpha}}$$

$$\chi''(\nu) = (\chi_T - \chi_S)\frac{(\nu\tau_{\mathrm{M}})^{1-\alpha}\cos\left(\frac{\pi\alpha}{2}\right)}{1 + 2(\nu\tau_{\mathrm{M}})^{1-\alpha}\sin\left(\frac{\pi\alpha}{2}\right) + (\nu\tau_{\mathrm{M}})^{2-2\alpha}} + b\nu$$

where τ_{M} is the relaxation time. The linear term ($b\nu$) is included to correct for a linear background that was found in $\chi''(\nu)$ measurements at high frequency (the background has noticeable contribution to χ'' curves only above > 100 Hz).

DFT computations. DFT calculations were performed at the PBE level[6] using the Orca package.[7, 8] For lanthanides with non-zero orbital momentum, all-electron DFT calculations can give ambiguous results because of the non-single-determinant wavefunction. To avoid potential problems, we used 4f-in-core effective core potentials of ECP*XX*MWB of Dolg et al. with corresponding ECP*XX*MWB-II basis sets.[9, 10] For C and F atoms, def2-TZVPP basis was used.[11] Molecular structures and isosurfaces were visualized with VMD.[12]

DFT calculations of $M_2@C_{80}$ in different electronic states

Table S1a. Relative energy of pT state (in eV) versus pS state in $M_2@I_h$-C_{80} and $M_2@D_{5h}$-C_{80} series.

M	$M_2@I_h$-C_{80}		$M_2@D_{5h}$-C_{80}	
	PBE	PBE0//PBE	PBE	PBE0//PBE
La	0.331	0.388	0.029	0.132
Ce	0.229	0.262	−0.068	−0.129
Pr	0.141	0.154	−0.140	−0.245
Nd	0.064	0.059	−0.206	−0.318
Gd	−0.177	−0.271	−0.318	−0.523
Tb	−0.214	−0.326	−0.324	−0.533
Dy	−0.242	−0.372	−0.321	−0.531
Ho	−0.260	−0.404	−0.308	−0.514
Er	−0.269	−0.422	−0.286	−0.484
Tm	−0.270	−0.436	−0.248	−0.408
Lu	−0.240	−0.715	−0.119	−0.225

The structure in pS and pT state were optimized at the PBE level with def2-TZVPP basis for carbon and Dolg's 4f-in-core MWB-II basis sets with core effective potentials for lanthanides, the relative energies obtained are denoted as "PBE"; singe-point calculations were then performed with the same basis using PBE0 functional, the energies are denoted as "PBE0//PBE".

$Nd_2@I_h$-C_{80} and $Ce_2@D_{5h}$-C_{80} were chosen to benchmark density functionals. Based on experimental evidence, pT state should be lower in energy than pS for the former, whereas pS state should be more stable for the latter. Table S1b compares relative energies computed with various functionals, including hybrid (PBE0, B3LYP, O3LYP, X3LYP, B3PW), meta-hybrid (TPSSh, TPSS0, M06, and M06-2X), and two range-separated hybrid functionals (CAM-B3LYP and ω-B97X). As can be concluded from Table S1b, none of the functionals gives correct order of pS and pT energies for two molecules at once, while M06 and M06-2X also converged to pT with different spin distribution, which resulted in the strong deviation of the energies. Other functional give close predictions, and we use PBE0 data in Fig. 2 in the main text.

Table S1b. Relative energy of pT state (in eV) versus pS in $Nd_2@I_h$-C_{80} and $Ce_2@D_{5h}$-C_{80} computed with different density functionals.

functional	$Nd_2@I_h$-C_{80}	$Ce_2@D_{5h}$-C_{80}
PBE	0.064	−0.068
PBE0	0.059	−0.129
B3LYP	−0.017	−0.199
O3LYP	0.213	0.047
X3LYP	−0.018	–0.203
B3PW	0.035	−0.143
TPSSh	0.000	−0.156
TPSS0	0.016	−0.168
M06	−0.404	−0.615
M062X	−0.004	−0.704
CAM-B3LYP	−0.030	−0.282
ω-B97X	0.077	−0.209

All calculations are single-point energies for PBE-optimized structures

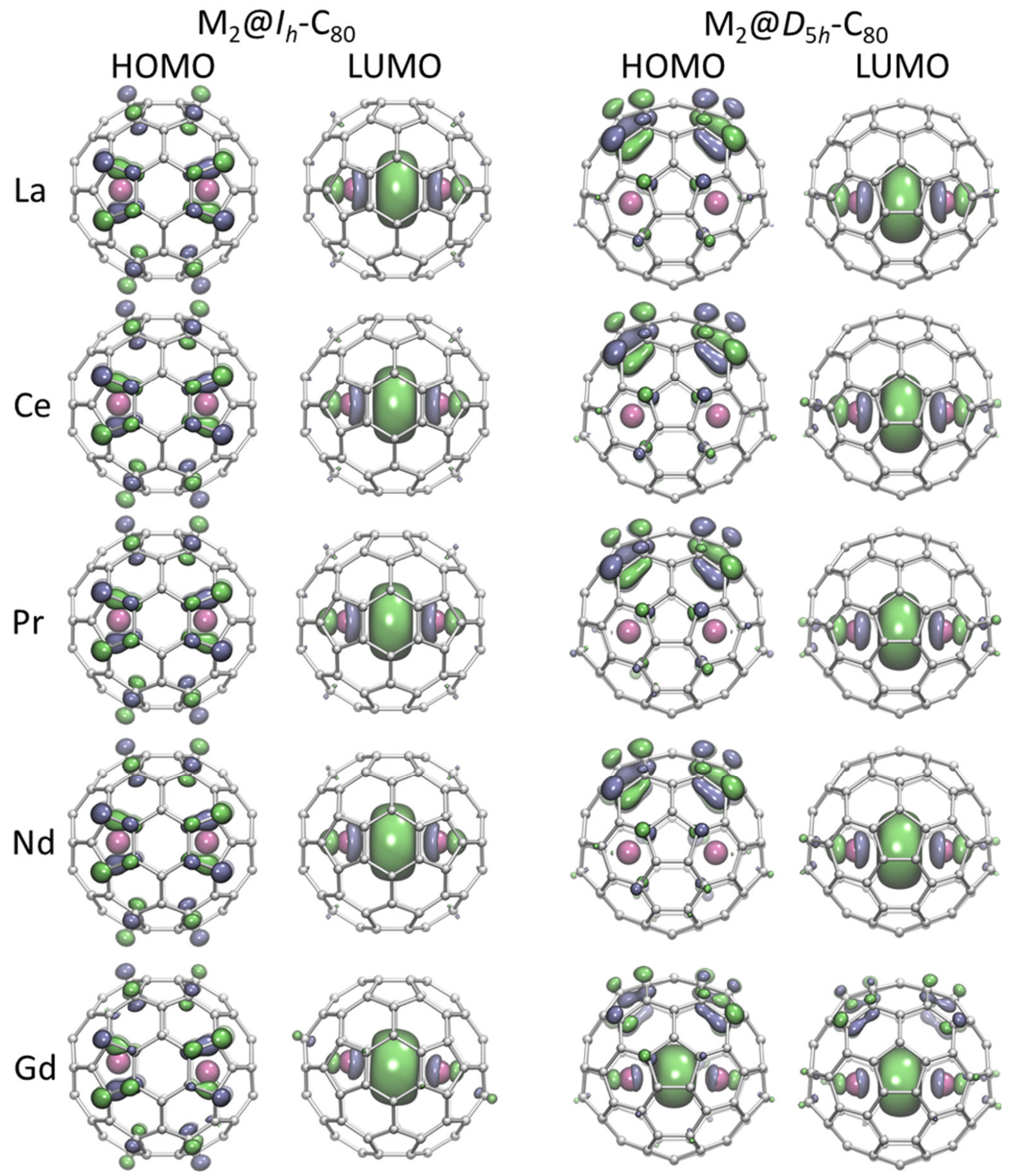

Figure S1 *(continued on the next page)*. HOMO and LUMO of selected pS-$M_2@I_h$-C_{80} and pS-$M_2@D_{5h}$-C_{80} at the PBE0//PBE level.

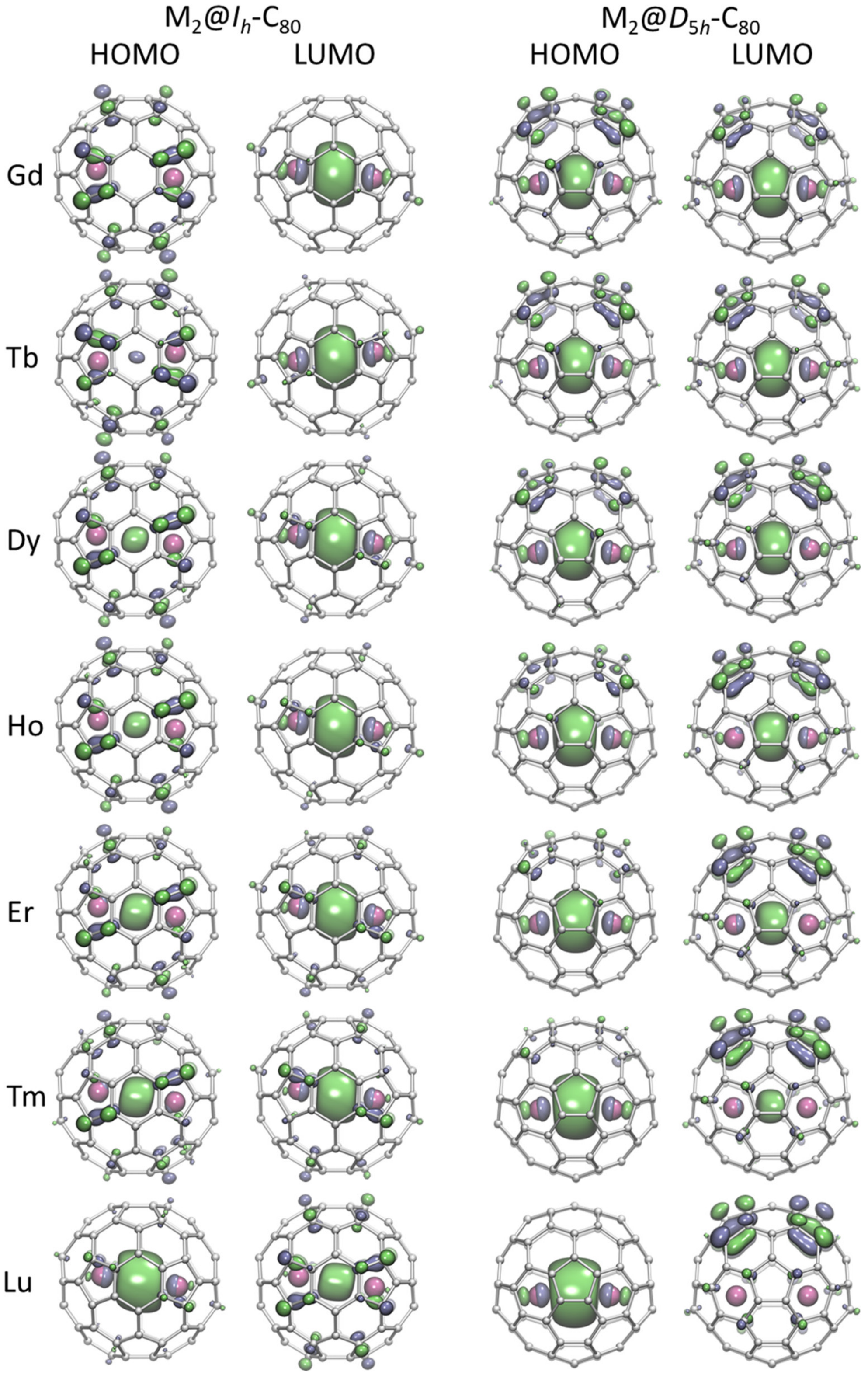

Figure S1 *(continued)*. HOMO and LUMO of selected pS-M_2@I_h-C_{80} and pS-M_2@D_{5h}-C_{80} at the PBE0//PBE level.

Spin density distribution in pT $Nd_2@C_{80}$, $[Nd_2@C_{80}]^-$, and $Nd_2@C_{80}(CF_3)$

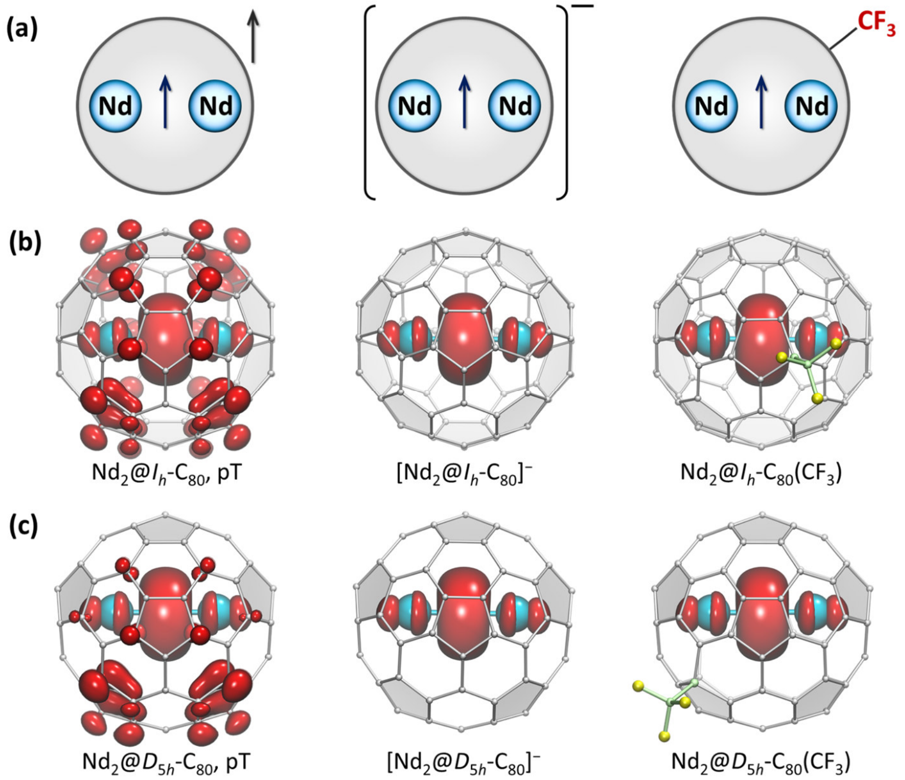

Figure S2. (a) Schematic description of neutral $Nd_2@C_{80}$ in pseudo-triplet (pT) state, $[Nd_2@C_{80}]^-$ anion with unpaired electron on the Nd–Nd bonding orbital, and $Nd_2@C_{80}(CF_3)$ with unpaired electron on the Nd–Nd bonding orbital. (b) DFT-computed valence spin density distribution in $Nd_2@C_{80}$ (pT state), $[Nd_2@C_{80}]^-$ anion and $Nd_2@C_{80}(CF_3)$ with I_h-C_{80} cage. (c) DFT-computed valence spin density distribution in $Nd_2@C_{80}$ (pT state), $[Nd_2@C_{80}]^-$ anion and $Nd_2@C_{80}(CF_3)$ with D_{5h}-C_{80} cage.

In the pT state of $Nd_2@C_{80}$, one SOMO is localized on the Nd_2 dimer, and one on the fullerene cage. Accordingly, the spin density has an equal contribution on Nd_2 dimer and on the fullerene cage. Upon single-electron reduction to $[Nd_2@C_{80}]^-$, the fullerene SOMO is populated by the second electron and turns into doubly-occupied MO, hence the fullerene cage attains a closed-shell electronic structure, leaving Nd_2 dimer with a single-electron Nd–Nd bond. The Nd–Nd bond is preserved in the neutral $Nd_2@C_{80}(CF_3)$ adduct.

Mass-spectra of EMF extracts

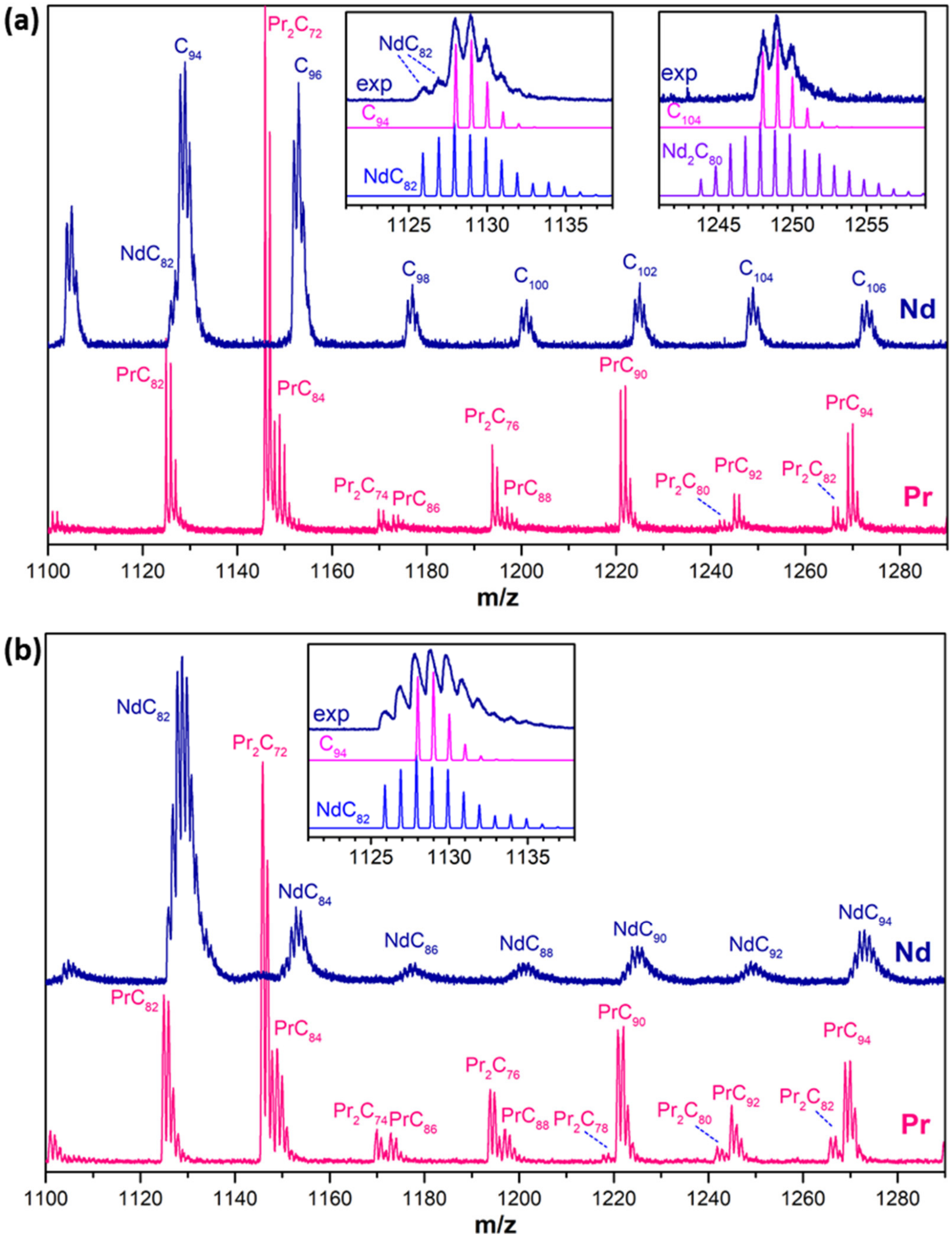

Figure S3. MALDI-TOF mass-spectra of Nd-EMF and Pr-EMF extracts in CS_2 in the range of $M@C_{82}$ and $M_2@C_{80}$ species: (a) negative ion mode, (b) positive ion mode. Insets show isotopic distribution and simulated mass-spectra. Note that mass-spectra of $Nd@C_n$ almost overlaps with C_{n+12} (e.g., $Nd@C_{82}$ with C_{94}), but can be distinguished b the presence of two peaks with lower mass for $Nd@C_{2n}$. Likewise, $Nd_2@C_n$ also overlaps with C_{n+24}, but $Nd_2@C_n$ has four peaks at lower mass which can be used to identify its presence. In mass-spectra of the CS_2 extract, we can detect the presence of monometallofullerenes $Nd@C_n$ but do not observe dimetallofullerenes $Nd_2@C_n$. For Pr, both $Pr@C_n$ and $Pr_2@C_n$ species can be detected.

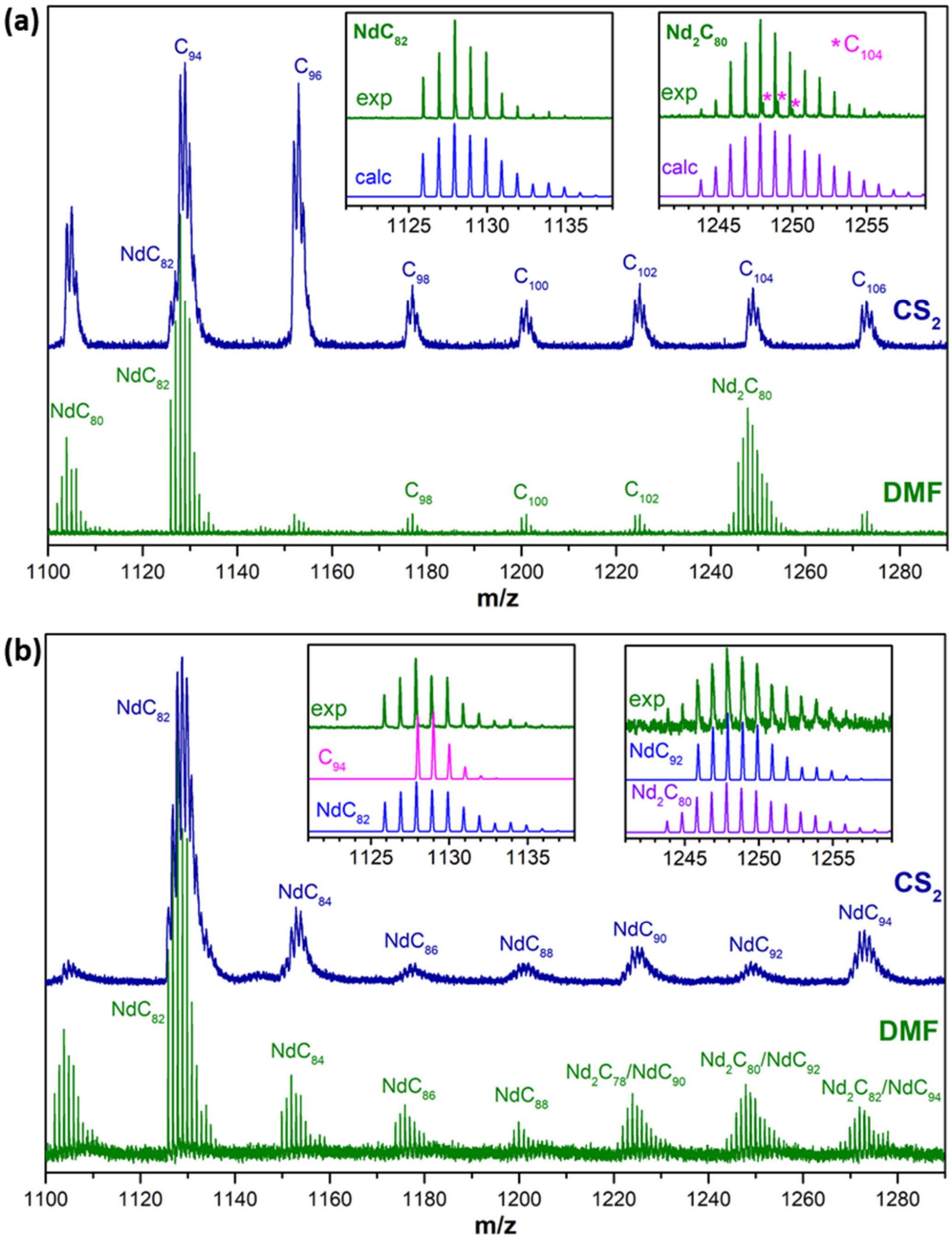

Figure S4. MALDI-TOF mass-spectra of Nd-EMF extracts in CS_2 and DMF in the range of Nd@C_{82} and Nd_2@C_{80} species: (a) negative ion mode, (b) positive ion mode. Insets show isotopic distribution and simulated mass-spectra. Whereas Nd_2@C_{2n} species are absent in mass-spectra of CS_2 extract, they can be readily detected in DMF extract. Note that mass-spectra of Nd_2@C_n species overlap with those of Nd@C_{n+12}, but di-EMFs have two additional peaks at lower masses, which allows to identify their presence in mixtures with mono-EMFs.

Synthesis and isolation of $Nd_2@C_{80}(CF_3)$ isomers

After arc-discharge evaporation, Nd-EMFs were extracted from the fullerene-containing soot by DMF under reflux. Umemoto reagent II was dissolved in DMF and added to the fullerene solution in DMF at room temperature. Optimal amount of URII for the synthesis of $Nd_2@C_{80}(CF_3)$ was determined by reacting aliquots of fullerene solution with different amount of URII (Fig. S5). After 5 minutes, DMF was removed under reduced pressure, the residue dissolved in toluene and processed by HPLC (Fig. S6). Note that reaction mixture also contained unidentified products of DMF decomposition, which elute at short retention time.

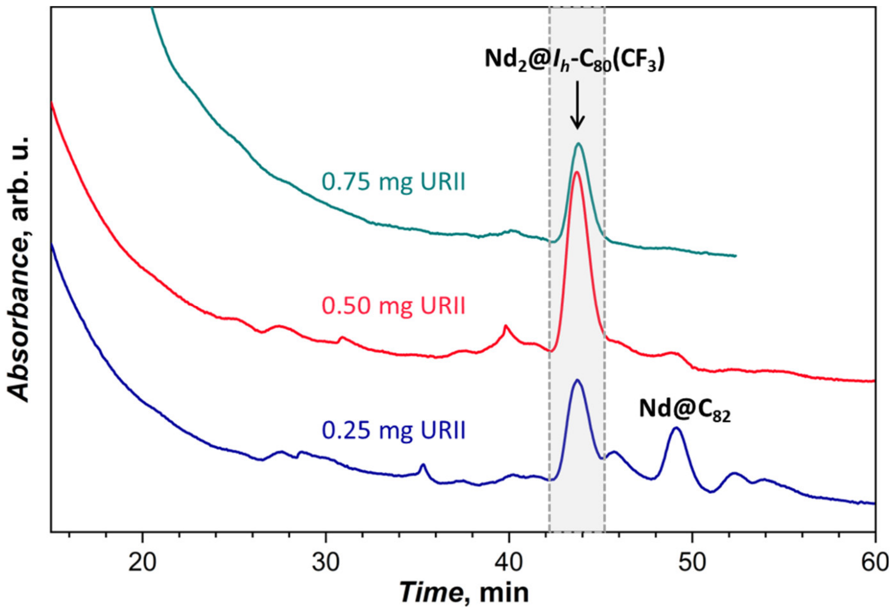

Figure S5. Optimization of the URII amount for the synthesis of $Nd_2@I_h$-$C_{80}(CF_3)$. To identical aliquots of Nd-EMF extract dissolved in DMF (5 mL each), weighed amount of URII was added, after which DMF was removed by evaporation under reduced pressure, the residue dissolved in toluene, and toluene-soluble part analyzed with HPLC. The main component of the fraction eluting at 42–45 min is $Nd_2@I_h$-$C_{80}(CF_3)$. Comparison of HPLC traces shows that addition of 0.5 mg of URII gives the best yield of the target compound. When 0.25 mg is added, the residual $Nd@C_{82}$ can be well seen in the chromatogram, while amount of the target fraction is comparably small. Addition of 0.75 mg also gives smaller amount of the product because it reacts further and presumably forms multiaddducts. Therefore, the main trifluoromethylation reaction was performed using the reagent ratio corresponding to 0.5 mg URII per 5 mL of the Nd-EMF extract.

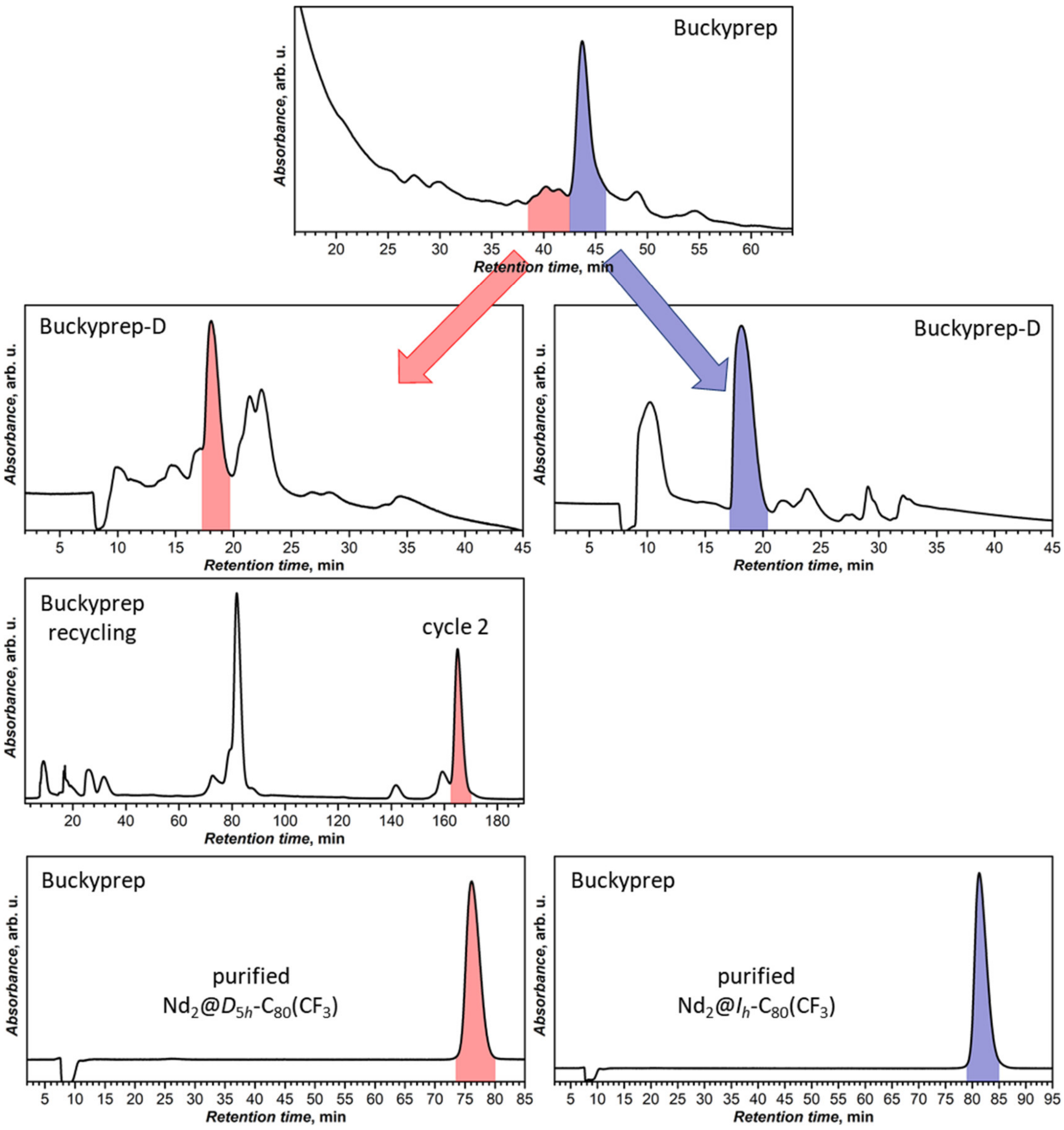

Figure S6. HPLC separation of $Nd_2@C_{80}(CF_3)$ isomers from the crude mixture of reaction products. Eluent – toluene. Separation of $Nd_2@I_h$-$C_{80}(CF_3)$ was achieved in 2 steps with Buckyprep and Byckyprep-D columns. For $Nd_2@D_{5h}$-$C_{80}(CF_3)$, an additional step of recycling HPLC on Buckyprep column was required.

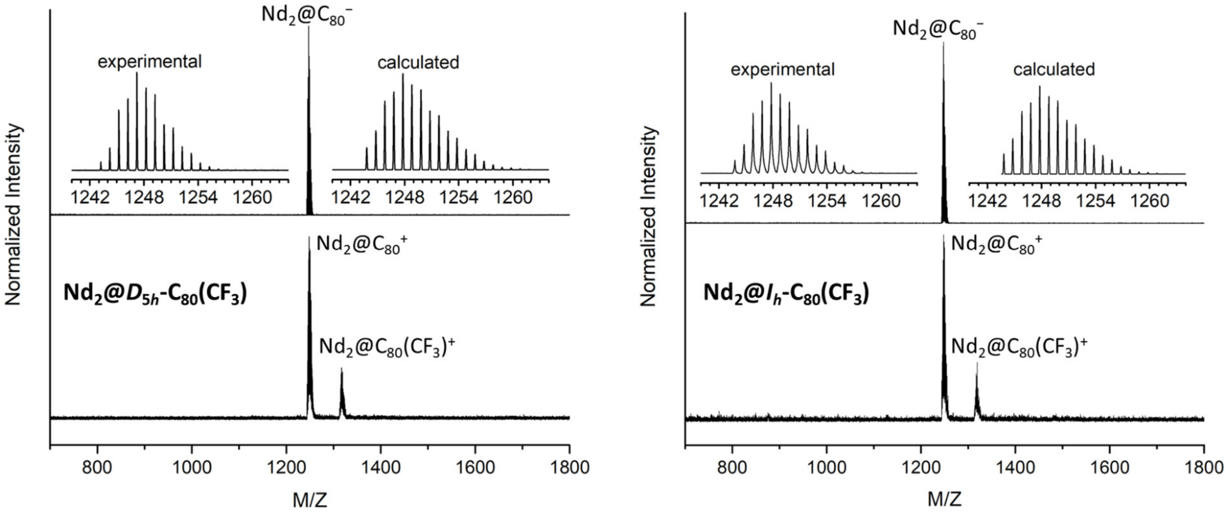

Figure S7. MALDI-TOF mass-spectra of purified $Nd_2@D_{5h}$-$C_{80}(CF_3)$ (left) and $Nd_2@I_h$-$C_{80}(CF_3)$ (right). Upper panel – negative ion mode, lower panel – positive ion mode. $Nd_2@C_{80}(CF_3)$ easily fragment in MALDI conditions, giving large $Nd_2@C_{80}^+$ fragment in positive ion mode. In negative mode, molecular ion could not be detected at all, giving only $Nd_2@C_{80}^-$ fragment.

IR spectroscopy

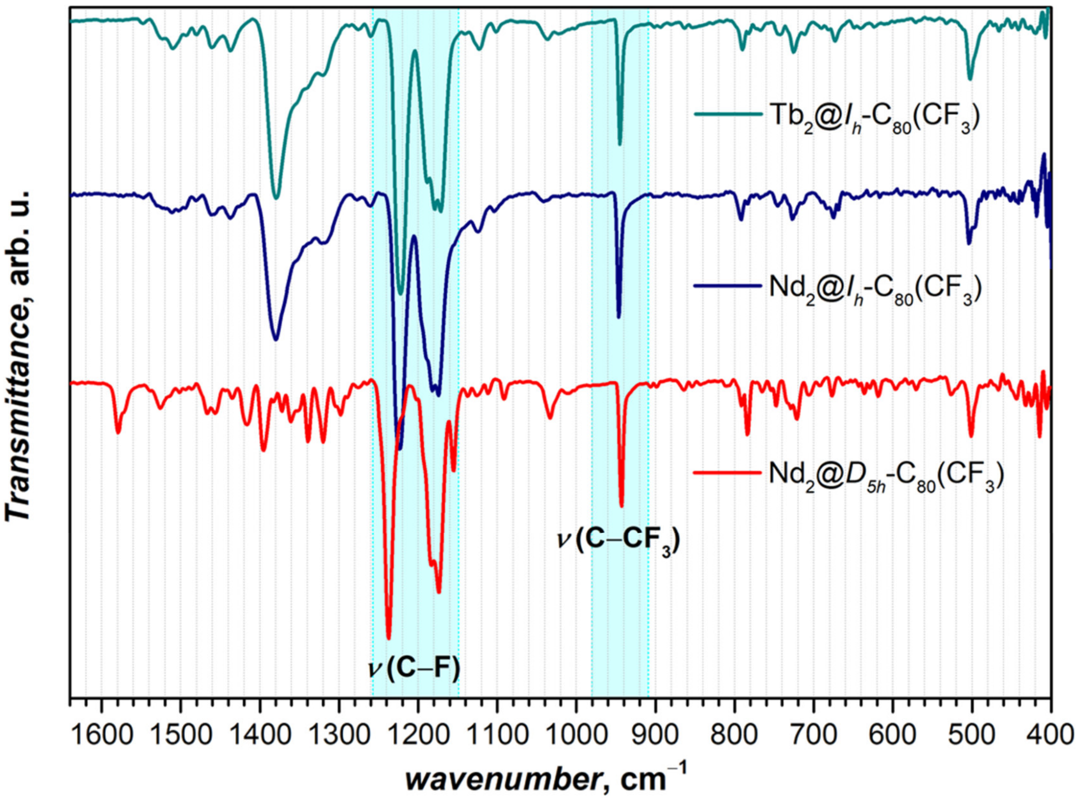

Figure S8. IR spectra of $Nd_2@D_{5h}$-$C_{80}(CF_3)$, $Nd_2@I_h$-$C_{80}(CF_3)$, and $Tb_2@I_h$-$C_{80}(CF_3)$ drop-casted on KBr substrate and measured in transmission mode with FTIR microscope. Characteristic vibrations of the CF_3 group are highlighted with pale cyan: in I_h isomers, strong absorptions at 1170–1195 cm^{-1} and 1222 cm^{-1} are assigned to antisymmetric (*E*-mode) and symmetric (A_1-mode) C–F stretching vibrations, respectively, denoted in the figure as *ν*(C–F). In D_{5h} isomer, the A_1-mode is shifted to 1237 cm^{-1}, whereas E-mode occurs at the same frequency as in the I_h isomer. The sharp line at 943–946 cm^{-1} in the spectra of all compounds is assigned to the stretching vibration of the C–C bond between fullerene and CF_3 group, *ν*(C–CF_3).

Raman spectra

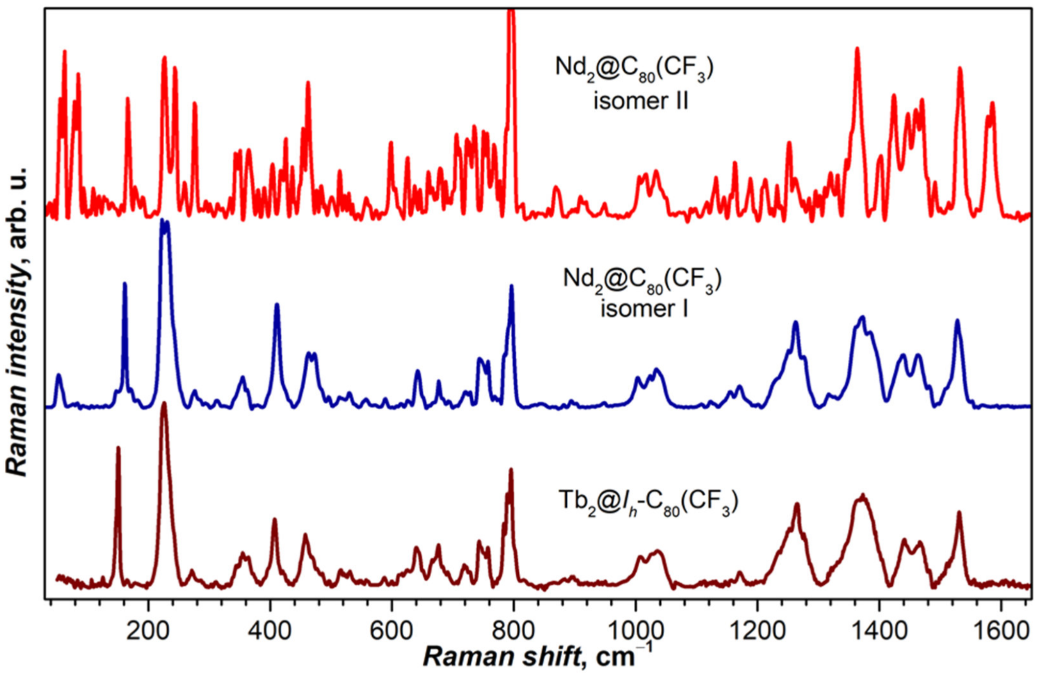

Figure S9. Raman spectra of $Nd_2@D_{5h}$-$C_{80}(CF_3)$ (isomer II), $Nd_2@I_h$-$C_{80}(CF_3)$ (isomer I), and $Tb_2@I_h$-$C_{80}(CF_3)$ drop-casted on KBr substrate. Spectra were excited at 532 nm and measured at 78 K.

Single-crystal X-ray diffraction

Table S2. Crystal data on isomers of $Nd_2@C_{80}(CF_3)$.

Crystal	$2Nd_2@I_h(7)-C_{80}(CF_3)\cdot 4NiOEP \cdot 1.63C_7H_8\cdot 0.37C_6H_6$	$Nd_2@D_{5h}(6)-C_{80}(CF_3)\cdot 2NiOEP\cdot C_6H_6$
**Formula**	$C_{319.63}H_{193.11}F_6N_{16}Nd_4Ni_4$	$C_{159}H_{94}F_3N_8Nd_2Ni_2$
**Formula weight**	5183.39	2579.32
**Color, habit**	Black, block	Black, block
**Crystal system**	monoclinic	monoclinic
**Space group**	*C*2/*c*	*C*2/*c*
***a*, Å**	43.010(9)	21.050(4)
***b*, Å**	41.170(8)	20.580(4)
***c*, Å**	23.490(5)	23.840(5)
***α*, deg**	90	90
***β*, deg**	97.04(3)	96.63(3)
***γ*, deg**	90	90
**Volume, Å^3**	41281(15)	10259(4)
Z	8	4
***T*, K**	100	100
**Radiation (λ, Å)**	Synchrotron Radiation (0.7999)	Synchrotron Radiation (0.77977)
**Unique data (R_{int})**	64226 (0.0800)	16888 (0.0682)
**Parameters**	4508	1614
**Restraints**	8477	1957
**Observed data ($I > 2\sigma(I)$)**	55817	15642
**$R_1{}^a$ (observed data)**	0.0883	0.0692
**$wR_2{}^b$ (all data)**	0.2357	0.1961
**CCDC NO.**	2283905	2283904

[a]For data with $I > 2\sigma(I)$, $R_1 = \frac{\sum||F_o|-|F_c||}{\sum|F_o|}$. [b]For all data, $wR_2 = \sqrt{\frac{\sum[w(F_o^2-F_c^2)^2]}{\sum[w(F_o^2)^2]}}$.

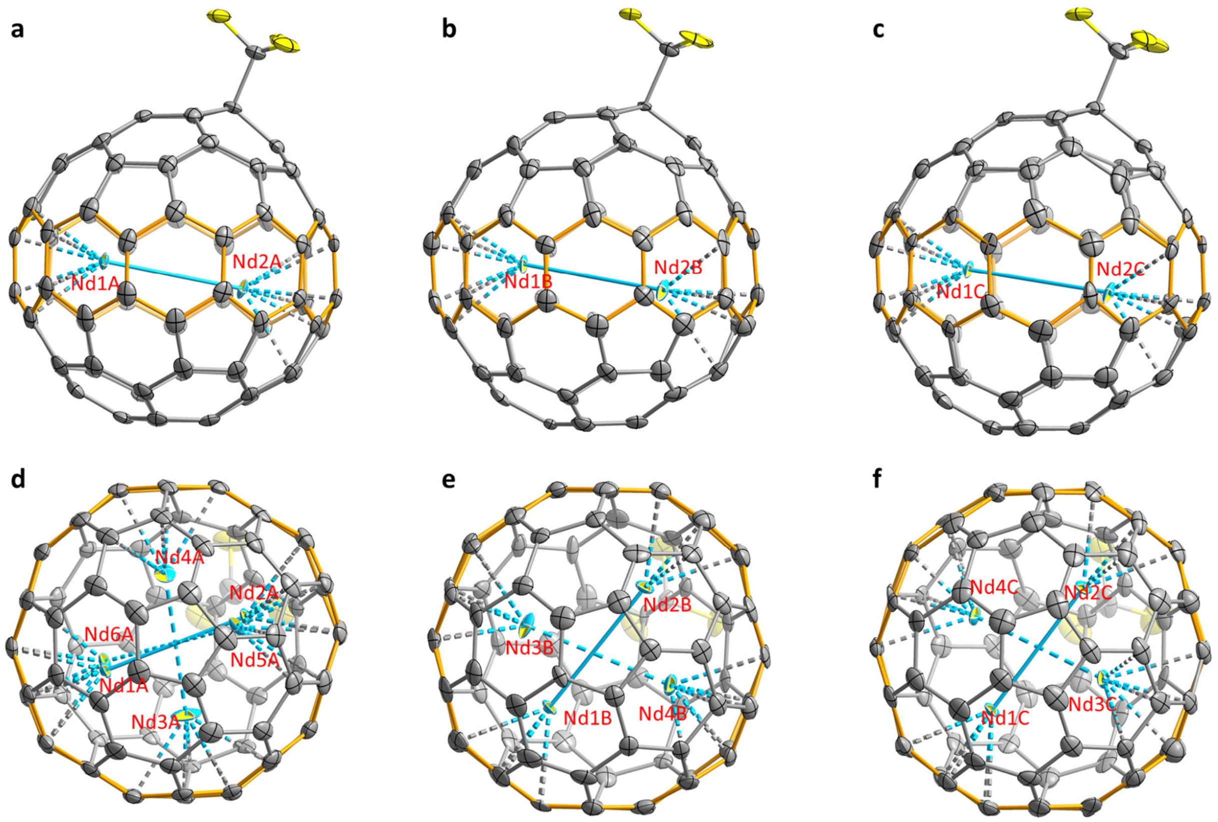

Figure S10. A, B, and C fullerene sites in 2Nd_2@I_h-$C_{80}(CF_3)$·4NiOEP·1.63C_7H_8·0.37C_6H_6. The displacement parameters are shown at the 30% probability level. Upper row shows only major Nd_2 sites, bottom row shows all Nd sites and a different orientation of the fullerene cage. Color code: grey for carbon, cyan for Nd, and yellow for F. The belt of hexagons around which metal dimer prefers to locate is highlighted yellow. The Nd disordered sites are labeled with site occupancies of 0.72, 0.20, and 0.07 for Nd1A/Nd2A, Nd3A/Nd4A, and Nd5A/Nd6A; 0.36, and 0.14 for Nd1B/Nd2B, and Nd3B/Nd4B; 0.36 and 0.14 for Nd1C/Nd2C, and Nd3C/Nd4C, respectively.

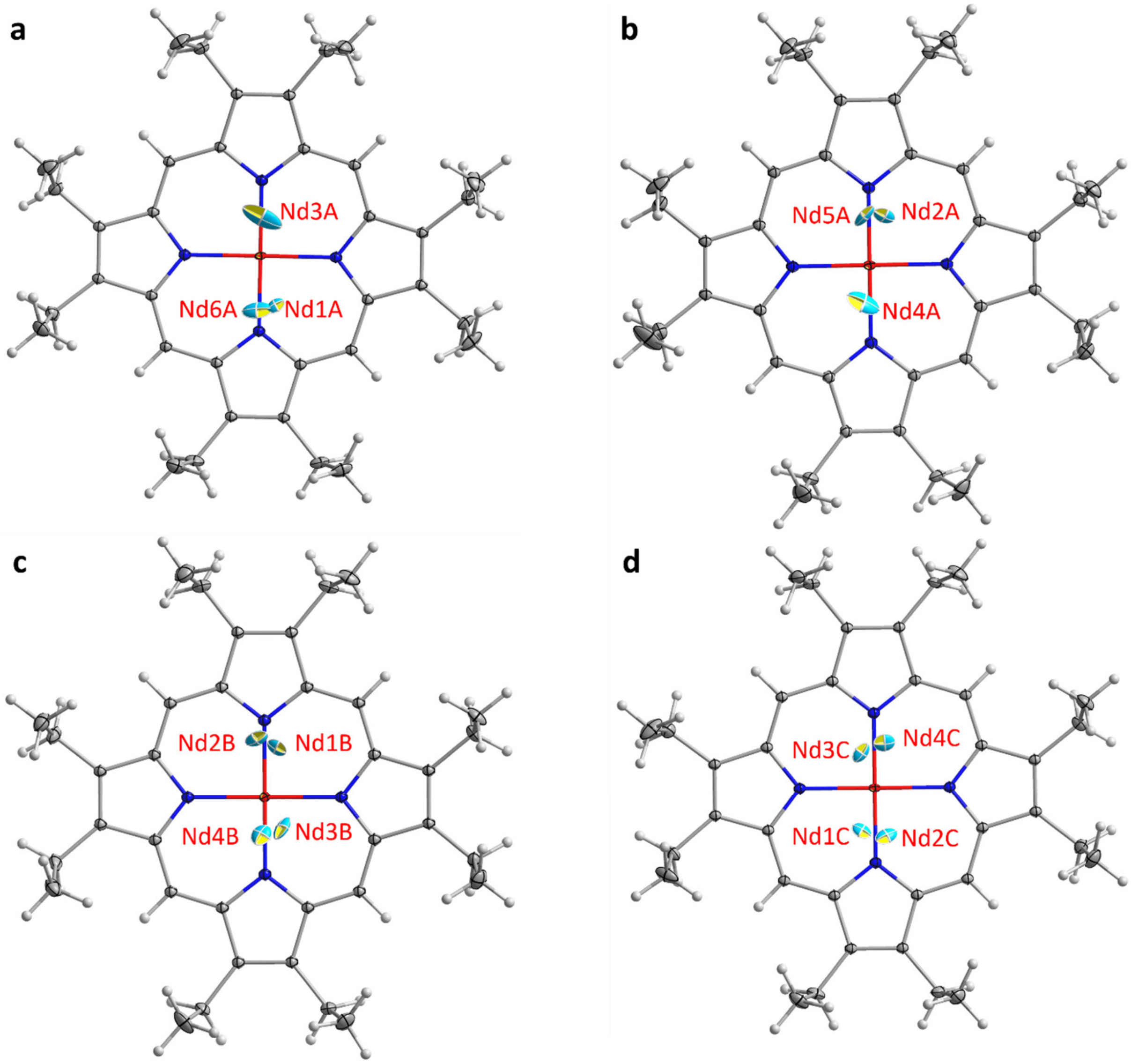

Figure S11. Relative position of endohedral Nd sites and NiOEP moieties in $2Nd_2@I_h$-$C_{80}(CF_3)\cdot 4NiOEP\cdot 1.63C_7H_8\cdot 0.37C_6H_6$. $C_{80}(CF_3)$ moieties and solvent molecules are omitted for clarity. The displacement parameters are shown at the 30% probability. Color code: grey for carbon, blue for nitrogen, white for hydrogen, red for nickel, and cyan for Nd.

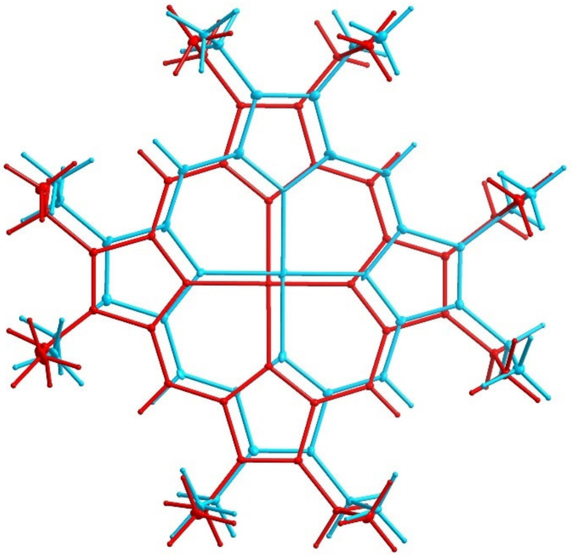

Figure S12. The disordered NiOEP molecule in $Nd_2@D_{5h}$-$C_{80}(CF_3)$·2NiOEP·C_6H_6, shown with red for the main site (site occupancy of 0.85) and cyan for the minor site (site occupancy of 0.15).

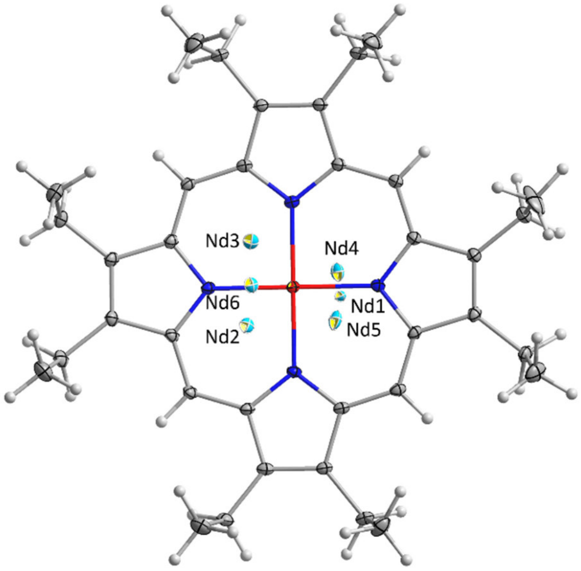

Figure S13. Relative position of endohedral Nd sites and NiOEP moieties in $Nd_2@D_{5h}$-$C_{80}(CF_3)$·2NiOEP·C_6H_6. $C_{80}(CF_3)$ moieties and solvent molecules are omitted for clarity. The displacement parameters are shown at the 30% probability. Colour code: grey for carbon, blue for nitrogen, white for hydrogen, red for nickel, and cyan for Nd.

Table S3. Metal-metal distances in lanthanide di-EMFs with I_h-C_{80} and D_{5h}-C_{80} fullerene cages.

di-EMF	C_{80} isomer	M–M distance	M–M bonding	M site occupancies [a]	Ref.
$La_2@C_{80}$	I_h(7)	3.84(2)	no	powder XRD	13
$La_2@C_{80}((CH_2)_2N\text{-}CPh_3)$	I_h(7)	3.823(1)	no	0.68/0.68	14, 15
$La_2@C_{80}((Si(Ph\text{-}(C_2H_5)_2)$ $CH_2CH(PhCH_3))$	I_h(7)	3.793(2)	no	0.86/0.86	16
$La_2@C_{80}((Si(Ph\text{-}(C_2H_5)_2)_2)_2CH_2)$	I_h(7)	3.796(3)	no	0.64/0.64	17
$La_2@C_{80}(C_{10}H_{14})$	I_h(7)	4.033(1)	no	0.98/0.98	18
$La_2@C_{80}(CClPh)$	I_h(7)	4.013(1)	no	1/1	19
$La_2@C_{80}(CClPh)(C_{10}H_{14})$	I_h(7)	4.159(3)	no	1/0.62	19
$La_2@C_{80}(C(CN)_2OC(CN)_2)$	I_h(7)	3.816(2)	no	0.38/0.36 (out of 0.5)	20
$La_2@C_{80}(CH_2Ph)$	I_h(7)	3.71(4) Å	yes	averaged over strongly disordered positions	21
$La_2@C_{80}(C_3N_3\text{-}Ph_2)$	I_h(7)	3.784(2)	yes	0.69/0.67	22
$[La_2@C_{80}(C_3N_3\text{-}Ph_2)(CH_2Ph)]_2$	I_h(7)	3.786(2)	yes	0.89/0.89	22
$La_2@C_{80}(C_3N_3\text{-}Ph_2)$	D_{5h}(6)	3.733(3)	yes	0.54/0.54	22
$Ce_2@C_{80}$	I_h(7)	3.767(6)	no	0.24/0.30	23
$Ce_2@C_{80}((CH_2)_2N\text{-}CPh_3)$	I_h(7)	3.900(2)	no	0.45/0.50	14
$Ce_2@C_{80}((Si(Ph\text{-}(CH_3)_3)_2)_2CH_2)$	I_h(7)	3.828(1)	no	0.87/0.87	24
$Ce_2@C_{80}(C(Ph)(C_3H_6COOCH_3))$	I_h(7)	4.059(1)	no	1/1	25
$Ce_2@C_{80}$	D_{5h}(6)	3.764(2)	no	0.36/0.28 (out of 0.5)	23
$Nd_2@C_{80}(CF_3)$, site A	I_h(7)	3.792(2)	yes	0.72/0.72	t.w.
$Nd_2@C_{80}(CF_3)$, site B	I_h(7)	3.791(2)	yes	0.36/0.36 (out of 0.5)	t.w.
$Nd_2@C_{80}(CF_3)$, site C	I_h(7)	3.778(2)	yes	0.36/0.36 (out of 0.5)	t.w.
$Nd_2@C_{80}(CF_3)$	D_{5h}(6)	3.788(2)	yes	0.26/0.21 (out of 0.5)	t.w.
$Gd_2@C_{79}N$	I_h(7)	3.835(9)	yes	0.21/0.21 (out of 0.5)	26
$Tb_2@C_{79}N$	I_h(7)	3.902(1)	yes	0.43/0.43	27
$Tb_2@C_{80}(CF_3)$, site A	I_h(7)	3.856 (2)	yes	0.58/0.58	27
$Tb_2@C_{80}(CF_3)$, site B	I_h(7)	3.850(2)	yes	0.38/0.38 (out of 0.5)	27
$Tb_2@C_{80}(CF_3)$, site C	I_h(7)	3.837(2)	yes	0.37/0.37 (out of 0.5)	27
$Dy_2@C_{80}(CH_2Ph)$	I_h(7)	3.896(1)	yes	0.70/0.66	28
$Dy_2@C_{79}N$	I_h(7)	3.890(2)	yes	0.31/0.23	29

[a] unless otherwise noted, site occupancies are out of 1

Conformers of $Nd_2@I_h$-$C_{80}(CF_3)$

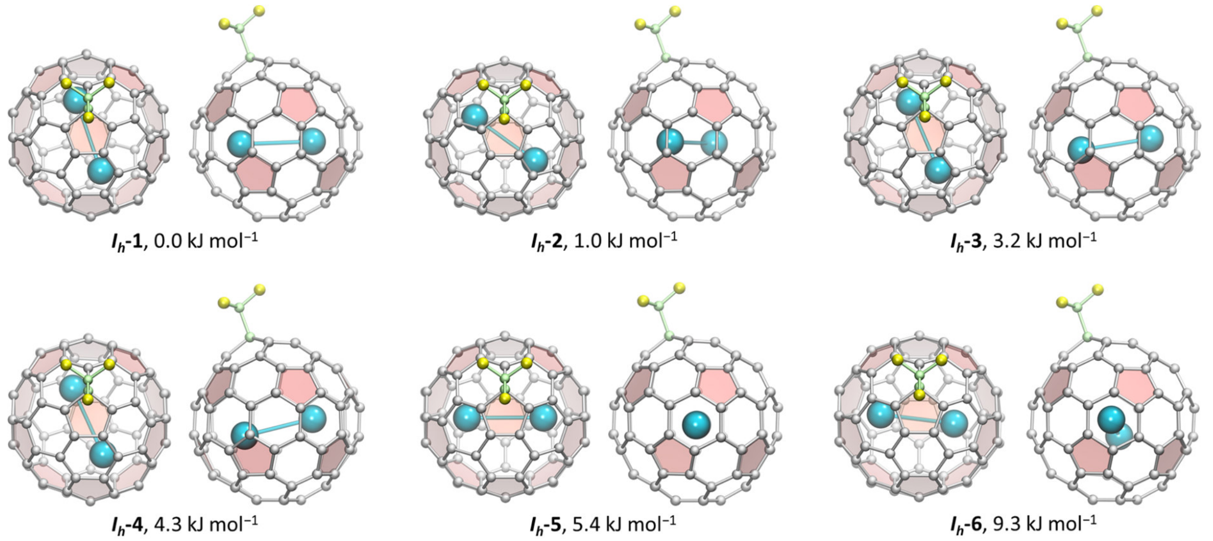

Figure S14. Conformers of $Nd_2@I_h$-$C_{80}(CF_3)$ obtained by varying a position of the Nd_2 dimer inside $C_{80}(CF_3)$ moiety. Two orientations of the fullerene cage are shown for each conformer along with its relative energy.

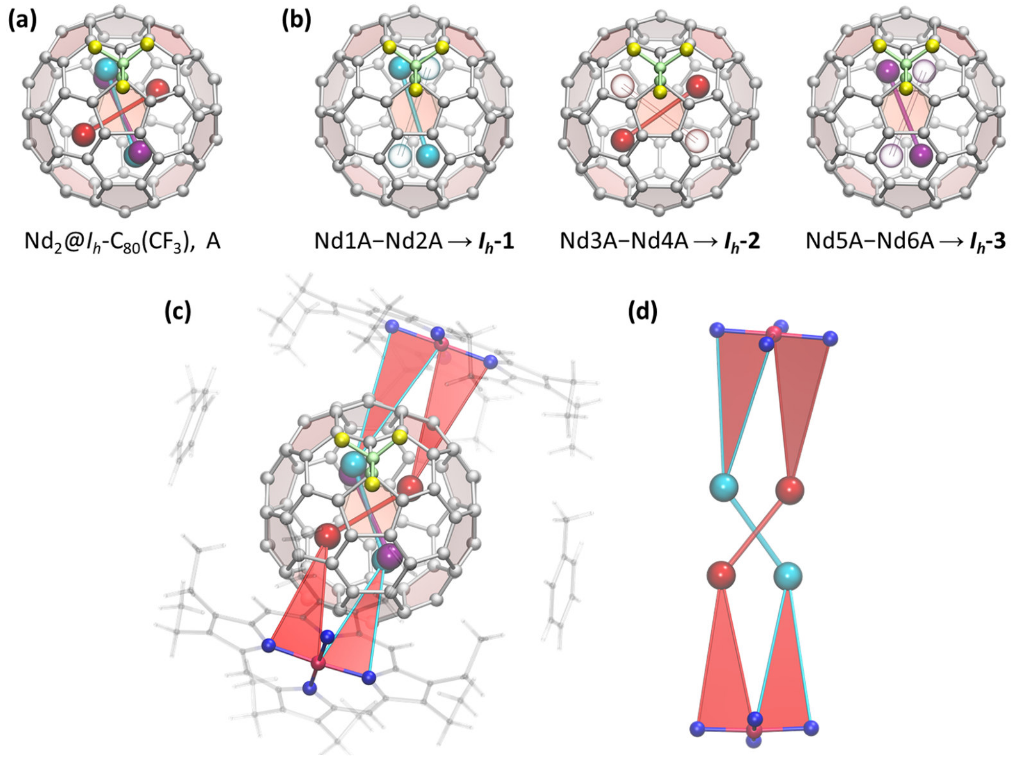

Figure S15. (a) $Nd_2@I_h$-$C_{80}(CF_3)$, site A from the SC-XRD structure; three Nd_2 sites are colored by cyan (Nd1A, Nd2A, 0.72), red (Nd3A, Nd4A, 0.20) and purple (Nd5A, Nd6A, 0.07). (b) DFT-optimized molecule A with each of the Nd_2 sites; also shown are symmetry replica of Nd atoms obtained by applying a mirror plane operation of the C_s-symmetric I_h-$C_{80}(CF_3)$ moiety. (c) the same as (a), but also showing two nearest NiOEP and toluene molecules. For each Nd atom, the nearest Ni–N bond in the NiOEP molecule is highlighted with semitransparent triangles. (d) the same as (c) but showing only Nd, Ni, and N atoms.

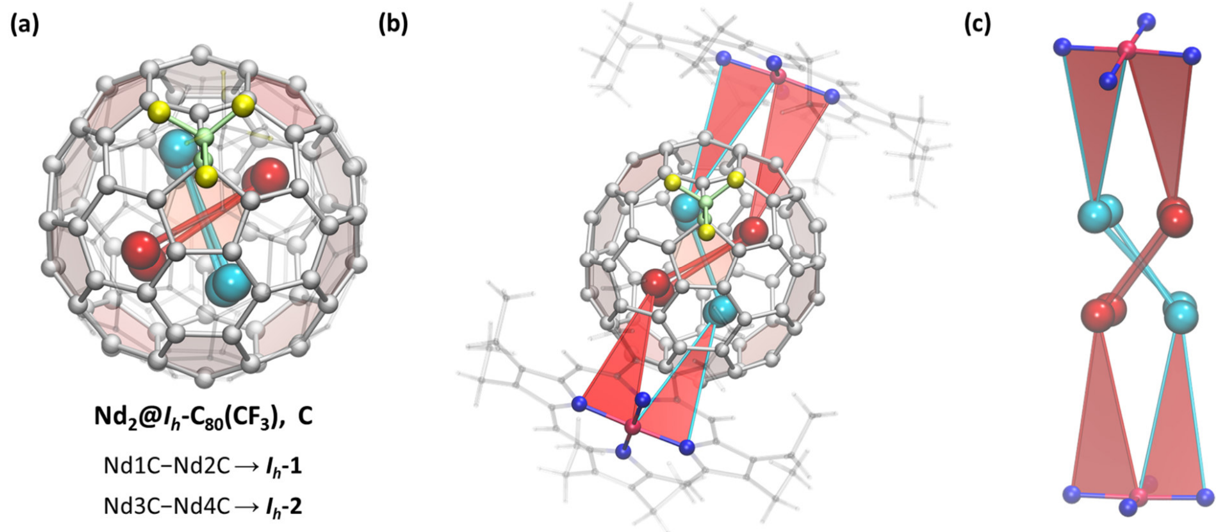

Figure S16. (a) Nd_2@I_h-C_{80}(CF_3), site C from the SC-XRD structure; one cage orientation is shown full-color, another one is semitransparent; two Nd_2 sites and their symmetry replicas are colored by cyan (Nd1C, Nd2C, 0.36) and red (Nd3A, Nd4A, 0.14). The site Nd1C–Nd2C corresponds to the conformer ***I***$\boldsymbol{I_h}$**-1** (see Fig. S14), while the site Nd3C–Nd4C corresponds to the conformer $\boldsymbol{I_h}$**-2**. (b) the same as (a), but also showing two nearest NiOEP; the second cage orientation is omitted. For each unique Nd atom, the nearest Ni–N bond in the NiOEP molecule is highlighted with semitransparent triangles. (c) the same as (b) but showing only Nd, Ni, and N atoms.

Table S4. DFT-computed[a] M–M and shortest M–C distances in M_2@I_h-C_{80}(CF_3) (M = Nd, Tb) and Nd_2@D_{5h}-C_{80}(CF_3).

	Nd_2@I_h-C_{80}(CF_3)		Tb_2@I_h-C_{80}(CF_3)		Nd_2@D_{5h}-C_{80}(CF_3)	
	Nd1–C_6	Nd2–C_6	Tb1–C_6	Tb2–C_6	Nd1–C_4	Nd2–C_4
M–C	2.478	2.452	2.354	2.339	2.430	2.435
	2.494	2.489	2.400	2.389	2.439	2.444
	2.505	2.492	2.404	2.412	2.528	2.539
	2.523	2.566	2.484	2.532	2.554	2.541
	2.551	2.576	2.512	2.540		
	2.552	2.608	2.542	2.599		
M–M	3.826		3.942		3.797	

[a] Analysis of experimental Nd–C distances in X-ray structure is complicated by the disorder, which makes the values not very reliable.

Conformers of $[Nd_2@D_{5h}\text{-}C_{80}]^-$

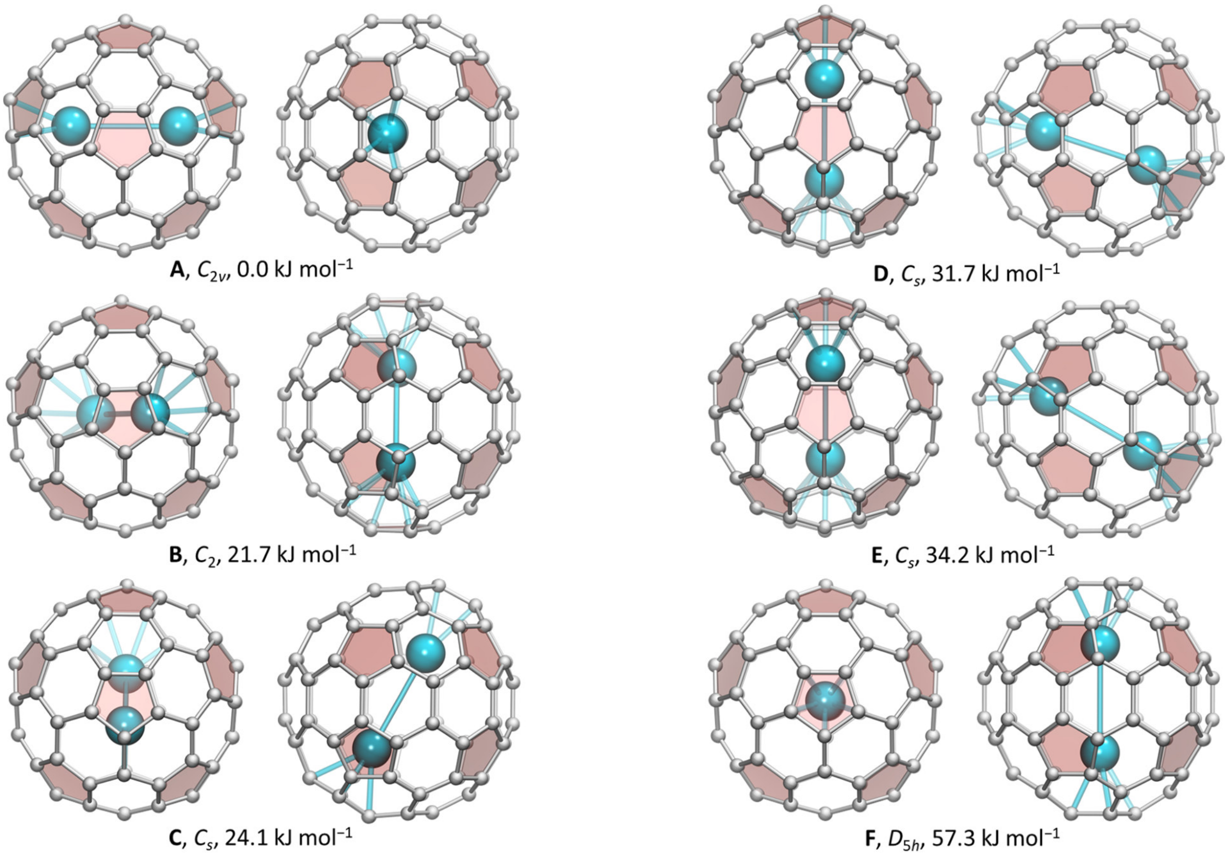

Figure S17. Six conformers of $[Nd_2@D_{5h}\text{-}C_{80}]^-$ found in DFT calculations, their point symmetry and relative energies. Note that no symmetry restrictions were used in the optimization.

Regioisomers of $Nd_2@D_{5h}$-$C_{80}(CF_3)$

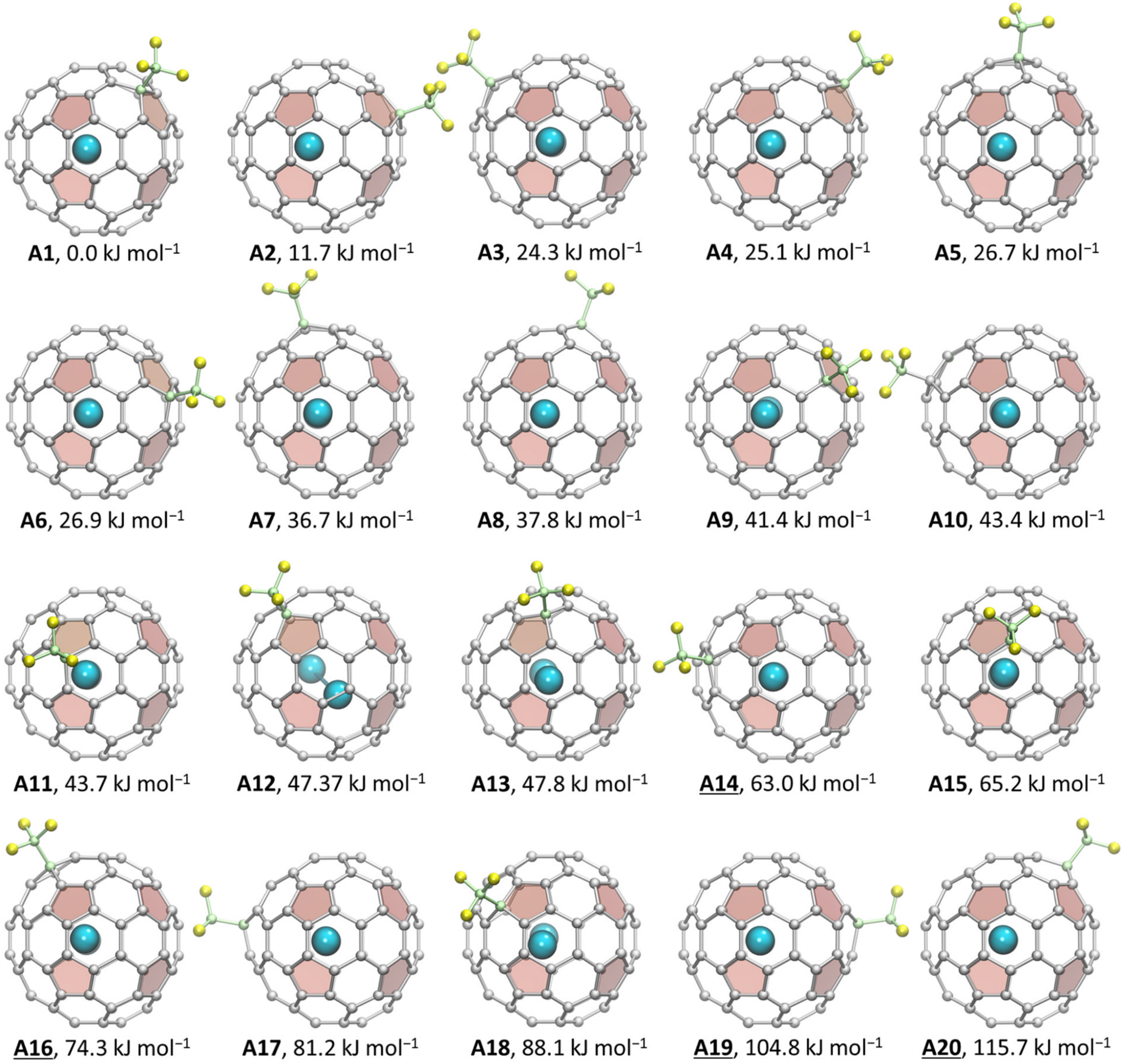

Figure S18. Isomers of $Nd_2@D_{5h}$-$C_{80}(CF_3)$ obtained by addition of CF_3 group to different carbons in the most stable conformer **A** of $Nd_2@D_{5h}$-C_{80}. For the isomers with CF_3 group added to the THJ carbon, the isomer number is underlined. Two more THJ isomers are not shown as we encountered sever SCF convergence problems for them and could not perform optimization. They are likely to be even less stable than other THJ isomer since convergence problems are caused by instabilities in their electronic structure.

Conformers of $Nd_2@D_{5h}$-$C_{80}(CF_3)$

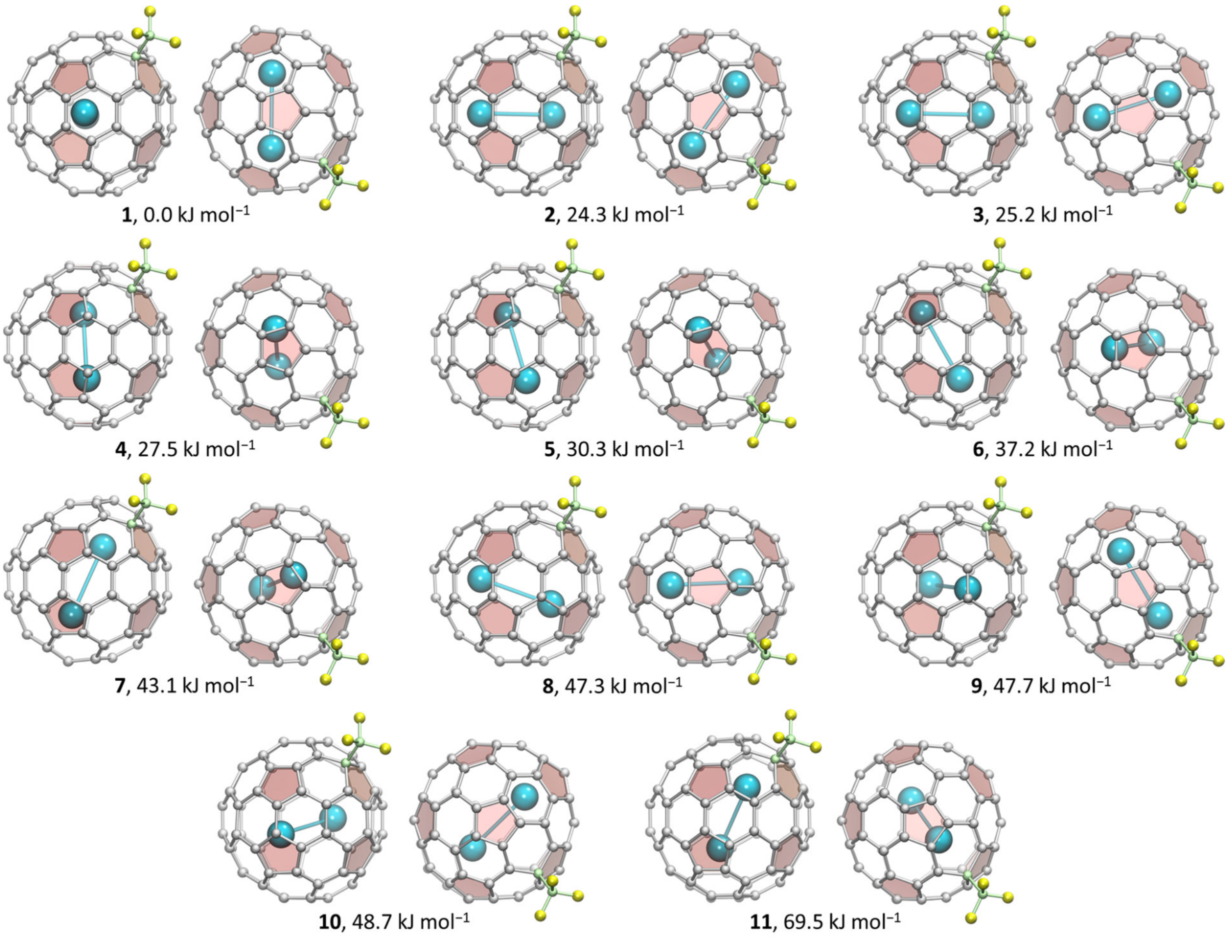

Figure S19. Conformers of $Nd_2@D_{5h}$-$C_{80}(CF_3)$ obtained by fixing the CF_3 position to that of the experimental structure and varying a position of the Nd_2 dimer. Two orientations of the fullerene cage are shown for each conformer along with the relative energy. Note that some of the structure are necessary repetitive with Fig. S18 (e.g., **1** and **A1**, **2** and **A3**, etc.)

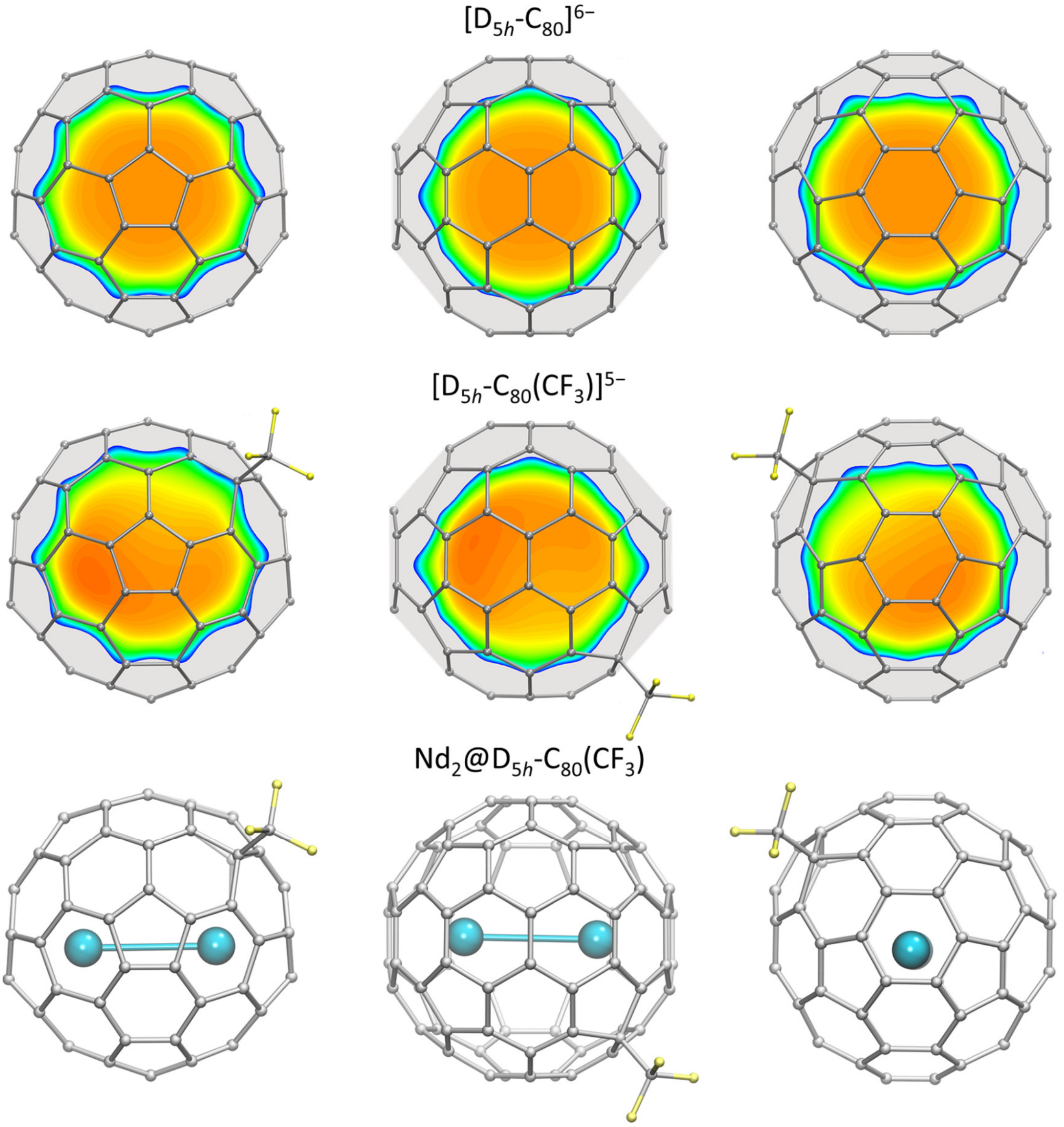

Figure S20. Electrostatic potential (ESP) maps inside the fullerene cage computed for $[D_{5h}\text{-}C_{80}]^{6-}$ (upper row) and $[D_{5h}\text{-}C_{80}(CF_3)]^{5-}$ (middle row) compared to the molecular structure of the lowest-energy conformer of $Nd_2@D_{5h}\text{-}C_{80}(CF_3)$ (bottom row). Each molecule is shown in three perpendicular orientations. While ESP in $[D_{5h}\text{-}C_{80}]^{6-}$ is distributed uniformly, addition of CF_3 group results in considerable inhomogeneity of ESP in $[D_{5h}\text{-}C_{80}(CF_3)]^{5-}$. The minimum of ESP (colored red) is close to the position of one of the Nd atoms in $Nd_2@D_{5h}\text{-}C_{80}(CF_3)$. Electrostatic stabilization of the metal position close to the ESP explains why this particular conformer is the most stable and hints to a possible hindered motion of metal atoms in $Nd_2@D_{5h}\text{-}C_{80}(CF_3)$ in comparison to $[Nd_2@D_{5h}\text{-}C_{80}]^{-}$. However, this factor cannot explain the difference of metal dynamics between I_h and D_{5h} cage isomers.

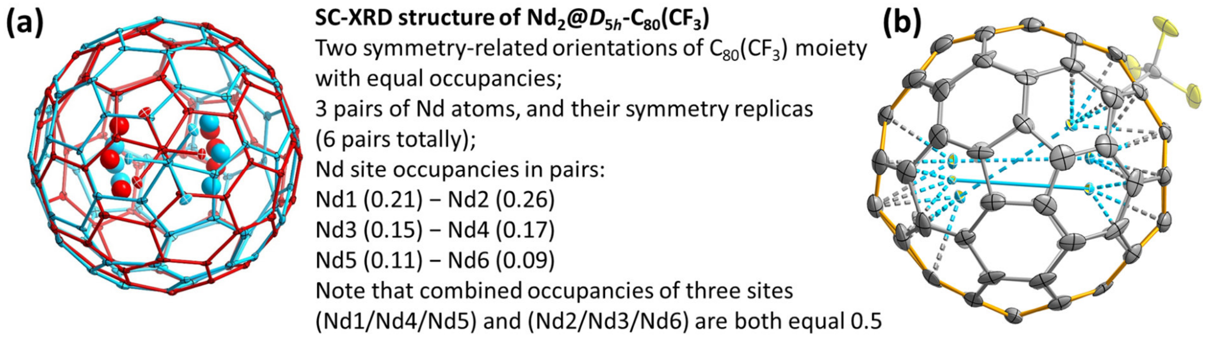

Figure S21. (a) SC-XRD structure of $Nd_2@D_{5h}$-$C_{80}(CF_3)$ with two overlapping D_{5h}-$C_{80}(CF_3)$ moieties and 6 pairs of Nd sites (3 unique pairs and tree symmetry replica). (b) One of the cage orientations with a probable distribution of three Nd_2 pairs in it.

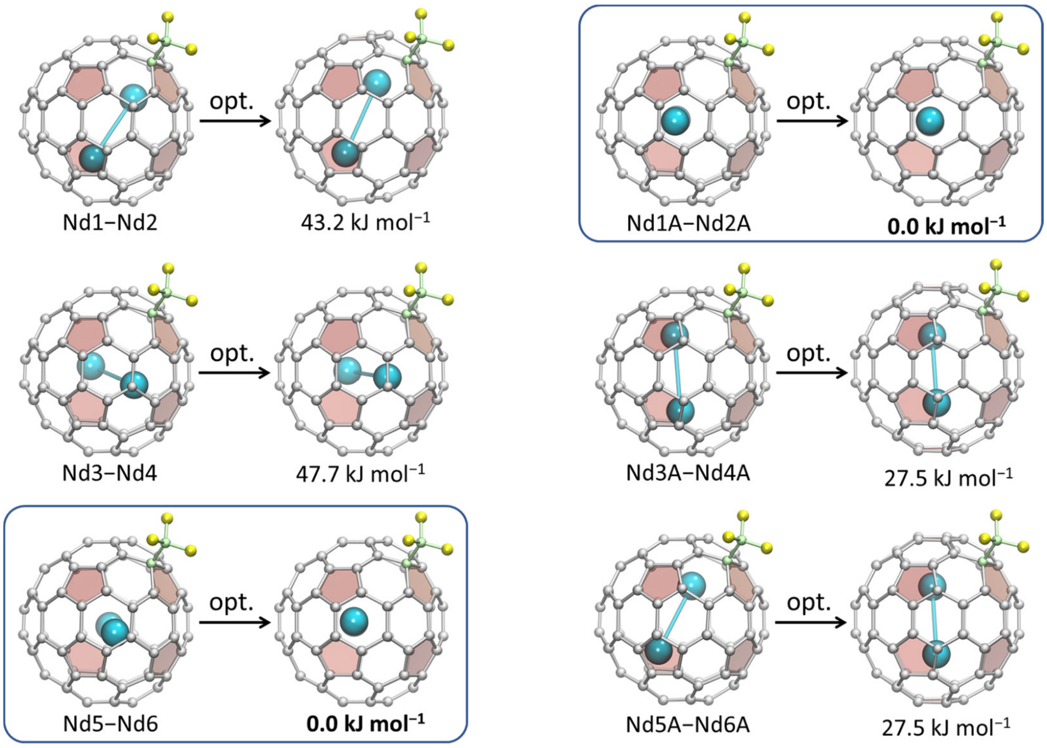

Figure S22. DFT-optimization of $Nd_2@D_{5h}$-$C_{80}(CF_3)$ structures, for which starting coordinates were based on one of the cage orientations from the SC-XRD structure combined with each of 6 pairs of Nd sites. 3 unique Nd pairs are designated as Nd1–Nd2, Nd3–Nd4, Nd5–Nd6, and 3 symmetry replicas are denoted by addition of "A", such as Nd1A–Nd2A. Ascribing metal dimers to particular cage orientations is not possible solely on the base of SC-XRD data. We therefore performed DFT calculations to aid the assignment. Optimized structures are shown with their relative energies. Nd1A–Nd2A and Nd5–Nd6 clearly correspond to the lowest-energy conformer of $Nd_2@D_{5h}$-$C_{80}(CF_3)$. For Nd3–Nd4/Nd3A–Nd4A pairs, assignment is more ambiguous as neither gives the most stable conformer. Although Nd3A–Nd4A gives more stable structure after optimization, Nd3–Nd4 pair is more preferred as it is close to the metal positions of Nd1A–Nd2A and Nd5–Nd6 pairs. The disorder of Nd_2 positions may be then caused by a large-amplitude motion of the Nd_2 dimer near its minimum.

References

1. Mueller, U.; Förster, R.; Hellmig, M.; Huschmann, F. U.; Kastner, A.; Malecki, P.; Pühringer, S.; Röwer, M.; Sparta, K.; Steffien, M.; Ühlein, M.; Wilk, P.; Weiss, M. S., The macromolecular crystallography beamlines at BESSY II of the Helmholtz-Zentrum Berlin: Current status and perspectives. *Eur. Phys. J. Plus* **2015,** *130* (7), 141.
2. Kabsch, W., XDS. *Acta Cryst. D* **2010,** *66* (2), 125-132.
3. Sparta, K. M.; Krug, M.; Heinemann, U.; Mueller, U.; Weiss, M. S., XDSAPP2.0. *J. Appl. Crystallogr.* **2016,** *49* (3), 1085-1092.
4. Sheldrick, G., Crystal structure refinement with SHELXL. *Acta Cryst. C* **2015,** *71* (1), 3-8.
5. Chilton, N. F.; Anderson, R. P.; Turner, L. D.; Soncini, A.; Murray, K. S., PHI: A powerful new program for the analysis of anisotropic monomeric and exchange-coupled polynuclear d- and f-block complexes. *J. Comput. Chem.* **2013,** *34* (13), 1164-1175.
6. Perdew, J. P.; Burke, K.; Ernzerhof, M., Generalized gradient approximation made simple. *Phys. Rev. Lett.* **1996,** *77* (18), 3865-3868.
7. Neese, F.; Wennmohs, F.; Becker, U.; Riplinger, C., The ORCA quantum chemistry program package. *J. Chem. Phys.* **2020,** *152* (22), 224108.
8. Neese, F., Software update: the ORCA program system, version 4.0. *WIREs Comput. Mol. Sci.* **2018,** *8* (1), e1327.
9. Dolg, M.; Stoll, H.; Savin, A.; Preuss, H., Energy-adjusted pseudopotentials for the rare earth elements. *Theor. Chim. Acta* **1989,** *75* (3), 173-194.
10. Yang, J.; Dolg, M., Valence basis sets for lanthanide 4f-in-core pseudopotentials adapted for crystal orbital ab initio calculations. *Theor. Chem. Acc.* **2005,** *113* (4), 212-224.
11. Weigend, F.; Ahlrichs, R., Balanced basis sets of split valence, triple zeta valence and quadruple zeta valence quality for H to Rn: Design and assessment of accuracy. *Phys. Chem. Chem. Phys.* **2005,** *7*, 3297-3305.
12. Humphrey, W.; Dalke, A.; Schulten, K., VMD - Visual Molecular Dynamics. *J. Molec. Graphics* **1996,** *14*, 33-38.
13. Nishibori, E.; Takata, M.; Sakata, M.; Taninaka, A.; Shinohara, H., Pentagonal-dodecahedral La_2 charge density in [80-I_h]fullerene: $La_2@C_{80}$. *Angew. Chem. Int. Ed.* **2001,** *40* (16), 2998-2999.
14. Yamada, M.; Okamura, M.; Sato, S.; Someya, C. I.; Mizorogi, N.; Tsuchiya, T.; Akasaka, T.; Kato, T.; Nagase, S., Two Regioisomers of Endohedral Pyrrolidinodimetallofullerenes $M_2@I_h$-$C_{80}(CH_2)_2$NTrt (M = La, Ce; Trt = trityl): Control of Metal Atom Positions by Addition Positions. *Chem. Eur. J.* **2009,** *15* (40), 10533-10542.
15. Yamada, M.; Wakahara, T.; Nakahodo, T.; Tsuchiya, T.; Maeda, Y.; Akasaka, T.; Yoza, K.; Horn, E.; Mizorogi, N.; Nagase, S., Synthesis and structural characterization of endohedral pyrrolidinodimetallofullerene: $La_2@C_{80}(CH_2)_2$NTrt. *J. Am. Chem. Soc.* **2006,** *128* (5), 1402-1403.
16. Wakahara, T.; Yamada, M.; Takahashi, S.; Nakahodo, T.; Tsuchiya, T.; Maeda, Y.; Akasaka, T.; Kako, M.; Yoza, K.; Horn, E.; Mizorogi, N.; Nagase, S., Two-dimensional hopping motion of encapsulated La atoms in silylated $La_2@C_{80}$. *Chem. Commun.* **2007**, (26), 2680-2682.
17. Yamada, M.; Minowa, M.; Sato, S.; Kako, M.; Slanina, Z.; Mizorogi, N.; Tsuchiya, T.; Maeda, Y.; Nagase, S.; Akasaka, T., Thermal Carbosilylation of Endohedral Dimetallofullerene $La_2@I_h$-C_{80} with Silirane. *J. Am. Chem. Soc.* **2010,** *132* (50), 17953-17960.
18. Yamada, M.; Someya, C.; Wakahara, T.; Tsuchiya, T.; Maeda, Y.; Akasaka, T.; Yoza, K.; Horn, E.; Liu, M. T. H.; Mizorogi, N.; Nagase, S., Metal atoms collinear with the spiro carbon of 6,6-open adducts, $M_2@C_{80}$(Ad) (M = La and Ce, Ad = adamantylidene). *J. Am. Chem. Soc.* **2008,** *130* (4), 1171-1176.
19. Ishitsuka, M. O.; Sano, S.; Enoki, H.; Sato, S.; Nikawa, H.; Tsuchiya, T.; Slanina, Z.; Mizorogi, N.; Liu, M. T. H.; Akasaka, T.; Nagase, S., Regioselective Bis-functionalization of Endohedral Dimetallofullerene, $La_2@C_{80}$: Extremal La–La Distance. *J. Am. Chem. Soc.* **2011,** *133* (18), 7128-34.

20. Yamada, M.; Minowa, M.; Sato, S.; Slanina, Z.; Tsuchiya, T.; Maeda, Y.; Nagase, S.; Akasaka, T., Regioselective Cycloaddition of $La_2@I_h$-C_{80} with Tetracyanoethylene Oxide: Formation of an Endohedral Dimetallofullerene Adduct Featuring Enhanced Electron-Accepting Character. *J. Am. Chem. Soc.* **2011,** *133* (11), 3796-3799.
21. Bao, L.; Chen, M.; Pan, C.; Yamaguchi, T.; Kato, T.; Olmstead, M. M.; Balch, A. L.; Akasaka, T.; Lu, X., Crystallographic Evidence for Direct Metal–Metal Bonding in a Stable Open-Shell $La_2@I_h$-C_{80} Derivative. *Angew. Chem. Int. Ed.* **2016,** *55* (13), 4242-4246.
22. Yamada, M.; Kurihara, H.; Suzuki, M.; Saito, M.; Slanina, Z.; Uhlik, F.; Aizawa, T.; Kato, T.; Olmstead, M. M.; Balch, A. L.; Maeda, Y.; Nagase, S.; Lu, X.; Akasaka, T., Hiding and Recovering Electrons in a Dimetallic Endohedral Fullerene: Air-Stable Products from Radical Additions. *J. Am. Chem. Soc.* **2015,** *137* (1), 232-238.
23. Feng, L.; Suzuki, M.; Mizorogi, N.; Lu, X.; Yamada, M.; Akasaka, T.; Nagase, S., Mapping the Metal Positions inside Spherical C_{80} Cages: Crystallographic and Theoretical Studies of $Ce_2@D_{5h}$-C_{80} and $Ce_2@I_h$-C_{80}. *Chem.-Eur. J.* **2013,** *19* (3), 988–993.
24. Yamada, M.; Nakahodo, T.; Wakahara, T.; Tsuchiya, T.; Maeda, Y.; Akasaka, T.; Kako, M.; Yoza, K.; Horn, E.; Mizorogi, N.; Kobayashi, K.; Nagase, S., Positional control of encapsulated atoms inside a fullerene cage by exohedral addition. *J. Am. Chem. Soc.* **2005,** *127* (42), 14570-14571.
25. Guldi, D. M.; Feng, L.; Radhakrishnan, S. G.; Nikawa, H.; Yamada, M.; Mizorogi, N.; Tsuchiya, T.; Akasaka, T.; Nagase, S.; Herranz, M. A.; Martin, N., A Molecular $Ce_2@I_h$-C_{80} Switch-Unprecedented Oxidative Pathway in Photoinduced Charge Transfer Reactivity. *J. Am. Chem. Soc.* **2010,** *132* (26), 9078-9086.
26. Hu, Z.; Dong, B.-W.; Liu, Z.; Liu, J.-J.; Su, J.; Yu, C.; Xiong, J.; Shi, D.-E.; Wang, Y.; Wang, B.-W.; Ardavan, A.; Shi, Z.; Jiang, S.-D.; Gao, S., Endohedral Metallofullerene as Molecular High Spin Qubit: Diverse Rabi Cycles in $Gd_2@C_{79}N$. *J. Am. Chem. Soc.* **2018,** *140*, 1123-1130.
27. Zuo, T.; Xu, L.; Beavers, C. M.; Olmstead, M. M.; Fu, W.; Crawford, T. D.; Balch, A. L.; Dorn, H. C., $M_2@C_{79}N$ (M = Y, Tb): Isolation and Characterization of Stable Endohedral Metallofullerenes Exhibiting M...M Bonding Interactions inside Aza[80]fullerene Cages. *J. Am. Chem. Soc.* **2008,** *130* (39), 12992-12997.
28. Liu, F.; Krylov, D. S.; Spree, L.; Avdoshenko, S. M.; Samoylova, N. A.; Rosenkranz, M.; Kostanyan, A.; Greber, T.; Wolter, A. U. B.; Büchner, B.; Popov, A. A., Single molecule magnet with an unpaired electron trapped between two lanthanide ions inside a fullerene. *Nat. Commun.* **2017,** *8*, 16098.
29. Wang, Y.; Xiong, J.; Su, J.; Hu, Z.-Q.; Ma, F.; Sun, R.; Tan, X.-Y.; Sun, H.-L.; Wang, B.; Shi, Z.; Gao, S., $Dy_2@C_{79}N$: A New Member of Dimetalloazafullerenes with Strong Single Molecular Magnetism. *Nanoscale* **2020,** *12*, 11130-11135.
