# Supplementary material for: Nd─Nd Bond in Ih and D5h Cage Isomers of Nd2@C80 Stabilized by Electrophilic CF3 Addition
Source: Adv Sci (Weinh). 2023 Nov 9;11(1):2305190. doi: 10.1002/advs.202305190 (PMC10767449; doi:10.1002/advs.202305190)

checkCIF/PLATON report

Structure factors have been supplied for datablock(s) nd2@c80-cf3-ii

THIS REPORT IS FOR GUIDANCE ONLY. IF USED AS PART OF A REVIEW PROCEDURE FOR PUBLICATION, IT SHOULD NOT REPLACE THE EXPERTISE OF AN EXPERIENCED CRYSTALLOGRAPHIC REFEREE.

No syntax errors found. CIF dictionary Interpreting this report

Datablock: nd2@c80-cf3-ii

Bond precision: = 0.0000 A Wavelength=0.77977

Cell:	a=21.050(4)	b=20.580(4)	c=23.840(5)
	alpha=90	beta=96.63(3)	gamma=90
Temperature:	100 K		

	Calculated	Reported
Volume	10259(4)	10259(4)
Space group	C 2/c	C 2/c
Hall group	-C 2yc	-C 2yc
Moiety formula	C81 F3 Nd2, 2(C36 H44 N4 Ni), C6 H6	;Nd2@D5H(6)-C80-Cf3/2NiOeP/BeNzEnE;
Sum formula	C159 H94 F3 N8 Nd2 Ni2	C159 H94 F3 N8 Nd2 Ni2
Mr	2579.17	2579.32
Dx,g cm-3	1.670	1.670
Z	4	4
Mu (mm-1)	1.820	1.830
F000	5227.8	5228.0
F000'	5232.13	
h,k,lmax	32,31,36	28,30,35
Nref	19531	16888
Tmin,Tmax	0.848,0.880	
Tmin'	0.848	

Correction method= Not given

Data completeness= 0.865 Theta(max)= 36.830

```
R(reflections)= 0.0692( 15642)          wR2(reflections)= 0.1961( 16888)
S = 1.061                  Npar= 1614
```

The following ALERTS were generated. Each ALERT has the format
test-name_ALERT_alert-type_alert-level.
Click on the hyperlinks for more details of the test.

Alert level C

```
SHFSU01_ALERT_2_C  The absolute value of parameter shift to su ratio > 0.05
            Absolute value of the parameter shift to su ratio given   0.066
            Additional refinement cycles may be required.
PLAT029_ALERT_3_C _diffrn_measured_fraction_theta_full value Low .      0.976 Why?
PLAT052_ALERT_1_C Info on Absorption Correction Method   Not Given     Please Do !
PLAT080_ALERT_2_C Maximum Shift/Error ...........................       0.07 Why ?
PLAT094_ALERT_2_C Ratio of Maximum / Minimum Residual Density ....       3.50 Report
PLAT215_ALERT_3_C Disordered C8        has ADP max/min Ratio .....        3.1 Note
PLAT215_ALERT_3_C Disordered C14       has ADP max/min Ratio .....        3.2 Note
PLAT215_ALERT_3_C Disordered C18       has ADP max/min Ratio .....        3.4 Note
PLAT215_ALERT_3_C Disordered C19       has ADP max/min Ratio .....        3.1 Note
PLAT215_ALERT_3_C Disordered C28       has ADP max/min Ratio .....        3.4 Note
PLAT215_ALERT_3_C Disordered C29       has ADP max/min Ratio .....        3.9 Note
PLAT215_ALERT_3_C Disordered C39       has ADP max/min Ratio .....        3.1 Note
PLAT215_ALERT_3_C Disordered C46       has ADP max/min Ratio .....        3.5 Note
PLAT215_ALERT_3_C Disordered C54       has ADP max/min Ratio .....        3.1 Note
PLAT215_ALERT_3_C Disordered C57       has ADP max/min Ratio .....        3.5 Note
PLAT215_ALERT_3_C Disordered C58       has ADP max/min Ratio .....        3.6 Note
PLAT215_ALERT_3_C Disordered C60       has ADP max/min Ratio .....        3.5 Note
PLAT215_ALERT_3_C Disordered C61       has ADP max/min Ratio .....        3.9 Note
PLAT215_ALERT_3_C Disordered C63       has ADP max/min Ratio .....        3.9 Note
PLAT215_ALERT_3_C Disordered C70       has ADP max/min Ratio .....        3.2 Note
PLAT250_ALERT_2_C Large U3/U1 Ratio for Average U(i,j) Tensor ....        2.2 Note
PLAT723_ALERT_1_C Torsion Calc      -12.00, Rep     -14(**) Dev...       2.00 Sigma
              C53 -C11 -C16 -C49   2_655  1_555  1_555  2_655      # 1208 Check
PLAT723_ALERT_1_C Torsion Calc      -43.00, Rep     -45(**) Dev...       2.00 Sigma
              C53 -C11 -C16 -C17   2_655  1_555  1_555  1_555      # 1215 Check
PLAT723_ALERT_1_C Torsion Calc      163.00, Rep     161(**) Dev...       2.00 Sigma
              C53 -C11 -C16 -C15   2_655  1_555  1_555  1_555      # 1222 Check
PLAT723_ALERT_1_C Torsion Calc      169.00, Rep     166(**) Dev...       3.00 Sigma
              C53 -C11 -C16 -C29   2_655  1_555  1_555  2_655      # 1229 Check
PLAT723_ALERT_1_C Torsion Calc      -46.00, Rep     -48(**) Dev...       2.00 Sigma
              C53 -C11 -C16 -C52   2_655  1_555  1_555  2_655      # 1236 Check
PLAT723_ALERT_1_C Torsion Calc     -147.00, Rep    -149(**) Dev...       2.00 Sigma
              C53 -C11 -C16 -ND4   2_655  1_555  1_555  2_655      # 1243 Check
PLAT723_ALERT_1_C Torsion Calc       70.00, Rep      73(32) Dev...       3.00 Sigma
              C15 -C28 -C29 -C20   2_655  1_555  1_555  2_655      # 2280 Check
PLAT723_ALERT_1_C Torsion Calc      152.00, Rep     155(33) Dev...       3.00 Sigma
              C15 -C28 -C29 -C53   2_655  1_555  1_555  1_555      # 2288 Check
PLAT723_ALERT_1_C Torsion Calc      -60.00, Rep     -58(31) Dev...       2.00 Sigma
              C15 -C28 -C29 -C14   2_655  1_555  1_555  2_655      # 2296 Check
PLAT723_ALERT_1_C Torsion Calc      -54.00, Rep     -51(32) Dev...       3.00 Sigma
              C15 -C28 -C29 -C30   2_655  1_555  1_555  1_555      # 2304 Check
PLAT723_ALERT_1_C Torsion Calc      162.00, Rep     165(33) Dev...       3.00 Sigma
              C15 -C28 -C29 -C16   2_655  1_555  1_555  2_655      # 2312 Check
```

```
PLAT723_ALERT_1_C Torsion Calc     -112.00, Rep    -109(32) Dev...       3.00 Sigma
              C15 -C28 -C29 -ND4   2_655  1_555  1_555  1_555      # 2320 Check
PLAT723_ALERT_1_C Torsion Calc     -105.00, Rep    -102(32) Dev...       3.00 Sigma
              C15 -C28 -C29 -ND1   2_655  1_555  1_555  2_655      # 2327 Check
PLAT723_ALERT_1_C Torsion Calc      104.00, Rep      98(52) Dev...       6.00 Sigma
              C24 -C57 -C58 -C25   2_655  1_555  1_555  2_655      # 5023 Check
PLAT723_ALERT_1_C Torsion Calc      -84.00, Rep     -91(52) Dev...       7.00 Sigma
              C24 -C57 -C58 -C54   2_655  1_555  1_555  1_555      # 5033 Check
PLAT723_ALERT_1_C Torsion Calc       62.00, Rep      56(52) Dev...       6.00 Sigma
              C24 -C57 -C58 -C22   2_655  1_555  1_555  1_555      # 5043 Check
PLAT723_ALERT_1_C Torsion Calc      -78.00, Rep     -85(52) Dev...       7.00 Sigma
              C24 -C57 -C58 -C19   2_655  1_555  1_555  2_655      # 5053 Check
PLAT723_ALERT_1_C Torsion Calc       54.00, Rep      47(52) Dev...       7.00 Sigma
              C24 -C57 -C58 -C23   2_655  1_555  1_555  2_655      # 5063 Check
PLAT723_ALERT_1_C Torsion Calc        3.00, Rep      -3(52) Dev...       6.00 Sigma
              C24 -C57 -C58 -ND5   2_655  1_555  1_555  2_655      # 5073 Check
PLAT723_ALERT_1_C Torsion Calc       16.00, Rep       9(52) Dev...       7.00 Sigma
              C24 -C57 -C58 -ND1   2_655  1_555  1_555  1_555      # 5082 Check
PLAT723_ALERT_1_C Torsion Calc     -105.00, Rep    -107(30) Dev...       2.00 Sigma
              C64 -C75 -C76 -C51   2_655  1_555  1_555  2_655      # 7516 Check
PLAT723_ALERT_1_C Torsion Calc      170.00, Rep     167(30) Dev...       3.00 Sigma
              C63 -C75 -C76 -C51   2_655  1_555  1_555  2_655      # 7517 Check
PLAT723_ALERT_1_C Torsion Calc      101.00, Rep      99(30) Dev...       2.00 Sigma
              C77 -C75 -C76 -C51   1_555  1_555  1_555  2_655      # 7518 Check
PLAT723_ALERT_1_C Torsion Calc     -114.00, Rep    -116(30) Dev...       2.00 Sigma
              C74 -C75 -C76 -C51   1_555  1_555  1_555  2_655      # 7519 Check
PLAT723_ALERT_1_C Torsion Calc      109.00, Rep     107(30) Dev...       2.00 Sigma
              C56 -C75 -C76 -C51   2_655  1_555  1_555  2_655      # 7520 Check
PLAT723_ALERT_1_C Torsion Calc      105.00, Rep     107(30) Dev...       2.00 Sigma
              C51 -C75 -C76 -C64   2_655  1_555  1_555  2_655      # 7524 Check
PLAT723_ALERT_1_C Torsion Calc      -97.00, Rep     -95(30) Dev...       2.00 Sigma
              C51 -C75 -C76 -C17   2_655  1_555  1_555  1_555      # 7530 Check
PLAT723_ALERT_1_C Torsion Calc      116.00, Rep     118(31) Dev...       2.00 Sigma
              C51 -C75 -C76 -C71   2_655  1_555  1_555  1_555      # 7536 Check
PLAT723_ALERT_1_C Torsion Calc      144.00, Rep     146(32) Dev...       2.00 Sigma
              C51 -C75 -C76 -C50   2_655  1_555  1_555  2_655      # 7542 Check
PLAT723_ALERT_1_C Torsion Calc      -95.00, Rep     -93(30) Dev...       2.00 Sigma
              C51 -C75 -C76 -C52   2_655  1_555  1_555  2_655      # 7548 Check
PLAT906_ALERT_3_C Large K Value in the Analysis of Variance ......      2.082 Check
PLAT911_ALERT_3_C Missing FCF Refl Between Thmin & STh/L=    0.600        219 Report
PLAT927_ALERT_1_C Reported and Calculated  wR2 Differ by .........    -0.0016 Check
PLAT971_ALERT_2_C Check Calcd Resid. Dens.  0.78Ang From C37           2.09 eA-3
PLAT971_ALERT_2_C Check Calcd Resid. Dens.  0.56Ang From C7            1.71 eA-3
PLAT971_ALERT_2_C Check Calcd Resid. Dens.  0.95Ang From C50           1.66 eA-3
PLAT971_ALERT_2_C Check Calcd Resid. Dens.  0.65Ang From C60           1.65 eA-3
PLAT971_ALERT_2_C Check Calcd Resid. Dens.  0.87Ang From C35           1.65 eA-3
PLAT971_ALERT_2_C Check Calcd Resid. Dens.  1.06Ang From C41           1.56 eA-3
```

Alert level G

```
FORMU01_ALERT_1_G  There is a discrepancy between the atom counts in the
            _chemical_formula_sum and _chemical_formula_moiety. This is
            usually due to the moiety formula being in the wrong format.
            Atom count from _chemical_formula_sum:   C159 H94 F3 N8 Nd2 Ni2
            Atom count from _chemical_formula_moiety:
ABSMU01_ALERT_1_G  Calculation of _exptl_absorpt_correction_mu
                not performed for this radiation type.
```

```
PLAT002_ALERT_2_G Number of Distance or Angle Restraints on AtSite        160 Note
PLAT003_ALERT_2_G Number of Uiso or Uij Restrained non-H Atoms ...        158 Report
PLAT042_ALERT_1_G Calc. and Reported Moiety Formula Strings Differ     Please Check
PLAT068_ALERT_1_G Reported F000 Differs from Calcd (or Missing)...     Please Check
PLAT083_ALERT_2_G SHELXL Second Parameter in WGHT  Unusually Large      37.84 Why ?
PLAT092_ALERT_4_G Check: Wavelength Given is not Cu,Ga,Mo,Ag,In Ka    0.77977 Ang.
PLAT175_ALERT_4_G The CIF-Embedded .res File Contains SAME Records          1 Report
PLAT176_ALERT_4_G The CIF-Embedded .res File Contains SADI Records         68 Report
PLAT178_ALERT_4_G The CIF-Embedded .res File Contains SIMU Records          7 Report
PLAT180_ALERT_4_G Check Cell Rounding: # of Values Ending with 0 =          3 Note
PLAT186_ALERT_4_G The CIF-Embedded .res File Contains ISOR Records          3 Report
PLAT300_ALERT_4_G Atom Site Occupancy of F1         Constrained at        0.5 Check
PLAT300_ALERT_4_G Atom Site Occupancy of F2         Constrained at        0.5 Check
PLAT300_ALERT_4_G Atom Site Occupancy of F3         Constrained at        0.5 Check
PLAT300_ALERT_4_G Atom Site Occupancy of C1         Constrained at        0.5 Check
PLAT300_ALERT_4_G Atom Site Occupancy of C2         Constrained at        0.5 Check
PLAT300_ALERT_4_G Atom Site Occupancy of C3         Constrained at        0.5 Check
PLAT300_ALERT_4_G Atom Site Occupancy of C4         Constrained at        0.5 Check
PLAT300_ALERT_4_G Atom Site Occupancy of C5         Constrained at        0.5 Check
PLAT300_ALERT_4_G Atom Site Occupancy of C6         Constrained at        0.5 Check
PLAT300_ALERT_4_G Atom Site Occupancy of C7         Constrained at        0.5 Check
PLAT300_ALERT_4_G Atom Site Occupancy of C8         Constrained at        0.5 Check
PLAT300_ALERT_4_G Atom Site Occupancy of C9         Constrained at        0.5 Check
PLAT300_ALERT_4_G Atom Site Occupancy of C10        Constrained at        0.5 Check
PLAT300_ALERT_4_G Atom Site Occupancy of C11        Constrained at        0.5 Check
PLAT300_ALERT_4_G Atom Site Occupancy of C12        Constrained at        0.5 Check
PLAT300_ALERT_4_G Atom Site Occupancy of C13        Constrained at        0.5 Check
PLAT300_ALERT_4_G Atom Site Occupancy of C14        Constrained at        0.5 Check
PLAT300_ALERT_4_G Atom Site Occupancy of C15        Constrained at        0.5 Check
PLAT300_ALERT_4_G Atom Site Occupancy of C16        Constrained at        0.5 Check
PLAT300_ALERT_4_G Atom Site Occupancy of C17        Constrained at        0.5 Check
PLAT300_ALERT_4_G Atom Site Occupancy of C18        Constrained at        0.5 Check
PLAT300_ALERT_4_G Atom Site Occupancy of C19        Constrained at        0.5 Check
PLAT300_ALERT_4_G Atom Site Occupancy of C20        Constrained at        0.5 Check
PLAT300_ALERT_4_G Atom Site Occupancy of C21        Constrained at        0.5 Check
PLAT300_ALERT_4_G Atom Site Occupancy of C22        Constrained at        0.5 Check
PLAT300_ALERT_4_G Atom Site Occupancy of C23        Constrained at        0.5 Check
PLAT300_ALERT_4_G Atom Site Occupancy of C24        Constrained at        0.5 Check
PLAT300_ALERT_4_G Atom Site Occupancy of C25        Constrained at        0.5 Check
PLAT300_ALERT_4_G Atom Site Occupancy of C26        Constrained at        0.5 Check
PLAT300_ALERT_4_G Atom Site Occupancy of C27        Constrained at        0.5 Check
PLAT300_ALERT_4_G Atom Site Occupancy of C28        Constrained at        0.5 Check
PLAT300_ALERT_4_G Atom Site Occupancy of C29        Constrained at        0.5 Check
PLAT300_ALERT_4_G Atom Site Occupancy of C30        Constrained at        0.5 Check
PLAT300_ALERT_4_G Atom Site Occupancy of C31        Constrained at        0.5 Check
PLAT300_ALERT_4_G Atom Site Occupancy of C32        Constrained at        0.5 Check
PLAT300_ALERT_4_G Atom Site Occupancy of C33        Constrained at        0.5 Check
PLAT300_ALERT_4_G Atom Site Occupancy of C34        Constrained at        0.5 Check
PLAT300_ALERT_4_G Atom Site Occupancy of C35        Constrained at        0.5 Check
PLAT300_ALERT_4_G Atom Site Occupancy of C36        Constrained at        0.5 Check
PLAT300_ALERT_4_G Atom Site Occupancy of C37        Constrained at        0.5 Check
PLAT300_ALERT_4_G Atom Site Occupancy of C38        Constrained at        0.5 Check
PLAT300_ALERT_4_G Atom Site Occupancy of C39        Constrained at        0.5 Check
PLAT300_ALERT_4_G Atom Site Occupancy of C40        Constrained at        0.5 Check
PLAT300_ALERT_4_G Atom Site Occupancy of C41        Constrained at        0.5 Check
PLAT300_ALERT_4_G Atom Site Occupancy of C42        Constrained at        0.5 Check
PLAT300_ALERT_4_G Atom Site Occupancy of C43        Constrained at        0.5 Check
```

```
PLAT300_ALERT_4_G Atom Site Occupancy of C44        Constrained at        0.5 Check
PLAT300_ALERT_4_G Atom Site Occupancy of C45        Constrained at        0.5 Check
PLAT300_ALERT_4_G Atom Site Occupancy of C46        Constrained at        0.5 Check
PLAT300_ALERT_4_G Atom Site Occupancy of C47        Constrained at        0.5 Check
PLAT300_ALERT_4_G Atom Site Occupancy of C48        Constrained at        0.5 Check
PLAT300_ALERT_4_G Atom Site Occupancy of C49        Constrained at        0.5 Check
PLAT300_ALERT_4_G Atom Site Occupancy of C50        Constrained at        0.5 Check
PLAT300_ALERT_4_G Atom Site Occupancy of C51        Constrained at        0.5 Check
PLAT300_ALERT_4_G Atom Site Occupancy of C52        Constrained at        0.5 Check
PLAT300_ALERT_4_G Atom Site Occupancy of C53        Constrained at        0.5 Check
PLAT300_ALERT_4_G Atom Site Occupancy of C54        Constrained at        0.5 Check
PLAT300_ALERT_4_G Atom Site Occupancy of C55        Constrained at        0.5 Check
PLAT300_ALERT_4_G Atom Site Occupancy of C56        Constrained at        0.5 Check
PLAT300_ALERT_4_G Atom Site Occupancy of C57        Constrained at        0.5 Check
PLAT300_ALERT_4_G Atom Site Occupancy of C58        Constrained at        0.5 Check
PLAT300_ALERT_4_G Atom Site Occupancy of C59        Constrained at        0.5 Check
PLAT300_ALERT_4_G Atom Site Occupancy of C60        Constrained at        0.5 Check
PLAT300_ALERT_4_G Atom Site Occupancy of C61        Constrained at        0.5 Check
PLAT300_ALERT_4_G Atom Site Occupancy of C62        Constrained at        0.5 Check
PLAT300_ALERT_4_G Atom Site Occupancy of C63        Constrained at        0.5 Check
PLAT300_ALERT_4_G Atom Site Occupancy of C64        Constrained at        0.5 Check
PLAT300_ALERT_4_G Atom Site Occupancy of C65        Constrained at        0.5 Check
PLAT300_ALERT_4_G Atom Site Occupancy of C66        Constrained at        0.5 Check
PLAT300_ALERT_4_G Atom Site Occupancy of C67        Constrained at        0.5 Check
PLAT300_ALERT_4_G Atom Site Occupancy of C68        Constrained at        0.5 Check
PLAT300_ALERT_4_G Atom Site Occupancy of C69        Constrained at        0.5 Check
PLAT300_ALERT_4_G Atom Site Occupancy of C70        Constrained at        0.5 Check
PLAT300_ALERT_4_G Atom Site Occupancy of C71        Constrained at        0.5 Check
PLAT300_ALERT_4_G Atom Site Occupancy of C72        Constrained at        0.5 Check
PLAT300_ALERT_4_G Atom Site Occupancy of C73        Constrained at        0.5 Check
PLAT300_ALERT_4_G Atom Site Occupancy of C74        Constrained at        0.5 Check
PLAT300_ALERT_4_G Atom Site Occupancy of C75        Constrained at        0.5 Check
PLAT300_ALERT_4_G Atom Site Occupancy of C76        Constrained at        0.5 Check
PLAT300_ALERT_4_G Atom Site Occupancy of C77        Constrained at        0.5 Check
PLAT300_ALERT_4_G Atom Site Occupancy of C78        Constrained at        0.5 Check
PLAT300_ALERT_4_G Atom Site Occupancy of C79        Constrained at        0.5 Check
PLAT300_ALERT_4_G Atom Site Occupancy of C80        Constrained at        0.5 Check
PLAT300_ALERT_4_G Atom Site Occupancy of C81        Constrained at        0.5 Check
PLAT300_ALERT_4_G Atom Site Occupancy of C1S        Constrained at        0.5 Check
PLAT300_ALERT_4_G Atom Site Occupancy of C2S        Constrained at        0.5 Check
PLAT300_ALERT_4_G Atom Site Occupancy of C3S        Constrained at        0.5 Check
PLAT300_ALERT_4_G Atom Site Occupancy of C4S        Constrained at        0.5 Check
PLAT300_ALERT_4_G Atom Site Occupancy of C5S        Constrained at        0.5 Check
PLAT300_ALERT_4_G Atom Site Occupancy of C6S        Constrained at        0.5 Check
PLAT300_ALERT_4_G Atom Site Occupancy of H1S        Constrained at        0.5 Check
PLAT300_ALERT_4_G Atom Site Occupancy of H2S        Constrained at        0.5 Check
PLAT300_ALERT_4_G Atom Site Occupancy of H3S        Constrained at        0.5 Check
PLAT300_ALERT_4_G Atom Site Occupancy of H4S        Constrained at        0.5 Check
PLAT300_ALERT_4_G Atom Site Occupancy of H5S        Constrained at        0.5 Check
PLAT300_ALERT_4_G Atom Site Occupancy of H6S        Constrained at        0.5 Check
PLAT301_ALERT_3_G Main Residue  Disorder .............(Resd  1  )       100% Note
PLAT302_ALERT_4_G Anion/Solvent/Minor-Residue Disorder (Resd  2  )       100% Note
PLAT302_ALERT_4_G Anion/Solvent/Minor-Residue Disorder (Resd  3  )       100% Note
PLAT302_ALERT_4_G Anion/Solvent/Minor-Residue Disorder (Resd  4  )       100% Note
PLAT304_ALERT_4_G Non-Integer Number of Atoms in ..... (Resd  2  )      72.28 Check
PLAT304_ALERT_4_G Non-Integer Number of Atoms in ..... (Resd  3  )      12.72 Check
PLAT333_ALERT_2_G Large Aver C6-Ring C-C Dist C2       -C41      .       1.43 Ang.
```

```
PLAT333_ALERT_2_G Large Aver C6-Ring C-C Dist C3       -C72      .       1.43 Ang.
PLAT333_ALERT_2_G Large Aver C6-Ring C-C Dist C4       -C9       .       1.45 Ang.
PLAT335_ALERT_2_G Check Large C6 Ring C-C Range C4       -C9             0.17 Ang.
PLAT335_ALERT_2_G Check Large C6 Ring C-C Range C7       -C68            0.18 Ang.
PLAT764_ALERT_4_G Overcomplete CIF Bond List Detected (Rep/Expd) .       2.31 Ratio
PLAT773_ALERT_2_G Check long C-C Bond in CIF: C1       --C2              1.78 Ang.
PLAT773_ALERT_2_G Check long C-C Bond in CIF: C1       --C5              1.80 Ang.
PLAT773_ALERT_2_G Check long C-C Bond in CIF: C3       --C47             1.87 Ang.
PLAT773_ALERT_2_G Check long C-C Bond in CIF: C3       --C43             2.02 Ang.
PLAT773_ALERT_2_G Check long C-C Bond in CIF: C4       --C47             1.83 Ang.
PLAT773_ALERT_2_G Check long C-C Bond in CIF: C4       --C45             1.94 Ang.
PLAT773_ALERT_2_G Check long C-C Bond in CIF: C4       --C42             2.04 Ang.
PLAT773_ALERT_2_G Check long C-C Bond in CIF: C5       --C41             1.91 Ang.
PLAT773_ALERT_2_G Check long C-C Bond in CIF: C6       --C43             1.74 Ang.
PLAT773_ALERT_2_G Check long C-C Bond in CIF: C6       --C37             1.77 Ang.
PLAT773_ALERT_2_G Check long C-C Bond in CIF: C6       --C40             2.03 Ang.
PLAT773_ALERT_2_G Check long C-C Bond in CIF: C7       --C36             1.90 Ang.
PLAT773_ALERT_2_G Check long C-C Bond in CIF: C7       --C39             2.02 Ang.
PLAT773_ALERT_2_G Check long C-C Bond in CIF: C10      --C35             1.93 Ang.
PLAT773_ALERT_2_G Check long C-C Bond in CIF: C10      --C36             1.97 Ang.
PLAT773_ALERT_2_G Check long C-C Bond in CIF: C11      --C53             1.79 Ang.
PLAT773_ALERT_2_G Check long C-C Bond in CIF: C12      --C49             1.83 Ang.
PLAT773_ALERT_2_G Check long C-C Bond in CIF: C13      --C35             1.76 Ang.
PLAT773_ALERT_2_G Check long C-C Bond in CIF: C13      --C32             1.88 Ang.
PLAT773_ALERT_2_G Check long C-C Bond in CIF: C13      --C31             1.97 Ang.
PLAT773_ALERT_2_G Check long C-C Bond in CIF: C17      --C50             2.03 Ang.
PLAT773_ALERT_2_G Check long C-C Bond in CIF: C20      --C27             1.71 Ang.
PLAT773_ALERT_2_G Check long C-C Bond in CIF: C22      --C22             1.75 Ang.
PLAT773_ALERT_2_G Check long C-C Bond in CIF: C22      --C26             1.97 Ang.
PLAT773_ALERT_2_G Check long C-C Bond in CIF: C26      --C57             1.93 Ang.
PLAT773_ALERT_2_G Check long C-C Bond in CIF: C31      --C61             2.03 Ang.
PLAT773_ALERT_2_G Check long C-C Bond in CIF: C33      --C61             1.74 Ang.
PLAT773_ALERT_2_G Check long C-C Bond in CIF: C34      --C70             1.79 Ang.
PLAT773_ALERT_2_G Check long C-C Bond in CIF: C38      --C67             1.87 Ang.
PLAT773_ALERT_2_G Check long C-C Bond in CIF: C39      --C62             1.74 Ang.
PLAT773_ALERT_2_G Check long C-C Bond in CIF: C40      --C65             1.91 Ang.
PLAT773_ALERT_2_G Check long C-C Bond in CIF: C41      --C45             1.82 Ang.
PLAT773_ALERT_2_G Check long C-C Bond in CIF: C41      --C66             1.84 Ang.
PLAT773_ALERT_2_G Check long C-C Bond in CIF: C46      --C73             1.83 Ang.
PLAT773_ALERT_2_G Check long C-C Bond in CIF: C56      --C75             1.74 Ang.
PLAT773_ALERT_2_G Check long C-C Bond in CIF: C57      --C77             1.85 Ang.
PLAT773_ALERT_2_G Check long C-C Bond in CIF: C61      --C80             1.77 Ang.
PLAT773_ALERT_2_G Check long C-C Bond in CIF: C61      --C78             2.01 Ang.
PLAT773_ALERT_2_G Check long C-C Bond in CIF: C63      --C78             1.71 Ang.
PLAT773_ALERT_2_G Check long C-C Bond in CIF: C63      --C79             1.88 Ang.
PLAT773_ALERT_2_G Check long C-C Bond in CIF: C64      --C73             1.70 Ang.
PLAT773_ALERT_2_G Check long C-C Bond in CIF: C64      --C71             1.91 Ang.
PLAT773_ALERT_2_G Check long C-C Bond in CIF: C65      --C71             1.94 Ang.
PLAT773_ALERT_2_G Check long C-C Bond in CIF: C66      --C74             1.86 Ang.
PLAT773_ALERT_2_G Check long C-C Bond in CIF: C67      --C74             1.82 Ang.
PLAT773_ALERT_2_G Check long C-C Bond in CIF: C67      --C73             2.03 Ang.
PLAT773_ALERT_2_G Check long C-C Bond in CIF: C69      --C73             1.98 Ang.
PLAT779_ALERT_4_G Suspect or Irrelevant (Bond) Angle(s) in CIF ...      21.30 Deg.
              C1   -C1   -C44     2_655   1_555   1_555 ......     #    4 Check
PLAT779_ALERT_4_G Suspect or Irrelevant (Bond) Angle(s) in CIF ...      15.80 Deg.
              C81  -C1   -C81     1_555   1_555   2_655 ......     #   15 Check
PLAT779_ALERT_4_G Suspect or Irrelevant (Bond) Angle(s) in CIF ...      39.00 Deg.
```

```
             C1   -C1   -C2      2_655   1_555   2_655 ......     #   22 Check
PLAT779_ALERT_4_G Suspect or Irrelevant (Bond) Angle(s) in CIF ...       42.00 Deg.
             C44  -C1   -C2      1_555   1_555   2_655 ......     #   25 Check
PLAT779_ALERT_4_G Suspect or Irrelevant (Bond) Angle(s) in CIF ...       40.00 Deg.
             C44  -C1   -C5      1_555   1_555   2_655 ......     #   32 Check
PLAT779_ALERT_4_G Suspect or Irrelevant (Bond) Angle(s) in CIF ...       20.10 Deg.
             C45  -C2   -C3      2_655   1_555   1_555 ......     #   40 Check
PLAT779_ALERT_4_G Suspect or Irrelevant (Bond) Angle(s) in CIF ...       25.30 Deg.
             C69  -C2   -C41     2_655   1_555   1_555 ......     #   45 Check
PLAT779_ALERT_4_G Suspect or Irrelevant (Bond) Angle(s) in CIF ...        9.40 Deg.
             C1   -C2   -C1      1_555   1_555   2_655 ......     #   57 Check
PLAT779_ALERT_4_G Suspect or Irrelevant (Bond) Angle(s) in CIF ...       26.30 Deg.
             C45  -C3   -C2      2_655   1_555   1_555 ......     #   68 Check
PLAT779_ALERT_4_G Suspect or Irrelevant (Bond) Angle(s) in CIF ...        9.70 Deg.
             C46  -C3   -C72     2_655   1_555   1_555 ......     #   76 Check
PLAT779_ALERT_4_G Suspect or Irrelevant (Bond) Angle(s) in CIF ...       43.50 Deg.
             C44  -C3   -C43     2_655   1_555   2_655 ......     #   88 Check
PLAT779_ALERT_4_G Suspect or Irrelevant (Bond) Angle(s) in CIF ...       14.70 Deg.
             C4   -C3   -C43     1_555   1_555   2_655 ......     #   90 Check
PLAT779_ALERT_4_G Suspect or Irrelevant (Bond) Angle(s) in CIF ...        2.00 Deg.
             C48  -C4   -C9      2_655   1_555   1_555 ......     #   97 Check
PLAT779_ALERT_4_G Suspect or Irrelevant (Bond) Angle(s) in CIF ...       27.50 Deg.
             C43  -C4   -C5      2_655   1_555   1_555 ......     #  103 Check
PLAT779_ALERT_4_G Suspect or Irrelevant (Bond) Angle(s) in CIF ...       14.10 Deg.
             C3   -C4   -C45     1_555   1_555   2_655 ......     #  118 Check
PLAT779_ALERT_4_G Suspect or Irrelevant (Bond) Angle(s) in CIF ...       15.30 Deg.
             C43  -C4   -C42     2_655   1_555   2_655 ......     #  121 Check
PLAT779_ALERT_4_G Suspect or Irrelevant (Bond) Angle(s) in CIF ...       38.40 Deg.
             C5   -C4   -C42     1_555   1_555   2_655 ......     #  126 Check
PLAT779_ALERT_4_G Suspect or Irrelevant (Bond) Angle(s) in CIF ...       23.50 Deg.
             C42  -C5   -C6      2_655   1_555   1_555 ......     #  143 Check
PLAT779_ALERT_4_G Suspect or Irrelevant (Bond) Angle(s) in CIF ...       24.20 Deg.
             C43  -C5   -C4      2_655   1_555   1_555 ......     #  144 Check
PLAT779_ALERT_4_G Suspect or Irrelevant (Bond) Angle(s) in CIF ...       11.30 Deg.
             C1   -C5   -C1      1_555   1_555   2_655 ......     #  158 Check
PLAT779_ALERT_4_G Suspect or Irrelevant (Bond) Angle(s) in CIF ...       28.20 Deg.
             C6   -C5   -C41     1_555   1_555   2_655 ......     #  162 Check
PLAT779_ALERT_4_G Suspect or Irrelevant (Bond) Angle(s) in CIF ...       20.24 Deg.
             ND3  -C5   -ND6     1_555   1_555   1_555 ......     #  182 Check
PLAT779_ALERT_4_G Suspect or Irrelevant (Bond) Angle(s) in CIF ...        5.90 Deg.
             C41  -C6   -C69     2_655   1_555   1_555 ......     #  190 Check
PLAT779_ALERT_4_G Suspect or Irrelevant (Bond) Angle(s) in CIF ...       36.80 Deg.
             C42  -C6   -C43     2_655   1_555   2_655 ......     #  193 Check
PLAT779_ALERT_4_G Suspect or Irrelevant (Bond) Angle(s) in CIF ...       28.40 Deg.
             C5   -C6   -C43     1_555   1_555   2_655 ......     #  195 Check
PLAT779_ALERT_4_G Suspect or Irrelevant (Bond) Angle(s) in CIF ...       14.80 Deg.
             C7   -C6   -C37     1_555   1_555   2_655 ......     #  201 Check
PLAT779_ALERT_4_G Suspect or Irrelevant (Bond) Angle(s) in CIF ...       40.30 Deg.
             C41  -C6   -C40     2_655   1_555   2_655 ......     #  205 Check
PLAT779_ALERT_4_G Suspect or Irrelevant (Bond) Angle(s) in CIF ...       34.70 Deg.
             C69  -C6   -C40     1_555   1_555   2_655 ......     #  208 Check
PLAT779_ALERT_4_G Suspect or Irrelevant (Bond) Angle(s) in CIF ...       21.18 Deg.
             ND6  -C6   -ND2     1_555   1_555   2_655 ......     #  227 Check
PLAT779_ALERT_4_G Suspect or Irrelevant (Bond) Angle(s) in CIF ...       20.49 Deg.
             ND6  -C6   -ND3     1_555   1_555   1_555 ......     #  236 Check
PLAT779_ALERT_4_G Suspect or Irrelevant (Bond) Angle(s) in CIF ...       39.78 Deg.
             ND2  -C6   -ND3     2_655   1_555   1_555 ......     #  237 Check
```

```
PLAT779_ALERT_4_G Suspect or Irrelevant (Bond) Angle(s) in CIF ...      18.20 Deg.
              C38  -C7   -C68     2_655   1_555   1_555 ......     #  242 Check
PLAT779_ALERT_4_G Suspect or Irrelevant (Bond) Angle(s) in CIF ...      22.80 Deg.
              C42  -C7   -C6      2_655   1_555   1_555 ......     #  246 Check
PLAT779_ALERT_4_G Suspect or Irrelevant (Bond) Angle(s) in CIF ...      15.90 Deg.
              C37  -C7   -C8      2_655   1_555   1_555 ......     #  248 Check
PLAT779_ALERT_4_G Suspect or Irrelevant (Bond) Angle(s) in CIF ...      18.10 Deg.
              C37  -C7   -C36     2_655   1_555   2_655 ......     #  253 Check
PLAT779_ALERT_4_G Suspect or Irrelevant (Bond) Angle(s) in CIF ...       4.70 Deg.
              C8   -C7   -C36     1_555   1_555   2_655 ......     #  258 Check
PLAT779_ALERT_4_G Suspect or Irrelevant (Bond) Angle(s) in CIF ...      29.60 Deg.
              C68  -C7   -C39     1_555   1_555   2_655 ......     #  262 Check
PLAT779_ALERT_4_G Suspect or Irrelevant (Bond) Angle(s) in CIF ...      23.50 Deg.
              ND6  -C7   -ND3     1_555   1_555   1_555 ......     #  282 Check
PLAT779_ALERT_4_G Suspect or Irrelevant (Bond) Angle(s) in CIF ...      20.47 Deg.
              ND6  -C7   -ND2     1_555   1_555   2_655 ......     #  291 Check
PLAT779_ALERT_4_G Suspect or Irrelevant (Bond) Angle(s) in CIF ...      43.12 Deg.
              ND3  -C7   -ND2     1_555   1_555   2_655 ......     #  292 Check
PLAT779_ALERT_4_G Suspect or Irrelevant (Bond) Angle(s) in CIF ...      36.00 Deg.
              C36  -C8   -C9      2_655   1_555   1_555 ......     #  294 Check
PLAT779_ALERT_4_G Suspect or Irrelevant (Bond) Angle(s) in CIF ...       7.70 Deg.
              C37  -C8   -C7      2_655   1_555   1_555 ......     #  300 Check
PLAT779_ALERT_4_G Suspect or Irrelevant (Bond) Angle(s) in CIF ...       9.50 Deg.
              C9   -C8   -C48     1_555   1_555   2_655 ......     #  305 Check
PLAT779_ALERT_4_G Suspect or Irrelevant (Bond) Angle(s) in CIF ...      15.40 Deg.
              C12  -C8   -C35     1_555   1_555   2_655 ......     #  311 Check
PLAT779_ALERT_4_G Suspect or Irrelevant (Bond) Angle(s) in CIF ...      24.82 Deg.
              ND3  -C8   -ND6     1_555   1_555   1_555 ......     #  328 Check
PLAT779_ALERT_4_G Suspect or Irrelevant (Bond) Angle(s) in CIF ...      10.90 Deg.
              C36  -C9   -C8      2_655   1_555   1_555 ......     #  331 Check
PLAT779_ALERT_4_G Suspect or Irrelevant (Bond) Angle(s) in CIF ...       7.00 Deg.
              C48  -C9   -C4      2_655   1_555   1_555 ......     #  332 Check
PLAT779_ALERT_4_G Suspect or Irrelevant (Bond) Angle(s) in CIF ...      41.80 Deg.
              C10  -C9   -C47     1_555   1_555   2_655 ......     #  343 Check
PLAT779_ALERT_4_G Suspect or Irrelevant (Bond) Angle(s) in CIF ...      28.00 Deg.
              C48  -C9   -C43     2_655   1_555   2_655 ......     #  344 Check
PLAT779_ALERT_4_G Suspect or Irrelevant (Bond) Angle(s) in CIF ...      25.80 Deg.
              C4   -C9   -C43     1_555   1_555   2_655 ......     #  347 Check
PLAT779_ALERT_4_G Suspect or Irrelevant (Bond) Angle(s) in CIF ...      18.02 Deg.
              ND3  -C9   -ND6     1_555   1_555   1_555 ......     #  364 Check
PLAT779_ALERT_4_G Suspect or Irrelevant (Bond) Angle(s) in CIF ...      42.60 Deg.
              C50  -C10  -C71     2_655   1_555   1_555 ......     #  366 Check
PLAT779_ALERT_4_G Suspect or Irrelevant (Bond) Angle(s) in CIF ...      16.70 Deg.
              C11  -C10  -C49     1_555   1_555   2_655 ......     #  378 Check
PLAT779_ALERT_4_G Suspect or Irrelevant (Bond) Angle(s) in CIF ...       9.80 Deg.
              C9   -C10  -C48     1_555   1_555   2_655 ......     #  384 Check
PLAT779_ALERT_4_G Suspect or Irrelevant (Bond) Angle(s) in CIF ...      29.80 Deg.
              C11  -C10  -C35     1_555   1_555   2_655 ......     #  389 Check
PLAT779_ALERT_4_G Suspect or Irrelevant (Bond) Angle(s) in CIF ...      34.10 Deg.
              C9   -C10  -C36     1_555   1_555   2_655 ......     #  397 Check
PLAT779_ALERT_4_G Suspect or Irrelevant (Bond) Angle(s) in CIF ...      43.30 Deg.
              C48  -C10  -C36     2_655   1_555   2_655 ......     #  399 Check
PLAT779_ALERT_4_G Suspect or Irrelevant (Bond) Angle(s) in CIF ...      44.00 Deg.
              C35  -C10  -C36     2_655   1_555   2_655 ......     #  400 Check
PLAT779_ALERT_4_G Suspect or Irrelevant (Bond) Angle(s) in CIF ...       4.30 Deg.
              C35  -C11  -C12     2_655   1_555   1_555 ......     #  414 Check
PLAT779_ALERT_4_G Suspect or Irrelevant (Bond) Angle(s) in CIF ...      40.00 Deg.
```

```
              C49  -C11  -C16     2_655   1_555   1_555 ......     #  416 Check
PLAT779_ALERT_4_G Suspect or Irrelevant (Bond) Angle(s) in CIF ...      36.60 Deg.
              C10  -C11  -C50     1_555   1_555   2_655 ......     #  422 Check
PLAT779_ALERT_4_G Suspect or Irrelevant (Bond) Angle(s) in CIF ...      39.10 Deg.
              C49  -C11  -C53     2_655   1_555   2_655 ......     #  425 Check
PLAT779_ALERT_4_G Suspect or Irrelevant (Bond) Angle(s) in CIF ...       0.50 Deg.
              C16  -C11  -C53     1_555   1_555   2_655 ......     #  429 Check
PLAT779_ALERT_4_G Suspect or Irrelevant (Bond) Angle(s) in CIF ...      15.00 Deg.
              C36  -C12  -C8      2_655   1_555   1_555 ......     #  443 Check
PLAT779_ALERT_4_G Suspect or Irrelevant (Bond) Angle(s) in CIF ...       9.00 Deg.
              C35  -C12  -C11     2_655   1_555   1_555 ......     #  444 Check
PLAT779_ALERT_4_G Suspect or Irrelevant (Bond) Angle(s) in CIF ...      13.60 Deg.
              C34  -C12  -C13     2_655   1_555   1_555 ......     #  449 Check
PLAT779_ALERT_4_G Suspect or Irrelevant (Bond) Angle(s) in CIF ...      13.00 Deg.
              C35  -C12  -C49     2_655   1_555   2_655 ......     #  453 Check
PLAT779_ALERT_4_G Suspect or Irrelevant (Bond) Angle(s) in CIF ...       4.00 Deg.
              C11  -C12  -C49     1_555   1_555   2_655 ......     #  457 Check
PLAT779_ALERT_4_G Suspect or Irrelevant (Bond) Angle(s) in CIF ...      21.79 Deg.
              ND3  -C12  -ND6     1_555   1_555   1_555 ......     #  473 Check
PLAT779_ALERT_4_G Suspect or Irrelevant (Bond) Angle(s) in CIF ...      44.10 Deg.
              ND3  -C12  -ND4     1_555   1_555   2_655 ......     #  481 Check
PLAT779_ALERT_4_G Suspect or Irrelevant (Bond) Angle(s) in CIF ...      41.64 Deg.
              ND6  -C12  -ND4     1_555   1_555   2_655 ......     #  482 Check
PLAT779_ALERT_4_G Suspect or Irrelevant (Bond) Angle(s) in CIF ...      10.80 Deg.
              C30  -C13  -C14     2_655   1_555   1_555 ......     #  488 Check
PLAT779_ALERT_4_G Suspect or Irrelevant (Bond) Angle(s) in CIF ...      11.70 Deg.
              C33  -C13  -C70     2_655   1_555   1_555 ......     #  490 Check
PLAT779_ALERT_4_G Suspect or Irrelevant (Bond) Angle(s) in CIF ...      39.00 Deg.
              C34  -C13  -C35     2_655   1_555   2_655 ......     #  498 Check
PLAT779_ALERT_4_G Suspect or Irrelevant (Bond) Angle(s) in CIF ...      11.80 Deg.
              C12  -C13  -C35     1_555   1_555   2_655 ......     #  503 Check
PLAT779_ALERT_4_G Suspect or Irrelevant (Bond) Angle(s) in CIF ...      38.50 Deg.
              C70  -C13  -C32     1_555   1_555   2_655 ......     #  508 Check
PLAT779_ALERT_4_G Suspect or Irrelevant (Bond) Angle(s) in CIF ...      39.70 Deg.
              C14  -C13  -C31     1_555   1_555   2_655 ......     #  514 Check
PLAT779_ALERT_4_G Suspect or Irrelevant (Bond) Angle(s) in CIF ...      44.20 Deg.
              C32  -C13  -C31     2_655   1_555   2_655 ......     #  518 Check
PLAT779_ALERT_4_G Suspect or Irrelevant (Bond) Angle(s) in CIF ...      11.68 Deg.
              ND4  -C13  -ND1     2_655   1_555   1_555 ......     #  537 Check
PLAT779_ALERT_4_G Suspect or Irrelevant (Bond) Angle(s) in CIF ...      34.80 Deg.
              C31  -C14  -C59     2_655   1_555   1_555 ......     #  551 Check
PLAT779_ALERT_4_G Suspect or Irrelevant (Bond) Angle(s) in CIF ...      15.80 Deg.
              C13  -C14  -C34     1_555   1_555   2_655 ......     #  562 Check
PLAT779_ALERT_4_G Suspect or Irrelevant (Bond) Angle(s) in CIF ...       7.80 Deg.
              C15  -C14  -C29     1_555   1_555   2_655 ......     #  568 Check
PLAT779_ALERT_4_G Suspect or Irrelevant (Bond) Angle(s) in CIF ...      12.80 Deg.
              ND4  -C14  -ND1     2_655   1_555   1_555 ......     #  584 Check
PLAT779_ALERT_4_G Suspect or Irrelevant (Bond) Angle(s) in CIF ...      19.27 Deg.
              ND4  -C14  -ND5     2_655   1_555   2_655 ......     #  592 Check
PLAT779_ALERT_4_G Suspect or Irrelevant (Bond) Angle(s) in CIF ...       6.53 Deg.
              ND1  -C14  -ND5     1_555   1_555   2_655 ......     #  593 Check
PLAT779_ALERT_4_G Suspect or Irrelevant (Bond) Angle(s) in CIF ...      11.00 Deg.
              C29  -C15  -C20     2_655   1_555   1_555 ......     #  597 Check
PLAT779_ALERT_4_G Suspect or Irrelevant (Bond) Angle(s) in CIF ...      10.80 Deg.
              C30  -C15  -C14     2_655   1_555   1_555 ......     #  602 Check
PLAT779_ALERT_4_G Suspect or Irrelevant (Bond) Angle(s) in CIF ...      14.30 Deg.
              C53  -C15  -C16     2_655   1_555   1_555 ......     #  605 Check
```

```
PLAT779_ALERT_4_G Suspect or Irrelevant (Bond) Angle(s) in CIF ...       9.00 Deg.
              C29  -C15  -C28     2_655   1_555   2_655 ......     #  609 Check
PLAT779_ALERT_4_G Suspect or Irrelevant (Bond) Angle(s) in CIF ...       5.20 Deg.
              C20  -C15  -C28     1_555   1_555   2_655 ......     #  612 Check
PLAT779_ALERT_4_G Suspect or Irrelevant (Bond) Angle(s) in CIF ...       7.33 Deg.
              ND4  -C15  -ND1     2_655   1_555   1_555 ......     #  629 Check
PLAT779_ALERT_4_G Suspect or Irrelevant (Bond) Angle(s) in CIF ...      13.70 Deg.
              C49  -C16  -C11     2_655   1_555   1_555 ......     #  634 Check
PLAT779_ALERT_4_G Suspect or Irrelevant (Bond) Angle(s) in CIF ...       7.30 Deg.
              C15  -C16  -C29     1_555   1_555   2_655 ......     #  644 Check
PLAT779_ALERT_4_G Suspect or Irrelevant (Bond) Angle(s) in CIF ...       9.50 Deg.
              C17  -C16  -C52     1_555   1_555   2_655 ......     #  647 Check
PLAT779_ALERT_4_G Suspect or Irrelevant (Bond) Angle(s) in CIF ...      18.00 Deg.
              C52  -C17  -C18     2_655   1_555   1_555 ......     #  661 Check
PLAT779_ALERT_4_G Suspect or Irrelevant (Bond) Angle(s) in CIF ...       6.20 Deg.
              C51  -C17  -C76     2_655   1_555   1_555 ......     #  666 Check
PLAT779_ALERT_4_G Suspect or Irrelevant (Bond) Angle(s) in CIF ...      14.20 Deg.
              C53  -C17  -C16     2_655   1_555   1_555 ......     #  669 Check
PLAT779_ALERT_4_G Suspect or Irrelevant (Bond) Angle(s) in CIF ...      13.00 Deg.
              C52  -C17  -C55     2_655   1_555   2_655 ......     #  673 Check
PLAT779_ALERT_4_G Suspect or Irrelevant (Bond) Angle(s) in CIF ...       4.90 Deg.
              C18  -C17  -C55     1_555   1_555   2_655 ......     #  676 Check
PLAT779_ALERT_4_G Suspect or Irrelevant (Bond) Angle(s) in CIF ...      44.90 Deg.
              C51  -C17  -C50     2_655   1_555   2_655 ......     #  681 Check
PLAT779_ALERT_4_G Suspect or Irrelevant (Bond) Angle(s) in CIF ...       4.90 Deg.
              C52  -C18  -C17     2_655   1_555   1_555 ......     #  693 Check
PLAT779_ALERT_4_G Suspect or Irrelevant (Bond) Angle(s) in CIF ...       5.90 Deg.
              C19  -C18  -C54     1_555   1_555   2_655 ......     #  698 Check
PLAT779_ALERT_4_G Suspect or Irrelevant (Bond) Angle(s) in CIF ...      15.90 Deg.
              C77  -C18  -C56     1_555   1_555   2_655 ......     #  704 Check
PLAT779_ALERT_4_G Suspect or Irrelevant (Bond) Angle(s) in CIF ...       7.10 Deg.
              C55  -C19  -C18     2_655   1_555   1_555 ......     #  716 Check
PLAT779_ALERT_4_G Suspect or Irrelevant (Bond) Angle(s) in CIF ...       7.60 Deg.
              C24  -C19  -C58     1_555   1_555   2_655 ......     #  728 Check
PLAT779_ALERT_4_G Suspect or Irrelevant (Bond) Angle(s) in CIF ...       9.50 Deg.
              C28  -C19  -C20     2_655   1_555   1_555 ......     #  732 Check
PLAT779_ALERT_4_G Suspect or Irrelevant (Bond) Angle(s) in CIF ...       1.90 Deg.
              C29  -C20  -C15     2_655   1_555   1_555 ......     #  746 Check
PLAT779_ALERT_4_G Suspect or Irrelevant (Bond) Angle(s) in CIF ...       6.30 Deg.
              C19  -C20  -C54     1_555   1_555   2_655 ......     #  756 Check
PLAT779_ALERT_4_G Suspect or Irrelevant (Bond) Angle(s) in CIF ...      40.00 Deg.
              C28  -C20  -C27     2_655   1_555   2_655 ......     #  757 Check
PLAT779_ALERT_4_G Suspect or Irrelevant (Bond) Angle(s) in CIF ...      11.40 Deg.
              C21  -C20  -C27     1_555   1_555   2_655 ......     #  759 Check
PLAT779_ALERT_4_G Suspect or Irrelevant (Bond) Angle(s) in CIF ...       9.56 Deg.
              ND4  -C20  -ND1     2_655   1_555   1_555 ......     #  777 Check
PLAT779_ALERT_4_G Suspect or Irrelevant (Bond) Angle(s) in CIF ...       9.00 Deg.
              C28  -C21  -C20     2_655   1_555   1_555 ......     #  782 Check
PLAT779_ALERT_4_G Suspect or Irrelevant (Bond) Angle(s) in CIF ...      35.60 Deg.
              C26  -C21  -C59     2_655   1_555   1_555 ......     #  786 Check
PLAT779_ALERT_4_G Suspect or Irrelevant (Bond) Angle(s) in CIF ...       8.00 Deg.
              C27  -C21  -C22     2_655   1_555   1_555 ......     #  788 Check
PLAT779_ALERT_4_G Suspect or Irrelevant (Bond) Angle(s) in CIF ...      18.00 Deg.
              C27  -C21  -C23     2_655   1_555   2_655 ......     #  793 Check
PLAT779_ALERT_4_G Suspect or Irrelevant (Bond) Angle(s) in CIF ...      11.10 Deg.
              C22  -C21  -C23     1_555   1_555   2_655 ......     #  798 Check
PLAT779_ALERT_4_G Suspect or Irrelevant (Bond) Angle(s) in CIF ...      12.94 Deg.
```

```
              ND4  -C21  -ND1     2_655   1_555   1_555 ......     #  813 Check
PLAT779_ALERT_4_G Suspect or Irrelevant (Bond) Angle(s) in CIF ...      23.33 Deg.
              ND4  -C21  -ND5     2_655   1_555   2_655 ......     #  821 Check
PLAT779_ALERT_4_G Suspect or Irrelevant (Bond) Angle(s) in CIF ...      10.67 Deg.
              ND1  -C21  -ND5     1_555   1_555   2_655 ......     #  822 Check
PLAT779_ALERT_4_G Suspect or Irrelevant (Bond) Angle(s) in CIF ...      14.00 Deg.
              C23  -C22  -C23     2_655   1_555   1_555 ......     #  826 Check
PLAT779_ALERT_4_G Suspect or Irrelevant (Bond) Angle(s) in CIF ...       6.80 Deg.
              C24  -C22  -C58     2_655   1_555   1_555 ......     #  831 Check
PLAT779_ALERT_4_G Suspect or Irrelevant (Bond) Angle(s) in CIF ...       3.00 Deg.
              C27  -C22  -C21     2_655   1_555   1_555 ......     #  834 Check
PLAT779_ALERT_4_G Suspect or Irrelevant (Bond) Angle(s) in CIF ...      11.00 Deg.
              C23  -C22  -C22     2_655   1_555   2_655 ......     #  838 Check
PLAT779_ALERT_4_G Suspect or Irrelevant (Bond) Angle(s) in CIF ...       2.50 Deg.
              C23  -C22  -C22     1_555   1_555   2_655 ......     #  841 Check
PLAT779_ALERT_4_G Suspect or Irrelevant (Bond) Angle(s) in CIF ...      40.70 Deg.
              C27  -C22  -C26     2_655   1_555   2_655 ......     #  845 Check
PLAT779_ALERT_4_G Suspect or Irrelevant (Bond) Angle(s) in CIF ...      42.30 Deg.
              C21  -C22  -C26     1_555   1_555   2_655 ......     #  849 Check
PLAT779_ALERT_4_G Suspect or Irrelevant (Bond) Angle(s) in CIF ...      12.01 Deg.
              ND1  -C22  -ND5     1_555   1_555   2_655 ......     #  867 Check
PLAT779_ALERT_4_G Suspect or Irrelevant (Bond) Angle(s) in CIF ...      10.88 Deg.
              ND1  -C22  -ND4     1_555   1_555   2_655 ......     #  876 Check
PLAT779_ALERT_4_G Suspect or Irrelevant (Bond) Angle(s) in CIF ...      22.49 Deg.
              ND5  -C22  -ND4     2_655   1_555   2_655 ......     #  877 Check
PLAT779_ALERT_4_G Suspect or Irrelevant (Bond) Angle(s) in CIF ...       4.00 Deg.
              C23  -C23  -C22     2_655   1_555   1_555 ......     #  882 Check
PLAT779_ALERT_4_G Suspect or Irrelevant (Bond) Angle(s) in CIF ...       6.00 Deg.
              C27  -C23  -C21     1_555   1_555   2_655 ......     #  890 Check
PLAT779_ALERT_4_G Suspect or Irrelevant (Bond) Angle(s) in CIF ...       5.40 Deg.
              C24  -C23  -C58     1_555   1_555   2_655 ......     #  897 Check
PLAT779_ALERT_4_G Suspect or Irrelevant (Bond) Angle(s) in CIF ...      11.57 Deg.
              ND1  -C23  -ND5     2_655   1_555   1_555 ......     #  913 Check
PLAT779_ALERT_4_G Suspect or Irrelevant (Bond) Angle(s) in CIF ...      10.78 Deg.
              ND1  -C23  -ND4     2_655   1_555   1_555 ......     #  921 Check
PLAT779_ALERT_4_G Suspect or Irrelevant (Bond) Angle(s) in CIF ...      21.93 Deg.
              ND5  -C23  -ND4     1_555   1_555   1_555 ......     #  922 Check
PLAT779_ALERT_4_G Suspect or Irrelevant (Bond) Angle(s) in CIF ...      23.00 Deg.
              C58  -C24  -C25     2_655   1_555   1_555 ......     #  924 Check
PLAT779_ALERT_4_G Suspect or Irrelevant (Bond) Angle(s) in CIF ...       6.60 Deg.
              C54  -C24  -C19     2_655   1_555   1_555 ......     #  932 Check
PLAT779_ALERT_4_G Suspect or Irrelevant (Bond) Angle(s) in CIF ...      10.70 Deg.
              C22  -C24  -C23     2_655   1_555   1_555 ......     #  934 Check
PLAT779_ALERT_4_G Suspect or Irrelevant (Bond) Angle(s) in CIF ...       5.00 Deg.
              C58  -C24  -C57     2_655   1_555   2_655 ......     #  938 Check
PLAT779_ALERT_4_G Suspect or Irrelevant (Bond) Angle(s) in CIF ...      23.30 Deg.
              C25  -C24  -C57     1_555   1_555   2_655 ......     #  940 Check
PLAT779_ALERT_4_G Suspect or Irrelevant (Bond) Angle(s) in CIF ...      11.51 Deg.
              ND5  -C24  -ND1     1_555   1_555   2_655 ......     #  958 Check
PLAT779_ALERT_4_G Suspect or Irrelevant (Bond) Angle(s) in CIF ...       3.80 Deg.
              C58  -C25  -C24     2_655   1_555   1_555 ......     #  961 Check
PLAT779_ALERT_4_G Suspect or Irrelevant (Bond) Angle(s) in CIF ...      19.70 Deg.
              C57  -C25  -C78     2_655   1_555   1_555 ......     #  969 Check
PLAT779_ALERT_4_G Suspect or Irrelevant (Bond) Angle(s) in CIF ...      13.34 Deg.
              ND5  -C25  -ND1     1_555   1_555   2_655 ......     #  986 Check
PLAT779_ALERT_4_G Suspect or Irrelevant (Bond) Angle(s) in CIF ...      20.98 Deg.
              ND5  -C25  -ND4     1_555   1_555   1_555 ......     #  993 Check
```

```
PLAT779_ALERT_4_G Suspect or Irrelevant (Bond) Angle(s) in CIF ...       7.78 Deg.
              ND1  -C25  -ND4     2_655   1_555   1_555 ......     #  994 Check
PLAT779_ALERT_4_G Suspect or Irrelevant (Bond) Angle(s) in CIF ...      18.00 Deg.
              C21  -C26  -C27     2_655   1_555   1_555 ......     #  997 Check
PLAT779_ALERT_4_G Suspect or Irrelevant (Bond) Angle(s) in CIF ...      26.60 Deg.
              C59  -C26  -C31     2_655   1_555   1_555 ......     # 1005 Check
PLAT779_ALERT_4_G Suspect or Irrelevant (Bond) Angle(s) in CIF ...      13.80 Deg.
              C25  -C26  -C57     1_555   1_555   2_655 ......     # 1014 Check
PLAT779_ALERT_4_G Suspect or Irrelevant (Bond) Angle(s) in CIF ...      30.10 Deg.
              C27  -C26  -C22     1_555   1_555   2_655 ......     # 1018 Check
PLAT779_ALERT_4_G Suspect or Irrelevant (Bond) Angle(s) in CIF ...      13.30 Deg.
              ND1  -C26  -ND5     2_655   1_555   1_555 ......     # 1039 Check
PLAT779_ALERT_4_G Suspect or Irrelevant (Bond) Angle(s) in CIF ...      12.45 Deg.
              ND1  -C26  -ND4     2_655   1_555   1_555 ......     # 1048 Check
PLAT779_ALERT_4_G Suspect or Irrelevant (Bond) Angle(s) in CIF ...      25.68 Deg.
              ND5  -C26  -ND4     1_555   1_555   1_555 ......     # 1049 Check
PLAT779_ALERT_4_G Suspect or Irrelevant (Bond) Angle(s) in CIF ...      14.60 Deg.
              C22  -C27  -C23     2_655   1_555   1_555 ......     # 1052 Check
PLAT779_ALERT_4_G Suspect or Irrelevant (Bond) Angle(s) in CIF ...      30.40 Deg.
              C26  -C27  -C59     1_555   1_555   2_655 ......     # 1063 Check
PLAT779_ALERT_4_G Suspect or Irrelevant (Bond) Angle(s) in CIF ...      41.60 Deg.
              C21  -C27  -C20     2_655   1_555   2_655 ......     # 1065 Check
PLAT779_ALERT_4_G Suspect or Irrelevant (Bond) Angle(s) in CIF ...       6.20 Deg.
              C28  -C27  -C20     1_555   1_555   2_655 ......     # 1069 Check
PLAT779_ALERT_4_G Suspect or Irrelevant (Bond) Angle(s) in CIF ...      12.66 Deg.
              ND1  -C27  -ND4     2_655   1_555   1_555 ......     # 1085 Check
PLAT779_ALERT_4_G Suspect or Irrelevant (Bond) Angle(s) in CIF ...      11.60 Deg.
              ND1  -C27  -ND5     2_655   1_555   1_555 ......     # 1093 Check
PLAT779_ALERT_4_G Suspect or Irrelevant (Bond) Angle(s) in CIF ...      23.95 Deg.
              ND4  -C27  -ND5     1_555   1_555   1_555 ......     # 1094 Check
PLAT779_ALERT_4_G Suspect or Irrelevant (Bond) Angle(s) in CIF ...      31.00 Deg.
              C20  -C28  -C29     2_655   1_555   1_555 ......     # 1096 Check
PLAT779_ALERT_4_G Suspect or Irrelevant (Bond) Angle(s) in CIF ...       6.70 Deg.
              C19  -C28  -C54     2_655   1_555   1_555 ......     # 1104 Check
PLAT779_ALERT_4_G Suspect or Irrelevant (Bond) Angle(s) in CIF ...      14.20 Deg.
              C21  -C28  -C27     2_655   1_555   1_555 ......     # 1106 Check
PLAT779_ALERT_4_G Suspect or Irrelevant (Bond) Angle(s) in CIF ...      30.00 Deg.
              C20  -C28  -C15     2_655   1_555   2_655 ......     # 1110 Check
PLAT779_ALERT_4_G Suspect or Irrelevant (Bond) Angle(s) in CIF ...       1.30 Deg.
              C29  -C28  -C15     1_555   1_555   2_655 ......     # 1112 Check
PLAT779_ALERT_4_G Suspect or Irrelevant (Bond) Angle(s) in CIF ...      10.22 Deg.
              ND4  -C28  -ND1     1_555   1_555   2_655 ......     # 1130 Check
PLAT779_ALERT_4_G Suspect or Irrelevant (Bond) Angle(s) in CIF ...       6.10 Deg.
              C20  -C29  -C28     2_655   1_555   1_555 ......     # 1135 Check
PLAT779_ALERT_4_G Suspect or Irrelevant (Bond) Angle(s) in CIF ...      10.20 Deg.
              C14  -C29  -C30     2_655   1_555   1_555 ......     # 1145 Check
PLAT779_ALERT_4_G Suspect or Irrelevant (Bond) Angle(s) in CIF ...      13.00 Deg.
              C53  -C29  -C16     1_555   1_555   2_655 ......     # 1148 Check
PLAT779_ALERT_4_G Suspect or Irrelevant (Bond) Angle(s) in CIF ...       7.71 Deg.
              ND4  -C29  -ND1     1_555   1_555   2_655 ......     # 1166 Check
PLAT779_ALERT_4_G Suspect or Irrelevant (Bond) Angle(s) in CIF ...      16.50 Deg.
              C34  -C30  -C13     1_555   1_555   2_655 ......     # 1169 Check
PLAT779_ALERT_4_G Suspect or Irrelevant (Bond) Angle(s) in CIF ...       7.30 Deg.
              C15  -C30  -C29     2_655   1_555   1_555 ......     # 1181 Check
PLAT779_ALERT_4_G Suspect or Irrelevant (Bond) Angle(s) in CIF ...      36.00 Deg.
              C14  -C30  -C59     2_655   1_555   2_655 ......     # 1182 Check
PLAT779_ALERT_4_G Suspect or Irrelevant (Bond) Angle(s) in CIF ...      30.10 Deg.
```

```
              C31  -C30  -C59     1_555   1_555   2_655 ......     # 1185 Check
PLAT779_ALERT_4_G Suspect or Irrelevant (Bond) Angle(s) in CIF ...      27.00 Deg.
              C14  -C30  -ND4     2_655   1_555   1_555 ......     # 1188 Check
PLAT779_ALERT_4_G Suspect or Irrelevant (Bond) Angle(s) in CIF ...      19.00 Deg.
              C14  -C30  -ND1     2_655   1_555   2_655 ......     # 1195 Check
PLAT779_ALERT_4_G Suspect or Irrelevant (Bond) Angle(s) in CIF ...      11.32 Deg.
              ND4  -C30  -ND1     1_555   1_555   2_655 ......     # 1202 Check
PLAT779_ALERT_4_G Suspect or Irrelevant (Bond) Angle(s) in CIF ...       9.90 Deg.
              C14  -C31  -C30     2_655   1_555   1_555 ......     # 1205 Check
PLAT779_ALERT_4_G Suspect or Irrelevant (Bond) Angle(s) in CIF ...      28.30 Deg.
              C59  -C31  -C26     2_655   1_555   1_555 ......     # 1213 Check
PLAT779_ALERT_4_G Suspect or Irrelevant (Bond) Angle(s) in CIF ...      44.40 Deg.
              C60  -C31  -C61     2_655   1_555   2_655 ......     # 1227 Check
PLAT779_ALERT_4_G Suspect or Irrelevant (Bond) Angle(s) in CIF ...      16.00 Deg.
              C32  -C31  -C61     1_555   1_555   2_655 ......     # 1228 Check
PLAT779_ALERT_4_G Suspect or Irrelevant (Bond) Angle(s) in CIF ...      13.38 Deg.
              ND1  -C31  -ND4     2_655   1_555   1_555 ......     # 1247 Check
PLAT779_ALERT_4_G Suspect or Irrelevant (Bond) Angle(s) in CIF ...      11.31 Deg.
              ND1  -C31  -ND5     2_655   1_555   1_555 ......     # 1256 Check
PLAT779_ALERT_4_G Suspect or Irrelevant (Bond) Angle(s) in CIF ...      24.67 Deg.
              ND4  -C31  -ND5     1_555   1_555   1_555 ......     # 1257 Check
PLAT779_ALERT_4_G Suspect or Irrelevant (Bond) Angle(s) in CIF ...      16.90 Deg.
              C70  -C32  -C33     2_655   1_555   1_555 ......     # 1260 Check
PLAT779_ALERT_4_G Suspect or Irrelevant (Bond) Angle(s) in CIF ...      18.00 Deg.
              C61  -C32  -C79     2_655   1_555   1_555 ......     # 1264 Check
PLAT779_ALERT_4_G Suspect or Irrelevant (Bond) Angle(s) in CIF ...      34.30 Deg.
              C33  -C32  -C13     1_555   1_555   2_655 ......     # 1275 Check
PLAT779_ALERT_4_G Suspect or Irrelevant (Bond) Angle(s) in CIF ...      12.99 Deg.
              ND1  -C32  -ND5     2_655   1_555   1_555 ......     # 1293 Check
PLAT779_ALERT_4_G Suspect or Irrelevant (Bond) Angle(s) in CIF ...      11.81 Deg.
              ND1  -C32  -ND4     2_655   1_555   1_555 ......     # 1301 Check
PLAT779_ALERT_4_G Suspect or Irrelevant (Bond) Angle(s) in CIF ...      24.57 Deg.
              ND5  -C32  -ND4     1_555   1_555   1_555 ......     # 1302 Check
PLAT779_ALERT_4_G Suspect or Irrelevant (Bond) Angle(s) in CIF ...      10.90 Deg.
              C13  -C33  -C34     2_655   1_555   1_555 ......     # 1305 Check
PLAT779_ALERT_4_G Suspect or Irrelevant (Bond) Angle(s) in CIF ...      14.20 Deg.
              C38  -C33  -C68     1_555   1_555   2_655 ......     # 1317 Check
PLAT779_ALERT_4_G Suspect or Irrelevant (Bond) Angle(s) in CIF ...      27.20 Deg.
              C70  -C33  -C61     2_655   1_555   2_655 ......     # 1318 Check
PLAT779_ALERT_4_G Suspect or Irrelevant (Bond) Angle(s) in CIF ...      24.70 Deg.
              C32  -C33  -C61     1_555   1_555   2_655 ......     # 1321 Check
PLAT779_ALERT_4_G Suspect or Irrelevant (Bond) Angle(s) in CIF ...      11.44 Deg.
              ND4  -C33  -ND1     1_555   1_555   2_655 ......     # 1347 Check
PLAT779_ALERT_4_G Suspect or Irrelevant (Bond) Angle(s) in CIF ...      19.97 Deg.
              ND6  -C33  -ND3     2_655   1_555   2_655 ......     # 1355 Check
PLAT779_ALERT_4_G Suspect or Irrelevant (Bond) Angle(s) in CIF ...      43.18 Deg.
              ND4  -C33  -ND3     1_555   1_555   2_655 ......     # 1356 Check
PLAT779_ALERT_4_G Suspect or Irrelevant (Bond) Angle(s) in CIF ...      21.42 Deg.
              ND4  -C33  -ND5     1_555   1_555   1_555 ......     # 1366 Check
PLAT779_ALERT_4_G Suspect or Irrelevant (Bond) Angle(s) in CIF ...      10.39 Deg.
              ND1  -C33  -ND5     2_655   1_555   1_555 ......     # 1367 Check
PLAT779_ALERT_4_G Suspect or Irrelevant (Bond) Angle(s) in CIF ...      30.00 Deg.
              C13  -C34  -C33     2_655   1_555   1_555 ......     # 1372 Check
PLAT779_ALERT_4_G Suspect or Irrelevant (Bond) Angle(s) in CIF ...      15.70 Deg.
              C12  -C34  -C35     2_655   1_555   1_555 ......     # 1376 Check
PLAT779_ALERT_4_G Suspect or Irrelevant (Bond) Angle(s) in CIF ...      10.20 Deg.
              C30  -C34  -C14     1_555   1_555   2_655 ......     # 1381 Check
```

```
PLAT779_ALERT_4_G Suspect or Irrelevant (Bond) Angle(s) in CIF ...      23.20 Deg.
             C13  -C34  -C70     2_655   1_555   2_655 ......     # 1384 Check
PLAT779_ALERT_4_G Suspect or Irrelevant (Bond) Angle(s) in CIF ...       7.20 Deg.
             C33  -C34  -C70     1_555   1_555   2_655 ......     # 1387 Check
PLAT779_ALERT_4_G Suspect or Irrelevant (Bond) Angle(s) in CIF ...      10.06 Deg.
             ND4  -C34  -ND1     1_555   1_555   2_655 ......     # 1412 Check
PLAT779_ALERT_4_G Suspect or Irrelevant (Bond) Angle(s) in CIF ...      43.58 Deg.
             ND4  -C34  -ND6     1_555   1_555   2_655 ......     # 1421 Check
PLAT779_ALERT_4_G Suspect or Irrelevant (Bond) Angle(s) in CIF ...      20.65 Deg.
             ND3  -C34  -ND6     2_655   1_555   2_655 ......     # 1422 Check
PLAT779_ALERT_4_G Suspect or Irrelevant (Bond) Angle(s) in CIF ...      43.18 Deg.
             ND1  -C34  -ND6     2_655   1_555   2_655 ......     # 1423 Check
PLAT779_ALERT_4_G Suspect or Irrelevant (Bond) Angle(s) in CIF ...       4.20 Deg.
             C11  -C35  -C49     2_655   1_555   1_555 ......     # 1426 Check
PLAT779_ALERT_4_G Suspect or Irrelevant (Bond) Angle(s) in CIF ...      12.60 Deg.
             C36  -C35  -C8      1_555   1_555   2_655 ......     # 1437 Check
PLAT779_ALERT_4_G Suspect or Irrelevant (Bond) Angle(s) in CIF ...      42.10 Deg.
             C12  -C35  -C13     2_655   1_555   2_655 ......     # 1439 Check
PLAT779_ALERT_4_G Suspect or Irrelevant (Bond) Angle(s) in CIF ...       9.90 Deg.
             C34  -C35  -C13     1_555   1_555   2_655 ......     # 1443 Check
PLAT779_ALERT_4_G Suspect or Irrelevant (Bond) Angle(s) in CIF ...      19.43 Deg.
             ND3  -C35  -ND6     2_655   1_555   2_655 ......     # 1468 Check
PLAT779_ALERT_4_G Suspect or Irrelevant (Bond) Angle(s) in CIF ...       9.50 Deg.
             C9   -C36  -C48     2_655   1_555   1_555 ......     # 1471 Check
PLAT779_ALERT_4_G Suspect or Irrelevant (Bond) Angle(s) in CIF ...      17.60 Deg.
             C8   -C36  -C37     2_655   1_555   1_555 ......     # 1475 Check
PLAT779_ALERT_4_G Suspect or Irrelevant (Bond) Angle(s) in CIF ...      17.80 Deg.
             C12  -C36  -C35     2_655   1_555   1_555 ......     # 1482 Check
PLAT779_ALERT_4_G Suspect or Irrelevant (Bond) Angle(s) in CIF ...      21.00 Deg.
             C8   -C36  -C7      2_655   1_555   2_655 ......     # 1484 Check
PLAT779_ALERT_4_G Suspect or Irrelevant (Bond) Angle(s) in CIF ...       6.60 Deg.
             C37  -C36  -C7      1_555   1_555   2_655 ......     # 1488 Check
PLAT779_ALERT_4_G Suspect or Irrelevant (Bond) Angle(s) in CIF ...      22.93 Deg.
             ND3  -C36  -ND6     2_655   1_555   2_655 ......     # 1513 Check
PLAT779_ALERT_4_G Suspect or Irrelevant (Bond) Angle(s) in CIF ...       5.90 Deg.
             C8   -C37  -C36     2_655   1_555   1_555 ......     # 1518 Check
PLAT779_ALERT_4_G Suspect or Irrelevant (Bond) Angle(s) in CIF ...      12.70 Deg.
             C38  -C37  -C68     1_555   1_555   2_655 ......     # 1526 Check
PLAT779_ALERT_4_G Suspect or Irrelevant (Bond) Angle(s) in CIF ...      16.70 Deg.
             C42  -C37  -C6      1_555   1_555   2_655 ......     # 1533 Check
PLAT779_ALERT_4_G Suspect or Irrelevant (Bond) Angle(s) in CIF ...      24.64 Deg.
             ND6  -C37  -ND3     2_655   1_555   2_655 ......     # 1549 Check
PLAT779_ALERT_4_G Suspect or Irrelevant (Bond) Angle(s) in CIF ...      16.56 Deg.
             ND6  -C37  -ND2     2_655   1_555   1_555 ......     # 1557 Check
PLAT779_ALERT_4_G Suspect or Irrelevant (Bond) Angle(s) in CIF ...      40.48 Deg.
             ND3  -C37  -ND2     2_655   1_555   1_555 ......     # 1558 Check
PLAT779_ALERT_4_G Suspect or Irrelevant (Bond) Angle(s) in CIF ...      21.90 Deg.
             C7   -C38  -C37     2_655   1_555   1_555 ......     # 1563 Check
PLAT779_ALERT_4_G Suspect or Irrelevant (Bond) Angle(s) in CIF ...      18.20 Deg.
             C70  -C38  -C33     2_655   1_555   1_555 ......     # 1567 Check
PLAT779_ALERT_4_G Suspect or Irrelevant (Bond) Angle(s) in CIF ...      10.00 Deg.
             C68  -C38  -C39     2_655   1_555   1_555 ......     # 1569 Check
PLAT779_ALERT_4_G Suspect or Irrelevant (Bond) Angle(s) in CIF ...      10.00 Deg.
             C68  -C38  -C67     2_655   1_555   2_655 ......     # 1574 Check
PLAT779_ALERT_4_G Suspect or Irrelevant (Bond) Angle(s) in CIF ...       8.00 Deg.
             C39  -C38  -C67     1_555   1_555   2_655 ......     # 1579 Check
PLAT779_ALERT_4_G Suspect or Irrelevant (Bond) Angle(s) in CIF ...      21.56 Deg.
```

```
              ND6  -C38  -ND2     2_655   1_555   1_555 ......     # 1594 Check
PLAT779_ALERT_4_G Suspect or Irrelevant (Bond) Angle(s) in CIF ...      19.74 Deg.
              ND6  -C38  -ND3     2_655   1_555   2_655 ......     # 1602 Check
PLAT779_ALERT_4_G Suspect or Irrelevant (Bond) Angle(s) in CIF ...      41.27 Deg.
              ND2  -C38  -ND3     1_555   1_555   2_655 ......     # 1603 Check
PLAT779_ALERT_4_G Suspect or Irrelevant (Bond) Angle(s) in CIF ...      26.50 Deg.
              C67  -C39  -C80     2_655   1_555   1_555 ......     # 1605 Check
PLAT779_ALERT_4_G Suspect or Irrelevant (Bond) Angle(s) in CIF ...       4.20 Deg.
              C68  -C39  -C38     2_655   1_555   1_555 ......     # 1611 Check
PLAT779_ALERT_4_G Suspect or Irrelevant (Bond) Angle(s) in CIF ...      18.10 Deg.
              C40  -C39  -C66     1_555   1_555   2_655 ......     # 1617 Check
PLAT779_ALERT_4_G Suspect or Irrelevant (Bond) Angle(s) in CIF ...      36.60 Deg.
              C67  -C39  -C62     2_655   1_555   2_655 ......     # 1619 Check
PLAT779_ALERT_4_G Suspect or Irrelevant (Bond) Angle(s) in CIF ...      10.10 Deg.
              C80  -C39  -C62     1_555   1_555   2_655 ......     # 1621 Check
PLAT779_ALERT_4_G Suspect or Irrelevant (Bond) Angle(s) in CIF ...      40.20 Deg.
              C68  -C39  -C7      2_655   1_555   2_655 ......     # 1626 Check
PLAT779_ALERT_4_G Suspect or Irrelevant (Bond) Angle(s) in CIF ...      37.30 Deg.
              C38  -C39  -C7      1_555   1_555   2_655 ......     # 1629 Check
PLAT779_ALERT_4_G Suspect or Irrelevant (Bond) Angle(s) in CIF ...      23.36 Deg.
              ND2  -C39  -ND6     1_555   1_555   2_655 ......     # 1648 Check
PLAT779_ALERT_4_G Suspect or Irrelevant (Bond) Angle(s) in CIF ...      14.30 Deg.
              C66  -C40  -C73     2_655   1_555   1_555 ......     # 1650 Check
PLAT779_ALERT_4_G Suspect or Irrelevant (Bond) Angle(s) in CIF ...      24.30 Deg.
              C69  -C40  -C41     2_655   1_555   1_555 ......     # 1656 Check
PLAT779_ALERT_4_G Suspect or Irrelevant (Bond) Angle(s) in CIF ...      17.60 Deg.
              C39  -C40  -C67     1_555   1_555   2_655 ......     # 1662 Check
PLAT779_ALERT_4_G Suspect or Irrelevant (Bond) Angle(s) in CIF ...      21.90 Deg.
              C66  -C40  -C65     2_655   1_555   2_655 ......     # 1664 Check
PLAT779_ALERT_4_G Suspect or Irrelevant (Bond) Angle(s) in CIF ...       8.10 Deg.
              C73  -C40  -C65     1_555   1_555   2_655 ......     # 1666 Check
PLAT779_ALERT_4_G Suspect or Irrelevant (Bond) Angle(s) in CIF ...      24.70 Deg.
              C41  -C40  -C6      1_555   1_555   2_655 ......     # 1674 Check
PLAT779_ALERT_4_G Suspect or Irrelevant (Bond) Angle(s) in CIF ...      20.43 Deg.
              ND2  -C40  -ND6     1_555   1_555   2_655 ......     # 1693 Check
PLAT779_ALERT_4_G Suspect or Irrelevant (Bond) Angle(s) in CIF ...       3.90 Deg.
              C6   -C41  -C42     2_655   1_555   1_555 ......     # 1701 Check
PLAT779_ALERT_4_G Suspect or Irrelevant (Bond) Angle(s) in CIF ...      44.00 Deg.
              C69  -C41  -C45     2_655   1_555   2_655 ......     # 1704 Check
PLAT779_ALERT_4_G Suspect or Irrelevant (Bond) Angle(s) in CIF ...      27.50 Deg.
              C2   -C41  -C45     1_555   1_555   2_655 ......     # 1706 Check
PLAT779_ALERT_4_G Suspect or Irrelevant (Bond) Angle(s) in CIF ...      39.10 Deg.
              C69  -C41  -C66     2_655   1_555   2_655 ......     # 1709 Check
PLAT779_ALERT_4_G Suspect or Irrelevant (Bond) Angle(s) in CIF ...      12.20 Deg.
              C40  -C41  -C66     1_555   1_555   2_655 ......     # 1712 Check
PLAT779_ALERT_4_G Suspect or Irrelevant (Bond) Angle(s) in CIF ...      41.90 Deg.
              C42  -C41  -C5      1_555   1_555   2_655 ......     # 1719 Check
PLAT779_ALERT_4_G Suspect or Irrelevant (Bond) Angle(s) in CIF ...      21.20 Deg.
              ND2  -C41  -ND6     1_555   1_555   2_655 ......     # 1738 Check
PLAT779_ALERT_4_G Suspect or Irrelevant (Bond) Angle(s) in CIF ...      37.30 Deg.
              C5   -C42  -C43     2_655   1_555   1_555 ......     # 1743 Check
PLAT779_ALERT_4_G Suspect or Irrelevant (Bond) Angle(s) in CIF ...      20.90 Deg.
              C7   -C42  -C37     2_655   1_555   1_555 ......     # 1747 Check
PLAT779_ALERT_4_G Suspect or Irrelevant (Bond) Angle(s) in CIF ...       6.40 Deg.
              C6   -C42  -C41     2_655   1_555   1_555 ......     # 1749 Check
PLAT779_ALERT_4_G Suspect or Irrelevant (Bond) Angle(s) in CIF ...      43.80 Deg.
              C5   -C42  -C4      2_655   1_555   2_655 ......     # 1755 Check
```

```
PLAT779_ALERT_4_G Suspect or Irrelevant (Bond) Angle(s) in CIF ...       8.40 Deg.
              C43  -C42  -C4      1_555   1_555   2_655 ......     # 1757 Check
PLAT779_ALERT_4_G Suspect or Irrelevant (Bond) Angle(s) in CIF ...      22.22 Deg.
              ND6  -C42  -ND3     2_655   1_555   2_655 ......     # 1774 Check
PLAT779_ALERT_4_G Suspect or Irrelevant (Bond) Angle(s) in CIF ...      19.07 Deg.
              ND6  -C42  -ND2     2_655   1_555   1_555 ......     # 1782 Check
PLAT779_ALERT_4_G Suspect or Irrelevant (Bond) Angle(s) in CIF ...      39.48 Deg.
              ND3  -C42  -ND2     2_655   1_555   1_555 ......     # 1783 Check
PLAT779_ALERT_4_G Suspect or Irrelevant (Bond) Angle(s) in CIF ...       6.10 Deg.
              C48  -C43  -C9      1_555   1_555   2_655 ......     # 1798 Check
PLAT779_ALERT_4_G Suspect or Irrelevant (Bond) Angle(s) in CIF ...      14.70 Deg.
              C42  -C43  -C6      1_555   1_555   2_655 ......     # 1801 Check
PLAT779_ALERT_4_G Suspect or Irrelevant (Bond) Angle(s) in CIF ...      29.00 Deg.
              C4   -C43  -C3      2_655   1_555   2_655 ......     # 1805 Check
PLAT779_ALERT_4_G Suspect or Irrelevant (Bond) Angle(s) in CIF ...      43.30 Deg.
              C44  -C43  -C3      1_555   1_555   2_655 ......     # 1808 Check
PLAT779_ALERT_4_G Suspect or Irrelevant (Bond) Angle(s) in CIF ...      20.91 Deg.
              ND3  -C43  -ND6     2_655   1_555   2_655 ......     # 1828 Check
PLAT779_ALERT_4_G Suspect or Irrelevant (Bond) Angle(s) in CIF ...      36.90 Deg.
              C5   -C44  -C43     2_655   1_555   1_555 ......     # 1839 Check
PLAT779_ALERT_4_G Suspect or Irrelevant (Bond) Angle(s) in CIF ...      31.30 Deg.
              C4   -C44  -C43     2_655   1_555   1_555 ......     # 1842 Check
PLAT779_ALERT_4_G Suspect or Irrelevant (Bond) Angle(s) in CIF ...      37.40 Deg.
              C2   -C44  -C45     2_655   1_555   1_555 ......     # 1846 Check
PLAT779_ALERT_4_G Suspect or Irrelevant (Bond) Angle(s) in CIF ...      27.50 Deg.
              C3   -C44  -C45     2_655   1_555   1_555 ......     # 1848 Check
PLAT779_ALERT_4_G Suspect or Irrelevant (Bond) Angle(s) in CIF ...       6.90 Deg.
              C1   -C44  -C1      2_655   1_555   1_555 ......     # 1851 Check
PLAT779_ALERT_4_G Suspect or Irrelevant (Bond) Angle(s) in CIF ...      12.60 Deg.
              C46  -C45  -C72     1_555   1_555   2_655 ......     # 1870 Check
PLAT779_ALERT_4_G Suspect or Irrelevant (Bond) Angle(s) in CIF ...      17.30 Deg.
              C69  -C45  -C41     1_555   1_555   2_655 ......     # 1876 Check
PLAT779_ALERT_4_G Suspect or Irrelevant (Bond) Angle(s) in CIF ...      31.20 Deg.
              C3   -C45  -C4      2_655   1_555   2_655 ......     # 1878 Check
PLAT779_ALERT_4_G Suspect or Irrelevant (Bond) Angle(s) in CIF ...      44.40 Deg.
              C44  -C45  -C4      1_555   1_555   2_655 ......     # 1880 Check
PLAT779_ALERT_4_G Suspect or Irrelevant (Bond) Angle(s) in CIF ...      26.40 Deg.
              C3   -C46  -C45     2_655   1_555   1_555 ......     # 1897 Check
PLAT779_ALERT_4_G Suspect or Irrelevant (Bond) Angle(s) in CIF ...      15.10 Deg.
              C72  -C46  -C65     2_655   1_555   1_555 ......     # 1899 Check
PLAT779_ALERT_4_G Suspect or Irrelevant (Bond) Angle(s) in CIF ...      44.40 Deg.
              C47  -C46  -C71     1_555   1_555   2_655 ......     # 1905 Check
PLAT779_ALERT_4_G Suspect or Irrelevant (Bond) Angle(s) in CIF ...      30.30 Deg.
              C72  -C46  -C73     2_655   1_555   2_655 ......     # 1908 Check
PLAT779_ALERT_4_G Suspect or Irrelevant (Bond) Angle(s) in CIF ...      15.40 Deg.
              C65  -C46  -C73     1_555   1_555   2_655 ......     # 1912 Check
PLAT779_ALERT_4_G Suspect or Irrelevant (Bond) Angle(s) in CIF ...      40.90 Deg.
              C10  -C47  -C50     2_655   1_555   1_555 ......     # 1927 Check
PLAT779_ALERT_4_G Suspect or Irrelevant (Bond) Angle(s) in CIF ...      41.10 Deg.
              C71  -C47  -C50     2_655   1_555   1_555 ......     # 1928 Check
PLAT779_ALERT_4_G Suspect or Irrelevant (Bond) Angle(s) in CIF ...      12.30 Deg.
              C48  -C47  -C9      1_555   1_555   2_655 ......     # 1934 Check
PLAT779_ALERT_4_G Suspect or Irrelevant (Bond) Angle(s) in CIF ...      14.80 Deg.
              C46  -C47  -C72     1_555   1_555   2_655 ......     # 1938 Check
PLAT779_ALERT_4_G Suspect or Irrelevant (Bond) Angle(s) in CIF ...      37.00 Deg.
              C48  -C47  -C4      1_555   1_555   2_655 ......     # 1945 Check
PLAT779_ALERT_4_G Suspect or Irrelevant (Bond) Angle(s) in CIF ...      35.40 Deg.
```

```
           C46  -C47  -C3      1_555   1_555   2_655 ......     # 1951 Check
PLAT779_ALERT_4_G Suspect or Irrelevant (Bond) Angle(s) in CIF ...      36.00 Deg.
           C9   -C48  -C36     2_655   1_555   1_555 ......     # 1967 Check
PLAT779_ALERT_4_G Suspect or Irrelevant (Bond) Angle(s) in CIF ...      31.20 Deg.
           C4   -C48  -C43     2_655   1_555   1_555 ......     # 1970 Check
PLAT779_ALERT_4_G Suspect or Irrelevant (Bond) Angle(s) in CIF ...      10.90 Deg.
           C36  -C48  -C8      1_555   1_555   2_655 ......     # 1978 Check
PLAT779_ALERT_4_G Suspect or Irrelevant (Bond) Angle(s) in CIF ...      39.20 Deg.
           C47  -C48  -C10     1_555   1_555   2_655 ......     # 1985 Check
PLAT779_ALERT_4_G Suspect or Irrelevant (Bond) Angle(s) in CIF ...      18.13 Deg.
           ND3  -C48  -ND6     2_655   1_555   2_655 ......     # 2001 Check
PLAT779_ALERT_4_G Suspect or Irrelevant (Bond) Angle(s) in CIF ...      10.00 Deg.
           C11  -C49  -C35     2_655   1_555   1_555 ......     # 2005 Check
PLAT779_ALERT_4_G Suspect or Irrelevant (Bond) Angle(s) in CIF ...      39.50 Deg.
           C50  -C49  -C10     1_555   1_555   2_655 ......     # 2010 Check
PLAT779_ALERT_4_G Suspect or Irrelevant (Bond) Angle(s) in CIF ...       3.80 Deg.
           C16  -C49  -C53     2_655   1_555   1_555 ......     # 2013 Check
PLAT779_ALERT_4_G Suspect or Irrelevant (Bond) Angle(s) in CIF ...      13.70 Deg.
           C11  -C49  -C12     2_655   1_555   2_655 ......     # 2017 Check
PLAT779_ALERT_4_G Suspect or Irrelevant (Bond) Angle(s) in CIF ...       4.10 Deg.
           C35  -C49  -C12     1_555   1_555   2_655 ......     # 2020 Check
PLAT779_ALERT_4_G Suspect or Irrelevant (Bond) Angle(s) in CIF ...      44.90 Deg.
           C10  -C50  -C47     2_655   1_555   1_555 ......     # 2036 Check
PLAT779_ALERT_4_G Suspect or Irrelevant (Bond) Angle(s) in CIF ...       3.60 Deg.
           C51  -C50  -C76     1_555   1_555   2_655 ......     # 2043 Check
PLAT779_ALERT_4_G Suspect or Irrelevant (Bond) Angle(s) in CIF ...      13.70 Deg.
           C49  -C50  -C11     1_555   1_555   2_655 ......     # 2047 Check
PLAT779_ALERT_4_G Suspect or Irrelevant (Bond) Angle(s) in CIF ...      43.20 Deg.
           C51  -C50  -C17     1_555   1_555   2_655 ......     # 2054 Check
PLAT779_ALERT_4_G Suspect or Irrelevant (Bond) Angle(s) in CIF ...      44.30 Deg.
           C76  -C50  -C17     2_655   1_555   2_655 ......     # 2056 Check
PLAT779_ALERT_4_G Suspect or Irrelevant (Bond) Angle(s) in CIF ...      31.00 Deg.
           C76  -C51  -C64     2_655   1_555   1_555 ......     # 2069 Check
PLAT779_ALERT_4_G Suspect or Irrelevant (Bond) Angle(s) in CIF ...      39.80 Deg.
           C71  -C51  -C50     2_655   1_555   1_555 ......     # 2074 Check
PLAT779_ALERT_4_G Suspect or Irrelevant (Bond) Angle(s) in CIF ...      12.00 Deg.
           C76  -C51  -C75     2_655   1_555   2_655 ......     # 2076 Check
PLAT779_ALERT_4_G Suspect or Irrelevant (Bond) Angle(s) in CIF ...      33.00 Deg.
           C64  -C51  -C75     1_555   1_555   2_655 ......     # 2079 Check
PLAT779_ALERT_4_G Suspect or Irrelevant (Bond) Angle(s) in CIF ...       9.60 Deg.
           C17  -C51  -C52     2_655   1_555   1_555 ......     # 2082 Check
PLAT779_ALERT_4_G Suspect or Irrelevant (Bond) Angle(s) in CIF ...       6.50 Deg.
           C18  -C52  -C55     2_655   1_555   1_555 ......     # 2089 Check
PLAT779_ALERT_4_G Suspect or Irrelevant (Bond) Angle(s) in CIF ...       5.70 Deg.
           C51  -C52  -C76     1_555   1_555   2_655 ......     # 2101 Check
PLAT779_ALERT_4_G Suspect or Irrelevant (Bond) Angle(s) in CIF ...      11.60 Deg.
           C53  -C52  -C16     1_555   1_555   2_655 ......     # 2105 Check
PLAT779_ALERT_4_G Suspect or Irrelevant (Bond) Angle(s) in CIF ...      12.20 Deg.
           C17  -C53  -C52     2_655   1_555   1_555 ......     # 2112 Check
PLAT779_ALERT_4_G Suspect or Irrelevant (Bond) Angle(s) in CIF ...       8.60 Deg.
           C15  -C53  -C29     2_655   1_555   1_555 ......     # 2116 Check
PLAT779_ALERT_4_G Suspect or Irrelevant (Bond) Angle(s) in CIF ...      12.00 Deg.
           C16  -C53  -C49     2_655   1_555   1_555 ......     # 2118 Check
PLAT779_ALERT_4_G Suspect or Irrelevant (Bond) Angle(s) in CIF ...       2.00 Deg.
           C16  -C53  -C11     2_655   1_555   2_655 ......     # 2123 Check
PLAT779_ALERT_4_G Suspect or Irrelevant (Bond) Angle(s) in CIF ...      10.30 Deg.
           C49  -C53  -C11     1_555   1_555   2_655 ......     # 2128 Check
```

```
PLAT779_ALERT_4_G Suspect or Irrelevant (Bond) Angle(s) in CIF ...       7.90 Deg.
            C24  -C54  -C58     2_655   1_555   1_555 ......     # 2138 Check
PLAT779_ALERT_4_G Suspect or Irrelevant (Bond) Angle(s) in CIF ...       6.60 Deg.
            C55  -C54  -C18     1_555   1_555   2_655 ......     # 2149 Check
PLAT779_ALERT_4_G Suspect or Irrelevant (Bond) Angle(s) in CIF ...       9.10 Deg.
            C28  -C54  -C20     1_555   1_555   2_655 ......     # 2155 Check
PLAT779_ALERT_4_G Suspect or Irrelevant (Bond) Angle(s) in CIF ...       6.40 Deg.
            C19  -C55  -C54     2_655   1_555   1_555 ......     # 2172 Check
PLAT779_ALERT_4_G Suspect or Irrelevant (Bond) Angle(s) in CIF ...      16.20 Deg.
            C77  -C55  -C56     2_655   1_555   1_555 ......     # 2177 Check
PLAT779_ALERT_4_G Suspect or Irrelevant (Bond) Angle(s) in CIF ...       3.30 Deg.
            C52  -C55  -C17     1_555   1_555   2_655 ......     # 2180 Check
PLAT779_ALERT_4_G Suspect or Irrelevant (Bond) Angle(s) in CIF ...      38.90 Deg.
            C78  -C56  -C57     2_655   1_555   1_555 ......     # 2189 Check
PLAT779_ALERT_4_G Suspect or Irrelevant (Bond) Angle(s) in CIF ...       6.40 Deg.
            C55  -C56  -C18     1_555   1_555   2_655 ......     # 2199 Check
PLAT779_ALERT_4_G Suspect or Irrelevant (Bond) Angle(s) in CIF ...      31.90 Deg.
            C77  -C56  -C75     2_655   1_555   2_655 ......     # 2200 Check
PLAT779_ALERT_4_G Suspect or Irrelevant (Bond) Angle(s) in CIF ...      36.10 Deg.
            C63  -C56  -C75     1_555   1_555   2_655 ......     # 2202 Check
PLAT779_ALERT_4_G Suspect or Irrelevant (Bond) Angle(s) in CIF ...       5.87 Deg.
            ND5  -C56  -ND1     2_655   1_555   1_555 ......     # 2220 Check
PLAT779_ALERT_4_G Suspect or Irrelevant (Bond) Angle(s) in CIF ...       0.70 Deg.
            C58  -C57  -C24     1_555   1_555   2_655 ......     # 2233 Check
PLAT779_ALERT_4_G Suspect or Irrelevant (Bond) Angle(s) in CIF ...       2.20 Deg.
            C56  -C57  -C77     1_555   1_555   2_655 ......     # 2239 Check
PLAT779_ALERT_4_G Suspect or Irrelevant (Bond) Angle(s) in CIF ...      31.40 Deg.
            C25  -C57  -C26     2_655   1_555   2_655 ......     # 2242 Check
PLAT779_ALERT_4_G Suspect or Irrelevant (Bond) Angle(s) in CIF ...      12.52 Deg.
            ND5  -C57  -ND1     2_655   1_555   1_555 ......     # 2265 Check
PLAT779_ALERT_4_G Suspect or Irrelevant (Bond) Angle(s) in CIF ...      17.91 Deg.
            ND5  -C57  -ND4     2_655   1_555   2_655 ......     # 2274 Check
PLAT779_ALERT_4_G Suspect or Irrelevant (Bond) Angle(s) in CIF ...       5.49 Deg.
            ND1  -C57  -ND4     1_555   1_555   2_655 ......     # 2275 Check
PLAT779_ALERT_4_G Suspect or Irrelevant (Bond) Angle(s) in CIF ...      27.10 Deg.
            C25  -C58  -C57     2_655   1_555   1_555 ......     # 2280 Check
PLAT779_ALERT_4_G Suspect or Irrelevant (Bond) Angle(s) in CIF ...       6.50 Deg.
            C54  -C58  -C19     1_555   1_555   2_655 ......     # 2288 Check
PLAT779_ALERT_4_G Suspect or Irrelevant (Bond) Angle(s) in CIF ...       9.20 Deg.
            C22  -C58  -C23     1_555   1_555   2_655 ......     # 2295 Check
PLAT779_ALERT_4_G Suspect or Irrelevant (Bond) Angle(s) in CIF ...      11.77 Deg.
            ND5  -C58  -ND1     2_655   1_555   1_555 ......     # 2311 Check
PLAT779_ALERT_4_G Suspect or Irrelevant (Bond) Angle(s) in CIF ...       6.50 Deg.
            C14  -C59  -C30     1_555   1_555   2_655 ......     # 2324 Check
PLAT779_ALERT_4_G Suspect or Irrelevant (Bond) Angle(s) in CIF ...      12.90 Deg.
            C21  -C59  -C27     1_555   1_555   2_655 ......     # 2330 Check
PLAT779_ALERT_4_G Suspect or Irrelevant (Bond) Angle(s) in CIF ...      15.07 Deg.
            ND1  -C59  -ND4     1_555   1_555   2_655 ......     # 2347 Check
PLAT779_ALERT_4_G Suspect or Irrelevant (Bond) Angle(s) in CIF ...      12.40 Deg.
            ND1  -C59  -ND5     1_555   1_555   2_655 ......     # 2355 Check
PLAT779_ALERT_4_G Suspect or Irrelevant (Bond) Angle(s) in CIF ...      27.46 Deg.
            ND4  -C59  -ND5     2_655   1_555   2_655 ......     # 2356 Check
PLAT779_ALERT_4_G Suspect or Irrelevant (Bond) Angle(s) in CIF ...      30.70 Deg.
            C32  -C60  -C61     2_655   1_555   1_555 ......     # 2362 Check
PLAT779_ALERT_4_G Suspect or Irrelevant (Bond) Angle(s) in CIF ...      34.50 Deg.
            C26  -C60  -C59     2_655   1_555   1_555 ......     # 2367 Check
PLAT779_ALERT_4_G Suspect or Irrelevant (Bond) Angle(s) in CIF ...      32.50 Deg.
```

```
              C31  -C60  -C59     2_655   1_555   1_555 ......     # 2368 Check
PLAT779_ALERT_4_G Suspect or Irrelevant (Bond) Angle(s) in CIF ...      25.60 Deg.
              C25  -C60  -C57     2_655   1_555   1_555 ......     # 2376 Check
PLAT779_ALERT_4_G Suspect or Irrelevant (Bond) Angle(s) in CIF ...      29.30 Deg.
              C61  -C60  -C79     1_555   1_555   2_655 ......     # 2381 Check
PLAT779_ALERT_4_G Suspect or Irrelevant (Bond) Angle(s) in CIF ...      34.50 Deg.
              C57  -C60  -C78     1_555   1_555   2_655 ......     # 2391 Check
PLAT779_ALERT_4_G Suspect or Irrelevant (Bond) Angle(s) in CIF ...      16.36 Deg.
              ND5  -C60  -ND1     2_655   1_555   1_555 ......     # 2411 Check
PLAT779_ALERT_4_G Suspect or Irrelevant (Bond) Angle(s) in CIF ...      27.57 Deg.
              ND5  -C60  -ND4     2_655   1_555   2_655 ......     # 2421 Check
PLAT779_ALERT_4_G Suspect or Irrelevant (Bond) Angle(s) in CIF ...      11.23 Deg.
              ND1  -C60  -ND4     1_555   1_555   2_655 ......     # 2422 Check
PLAT779_ALERT_4_G Suspect or Irrelevant (Bond) Angle(s) in CIF ...       8.50 Deg.
              C70  -C61  -C33     1_555   1_555   2_655 ......     # 2435 Check
PLAT779_ALERT_4_G Suspect or Irrelevant (Bond) Angle(s) in CIF ...      11.80 Deg.
              C62  -C61  -C80     1_555   1_555   2_655 ......     # 2442 Check
PLAT779_ALERT_4_G Suspect or Irrelevant (Bond) Angle(s) in CIF ...      35.40 Deg.
              C79  -C61  -C78     2_655   1_555   2_655 ......     # 2444 Check
PLAT779_ALERT_4_G Suspect or Irrelevant (Bond) Angle(s) in CIF ...      32.20 Deg.
              C32  -C61  -C31     2_655   1_555   2_655 ......     # 2452 Check
PLAT779_ALERT_4_G Suspect or Irrelevant (Bond) Angle(s) in CIF ...      43.70 Deg.
              C60  -C61  -C31     1_555   1_555   2_655 ......     # 2454 Check
PLAT779_ALERT_4_G Suspect or Irrelevant (Bond) Angle(s) in CIF ...      14.14 Deg.
              ND5  -C61  -ND1     2_655   1_555   1_555 ......     # 2477 Check
PLAT779_ALERT_4_G Suspect or Irrelevant (Bond) Angle(s) in CIF ...      24.41 Deg.
              ND5  -C61  -ND4     2_655   1_555   2_655 ......     # 2487 Check
PLAT779_ALERT_4_G Suspect or Irrelevant (Bond) Angle(s) in CIF ...      10.61 Deg.
              ND1  -C61  -ND4     1_555   1_555   2_655 ......     # 2488 Check
PLAT779_ALERT_4_G Suspect or Irrelevant (Bond) Angle(s) in CIF ...      20.60 Deg.
              C80  -C62  -C67     2_655   1_555   1_555 ......     # 2490 Check
PLAT779_ALERT_4_G Suspect or Irrelevant (Bond) Angle(s) in CIF ...      30.00 Deg.
              C79  -C62  -C61     2_655   1_555   1_555 ......     # 2496 Check
PLAT779_ALERT_4_G Suspect or Irrelevant (Bond) Angle(s) in CIF ...      31.30 Deg.
              C80  -C62  -C39     2_655   1_555   2_655 ......     # 2504 Check
PLAT779_ALERT_4_G Suspect or Irrelevant (Bond) Angle(s) in CIF ...      10.80 Deg.
              C67  -C62  -C39     1_555   1_555   2_655 ......     # 2506 Check
PLAT779_ALERT_4_G Suspect or Irrelevant (Bond) Angle(s) in CIF ...      10.05 Deg.
              ND5  -C62  -ND1     2_655   1_555   1_555 ......     # 2533 Check
PLAT779_ALERT_4_G Suspect or Irrelevant (Bond) Angle(s) in CIF ...      18.19 Deg.
              ND2  -C62  -ND6     2_655   1_555   1_555 ......     # 2541 Check
PLAT779_ALERT_4_G Suspect or Irrelevant (Bond) Angle(s) in CIF ...      41.56 Deg.
              ND1  -C62  -ND6     1_555   1_555   1_555 ......     # 2543 Check
PLAT779_ALERT_4_G Suspect or Irrelevant (Bond) Angle(s) in CIF ...      17.70 Deg.
              C77  -C63  -C56     2_655   1_555   1_555 ......     # 2549 Check
PLAT779_ALERT_4_G Suspect or Irrelevant (Bond) Angle(s) in CIF ...      30.70 Deg.
              C75  -C63  -C64     2_655   1_555   1_555 ......     # 2554 Check
PLAT779_ALERT_4_G Suspect or Irrelevant (Bond) Angle(s) in CIF ...      15.70 Deg.
              C62  -C63  -C80     1_555   1_555   2_655 ......     # 2563 Check
PLAT779_ALERT_4_G Suspect or Irrelevant (Bond) Angle(s) in CIF ...      38.00 Deg.
              C56  -C63  -C78     1_555   1_555   2_655 ......     # 2568 Check
PLAT779_ALERT_4_G Suspect or Irrelevant (Bond) Angle(s) in CIF ...      34.40 Deg.
              C62  -C63  -C79     1_555   1_555   2_655 ......     # 2576 Check
PLAT779_ALERT_4_G Suspect or Irrelevant (Bond) Angle(s) in CIF ...      43.36 Deg.
              ND5  -C63  -ND2     2_655   1_555   2_655 ......     # 2598 Check
PLAT779_ALERT_4_G Suspect or Irrelevant (Bond) Angle(s) in CIF ...       3.60 Deg.
              C76  -C64  -C51     2_655   1_555   1_555 ......     # 2607 Check
```

```
PLAT779_ALERT_4_G Suspect or Irrelevant (Bond) Angle(s) in CIF ...      37.90 Deg.
              C75  -C64  -C63     2_655   1_555   1_555 ......     # 2609 Check
PLAT779_ALERT_4_G Suspect or Irrelevant (Bond) Angle(s) in CIF ...      17.70 Deg.
              C65  -C64  -C73     1_555   1_555   2_655 ......     # 2617 Check
PLAT779_ALERT_4_G Suspect or Irrelevant (Bond) Angle(s) in CIF ...      13.10 Deg.
              C73  -C65  -C66     2_655   1_555   1_555 ......     # 2638 Check
PLAT779_ALERT_4_G Suspect or Irrelevant (Bond) Angle(s) in CIF ...       5.90 Deg.
              C72  -C65  -C46     2_655   1_555   1_555 ......     # 2642 Check
PLAT779_ALERT_4_G Suspect or Irrelevant (Bond) Angle(s) in CIF ...      20.20 Deg.
              C73  -C65  -C40     2_655   1_555   2_655 ......     # 2650 Check
PLAT779_ALERT_4_G Suspect or Irrelevant (Bond) Angle(s) in CIF ...       7.70 Deg.
              C66  -C65  -C40     1_555   1_555   2_655 ......     # 2653 Check
PLAT779_ALERT_4_G Suspect or Irrelevant (Bond) Angle(s) in CIF ...       8.20 Deg.
              C73  -C66  -C65     2_655   1_555   1_555 ......     # 2675 Check
PLAT779_ALERT_4_G Suspect or Irrelevant (Bond) Angle(s) in CIF ...      15.90 Deg.
              C67  -C66  -C39     1_555   1_555   2_655 ......     # 2685 Check
PLAT779_ALERT_4_G Suspect or Irrelevant (Bond) Angle(s) in CIF ...      36.10 Deg.
              C40  -C66  -C41     2_655   1_555   2_655 ......     # 2686 Check
PLAT779_ALERT_4_G Suspect or Irrelevant (Bond) Angle(s) in CIF ...      16.00 Deg.
              C69  -C66  -C41     1_555   1_555   2_655 ......     # 2688 Check
PLAT779_ALERT_4_G Suspect or Irrelevant (Bond) Angle(s) in CIF ...      17.05 Deg.
              ND2  -C66  -ND6     2_655   1_555   1_555 ......     # 2715 Check
PLAT779_ALERT_4_G Suspect or Irrelevant (Bond) Angle(s) in CIF ...       9.40 Deg.
              C80  -C67  -C62     2_655   1_555   1_555 ......     # 2718 Check
PLAT779_ALERT_4_G Suspect or Irrelevant (Bond) Angle(s) in CIF ...      27.00 Deg.
              C39  -C67  -C68     2_655   1_555   1_555 ......     # 2719 Check
PLAT779_ALERT_4_G Suspect or Irrelevant (Bond) Angle(s) in CIF ...      19.80 Deg.
              C40  -C67  -C66     2_655   1_555   1_555 ......     # 2730 Check
PLAT779_ALERT_4_G Suspect or Irrelevant (Bond) Angle(s) in CIF ...      28.10 Deg.
              C39  -C67  -C38     2_655   1_555   2_655 ......     # 2737 Check
PLAT779_ALERT_4_G Suspect or Irrelevant (Bond) Angle(s) in CIF ...       3.20 Deg.
              C68  -C67  -C38     1_555   1_555   2_655 ......     # 2740 Check
PLAT779_ALERT_4_G Suspect or Irrelevant (Bond) Angle(s) in CIF ...      43.20 Deg.
              C40  -C67  -C73     2_655   1_555   2_655 ......     # 2748 Check
PLAT779_ALERT_4_G Suspect or Irrelevant (Bond) Angle(s) in CIF ...      24.40 Deg.
              C66  -C67  -C73     1_555   1_555   2_655 ......     # 2749 Check
PLAT779_ALERT_4_G Suspect or Irrelevant (Bond) Angle(s) in CIF ...      42.70 Deg.
              C74  -C67  -C73     2_655   1_555   2_655 ......     # 2750 Check
PLAT779_ALERT_4_G Suspect or Irrelevant (Bond) Angle(s) in CIF ...      21.24 Deg.
              ND2  -C67  -ND6     2_655   1_555   1_555 ......     # 2770 Check
PLAT779_ALERT_4_G Suspect or Irrelevant (Bond) Angle(s) in CIF ...      10.90 Deg.
              C39  -C68  -C67     2_655   1_555   1_555 ......     # 2775 Check
PLAT779_ALERT_4_G Suspect or Irrelevant (Bond) Angle(s) in CIF ...      14.90 Deg.
              C70  -C68  -C33     1_555   1_555   2_655 ......     # 2785 Check
PLAT779_ALERT_4_G Suspect or Irrelevant (Bond) Angle(s) in CIF ...      43.80 Deg.
              C38  -C68  -C37     2_655   1_555   2_655 ......     # 2786 Check
PLAT779_ALERT_4_G Suspect or Irrelevant (Bond) Angle(s) in CIF ...      16.40 Deg.
              C7   -C68  -C37     1_555   1_555   2_655 ......     # 2788 Check
PLAT779_ALERT_4_G Suspect or Irrelevant (Bond) Angle(s) in CIF ...      23.19 Deg.
              ND6  -C68  -ND2     1_555   1_555   2_655 ......     # 2806 Check
PLAT779_ALERT_4_G Suspect or Irrelevant (Bond) Angle(s) in CIF ...      16.59 Deg.
              ND6  -C68  -ND3     1_555   1_555   1_555 ......     # 2814 Check
PLAT779_ALERT_4_G Suspect or Irrelevant (Bond) Angle(s) in CIF ...      39.73 Deg.
              ND2  -C68  -ND3     2_655   1_555   1_555 ......     # 2815 Check
PLAT779_ALERT_4_G Suspect or Irrelevant (Bond) Angle(s) in CIF ...      20.50 Deg.
              C40  -C69  -C66     2_655   1_555   1_555 ......     # 2820 Check
PLAT779_ALERT_4_G Suspect or Irrelevant (Bond) Angle(s) in CIF ...      35.60 Deg.
```

```
              C2   -C69  -C45     2_655   1_555   1_555 ......     # 2824 Check
PLAT779_ALERT_4_G Suspect or Irrelevant (Bond) Angle(s) in CIF ...       8.90 Deg.
              C41  -C69  -C6      2_655   1_555   1_555 ......     # 2826 Check
PLAT779_ALERT_4_G Suspect or Irrelevant (Bond) Angle(s) in CIF ...      43.70 Deg.
              C40  -C69  -C73     2_655   1_555   2_655 ......     # 2832 Check
PLAT779_ALERT_4_G Suspect or Irrelevant (Bond) Angle(s) in CIF ...      24.10 Deg.
              C66  -C69  -C73     1_555   1_555   2_655 ......     # 2834 Check
PLAT779_ALERT_4_G Suspect or Irrelevant (Bond) Angle(s) in CIF ...      19.49 Deg.
              ND2  -C69  -ND6     2_655   1_555   1_555 ......     # 2851 Check
PLAT779_ALERT_4_G Suspect or Irrelevant (Bond) Angle(s) in CIF ...      33.30 Deg.
              C32  -C70  -C61     2_655   1_555   1_555 ......     # 2856 Check
PLAT779_ALERT_4_G Suspect or Irrelevant (Bond) Angle(s) in CIF ...      29.50 Deg.
              C33  -C70  -C13     2_655   1_555   1_555 ......     # 2858 Check
PLAT779_ALERT_4_G Suspect or Irrelevant (Bond) Angle(s) in CIF ...      17.50 Deg.
              C38  -C70  -C68     2_655   1_555   1_555 ......     # 2864 Check
PLAT779_ALERT_4_G Suspect or Irrelevant (Bond) Angle(s) in CIF ...      23.30 Deg.
              C33  -C70  -C34     2_655   1_555   2_655 ......     # 2867 Check
PLAT779_ALERT_4_G Suspect or Irrelevant (Bond) Angle(s) in CIF ...       6.30 Deg.
              C13  -C70  -C34     1_555   1_555   2_655 ......     # 2871 Check
PLAT779_ALERT_4_G Suspect or Irrelevant (Bond) Angle(s) in CIF ...      11.68 Deg.
              ND1  -C70  -ND4     1_555   1_555   2_655 ......     # 2896 Check
PLAT779_ALERT_4_G Suspect or Irrelevant (Bond) Angle(s) in CIF ...      11.53 Deg.
              ND1  -C70  -ND5     1_555   1_555   2_655 ......     # 2905 Check
PLAT779_ALERT_4_G Suspect or Irrelevant (Bond) Angle(s) in CIF ...      22.80 Deg.
              ND4  -C70  -ND5     2_655   1_555   2_655 ......     # 2906 Check
PLAT779_ALERT_4_G Suspect or Irrelevant (Bond) Angle(s) in CIF ...      19.71 Deg.
              ND6  -C70  -ND2     1_555   1_555   2_655 ......     # 2914 Check
PLAT779_ALERT_4_G Suspect or Irrelevant (Bond) Angle(s) in CIF ...      42.23 Deg.
              ND5  -C70  -ND2     2_655   1_555   2_655 ......     # 2917 Check
PLAT779_ALERT_4_G Suspect or Irrelevant (Bond) Angle(s) in CIF ...      42.50 Deg.
              C50  -C71  -C10     2_655   1_555   1_555 ......     # 2921 Check
PLAT779_ALERT_4_G Suspect or Irrelevant (Bond) Angle(s) in CIF ...       5.70 Deg.
              C51  -C71  -C76     2_655   1_555   1_555 ......     # 2930 Check
PLAT779_ALERT_4_G Suspect or Irrelevant (Bond) Angle(s) in CIF ...      15.30 Deg.
              C72  -C71  -C46     1_555   1_555   2_655 ......     # 2937 Check
PLAT779_ALERT_4_G Suspect or Irrelevant (Bond) Angle(s) in CIF ...      42.20 Deg.
              C76  -C71  -C64     1_555   1_555   2_655 ......     # 2944 Check
PLAT779_ALERT_4_G Suspect or Irrelevant (Bond) Angle(s) in CIF ...      32.70 Deg.
              C72  -C71  -C65     1_555   1_555   2_655 ......     # 2950 Check
PLAT779_ALERT_4_G Suspect or Irrelevant (Bond) Angle(s) in CIF ...      42.20 Deg.
              C64  -C71  -C65     2_655   1_555   2_655 ......     # 2953 Check
PLAT779_ALERT_4_G Suspect or Irrelevant (Bond) Angle(s) in CIF ...      26.00 Deg.
              C46  -C72  -C3      2_655   1_555   1_555 ......     # 2966 Check
PLAT779_ALERT_4_G Suspect or Irrelevant (Bond) Angle(s) in CIF ...      17.90 Deg.
              C65  -C72  -C73     2_655   1_555   1_555 ......     # 2970 Check
PLAT779_ALERT_4_G Suspect or Irrelevant (Bond) Angle(s) in CIF ...      43.90 Deg.
              C71  -C72  -C47     1_555   1_555   2_655 ......     # 2975 Check
PLAT779_ALERT_4_G Suspect or Irrelevant (Bond) Angle(s) in CIF ...      23.10 Deg.
              C3   -C72  -C45     1_555   1_555   2_655 ......     # 2981 Check
PLAT779_ALERT_4_G Suspect or Irrelevant (Bond) Angle(s) in CIF ...       8.20 Deg.
              C66  -C73  -C40     2_655   1_555   1_555 ......     # 2993 Check
PLAT779_ALERT_4_G Suspect or Irrelevant (Bond) Angle(s) in CIF ...      35.30 Deg.
              C65  -C73  -C72     2_655   1_555   1_555 ......     # 2997 Check
PLAT779_ALERT_4_G Suspect or Irrelevant (Bond) Angle(s) in CIF ...      43.40 Deg.
              C74  -C73  -C64     1_555   1_555   2_655 ......     # 3004 Check
PLAT779_ALERT_4_G Suspect or Irrelevant (Bond) Angle(s) in CIF ...      43.50 Deg.
              C65  -C73  -C46     2_655   1_555   2_655 ......     # 3006 Check
```

```
PLAT779_ALERT_4_G Suspect or Irrelevant (Bond) Angle(s) in CIF ...       8.30 Deg.
              C72  -C73  -C46     1_555   1_555   2_655 ......     # 3010 Check
PLAT779_ALERT_4_G Suspect or Irrelevant (Bond) Angle(s) in CIF ...      40.00 Deg.
              C66  -C73  -C69     2_655   1_555   2_655 ......     # 3013 Check
PLAT779_ALERT_4_G Suspect or Irrelevant (Bond) Angle(s) in CIF ...      35.30 Deg.
              C40  -C73  -C69     1_555   1_555   2_655 ......     # 3014 Check
PLAT779_ALERT_4_G Suspect or Irrelevant (Bond) Angle(s) in CIF ...      44.70 Deg.
              C66  -C73  -C67     2_655   1_555   2_655 ......     # 3020 Check
PLAT779_ALERT_4_G Suspect or Irrelevant (Bond) Angle(s) in CIF ...      37.10 Deg.
              C64  -C74  -C75     2_655   1_555   1_555 ......     # 3039 Check
PLAT779_ALERT_4_G Suspect or Irrelevant (Bond) Angle(s) in CIF ...      21.70 Deg.
              C73  -C74  -C65     1_555   1_555   2_655 ......     # 3050 Check
PLAT779_ALERT_4_G Suspect or Irrelevant (Bond) Angle(s) in CIF ...      16.90 Deg.
              C80  -C74  -C62     1_555   1_555   2_655 ......     # 3053 Check
PLAT779_ALERT_4_G Suspect or Irrelevant (Bond) Angle(s) in CIF ...      32.40 Deg.
              C80  -C74  -C67     1_555   1_555   2_655 ......     # 3059 Check
PLAT779_ALERT_4_G Suspect or Irrelevant (Bond) Angle(s) in CIF ...      27.80 Deg.
              C73  -C74  -C66     1_555   1_555   2_655 ......     # 3068 Check
PLAT779_ALERT_4_G Suspect or Irrelevant (Bond) Angle(s) in CIF ...       1.30 Deg.
              C76  -C75  -C51     1_555   1_555   2_655 ......     # 3094 Check
PLAT779_ALERT_4_G Suspect or Irrelevant (Bond) Angle(s) in CIF ...       9.40 Deg.
              C77  -C75  -C56     1_555   1_555   2_655 ......     # 3098 Check
PLAT779_ALERT_4_G Suspect or Irrelevant (Bond) Angle(s) in CIF ...      36.70 Deg.
              C64  -C76  -C75     2_655   1_555   1_555 ......     # 3104 Check
PLAT779_ALERT_4_G Suspect or Irrelevant (Bond) Angle(s) in CIF ...      35.00 Deg.
              C51  -C76  -C50     2_655   1_555   2_655 ......     # 3112 Check
PLAT779_ALERT_4_G Suspect or Irrelevant (Bond) Angle(s) in CIF ...      37.10 Deg.
              C71  -C76  -C50     1_555   1_555   2_655 ......     # 3116 Check
PLAT779_ALERT_4_G Suspect or Irrelevant (Bond) Angle(s) in CIF ...      10.10 Deg.
              C17  -C76  -C52     1_555   1_555   2_655 ......     # 3120 Check
PLAT779_ALERT_4_G Suspect or Irrelevant (Bond) Angle(s) in CIF ...      44.80 Deg.
              C63  -C77  -C75     2_655   1_555   1_555 ......     # 3125 Check
PLAT779_ALERT_4_G Suspect or Irrelevant (Bond) Angle(s) in CIF ...       7.00 Deg.
              C55  -C77  -C18     2_655   1_555   1_555 ......     # 3132 Check
PLAT779_ALERT_4_G Suspect or Irrelevant (Bond) Angle(s) in CIF ...      21.10 Deg.
              C56  -C77  -C78     2_655   1_555   1_555 ......     # 3133 Check
PLAT779_ALERT_4_G Suspect or Irrelevant (Bond) Angle(s) in CIF ...       7.30 Deg.
              C56  -C77  -C57     2_655   1_555   2_655 ......     # 3138 Check
PLAT779_ALERT_4_G Suspect or Irrelevant (Bond) Angle(s) in CIF ...      28.30 Deg.
              C78  -C77  -C57     1_555   1_555   2_655 ......     # 3143 Check
PLAT779_ALERT_4_G Suspect or Irrelevant (Bond) Angle(s) in CIF ...       8.40 Deg.
              C56  -C78  -C77     2_655   1_555   1_555 ......     # 3153 Check
PLAT779_ALERT_4_G Suspect or Irrelevant (Bond) Angle(s) in CIF ...      14.20 Deg.
              C57  -C78  -C25     2_655   1_555   1_555 ......     # 3157 Check
PLAT779_ALERT_4_G Suspect or Irrelevant (Bond) Angle(s) in CIF ...      17.20 Deg.
              C79  -C78  -C61     1_555   1_555   2_655 ......     # 3175 Check
PLAT779_ALERT_4_G Suspect or Irrelevant (Bond) Angle(s) in CIF ...      44.90 Deg.
              C60  -C78  -C61     2_655   1_555   2_655 ......     # 3177 Check
PLAT779_ALERT_4_G Suspect or Irrelevant (Bond) Angle(s) in CIF ...      10.56 Deg.
              ND5  -C78  -ND1     1_555   1_555   2_655 ......     # 3195 Check
PLAT779_ALERT_4_G Suspect or Irrelevant (Bond) Angle(s) in CIF ...      18.20 Deg.
              C61  -C79  -C32     2_655   1_555   1_555 ......     # 3197 Check
PLAT779_ALERT_4_G Suspect or Irrelevant (Bond) Angle(s) in CIF ...       7.10 Deg.
              C62  -C79  -C80     2_655   1_555   1_555 ......     # 3207 Check
PLAT779_ALERT_4_G Suspect or Irrelevant (Bond) Angle(s) in CIF ...      12.30 Deg.
              ND5  -C79  -ND1     1_555   1_555   2_655 ......     # 3231 Check
PLAT779_ALERT_4_G Suspect or Irrelevant (Bond) Angle(s) in CIF ...      42.69 Deg.
```

```
             ND5  -C79  -ND2     1_555   1_555   1_555 ......     # 3239 Check
PLAT779_ALERT_4_G Suspect or Irrelevant (Bond) Angle(s) in CIF ...      19.62 Deg.
             ND5  -C79  -ND4     1_555   1_555   1_555 ......     # 3248 Check
PLAT779_ALERT_4_G Suspect or Irrelevant (Bond) Angle(s) in CIF ...       7.64 Deg.
             ND1  -C79  -ND4     2_655   1_555   1_555 ......     # 3249 Check
PLAT779_ALERT_4_G Suspect or Irrelevant (Bond) Angle(s) in CIF ...      11.50 Deg.
             C67  -C80  -C39     2_655   1_555   1_555 ......     # 3255 Check
PLAT779_ALERT_4_G Suspect or Irrelevant (Bond) Angle(s) in CIF ...      16.70 Deg.
             C62  -C80  -C79     2_655   1_555   1_555 ......     # 3257 Check
PLAT779_ALERT_4_G Suspect or Irrelevant (Bond) Angle(s) in CIF ...      39.90 Deg.
             C62  -C80  -C61     2_655   1_555   2_655 ......     # 3266 Check
PLAT779_ALERT_4_G Suspect or Irrelevant (Bond) Angle(s) in CIF ...      24.60 Deg.
             C79  -C80  -C61     1_555   1_555   2_655 ......     # 3270 Check
PLAT779_ALERT_4_G Suspect or Irrelevant (Bond) Angle(s) in CIF ...      18.45 Deg.
             ND2  -C80  -ND6     1_555   1_555   2_655 ......     # 3286 Check
PLAT779_ALERT_4_G Suspect or Irrelevant (Bond) Angle(s) in CIF ...      43.56 Deg.
             ND2  -C80  -ND5     1_555   1_555   1_555 ......     # 3294 Check
PLAT779_ALERT_4_G Suspect or Irrelevant (Bond) Angle(s) in CIF ...      43.96 Deg.
             ND6  -C80  -ND5     2_655   1_555   1_555 ......     # 3295 Check
PLAT779_ALERT_4_G Suspect or Irrelevant (Bond) Angle(s) in CIF ...      35.50 Deg.
             F3   -C81  -F2      2_655   1_555   1_555 ......     # 3301 Check
PLAT779_ALERT_4_G Suspect or Irrelevant (Bond) Angle(s) in CIF ...      35.30 Deg.
             F2   -C81  -F3      2_655   1_555   1_555 ......     # 3307 Check
PLAT779_ALERT_4_G Suspect or Irrelevant (Bond) Angle(s) in CIF ...      14.00 Deg.
             C1   -C81  -C1      1_555   1_555   2_655 ......     # 3323 Check
PLAT779_ALERT_4_G Suspect or Irrelevant (Bond) Angle(s) in CIF ...      15.70 Deg.
             C81  -C81  -F1      2_655   1_555   2_655 ......     # 3324 Check
PLAT779_ALERT_4_G Suspect or Irrelevant (Bond) Angle(s) in CIF ...       5.00 Deg.
             C81  -F1   -C81     1_555   1_555   2_655 ......     # 3335 Check
PLAT779_ALERT_4_G Suspect or Irrelevant (Bond) Angle(s) in CIF ...      18.90 Deg.
             C81  -F2   -C81     2_655   1_555   1_555 ......     # 3340 Check
PLAT779_ALERT_4_G Suspect or Irrelevant (Bond) Angle(s) in CIF ...      18.80 Deg.
             C81  -F3   -C81     2_655   1_555   1_555 ......     # 3346 Check
PLAT789_ALERT_4_G Atoms with Negative _atom_site_disorder_group  #         12 Check
PLAT793_ALERT_4_G Model has Chirality at C1          (Centro SPGR)          R Verify
PLAT860_ALERT_3_G Number of Least-Squares Restraints ............       1957 Note
PLAT883_ALERT_1_G No Info/Value for _atom_sites_solution_primary .     Please Do !
PLAT912_ALERT_4_G Missing # of FCF Reflections Above STh/L=  0.600       1961 Note
PLAT913_ALERT_3_G Missing # of Very Strong Reflections in FCF ....          2 Note
PLAT950_ALERT_5_G Calculated (ThMax) and CIF-Reported Hmax Differ           4 Units
PLAT956_ALERT_1_G Calculated (ThMax) and Actual (FCF) Hmax Differ           4 Units
PLAT984_ALERT_1_G The  F-f'=    0.0175 Deviates from the B&C-Value     0.0214 Check
PLAT984_ALERT_1_G The  N-f'=    0.0058 Deviates from the B&C-Value     0.0077 Check
PLAT984_ALERT_1_G The Nd-f'=   -0.4524 Deviates from the B&C-Value    -0.0672 Check
PLAT984_ALERT_1_G The Ni-f'=    0.2631 Deviates from the B&C-Value     0.3241 Check
PLAT985_ALERT_1_G The Nd-f"=    3.5469 Deviates from the B&C-Value     3.5589 Check
PLAT985_ALERT_1_G The Ni-f"=    1.3158 Deviates from the B&C-Value     1.3169 Check
PLAT992_ALERT_5_G Repd & Actual _reflns_number_gt Values Differ by          3 Check
```

---

   0 **ALERT level A** = Most likely a serious problem - resolve or explain
   0 **ALERT level B** = A potentially serious problem, consider carefully
  60 **ALERT level C** = Check. Ensure it is not caused by an omission or oversight
 658 **ALERT level G** = General information/check it is not something unexpected

  44 ALERT type 1 CIF construction/syntax error, inconsistent or missing data

```
 65 ALERT type 2 Indicator that the structure model may be wrong or deficient
 21 ALERT type 3 Indicator that the structure quality may be low
586 ALERT type 4 Improvement, methodology, query or suggestion
  2 ALERT type 5 Informative message, check
```

It is advisable to attempt to resolve as many as possible of the alerts in all categories. Often the minor alerts point to easily fixed oversights, errors and omissions in your CIF or refinement strategy, so attention to these fine details can be worthwhile. In order to resolve some of the more serious problems it may be necessary to carry out additional measurements or structure refinements. However, the purpose of your study may justify the reported deviations and the more serious of these should normally be commented upon in the discussion or experimental section of a paper or in the "special_details" fields of the CIF. checkCIF was carefully designed to identify outliers and unusual parameters, but every test has its limitations and alerts that are not important in a particular case may appear. Conversely, the absence of alerts does not guarantee there are no aspects of the results needing attention. It is up to the individual to critically assess their own results and, if necessary, seek expert advice.

Publication of your CIF in IUCr journals

A basic structural check has been run on your CIF. These basic checks will be run on all CIFs submitted for publication in IUCr journals (*Acta Crystallographica*, *Journal of Applied Crystallography*, *Journal of Synchrotron Radiation*); however, if you intend to submit to *Acta Crystallographica Section C* or *E* or *IUCrData*, you should make sure that full publication checks are run on the final version of your CIF prior to submission.

Publication of your CIF in other journals

Please refer to the *Notes for Authors* of the relevant journal for any special instructions relating to CIF submission.

PLATON version of 20/01/2022; check.def file version of 19/01/2022

Datablock nd2@c80-cf3-ii - ellipsoid plot

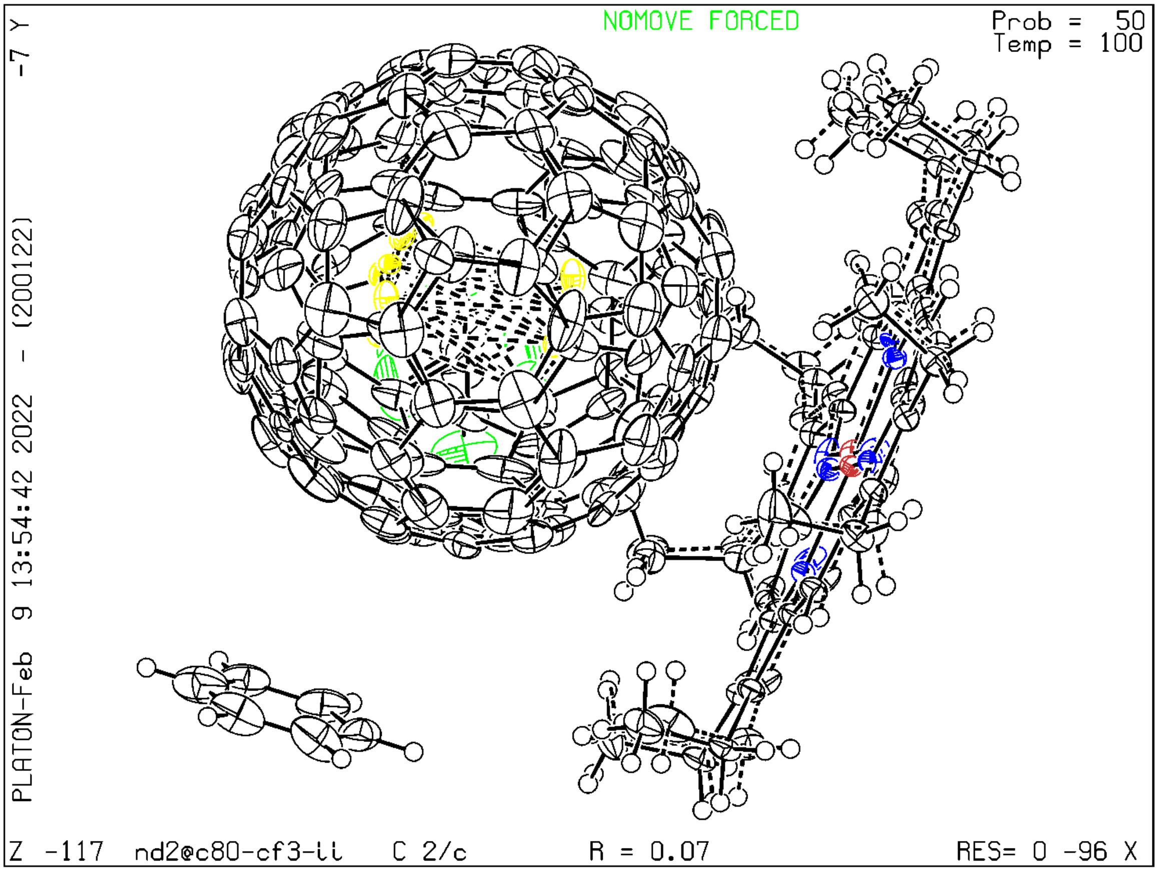

Supplement: Supplementary file 2 — Supporting Information [file ADVS-11-2305190-s002.zip › advs202305190-sup-0002-cif/nd2@c80-cf3-ii_submission_checkcif.pdf]
