# Supplementary material for: Nd─Nd Bond in Ih and D5h Cage Isomers of Nd2@C80 Stabilized by Electrophilic CF3 Addition
Source: Adv Sci (Weinh). 2023 Nov 9;11(1):2305190. doi: 10.1002/advs.202305190 (PMC10767449; doi:10.1002/advs.202305190)

checkCIF/PLATON report

Structure factors have been supplied for datablock(s) nd2@c80-cf3-i

THIS REPORT IS FOR GUIDANCE ONLY. IF USED AS PART OF A REVIEW PROCEDURE FOR PUBLICATION, IT SHOULD NOT REPLACE THE EXPERTISE OF AN EXPERIENCED CRYSTALLOGRAPHIC REFEREE.

No syntax errors found. CIF dictionary Interpreting this report

Datablock: nd2@c80-cf3-i

Bond precision:	C-C = 0.0070 A	Wavelength=0.79990	
Cell:	a=43.010(9)	b=41.170(8)	c=23.490(5)
	alpha=90	beta=97.04(3)	gamma=90
Temperature:	100 K		

	Calculated	Reported
Volume	41281(15)	41281(15)
Space group	C 2/c	C 2/c
Hall group	-C 2yc	-C 2yc
Moiety formula	2(C81 F3 Nd2), 2(C81 F3), 4(C36 H44.50 N4 Ni), 2(C36 H44.42 N4	;2Nd2@C80-Cf3/4NiOeP/1.63ToLuEnE/0.37BeNzEnE;
Sum formula	C639.27 H386.23 F12 N32 Nd8 Ni8	C319.63 H193.11 F6 N16 Nd4 Ni4
Mr	10366.50	5183.39
Dx,g cm-3	1.668	1.668
Z	4	8
Mu (mm-1)	1.936	1.956
F000	21031.0	21031.0
F000'	21049.43	
h,k,lmax	64,61,35	63,60,33
Nref	71874	64226
Tmin,Tmax	0.829,0.872	
Tmin'	0.822	

Correction method= Not given

Data completeness= 0.894 Theta(max)= 36.651

R(reflections)= 0.0883(55817) wR2(reflections)= 0.2357(64226)

S = 1.067 Npar= 4508

The following ALERTS were generated. Each ALERT has the format
test-name_ALERT_alert-type_alert-level.
Click on the hyperlinks for more details of the test.

Alert level A

PLAT308_ALERT_2_A Single Bonded Metal Atom in Structure (Unusual) Nd1B Check

Author Response: This is metal atom encapsulated within C80 with disorder.

PLAT308_ALERT_2_A Single Bonded Metal Atom in Structure (Unusual) Nd2B Check

Author Response: This is metal atom encapsulated within C80 with disorder.

PLAT308_ALERT_2_A Single Bonded Metal Atom in Structure (Unusual) Nd1C Check

Author Response: This is metal atom encapsulated within C80 with disorder.

PLAT308_ALERT_2_A Single Bonded Metal Atom in Structure (Unusual) Nd2C Check

Author Response: This is metal atom encapsulated within C80 with disorder.

PLAT308_ALERT_2_A Single Bonded Metal Atom in Structure (Unusual) Nd3B Check

Author Response: This is metal atom encapsulated within C80 with disorder.

PLAT308_ALERT_2_A Single Bonded Metal Atom in Structure (Unusual) Nd4B Check

Author Response: This is metal atom encapsulated within C80 with disorder.

PLAT308_ALERT_2_A Single Bonded Metal Atom in Structure (Unusual) Nd3C Check

Author Response: This is metal atom encapsulated within C80 with disorder.

```
PLAT308_ALERT_2_A Single Bonded Metal Atom in Structure  (Unusual)       Nd4C Check
```

Author Response: This is metal atom encapsulated within C80 with disorder.

Alert level B

```
PLAT213_ALERT_2_B Atom Nd3A            has ADP max/min Ratio .....         4.4 prolat
PLAT221_ALERT_2_B Solv./Anion  Resd 4 Ni   Ueq(max)/Ueq(min) Range       10.0 Ratio
PLAT972_ALERT_2_B Check Calcd Resid. Dens.  0.25Ang From Nd3A           -2.67 eA-3
```

Alert level C

```
PLAT041_ALERT_1_C Calc. and Reported SumFormula    Strings  Differ     Please Check
PLAT052_ALERT_1_C Info on Absorption Correction Method   Not Given     Please Do !
PLAT213_ALERT_2_C Atom Nd4A            has ADP max/min Ratio .....         3.3 prolat
PLAT213_ALERT_2_C Atom Nd6A            has ADP max/min Ratio .....         3.3 prolat
PLAT213_ALERT_2_C Atom C41A            has ADP max/min Ratio .....         3.1 prolat
PLAT213_ALERT_2_C Atom C52A            has ADP max/min Ratio .....         3.1 prolat
PLAT213_ALERT_2_C Atom C58A            has ADP max/min Ratio .....         3.3 prolat
PLAT216_ALERT_3_C Disordered C11B      (An/Solv) ADP max/min Ratio         5.2 Note
PLAT216_ALERT_3_C Disordered C70C      (An/Solv) ADP max/min Ratio         6.6 Note
PLAT220_ALERT_2_C NonSolvent   Resd 1 Nd   Ueq(max)/Ueq(min) Range         3.6 Ratio
PLAT221_ALERT_2_C Solv./Anion  Resd 6  C   Ueq(max)/Ueq(min) Range         4.1 Ratio
PLAT221_ALERT_2_C Solv./Anion  Resd 6 Ni   Ueq(max)/Ueq(min) Range         7.2 Ratio
PLAT223_ALERT_4_C Solv./Anion  Resd 6  H   Ueq(max)/Ueq(min) Range         5.2 Ratio
PLAT242_ALERT_2_C Low    'MainMol' Ueq as Compared to Neighbors of        C68A Check
PLAT250_ALERT_2_C Large U3/U1 Ratio for Average U(i,j) Tensor ....        2.7 Note
PLAT250_ALERT_2_C Large U3/U1 Ratio for Average U(i,j) Tensor ....        3.4 Note
PLAT723_ALERT_1_C Torsion Calc        0.00, Rep      -3(35) Dev...        3.00 Sigma
              C57C-C10C-C11C-C49C  2_556  1_555  1_555  2_556      # 1760 Check
PLAT723_ALERT_1_C Torsion Calc     -166.00, Rep    -168(36) Dev...        2.00 Sigma
              C57C-C10C-C11C-C12C  2_556  1_555  1_555  1_555      # 1767 Check
PLAT723_ALERT_1_C Torsion Calc      -25.00, Rep     -27(34) Dev...        2.00 Sigma
              C57C-C10C-C11C-C64C  2_556  1_555  1_555  1_555      # 1774 Check
PLAT723_ALERT_1_C Torsion Calc     -176.00, Rep    -178(**) Dev...        2.00 Sigma
              C57C-C10C-C11C-C36C  2_556  1_555  1_555  2_556      # 1781 Check
PLAT723_ALERT_1_C Torsion Calc      -45.00, Rep     -48(34) Dev...        3.00 Sigma
              C57C-C10C-C11C-C56C  2_556  1_555  1_555  2_556      # 1788 Check
PLAT723_ALERT_1_C Torsion Calc     -103.00, Rep    -105(35) Dev...        2.00 Sigma
              C57C-C10C-C11C-ND2C  2_556  1_555  1_555  2_556      # 1795 Check
PLAT723_ALERT_1_C Torsion Calc      -95.00, Rep     -97(35) Dev...        2.00 Sigma
              C57C-C10C-C11C-ND1C  2_556  1_555  1_555  1_555      # 1801 Check
PLAT723_ALERT_1_C Torsion Calc      157.00, Rep     160(69) Dev...        3.00 Sigma
              C62C-C49C-C50C-C63C  2_556  1_555  1_555  2_556      # 4645 Check
PLAT723_ALERT_1_C Torsion Calc       -5.00, Rep      -2(68) Dev...        3.00 Sigma
              C62C-C49C-C50C-C64C  2_556  1_555  1_555  2_556      # 4654 Check
PLAT723_ALERT_1_C Torsion Calc        0.00, Rep       3(68) Dev...        3.00 Sigma
              C62C-C49C-C50C-C55C  2_556  1_555  1_555  1_555      # 4662 Check
PLAT723_ALERT_1_C Torsion Calc      155.00, Rep     158(70) Dev...        3.00 Sigma
              C62C-C49C-C50C-C51C  2_556  1_555  1_555  1_555      # 4671 Check
PLAT723_ALERT_1_C Torsion Calc      102.00, Rep     105(70) Dev...        3.00 Sigma
              C62C-C49C-C50C-C61C  2_556  1_555  1_555  2_556      # 4680 Check
PLAT723_ALERT_1_C Torsion Calc       69.00, Rep      71(69) Dev...        2.00 Sigma
              C62C-C49C-C50C-ND2C  2_556  1_555  1_555  1_555      # 4689 Check
```

```
PLAT723_ALERT_1_C Torsion Calc       59.00, Rep      62(69) Dev...       3.00 Sigma
              C62C-C49C-C50C-ND1C  2_556  1_555  1_555  2_556      # 4697 Check
PLAT723_ALERT_1_C Torsion Calc       85.00, Rep      88(69) Dev...       3.00 Sigma
              C62C-C49C-C50C-ND3C  2_556  1_555  1_555  1_555      # 4705 Check
PLAT723_ALERT_1_C Torsion Calc       98.00, Rep     101(69) Dev...       3.00 Sigma
              C62C-C49C-C50C-ND4C  2_556  1_555  1_555  2_556      # 4714 Check
PLAT723_ALERT_1_C Torsion Calc     -167.00, Rep    -169(**) Dev...       2.00 Sigma
              C77C-C78C-C79C-C69C  1_555  1_555  1_555  2_556      # 8524 Check
PLAT723_ALERT_1_C Torsion Calc      140.00, Rep     138(30) Dev...       2.00 Sigma
              ND4C-C78C-C79C-C69C  2_556  1_555  1_555  2_556      # 8528 Check
PLAT723_ALERT_1_C Torsion Calc      -49.00, Rep     -47(30) Dev...       2.00 Sigma
              C69C-C78C-C79C-C21C  2_556  1_555  1_555  1_555      # 8537 Check
PLAT723_ALERT_1_C Torsion Calc      -57.00, Rep     -55(30) Dev...       2.00 Sigma
              C69C-C78C-C79C-C26C  2_556  1_555  1_555  2_556      # 8545 Check
PLAT723_ALERT_1_C Torsion Calc      173.00, Rep     175(**) Dev...       2.00 Sigma
              C69C-C78C-C79C-C67C  2_556  1_555  1_555  2_556      # 8553 Check
PLAT723_ALERT_1_C Torsion Calc     -140.00, Rep    -138(30) Dev...       2.00 Sigma
              C69C-C78C-C79C-ND4C  2_556  1_555  1_555  2_556      # 8575 Check
PLAT723_ALERT_1_C Torsion Calc      157.00, Rep     142(**) Dev...      15.00 Sigma
              C58B-C34B-C35B-C21B  2_656  1_555  1_555  2_656      #11367 Check
PLAT723_ALERT_1_C Torsion Calc       55.00, Rep      40(**) Dev...      15.00 Sigma
              C59B-C34B-C35B-C21B  2_656  1_555  1_555  2_656      #11368 Check
PLAT723_ALERT_1_C Torsion Calc       -9.00, Rep     -23(**) Dev...      14.00 Sigma
              C39B-C34B-C35B-C21B  1_555  1_555  1_555  2_656      #11369 Check
PLAT723_ALERT_1_C Torsion Calc      141.00, Rep     127(**) Dev...      14.00 Sigma
              C33B-C34B-C35B-C21B  1_555  1_555  1_555  2_656      #11370 Check
PLAT723_ALERT_1_C Torsion Calc      -16.00, Rep     -31(**) Dev...      15.00 Sigma
              C20B-C34B-C35B-C21B  2_656  1_555  1_555  2_656      #11371 Check
PLAT723_ALERT_1_C Torsion Calc       49.00, Rep      34(**) Dev...      15.00 Sigma
              ND1B-C34B-C35B-C21B  1_555  1_555  1_555  2_656      #11372 Check
PLAT723_ALERT_1_C Torsion Calc       45.00, Rep      30(**) Dev...      15.00 Sigma
              ND2B-C34B-C35B-C21B  2_656  1_555  1_555  2_656      #11373 Check
PLAT723_ALERT_1_C Torsion Calc     -130.00, Rep    -115(**) Dev...      15.00 Sigma
              C21B-C34B-C35B-C22B  2_656  1_555  1_555  2_656      #11375 Check
PLAT723_ALERT_1_C Torsion Calc     -138.00, Rep    -124(**) Dev...      14.00 Sigma
              C21B-C34B-C35B-C31B  2_656  1_555  1_555  1_555      #11383 Check
PLAT723_ALERT_1_C Torsion Calc        3.00, Rep      18(**) Dev...      15.00 Sigma
              C21B-C34B-C35B-C36B  2_656  1_555  1_555  1_555      #11391 Check
PLAT723_ALERT_1_C Torsion Calc       16.00, Rep      31(**) Dev...      15.00 Sigma
              C21B-C34B-C35B-C20B  2_656  1_555  1_555  2_656      #11399 Check
PLAT723_ALERT_1_C Torsion Calc     -157.00, Rep    -142(**) Dev...      15.00 Sigma
              C21B-C34B-C35B-C58B  2_656  1_555  1_555  2_656      #11405 Check
PLAT723_ALERT_1_C Torsion Calc      -49.00, Rep     -34(**) Dev...      15.00 Sigma
              C21B-C34B-C35B-ND1B  2_656  1_555  1_555  1_555      #11413 Check
PLAT723_ALERT_1_C Torsion Calc      -45.00, Rep     -30(**) Dev...      15.00 Sigma
              C21B-C34B-C35B-ND2B  2_656  1_555  1_555  2_656      #11420 Check
PLAT723_ALERT_1_C Torsion Calc      -51.00, Rep     -46(**) Dev...       5.00 Sigma
              C76B-C51B-C56B-C77B  2_656  1_555  1_555  2_656      #13205 Check
PLAT723_ALERT_1_C Torsion Calc       22.00, Rep      17(**) Dev...       5.00 Sigma
              C75B-C51B-C56B-C76B  2_656  1_555  1_555  2_656      #13213 Check
PLAT723_ALERT_1_C Torsion Calc      173.00, Rep     167(**) Dev...       6.00 Sigma
              C26B-C51B-C56B-C76B  2_656  1_555  1_555  2_656      #13214 Check
PLAT723_ALERT_1_C Torsion Calc      174.00, Rep     168(**) Dev...       6.00 Sigma
              C50B-C51B-C56B-C76B  1_555  1_555  1_555  2_656      #13215 Check
PLAT723_ALERT_1_C Torsion Calc       19.00, Rep      13(**) Dev...       6.00 Sigma
              C52B-C51B-C56B-C76B  1_555  1_555  1_555  2_656      #13216 Check
PLAT723_ALERT_1_C Torsion Calc       51.00, Rep      46(**) Dev...       5.00 Sigma
```

```
             C77B-C51B-C56B-C76B  2_656  1_555  1_555  2_656      #13217 Check
PLAT723_ALERT_1_C Torsion Calc       75.00, Rep      69(**) Dev...       6.00 Sigma
             C74B-C51B-C56B-C76B  2_656  1_555  1_555  2_656      #13218 Check
PLAT723_ALERT_1_C Torsion Calc       98.00, Rep      92(**) Dev...       6.00 Sigma
             ND3B-C51B-C56B-C76B  1_555  1_555  1_555  2_656      #13219 Check
PLAT723_ALERT_1_C Torsion Calc       86.00, Rep      80(**) Dev...       6.00 Sigma
             ND4B-C51B-C56B-C76B  2_656  1_555  1_555  2_656      #13220 Check
PLAT723_ALERT_1_C Torsion Calc     -166.00, Rep    -160(**) Dev...       6.00 Sigma
             C76B-C51B-C56B-C57B  2_656  1_555  1_555  1_555      #13221 Check
PLAT723_ALERT_1_C Torsion Calc      -27.00, Rep     -21(**) Dev...       6.00 Sigma
             C76B-C51B-C56B-C55B  2_656  1_555  1_555  1_555      #13230 Check
PLAT723_ALERT_1_C Torsion Calc     -143.00, Rep    -138(**) Dev...       5.00 Sigma
             C76B-C51B-C56B-C33B  2_656  1_555  1_555  2_656      #13239 Check
PLAT723_ALERT_1_C Torsion Calc      -29.00, Rep     -24(**) Dev...       5.00 Sigma
             C76B-C51B-C56B-C78B  2_656  1_555  1_555  2_656      #13248 Check
PLAT723_ALERT_1_C Torsion Calc      -98.00, Rep     -92(**) Dev...       6.00 Sigma
             C76B-C51B-C56B-ND3B  2_656  1_555  1_555  1_555      #13257 Check
PLAT906_ALERT_3_C Large K Value in the Analysis of Variance ......      8.761 Check
PLAT906_ALERT_3_C Large K Value in the Analysis of Variance ......      2.384 Check
PLAT911_ALERT_3_C Missing FCF Refl Between Thmin & STh/L=    0.600        481 Report
PLAT913_ALERT_3_C Missing # of Very Strong Reflections in FCF ....         17 Note
PLAT918_ALERT_3_C Reflection(s) with I(obs) much Smaller I(calc) .          2 Check
PLAT971_ALERT_2_C Check Calcd Resid. Dens.  0.93Ang From Nd3A           2.22 eA-3
PLAT971_ALERT_2_C Check Calcd Resid. Dens.  0.27Ang From Nd3A           2.12 eA-3
PLAT971_ALERT_2_C Check Calcd Resid. Dens.  0.85Ang From C34C           1.66 eA-3
PLAT971_ALERT_2_C Check Calcd Resid. Dens.  0.54Ang From C11B           1.63 eA-3
PLAT972_ALERT_2_C Check Calcd Resid. Dens.  0.94Ang From Nd3A          -2.45 eA-3
PLAT972_ALERT_2_C Check Calcd Resid. Dens.  0.67Ang From Nd4A          -1.77 eA-3
PLAT972_ALERT_2_C Check Calcd Resid. Dens.  0.68Ang From Nd2A          -1.55 eA-3
PLAT974_ALERT_2_C Check Calcd Negative Resid. Density on      Nd1A      -1.29 eA-3
PLAT974_ALERT_2_C Check Calcd Negative Resid. Density on      Nd2A      -1.19 eA-3
PLAT977_ALERT_2_C Check Negative Difference Density on H10T      .      -0.43 eA-3
PLAT977_ALERT_2_C Check Negative Difference Density on H12S      .      -0.36 eA-3
PLAT977_ALERT_2_C Check Negative Difference Density on H13Z      .      -0.45 eA-3
PLAT977_ALERT_2_C Check Negative Difference Density on H20S      .      -0.56 eA-3
PLAT977_ALERT_2_C Check Negative Difference Density on H35B      .      -0.36 eA-3
PLAT977_ALERT_2_C Check Negative Difference Density on H71B      .      -0.36 eA-3
PLAT977_ALERT_2_C Check Negative Difference Density on H6S       .      -0.37 eA-3
PLAT977_ALERT_2_C Check Negative Difference Density on H24S      .      -0.66 eA-3
PLAT977_ALERT_2_C Check Negative Difference Density on H202      .      -0.64 eA-3
```

Alert level G

```
FORMU01_ALERT_1_G  There is a discrepancy between the atom counts in the
            _chemical_formula_sum and _chemical_formula_moiety. This is
            usually due to the moiety formula being in the wrong format.
            Atom count from _chemical_formula_sum:   C319.63 H193.11 F6 N16 Nd4 Ni
            Atom count from _chemical_formula_moiety:
ABSMU01_ALERT_1_G  Calculation of _exptl_absorpt_correction_mu
                not performed for this radiation type.
CELLZ01_ALERT_1_G Difference between formula and atom_site contents detected.
CELLZ01_ALERT_1_G ALERT: check formula stoichiometry or atom site occupancies.
           From the CIF: _cell_formula_units_Z    8
           From the CIF: _chemical_formula_sum  C319.63 H193.11 F6 N16 Nd4 Ni4
           TEST: Compare cell contents of formula and atom_site data

           atom    Z*formula  cif sites diff
```

```
           C       2557.04   2557.07   -0.03
           H       1544.88   1544.93   -0.05
           F         48.00     48.00    0.00
           N        128.00    128.00    0.00
           Nd        32.00     32.00    0.00
           Ni        32.00     32.00    0.00
PLAT002_ALERT_2_G Number of Distance or Angle Restraints on AtSite        352 Note
PLAT003_ALERT_2_G Number of Uiso or Uij Restrained non-H Atoms ...        468 Report
PLAT042_ALERT_1_G Calc. and Reported Moiety Formula Strings Differ     Please Check
PLAT045_ALERT_1_G Calculated and Reported Z Differ by a Factor ...     0.5000 Check
PLAT083_ALERT_2_G SHELXL Second Parameter in WGHT  Unusually Large     353.85 Why ?
PLAT092_ALERT_4_G Check: Wavelength Given is not Cu,Ga,Mo,Ag,In Ka    0.79990 Ang.
PLAT174_ALERT_4_G The CIF-Embedded .res File Contains FLAT Records          5 Report
PLAT175_ALERT_4_G The CIF-Embedded .res File Contains SAME Records          5 Report
PLAT176_ALERT_4_G The CIF-Embedded .res File Contains SADI Records        138 Report
PLAT178_ALERT_4_G The CIF-Embedded .res File Contains SIMU Records          9 Report
PLAT180_ALERT_4_G Check Cell Rounding: # of Values Ending with 0 =          3 Note
PLAT186_ALERT_4_G The CIF-Embedded .res File Contains ISOR Records          6 Report
PLAT242_ALERT_2_G Low    'MainMol' Ueq as Compared to Neighbors of       C81A Check
PLAT300_ALERT_4_G Atom Site Occupancy of F4         Constrained at        0.5 Check
PLAT300_ALERT_4_G Atom Site Occupancy of F5         Constrained at        0.5 Check
PLAT300_ALERT_4_G Atom Site Occupancy of F6         Constrained at        0.5 Check
PLAT300_ALERT_4_G Atom Site Occupancy of C1B        Constrained at        0.5 Check
PLAT300_ALERT_4_G Atom Site Occupancy of C2B        Constrained at        0.5 Check
PLAT300_ALERT_4_G Atom Site Occupancy of C3B        Constrained at        0.5 Check
PLAT300_ALERT_4_G Atom Site Occupancy of C4B        Constrained at        0.5 Check
PLAT300_ALERT_4_G Atom Site Occupancy of C5B        Constrained at        0.5 Check
PLAT300_ALERT_4_G Atom Site Occupancy of C6B        Constrained at        0.5 Check
PLAT300_ALERT_4_G Atom Site Occupancy of C7B        Constrained at        0.5 Check
PLAT300_ALERT_4_G Atom Site Occupancy of C8B        Constrained at        0.5 Check
PLAT300_ALERT_4_G Atom Site Occupancy of C9B        Constrained at        0.5 Check
PLAT300_ALERT_4_G Atom Site Occupancy of C10B       Constrained at        0.5 Check
PLAT300_ALERT_4_G Atom Site Occupancy of C11B       Constrained at        0.5 Check
PLAT300_ALERT_4_G Atom Site Occupancy of C12B       Constrained at        0.5 Check
PLAT300_ALERT_4_G Atom Site Occupancy of C13B       Constrained at        0.5 Check
PLAT300_ALERT_4_G Atom Site Occupancy of C14B       Constrained at        0.5 Check
PLAT300_ALERT_4_G Atom Site Occupancy of C15B       Constrained at        0.5 Check
PLAT300_ALERT_4_G Atom Site Occupancy of C16B       Constrained at        0.5 Check
PLAT300_ALERT_4_G Atom Site Occupancy of C17B       Constrained at        0.5 Check
PLAT300_ALERT_4_G Atom Site Occupancy of C18B       Constrained at        0.5 Check
PLAT300_ALERT_4_G Atom Site Occupancy of C19B       Constrained at        0.5 Check
PLAT300_ALERT_4_G Atom Site Occupancy of C20B       Constrained at        0.5 Check
PLAT300_ALERT_4_G Atom Site Occupancy of C21B       Constrained at        0.5 Check
PLAT300_ALERT_4_G Atom Site Occupancy of C22B       Constrained at        0.5 Check
PLAT300_ALERT_4_G Atom Site Occupancy of C23B       Constrained at        0.5 Check
PLAT300_ALERT_4_G Atom Site Occupancy of C24B       Constrained at        0.5 Check
PLAT300_ALERT_4_G Atom Site Occupancy of C25B       Constrained at        0.5 Check
PLAT300_ALERT_4_G Atom Site Occupancy of C26B       Constrained at        0.5 Check
PLAT300_ALERT_4_G Atom Site Occupancy of C27B       Constrained at        0.5 Check
PLAT300_ALERT_4_G Atom Site Occupancy of C28B       Constrained at        0.5 Check
PLAT300_ALERT_4_G Atom Site Occupancy of C29B       Constrained at        0.5 Check
PLAT300_ALERT_4_G Atom Site Occupancy of C30B       Constrained at        0.5 Check
PLAT300_ALERT_4_G Atom Site Occupancy of C31B       Constrained at        0.5 Check
PLAT300_ALERT_4_G Atom Site Occupancy of C32B       Constrained at        0.5 Check
PLAT300_ALERT_4_G Atom Site Occupancy of C33B       Constrained at        0.5 Check
PLAT300_ALERT_4_G Atom Site Occupancy of C34B       Constrained at        0.5 Check
PLAT300_ALERT_4_G Atom Site Occupancy of C35B       Constrained at        0.5 Check
```

```
PLAT300_ALERT_4_G Atom Site Occupancy of C36B       Constrained at        0.5 Check
PLAT300_ALERT_4_G Atom Site Occupancy of C37B       Constrained at        0.5 Check
PLAT300_ALERT_4_G Atom Site Occupancy of C38B       Constrained at        0.5 Check
PLAT300_ALERT_4_G Atom Site Occupancy of C39B       Constrained at        0.5 Check
PLAT300_ALERT_4_G Atom Site Occupancy of C40B       Constrained at        0.5 Check
PLAT300_ALERT_4_G Atom Site Occupancy of C41B       Constrained at        0.5 Check
PLAT300_ALERT_4_G Atom Site Occupancy of C42B       Constrained at        0.5 Check
PLAT300_ALERT_4_G Atom Site Occupancy of C43B       Constrained at        0.5 Check
PLAT300_ALERT_4_G Atom Site Occupancy of C44B       Constrained at        0.5 Check
PLAT300_ALERT_4_G Atom Site Occupancy of C45B       Constrained at        0.5 Check
PLAT300_ALERT_4_G Atom Site Occupancy of C46B       Constrained at        0.5 Check
PLAT300_ALERT_4_G Atom Site Occupancy of C47B       Constrained at        0.5 Check
PLAT300_ALERT_4_G Atom Site Occupancy of C48B       Constrained at        0.5 Check
PLAT300_ALERT_4_G Atom Site Occupancy of C49B       Constrained at        0.5 Check
PLAT300_ALERT_4_G Atom Site Occupancy of C50B       Constrained at        0.5 Check
PLAT300_ALERT_4_G Atom Site Occupancy of C51B       Constrained at        0.5 Check
PLAT300_ALERT_4_G Atom Site Occupancy of C52B       Constrained at        0.5 Check
PLAT300_ALERT_4_G Atom Site Occupancy of C53B       Constrained at        0.5 Check
PLAT300_ALERT_4_G Atom Site Occupancy of C54B       Constrained at        0.5 Check
PLAT300_ALERT_4_G Atom Site Occupancy of C55B       Constrained at        0.5 Check
PLAT300_ALERT_4_G Atom Site Occupancy of C56B       Constrained at        0.5 Check
PLAT300_ALERT_4_G Atom Site Occupancy of C57B       Constrained at        0.5 Check
PLAT300_ALERT_4_G Atom Site Occupancy of C58B       Constrained at        0.5 Check
PLAT300_ALERT_4_G Atom Site Occupancy of C59B       Constrained at        0.5 Check
PLAT300_ALERT_4_G Atom Site Occupancy of C60B       Constrained at        0.5 Check
PLAT300_ALERT_4_G Atom Site Occupancy of C61B       Constrained at        0.5 Check
PLAT300_ALERT_4_G Atom Site Occupancy of C62B       Constrained at        0.5 Check
PLAT300_ALERT_4_G Atom Site Occupancy of C63B       Constrained at        0.5 Check
PLAT300_ALERT_4_G Atom Site Occupancy of C64B       Constrained at        0.5 Check
PLAT300_ALERT_4_G Atom Site Occupancy of C65B       Constrained at        0.5 Check
PLAT300_ALERT_4_G Atom Site Occupancy of C66B       Constrained at        0.5 Check
PLAT300_ALERT_4_G Atom Site Occupancy of C67B       Constrained at        0.5 Check
PLAT300_ALERT_4_G Atom Site Occupancy of C68B       Constrained at        0.5 Check
PLAT300_ALERT_4_G Atom Site Occupancy of C69B       Constrained at        0.5 Check
PLAT300_ALERT_4_G Atom Site Occupancy of C70B       Constrained at        0.5 Check
PLAT300_ALERT_4_G Atom Site Occupancy of C71B       Constrained at        0.5 Check
PLAT300_ALERT_4_G Atom Site Occupancy of C72B       Constrained at        0.5 Check
PLAT300_ALERT_4_G Atom Site Occupancy of C73B       Constrained at        0.5 Check
PLAT300_ALERT_4_G Atom Site Occupancy of C74B       Constrained at        0.5 Check
PLAT300_ALERT_4_G Atom Site Occupancy of C75B       Constrained at        0.5 Check
PLAT300_ALERT_4_G Atom Site Occupancy of C76B       Constrained at        0.5 Check
PLAT300_ALERT_4_G Atom Site Occupancy of C77B       Constrained at        0.5 Check
PLAT300_ALERT_4_G Atom Site Occupancy of C78B       Constrained at        0.5 Check
PLAT300_ALERT_4_G Atom Site Occupancy of C79B       Constrained at        0.5 Check
PLAT300_ALERT_4_G Atom Site Occupancy of C80B       Constrained at        0.5 Check
PLAT300_ALERT_4_G Atom Site Occupancy of C81B       Constrained at        0.5 Check
PLAT300_ALERT_4_G Atom Site Occupancy of F7         Constrained at        0.5 Check
PLAT300_ALERT_4_G Atom Site Occupancy of F8         Constrained at        0.5 Check
PLAT300_ALERT_4_G Atom Site Occupancy of F9         Constrained at        0.5 Check
PLAT300_ALERT_4_G Atom Site Occupancy of C1C        Constrained at        0.5 Check
PLAT300_ALERT_4_G Atom Site Occupancy of C2C        Constrained at        0.5 Check
PLAT300_ALERT_4_G Atom Site Occupancy of C3C        Constrained at        0.5 Check
PLAT300_ALERT_4_G Atom Site Occupancy of C4C        Constrained at        0.5 Check
PLAT300_ALERT_4_G Atom Site Occupancy of C5C        Constrained at        0.5 Check
PLAT300_ALERT_4_G Atom Site Occupancy of C6C        Constrained at        0.5 Check
PLAT300_ALERT_4_G Atom Site Occupancy of C7C        Constrained at        0.5 Check
PLAT300_ALERT_4_G Atom Site Occupancy of C8C        Constrained at        0.5 Check
```

```
PLAT300_ALERT_4_G Atom Site Occupancy of C9C        Constrained at        0.5 Check
PLAT300_ALERT_4_G Atom Site Occupancy of C10C       Constrained at        0.5 Check
PLAT300_ALERT_4_G Atom Site Occupancy of C11C       Constrained at        0.5 Check
PLAT300_ALERT_4_G Atom Site Occupancy of C12C       Constrained at        0.5 Check
PLAT300_ALERT_4_G Atom Site Occupancy of C13C       Constrained at        0.5 Check
PLAT300_ALERT_4_G Atom Site Occupancy of C14C       Constrained at        0.5 Check
PLAT300_ALERT_4_G Atom Site Occupancy of C15C       Constrained at        0.5 Check
PLAT300_ALERT_4_G Atom Site Occupancy of C16C       Constrained at        0.5 Check
PLAT300_ALERT_4_G Atom Site Occupancy of C17C       Constrained at        0.5 Check
PLAT300_ALERT_4_G Atom Site Occupancy of C18C       Constrained at        0.5 Check
PLAT300_ALERT_4_G Atom Site Occupancy of C19C       Constrained at        0.5 Check
PLAT300_ALERT_4_G Atom Site Occupancy of C20C       Constrained at        0.5 Check
PLAT300_ALERT_4_G Atom Site Occupancy of C21C       Constrained at        0.5 Check
PLAT300_ALERT_4_G Atom Site Occupancy of C22C       Constrained at        0.5 Check
PLAT300_ALERT_4_G Atom Site Occupancy of C23C       Constrained at        0.5 Check
PLAT300_ALERT_4_G Atom Site Occupancy of C24C       Constrained at        0.5 Check
PLAT300_ALERT_4_G Atom Site Occupancy of C25C       Constrained at        0.5 Check
PLAT300_ALERT_4_G Atom Site Occupancy of C26C       Constrained at        0.5 Check
PLAT300_ALERT_4_G Atom Site Occupancy of C27C       Constrained at        0.5 Check
PLAT300_ALERT_4_G Atom Site Occupancy of C28C       Constrained at        0.5 Check
PLAT300_ALERT_4_G Atom Site Occupancy of C29C       Constrained at        0.5 Check
PLAT300_ALERT_4_G Atom Site Occupancy of C30C       Constrained at        0.5 Check
PLAT300_ALERT_4_G Atom Site Occupancy of C31C       Constrained at        0.5 Check
PLAT300_ALERT_4_G Atom Site Occupancy of C32C       Constrained at        0.5 Check
PLAT300_ALERT_4_G Atom Site Occupancy of C33C       Constrained at        0.5 Check
PLAT300_ALERT_4_G Atom Site Occupancy of C34C       Constrained at        0.5 Check
PLAT300_ALERT_4_G Atom Site Occupancy of C35C       Constrained at        0.5 Check
PLAT300_ALERT_4_G Atom Site Occupancy of C36C       Constrained at        0.5 Check
PLAT300_ALERT_4_G Atom Site Occupancy of C37C       Constrained at        0.5 Check
PLAT300_ALERT_4_G Atom Site Occupancy of C38C       Constrained at        0.5 Check
PLAT300_ALERT_4_G Atom Site Occupancy of C39C       Constrained at        0.5 Check
PLAT300_ALERT_4_G Atom Site Occupancy of C40C       Constrained at        0.5 Check
PLAT300_ALERT_4_G Atom Site Occupancy of C41C       Constrained at        0.5 Check
PLAT300_ALERT_4_G Atom Site Occupancy of C42C       Constrained at        0.5 Check
PLAT300_ALERT_4_G Atom Site Occupancy of C43C       Constrained at        0.5 Check
PLAT300_ALERT_4_G Atom Site Occupancy of C44C       Constrained at        0.5 Check
PLAT300_ALERT_4_G Atom Site Occupancy of C45C       Constrained at        0.5 Check
PLAT300_ALERT_4_G Atom Site Occupancy of C46C       Constrained at        0.5 Check
PLAT300_ALERT_4_G Atom Site Occupancy of C47C       Constrained at        0.5 Check
PLAT300_ALERT_4_G Atom Site Occupancy of C48C       Constrained at        0.5 Check
PLAT300_ALERT_4_G Atom Site Occupancy of C49C       Constrained at        0.5 Check
PLAT300_ALERT_4_G Atom Site Occupancy of C50C       Constrained at        0.5 Check
PLAT300_ALERT_4_G Atom Site Occupancy of C51C       Constrained at        0.5 Check
PLAT300_ALERT_4_G Atom Site Occupancy of C52C       Constrained at        0.5 Check
PLAT300_ALERT_4_G Atom Site Occupancy of C53C       Constrained at        0.5 Check
PLAT300_ALERT_4_G Atom Site Occupancy of C54C       Constrained at        0.5 Check
PLAT300_ALERT_4_G Atom Site Occupancy of C55C       Constrained at        0.5 Check
PLAT300_ALERT_4_G Atom Site Occupancy of C56C       Constrained at        0.5 Check
PLAT300_ALERT_4_G Atom Site Occupancy of C57C       Constrained at        0.5 Check
PLAT300_ALERT_4_G Atom Site Occupancy of C58C       Constrained at        0.5 Check
PLAT300_ALERT_4_G Atom Site Occupancy of C59C       Constrained at        0.5 Check
PLAT300_ALERT_4_G Atom Site Occupancy of C60C       Constrained at        0.5 Check
PLAT300_ALERT_4_G Atom Site Occupancy of C61C       Constrained at        0.5 Check
PLAT300_ALERT_4_G Atom Site Occupancy of C62C       Constrained at        0.5 Check
PLAT300_ALERT_4_G Atom Site Occupancy of C63C       Constrained at        0.5 Check
PLAT300_ALERT_4_G Atom Site Occupancy of C64C       Constrained at        0.5 Check
PLAT300_ALERT_4_G Atom Site Occupancy of C65C       Constrained at        0.5 Check
```

```
PLAT300_ALERT_4_G Atom Site Occupancy of C66C       Constrained at        0.5 Check
PLAT300_ALERT_4_G Atom Site Occupancy of C67C       Constrained at        0.5 Check
PLAT300_ALERT_4_G Atom Site Occupancy of C68C       Constrained at        0.5 Check
PLAT300_ALERT_4_G Atom Site Occupancy of C69C       Constrained at        0.5 Check
PLAT300_ALERT_4_G Atom Site Occupancy of C70C       Constrained at        0.5 Check
PLAT300_ALERT_4_G Atom Site Occupancy of C71C       Constrained at        0.5 Check
PLAT300_ALERT_4_G Atom Site Occupancy of C72C       Constrained at        0.5 Check
PLAT300_ALERT_4_G Atom Site Occupancy of C73C       Constrained at        0.5 Check
PLAT300_ALERT_4_G Atom Site Occupancy of C74C       Constrained at        0.5 Check
PLAT300_ALERT_4_G Atom Site Occupancy of C75C       Constrained at        0.5 Check
PLAT300_ALERT_4_G Atom Site Occupancy of C76C       Constrained at        0.5 Check
PLAT300_ALERT_4_G Atom Site Occupancy of C77C       Constrained at        0.5 Check
PLAT300_ALERT_4_G Atom Site Occupancy of C78C       Constrained at        0.5 Check
PLAT300_ALERT_4_G Atom Site Occupancy of C79C       Constrained at        0.5 Check
PLAT300_ALERT_4_G Atom Site Occupancy of C80C       Constrained at        0.5 Check
PLAT300_ALERT_4_G Atom Site Occupancy of C81C       Constrained at        0.5 Check
PLAT301_ALERT_3_G Main Residue  Disorder .............(Resd  1  )          2% Note
PLAT302_ALERT_4_G Anion/Solvent/Minor-Residue Disorder (Resd  2  )        100% Note
PLAT302_ALERT_4_G Anion/Solvent/Minor-Residue Disorder (Resd  3  )        100% Note
PLAT302_ALERT_4_G Anion/Solvent/Minor-Residue Disorder (Resd  4  )         61% Note
PLAT302_ALERT_4_G Anion/Solvent/Minor-Residue Disorder (Resd  5  )         61% Note
PLAT302_ALERT_4_G Anion/Solvent/Minor-Residue Disorder (Resd  6  )         61% Note
PLAT302_ALERT_4_G Anion/Solvent/Minor-Residue Disorder (Resd  7  )         59% Note
PLAT302_ALERT_4_G Anion/Solvent/Minor-Residue Disorder (Resd  8  )        100% Note
PLAT302_ALERT_4_G Anion/Solvent/Minor-Residue Disorder (Resd  9  )        100% Note
PLAT302_ALERT_4_G Anion/Solvent/Minor-Residue Disorder (Resd 10  )        100% Note
PLAT302_ALERT_4_G Anion/Solvent/Minor-Residue Disorder (Resd 11  )        100% Note
PLAT302_ALERT_4_G Anion/Solvent/Minor-Residue Disorder (Resd 12  )        100% Note
PLAT302_ALERT_4_G Anion/Solvent/Minor-Residue Disorder (Resd 13  )        100% Note
PLAT302_ALERT_4_G Anion/Solvent/Minor-Residue Disorder (Resd 14  )        100% Note
PLAT302_ALERT_4_G Anion/Solvent/Minor-Residue Disorder (Resd 15  )        100% Note
PLAT304_ALERT_4_G Non-Integer Number of Atoms in ..... (Resd  4  )      85.48 Check
PLAT304_ALERT_4_G Non-Integer Number of Atoms in ..... (Resd  5  )      85.50 Check
PLAT304_ALERT_4_G Non-Integer Number of Atoms in ..... (Resd  6  )      85.42 Check
PLAT304_ALERT_4_G Non-Integer Number of Atoms in ..... (Resd  7  )      85.46 Check
PLAT304_ALERT_4_G Non-Integer Number of Atoms in ..... (Resd  8  )       9.49 Check
PLAT304_ALERT_4_G Non-Integer Number of Atoms in ..... (Resd  9  )       8.31 Check
PLAT304_ALERT_4_G Non-Integer Number of Atoms in ..... (Resd 10  )       6.69 Check
PLAT304_ALERT_4_G Non-Integer Number of Atoms in ..... (Resd 11  )       4.40 Check
PLAT304_ALERT_4_G Non-Integer Number of Atoms in ..... (Resd 12  )       1.44 Check
PLAT304_ALERT_4_G Non-Integer Number of Atoms in ..... (Resd 13  )       1.44 Check
PLAT304_ALERT_4_G Non-Integer Number of Atoms in ..... (Resd 14  )       0.56 Check
PLAT304_ALERT_4_G Non-Integer Number of Atoms in ..... (Resd 15  )       0.56 Check
PLAT315_ALERT_2_G Singly Bonded Carbon Detected (H-atoms Missing).       C321 Check
PLAT333_ALERT_2_G Large Aver C6-Ring C-C Dist C4A      -C9A      .       1.42 Ang.
PLAT333_ALERT_2_G Large Aver C6-Ring C-C Dist C6A      -C67A     .       1.42 Ang.
PLAT333_ALERT_2_G Large Aver C6-Ring C-C Dist C14A     -C66A     .       1.42 Ang.
PLAT333_ALERT_2_G Large Aver C6-Ring C-C Dist C15A     -C59A     .       1.43 Ang.
PLAT333_ALERT_2_G Large Aver C6-Ring C-C Dist C19A     -C24A     .       1.42 Ang.
PLAT333_ALERT_2_G Large Aver C6-Ring C-C Dist C4B      -C45B     .       1.44 Ang.
PLAT333_ALERT_2_G Large Aver C6-Ring C-C Dist C6B      -C79B     .       1.44 Ang.
PLAT333_ALERT_2_G Large Aver C6-Ring C-C Dist C14B     -C80B     .       1.43 Ang.
PLAT333_ALERT_2_G Large Aver C6-Ring C-C Dist C18B     -C73B     .       1.42 Ang.
PLAT333_ALERT_2_G Large Aver C6-Ring C-C Dist C3C      -C58C     .       1.43 Ang.
PLAT333_ALERT_2_G Large Aver C6-Ring C-C Dist C12C     -C17C     .       1.43 Ang.
PLAT333_ALERT_2_G Large Aver C6-Ring C-C Dist C15C     -C78C     .       1.43 Ang.
PLAT333_ALERT_2_G Large Aver C6-Ring C-C Dist C19C     -C24C     .       1.43 Ang.
```

```
PLAT410_ALERT_2_G Short Intra H...H Contact  H21A     ..H202      .       1.71 Ang.
                                                      x,y,z  =     1_555 Check
PLAT410_ALERT_2_G Short Intra H...H Contact  H21B     ..H202      .       1.72 Ang.
                                                      x,y,z  =     1_555 Check
PLAT410_ALERT_2_G Short Intra H...H Contact  H23A     ..H203      .       1.22 Ang.
                                                      x,y,z  =     1_555 Check
PLAT410_ALERT_2_G Short Intra H...H Contact  H23B     ..H203      .       1.71 Ang.
                                                      x,y,z  =     1_555 Check
PLAT410_ALERT_2_G Short Intra H...H Contact  H25A     ..H207      .       1.12 Ang.
                                                      x,y,z  =     1_555 Check
PLAT410_ALERT_2_G Short Intra H...H Contact  H25B     ..H205      .       1.84 Ang.
                                                      x,y,z  =     1_555 Check
PLAT410_ALERT_2_G Short Intra H...H Contact  H25B     ..H207      .       1.73 Ang.
                                                      x,y,z  =     1_555 Check
PLAT410_ALERT_2_G Short Intra H...H Contact  H27A     ..H208      .       1.39 Ang.
                                                      x,y,z  =     1_555 Check
PLAT410_ALERT_2_G Short Intra H...H Contact  H27B     ..H208      .       1.33 Ang.
                                                      x,y,z  =     1_555 Check
PLAT410_ALERT_2_G Short Intra H...H Contact  H29A     ..H212      .       1.12 Ang.
                                                      x,y,z  =     1_555 Check
PLAT410_ALERT_2_G Short Intra H...H Contact  H29B     ..H212      .       1.17 Ang.
                                                      x,y,z  =     1_555 Check
PLAT410_ALERT_2_G Short Intra H...H Contact  H31A     ..H213      .       1.67 Ang.
                                                      x,y,z  =     1_555 Check
PLAT410_ALERT_2_G Short Intra H...H Contact  H31A     ..H215      .       1.73 Ang.
                                                      x,y,z  =     1_555 Check
PLAT410_ALERT_2_G Short Intra H...H Contact  H31B     ..H213      .       0.92 Ang.
                                                      x,y,z  =     1_555 Check
PLAT410_ALERT_2_G Short Intra H...H Contact  H33A     ..H217      .       1.70 Ang.
                                                      x,y,z  =     1_555 Check
PLAT410_ALERT_2_G Short Intra H...H Contact  H33B     ..H217      .       0.97 Ang.
                                                      x,y,z  =     1_555 Check
PLAT410_ALERT_2_G Short Intra H...H Contact  H35A     ..H218      .       1.77 Ang.
                                                      x,y,z  =     1_555 Check
PLAT410_ALERT_2_G Short Intra H...H Contact  H35B     ..H218      .       1.63 Ang.
                                                      x,y,z  =     1_555 Check
PLAT410_ALERT_2_G Short Intra H...H Contact  H57A     ..H238      .       0.87 Ang.
                                                      x,y,z  =     1_555 Check
PLAT410_ALERT_2_G Short Intra H...H Contact  H57B     ..H238      .       1.70 Ang.
                                                      x,y,z  =     1_555 Check
PLAT410_ALERT_2_G Short Intra H...H Contact  H57B     ..H256      .       1.65 Ang.
                                                      x,y,z  =     1_555 Check
PLAT410_ALERT_2_G Short Intra H...H Contact  H59A     ..H239      .       1.03 Ang.
                                                      x,y,z  =     1_555 Check
PLAT410_ALERT_2_G Short Intra H...H Contact  H59B     ..H238      .       2.12 Ang.
                                                      x,y,z  =     1_555 Check
PLAT410_ALERT_2_G Short Intra H...H Contact  H59B     ..H239      .       1.05 Ang.
                                                      x,y,z  =     1_555 Check
PLAT410_ALERT_2_G Short Intra H...H Contact  H61A     ..H243      .       1.20 Ang.
                                                      x,y,z  =     1_555 Check
PLAT410_ALERT_2_G Short Intra H...H Contact  H61B     ..H243      .       1.26 Ang.
                                                      x,y,z  =     1_555 Check
PLAT410_ALERT_2_G Short Intra H...H Contact  H63A     ..H244      .       1.71 Ang.
                                                      x,y,z  =     1_555 Check
PLAT410_ALERT_2_G Short Intra H...H Contact  H63A     ..H246      .       1.91 Ang.
                                                      x,y,z  =     1_555 Check
PLAT410_ALERT_2_G Short Intra H...H Contact  H63B     ..H244      .       1.04 Ang.
```

```
                                                      x,y,z  =      1_555 Check
PLAT410_ALERT_2_G Short Intra H...H Contact  H65A     ..H248      .       1.74 Ang.
                                                      x,y,z  =      1_555 Check
PLAT410_ALERT_2_G Short Intra H...H Contact  H65B     ..H248      .       1.21 Ang.
                                                      x,y,z  =      1_555 Check
PLAT410_ALERT_2_G Short Intra H...H Contact  H67A     ..H249      .       1.66 Ang.
                                                      x,y,z  =      1_555 Check
PLAT410_ALERT_2_G Short Intra H...H Contact  H67B     ..H249      .       1.77 Ang.
                                                      x,y,z  =      1_555 Check
PLAT410_ALERT_2_G Short Intra H...H Contact  H69A     ..H253      .       1.64 Ang.
                                                      x,y,z  =      1_555 Check
PLAT410_ALERT_2_G Short Intra H...H Contact  H69B     ..H253      .       1.77 Ang.
                                                      x,y,z  =      1_555 Check
PLAT410_ALERT_2_G Short Intra H...H Contact  H71A     ..H254      .       0.97 Ang.
                                                      x,y,z  =      1_555 Check
PLAT410_ALERT_2_G Short Intra H...H Contact  H71B     ..H254      .       1.68 Ang.
                                                      x,y,z  =      1_555 Check
PLAT410_ALERT_2_G Short Intra H...H Contact  H10D     ..H282      .       1.96 Ang.
                                                      x,y,z  =      1_555 Check
PLAT410_ALERT_2_G Short Intra H...H Contact  H10D     ..H284      .       1.09 Ang.
                                                      x,y,z  =      1_555 Check
PLAT410_ALERT_2_G Short Intra H...H Contact  H10E     ..H284      .       0.94 Ang.
                                                      x,y,z  =      1_555 Check
PLAT410_ALERT_2_G Short Intra H...H Contact  H10I     ..H285      .       0.88 Ang.
                                                      x,y,z  =      1_555 Check
PLAT410_ALERT_2_G Short Intra H...H Contact  H10J     ..H285      .       1.62 Ang.
                                                      x,y,z  =      1_555 Check
PLAT410_ALERT_2_G Short Intra H...H Contact  H10J     ..H287      .       1.79 Ang.
                                                      x,y,z  =      1_555 Check
PLAT410_ALERT_2_G Short Intra H...H Contact  H10N     ..H289      .       1.09 Ang.
                                                      x,y,z  =      1_555 Check
PLAT410_ALERT_2_G Short Intra H...H Contact  H10O     ..H289      .       1.69 Ang.
                                                      x,y,z  =      1_555 Check
PLAT410_ALERT_2_G Short Intra H...H Contact  H10S     ..H290      .       1.67 Ang.
                                                      x,y,z  =      1_555 Check
PLAT410_ALERT_2_G Short Intra H...H Contact  H10T     ..H290      .       1.76 Ang.
                                                      x,y,z  =      1_555 Check
PLAT410_ALERT_2_G Short Intra H...H Contact  H93A     ..H274      .       1.80 Ang.
                                                      x,y,z  =      1_555 Check
PLAT410_ALERT_2_G Short Intra H...H Contact  H93B     ..H274      .       1.71 Ang.
                                                      x,y,z  =      1_555 Check
PLAT410_ALERT_2_G Short Intra H...H Contact  H95A     ..H275      .       1.78 Ang.
                                                      x,y,z  =      1_555 Check
PLAT410_ALERT_2_G Short Intra H...H Contact  H95B     ..H275      .       1.15 Ang.
                                                      x,y,z  =      1_555 Check
PLAT410_ALERT_2_G Short Intra H...H Contact  H97A     ..H277      .       1.81 Ang.
                                                      x,y,z  =      1_555 Check
PLAT410_ALERT_2_G Short Intra H...H Contact  H97A     ..H279      .       1.81 Ang.
                                                      x,y,z  =      1_555 Check
PLAT410_ALERT_2_G Short Intra H...H Contact  H97B     ..H279      .       1.04 Ang.
                                                      x,y,z  =      1_555 Check
PLAT410_ALERT_2_G Short Intra H...H Contact  H99A     ..H280      .       1.24 Ang.
                                                      x,y,z  =      1_555 Check
PLAT410_ALERT_2_G Short Intra H...H Contact  H99B     ..H280      .       1.07 Ang.
                                                      x,y,z  =      1_555 Check
PLAT410_ALERT_2_G Short Intra H...H Contact  H12A     ..H310      .       1.21 Ang.
                                                      x,y,z  =      1_555 Check
```

```
PLAT410_ALERT_2_G Short Intra H...H Contact  H12B     ..H310     .       1.38 Ang.
                                                      x,y,z  =     1_555 Check
PLAT410_ALERT_2_G Short Intra H...H Contact  H12B     ..H328     .       1.99 Ang.
                                                      x,y,z  =     1_555 Check
PLAT410_ALERT_2_G Short Intra H...H Contact  H13D     ..H311     .       1.47 Ang.
                                                      x,y,z  =     1_555 Check
PLAT410_ALERT_2_G Short Intra H...H Contact  H13D     ..H313     .       1.93 Ang.
                                                      x,y,z  =     1_555 Check
PLAT410_ALERT_2_G Short Intra H...H Contact  H13E     ..H311     .       0.89 Ang.
                                                      x,y,z  =     1_555 Check
PLAT410_ALERT_2_G Short Intra H...H Contact  H13I     ..H315     .       1.58 Ang.
                                                      x,y,z  =     1_555 Check
PLAT410_ALERT_2_G Short Intra H...H Contact  H13J     ..H315     .       0.96 Ang.
                                                      x,y,z  =     1_555 Check
PLAT410_ALERT_2_G Short Intra H...H Contact  H13N     ..H316     .       1.69 Ang.
                                                      x,y,z  =     1_555 Check
PLAT410_ALERT_2_G Short Intra H...H Contact  H13O     ..H316     .       1.49 Ang.
                                                      x,y,z  =     1_555 Check
PLAT410_ALERT_2_G Short Intra H...H Contact  H14D     ..H325     .       1.18 Ang.
                                                      x,y,z  =     1_555 Check
PLAT410_ALERT_2_G Short Intra H...H Contact  H14E     ..H323     .       1.87 Ang.
                                                      x,y,z  =     1_555 Check
PLAT410_ALERT_2_G Short Intra H...H Contact  H14E     ..H325     .       1.79 Ang.
                                                      x,y,z  =     1_555 Check
PLAT410_ALERT_2_G Short Intra H...H Contact  H14I     ..H326     .       0.78 Ang.
                                                      x,y,z  =     1_555 Check
PLAT410_ALERT_2_G Short Intra H...H Contact  H14J     ..H326     .       1.30 Ang.
                                                      x,y,z  =     1_555 Check
PLAT412_ALERT_2_G Short Intra XH3 .. XHn     H24C     ..H203     .       1.94 Ang.
                                                      x,y,z  =     1_555 Check
PLAT412_ALERT_2_G Short Intra XH3 .. XHn     H26C     ..H207     .       1.83 Ang.
                                                      x,y,z  =     1_555 Check
PLAT412_ALERT_2_G Short Intra XH3 .. XHn     H28A     ..H208     .       1.54 Ang.
                                                      x,y,z  =     1_555 Check
PLAT412_ALERT_2_G Short Intra XH3 .. XHn     H28B     ..H208     .       1.61 Ang.
                                                      x,y,z  =     1_555 Check
PLAT412_ALERT_2_G Short Intra XH3 .. XHn     H28B     ..H210     .       2.03 Ang.
                                                      x,y,z  =     1_555 Check
PLAT412_ALERT_2_G Short Intra XH3 .. XHn     H28C     ..H208     .       1.78 Ang.
                                                      x,y,z  =     1_555 Check
PLAT412_ALERT_2_G Short Intra XH3 .. XHn     H30A     ..H210     .       2.05 Ang.
                                                      x,y,z  =     1_555 Check
PLAT412_ALERT_2_G Short Intra XH3 .. XHn     H30A     ..H212     .       1.83 Ang.
                                                      x,y,z  =     1_555 Check
PLAT412_ALERT_2_G Short Intra XH3 .. XHn     H30B     ..H212     .       1.80 Ang.
                                                      x,y,z  =     1_555 Check
PLAT412_ALERT_2_G Short Intra XH3 .. XHn     H30C     ..H212     .       1.86 Ang.
                                                      x,y,z  =     1_555 Check
PLAT412_ALERT_2_G Short Intra XH3 .. XHn     H60A     ..H239     .       1.94 Ang.
                                                      x,y,z  =     1_555 Check
PLAT412_ALERT_2_G Short Intra XH3 .. XHn     H60B     ..H239     .       1.93 Ang.
                                                      x,y,z  =     1_555 Check
PLAT412_ALERT_2_G Short Intra XH3 .. XHn     H60C     ..H239     .       1.92 Ang.
                                                      x,y,z  =     1_555 Check
PLAT412_ALERT_2_G Short Intra XH3 .. XHn     H62A     ..H241     .       2.11 Ang.
                                                      x,y,z  =     1_555 Check
PLAT412_ALERT_2_G Short Intra XH3 .. XHn     H62A     ..H243     .       1.75 Ang.
```

```
                                                 x,y,z  =     1_555 Check
PLAT412_ALERT_2_G Short Intra XH3 .. XHn     H62B     ..H243     .       1.71 Ang.
                                                 x,y,z  =     1_555 Check
PLAT412_ALERT_2_G Short Intra XH3 .. XHn     H62C     ..H243     .       1.85 Ang.
                                                 x,y,z  =     1_555 Check
PLAT412_ALERT_2_G Short Intra XH3 .. XHn     H64C     ..H244     .       2.01 Ang.
                                                 x,y,z  =     1_555 Check
PLAT412_ALERT_2_G Short Intra XH3 .. XHn     H72C     ..H254     .       2.07 Ang.
                                                 x,y,z  =     1_555 Check
PLAT412_ALERT_2_G Short Intra XH3 .. XHn     H10A     ..H280     .       1.73 Ang.
                                                 x,y,z  =     1_555 Check
PLAT412_ALERT_2_G Short Intra XH3 .. XHn     H10B     ..H280     .       1.84 Ang.
                                                 x,y,z  =     1_555 Check
PLAT412_ALERT_2_G Short Intra XH3 .. XHn     H10C     ..H280     .       1.81 Ang.
                                                 x,y,z  =     1_555 Check
PLAT412_ALERT_2_G Short Intra XH3 .. XHn     H10F     ..H284     .       1.97 Ang.
                                                 x,y,z  =     1_555 Check
PLAT412_ALERT_2_G Short Intra XH3 .. XHn     H10G     ..H284     .       2.04 Ang.
                                                 x,y,z  =     1_555 Check
PLAT412_ALERT_2_G Short Intra XH3 .. XHn     H10H     ..H284     .       1.99 Ang.
                                                 x,y,z  =     1_555 Check
PLAT412_ALERT_2_G Short Intra XH3 .. XHn     H10M     ..H285     .       2.02 Ang.
                                                 x,y,z  =     1_555 Check
PLAT412_ALERT_2_G Short Intra XH3 .. XHn     H10R     ..H289     .       2.02 Ang.
                                                 x,y,z  =     1_555 Check
PLAT412_ALERT_2_G Short Intra XH3 .. XHn     H96C     ..H275     .       2.09 Ang.
                                                 x,y,z  =     1_555 Check
PLAT412_ALERT_2_G Short Intra XH3 .. XHn     H98C     ..H279     .       2.06 Ang.
                                                 x,y,z  =     1_555 Check
PLAT412_ALERT_2_G Short Intra XH3 .. XHn     H13A     ..H310     .       1.90 Ang.
                                                 x,y,z  =     1_555 Check
PLAT412_ALERT_2_G Short Intra XH3 .. XHn     H13B     ..H310     .       1.78 Ang.
                                                 x,y,z  =     1_555 Check
PLAT412_ALERT_2_G Short Intra XH3 .. XHn     H13C     ..H310     .       1.65 Ang.
                                                 x,y,z  =     1_555 Check
PLAT412_ALERT_2_G Short Intra XH3 .. XHn     H13K     ..H315     .       2.11 Ang.
                                                 x,y,z  =     1_555 Check
PLAT412_ALERT_2_G Short Intra XH3 .. XHn     H14M     ..H326     .       2.13 Ang.
                                                 x,y,z  =     1_555 Check
PLAT413_ALERT_2_G Short Inter XH3 .. XHn     H68A     ..H282     .       1.94 Ang.
                                          x,1-y,-1/2+z  =     6_565 Check
PLAT432_ALERT_2_G Short Inter X...Y Contact  F4       ..C81B     .       1.45 Ang.
                                           1-x,y,3/2-z  =     2_656 Check
PLAT432_ALERT_2_G Short Inter X...Y Contact  F4       ..C1B      .       2.56 Ang.
                                           1-x,y,3/2-z  =     2_656 Check
PLAT432_ALERT_2_G Short Inter X...Y Contact  F4       ..C9B      .       2.59 Ang.
                                           1-x,y,3/2-z  =     2_656 Check
PLAT432_ALERT_2_G Short Inter X...Y Contact  F5       ..C81B     .       1.60 Ang.
                                           1-x,y,3/2-z  =     2_656 Check
PLAT432_ALERT_2_G Short Inter X...Y Contact  F5       ..C1B      .       2.50 Ang.
                                           1-x,y,3/2-z  =     2_656 Check
PLAT432_ALERT_2_G Short Inter X...Y Contact  F5       ..C2B      .       2.56 Ang.
                                           1-x,y,3/2-z  =     2_656 Check
PLAT432_ALERT_2_G Short Inter X...Y Contact  F6       ..C81B     .       1.02 Ang.
                                           1-x,y,3/2-z  =     2_656 Check
PLAT432_ALERT_2_G Short Inter X...Y Contact  F6       ..C1B      .       2.22 Ang.
                                           1-x,y,3/2-z  =     2_656 Check
```

```
PLAT432_ALERT_2_G Short Inter X...Y Contact  F6       ..C5B      .       2.63 Ang.
                                              1-x,y,3/2-z  =     2_656 Check
PLAT432_ALERT_2_G Short Inter X...Y Contact  F7       ..C81C     .       1.73 Ang.
                                               -x,y,3/2-z  =     2_556 Check
PLAT432_ALERT_2_G Short Inter X...Y Contact  F7       ..C1C      .       2.59 Ang.
                                               -x,y,3/2-z  =     2_556 Check
PLAT432_ALERT_2_G Short Inter X...Y Contact  F7       ..C5C      .       2.66 Ang.
                                               -x,y,3/2-z  =     2_556 Check
PLAT432_ALERT_2_G Short Inter X...Y Contact  F8       ..C81C     .       1.72 Ang.
                                               -x,y,3/2-z  =     2_556 Check
PLAT432_ALERT_2_G Short Inter X...Y Contact  F8       ..C45C     .       2.58 Ang.
                                               -x,y,3/2-z  =     2_556 Check
PLAT432_ALERT_2_G Short Inter X...Y Contact  F8       ..C1C      .       2.64 Ang.
                                               -x,y,3/2-z  =     2_556 Check
PLAT432_ALERT_2_G Short Inter X...Y Contact  F9       ..C81C     .       0.78 Ang.
                                               -x,y,3/2-z  =     2_556 Check
PLAT432_ALERT_2_G Short Inter X...Y Contact  F9       ..C1C      .       2.16 Ang.
                                               -x,y,3/2-z  =     2_556 Check
PLAT432_ALERT_2_G Short Inter X...Y Contact  F9       ..C2C      .       2.79 Ang.
                                               -x,y,3/2-z  =     2_556 Check
PLAT432_ALERT_2_G Short Inter X...Y Contact  N2       ..C17B     .       2.98 Ang.
                                              1-x,y,3/2-z  =     2_656 Check
PLAT432_ALERT_2_G Short Inter X...Y Contact  N10      ..C51A     .       3.02 Ang.
                                                    x,y,z  =     1_555 Check
PLAT432_ALERT_2_G Short Inter X...Y Contact  N16      ..C49C     .       2.96 Ang.
                                                    x,y,z  =     1_555 Check
PLAT432_ALERT_2_G Short Inter X...Y Contact  C1B      ..C1B      .       0.46 Ang.
                                              1-x,y,3/2-z  =     2_656 Check
PLAT432_ALERT_2_G Short Inter X...Y Contact  C1B      ..C9B      .       1.28 Ang.
                                              1-x,y,3/2-z  =     2_656 Check
PLAT432_ALERT_2_G Short Inter X...Y Contact  C1B      ..C2B      .       1.53 Ang.
                                              1-x,y,3/2-z  =     2_656 Check
PLAT432_ALERT_2_G Short Inter X...Y Contact  C1B      ..C81B     .       1.62 Ang.
                                              1-x,y,3/2-z  =     2_656 Check
PLAT432_ALERT_2_G Short Inter X...Y Contact  C1B      ..C5B      .       1.91 Ang.
                                              1-x,y,3/2-z  =     2_656 Check
PLAT432_ALERT_2_G Short Inter X...Y Contact  C1B      ..C10B     .       2.15 Ang.
                                              1-x,y,3/2-z  =     2_656 Check
PLAT432_ALERT_2_G Short Inter X...Y Contact  C1B      ..C66B     .       2.32 Ang.
                                              1-x,y,3/2-z  =     2_656 Check
PLAT432_ALERT_2_G Short Inter X...Y Contact  C1B      ..C8B      .       2.48 Ang.
                                              1-x,y,3/2-z  =     2_656 Check
PLAT432_ALERT_2_G Short Inter X...Y Contact  C1B      ..C71B     .       2.53 Ang.
                                              1-x,y,3/2-z  =     2_656 Check
PLAT432_ALERT_2_G Short Inter X...Y Contact  C1B      ..C3B      .       2.63 Ang.
                                              1-x,y,3/2-z  =     2_656 Check
PLAT432_ALERT_2_G Short Inter X...Y Contact  C1B      ..C4B      .       2.78 Ang.
                                              1-x,y,3/2-z  =     2_656 Check
PLAT432_ALERT_2_G Short Inter X...Y Contact  C1B      ..C6B      .       2.88 Ang.
                                              1-x,y,3/2-z  =     2_656 Check
PLAT432_ALERT_2_G Short Inter X...Y Contact  C1B      ..C7B      .       3.05 Ang.
                                              1-x,y,3/2-z  =     2_656 Check
PLAT432_ALERT_2_G Short Inter X...Y Contact  C1C      ..C1C      .       0.56 Ang.
                                               -x,y,3/2-z  =     2_556 Check
PLAT432_ALERT_2_G Short Inter X...Y Contact  C1C      ..C45C     .       1.31 Ang.
                                               -x,y,3/2-z  =     2_556 Check
PLAT432_ALERT_2_G Short Inter X...Y Contact  C1C      ..C5C      .       1.54 Ang.
```

```
                                       -x,y,3/2-z  =      2_556 Check
PLAT432_ALERT_2_G Short Inter X...Y Contact  C1C      ..C81C     .       1.66 Ang.
                                       -x,y,3/2-z  =      2_556 Check
PLAT432_ALERT_2_G Short Inter X...Y Contact  C1C      ..C46C     .       1.96 Ang.
                                       -x,y,3/2-z  =      2_556 Check
PLAT432_ALERT_2_G Short Inter X...Y Contact  C1C      ..C2C      .       2.07 Ang.
                                       -x,y,3/2-z  =      2_556 Check
PLAT432_ALERT_2_G Short Inter X...Y Contact  C1C      ..C74C     .       2.32 Ang.
                                       -x,y,3/2-z  =      2_556 Check
PLAT432_ALERT_2_G Short Inter X...Y Contact  C1C      ..C6C      .       2.36 Ang.
                                       -x,y,3/2-z  =      2_556 Check
PLAT432_ALERT_2_G Short Inter X...Y Contact  C1C      ..C44C     .       2.62 Ang.
                                       -x,y,3/2-z  =      2_556 Check
PLAT432_ALERT_2_G Short Inter X...Y Contact  C1C      ..C4C      .       2.64 Ang.
                                       -x,y,3/2-z  =      2_556 Check
PLAT432_ALERT_2_G Short Inter X...Y Contact  C1C      ..C3C      .       2.85 Ang.
                                       -x,y,3/2-z  =      2_556 Check
PLAT432_ALERT_2_G Short Inter X...Y Contact  C1C      ..C42C     .       3.13 Ang.
                                       -x,y,3/2-z  =      2_556 Check
PLAT432_ALERT_2_G Short Inter X...Y Contact  C2B      ..C9B      .       0.88 Ang.
                                      1-x,y,3/2-z  =      2_656 Check
PLAT432_ALERT_2_G Short Inter X...Y Contact  C2B      ..C8B      .       1.07 Ang.
                                      1-x,y,3/2-z  =      2_656 Check
PLAT432_ALERT_2_G Short Inter X...Y Contact  C2B      ..C7B      .       1.67 Ang.
                                      1-x,y,3/2-z  =      2_656 Check
PLAT432_ALERT_2_G Short Inter X...Y Contact  C2B      ..C5B      .       2.00 Ang.
                                      1-x,y,3/2-z  =      2_656 Check
PLAT432_ALERT_2_G Short Inter X...Y Contact  C2B      ..C6B      .       2.11 Ang.
                                      1-x,y,3/2-z  =      2_656 Check
PLAT432_ALERT_2_G Short Inter X...Y Contact  C2B      ..C10B     .       2.30 Ang.
                                      1-x,y,3/2-z  =      2_656 Check
PLAT432_ALERT_2_G Short Inter X...Y Contact  C2B      ..C81B     .       2.43 Ang.
                                      1-x,y,3/2-z  =      2_656 Check
PLAT432_ALERT_2_G Short Inter X...Y Contact  C2B      ..C13B     .       2.43 Ang.
                                      1-x,y,3/2-z  =      2_656 Check
PLAT432_ALERT_2_G Short Inter X...Y Contact  C2B      ..C2B      .       2.85 Ang.
                                      1-x,y,3/2-z  =      2_656 Check
PLAT432_ALERT_2_G Short Inter X...Y Contact  C2B      ..C80B     .       2.92 Ang.
                                      1-x,y,3/2-z  =      2_656 Check
PLAT432_ALERT_2_G Short Inter X...Y Contact  C2B      ..C11B     .       3.11 Ang.
                                      1-x,y,3/2-z  =      2_656 Check
PLAT432_ALERT_2_G Short Inter X...Y Contact  C2C      ..C74C     .       0.88 Ang.
                                       -x,y,3/2-z  =      2_556 Check
PLAT432_ALERT_2_G Short Inter X...Y Contact  C2C      ..C46C     .       0.95 Ang.
                                       -x,y,3/2-z  =      2_556 Check
PLAT432_ALERT_2_G Short Inter X...Y Contact  C2C      ..C45C     .       1.69 Ang.
                                       -x,y,3/2-z  =      2_556 Check
PLAT432_ALERT_2_G Short Inter X...Y Contact  C2C      ..C6C      .       1.78 Ang.
                                       -x,y,3/2-z  =      2_556 Check
PLAT432_ALERT_2_G Short Inter X...Y Contact  C2C      ..C5C      .       2.07 Ang.
                                       -x,y,3/2-z  =      2_556 Check
PLAT432_ALERT_2_G Short Inter X...Y Contact  C2C      ..C73C     .       2.18 Ang.
                                       -x,y,3/2-z  =      2_556 Check
PLAT432_ALERT_2_G Short Inter X...Y Contact  C2C      ..C47C     .       2.30 Ang.
                                       -x,y,3/2-z  =      2_556 Check
PLAT432_ALERT_2_G Short Inter X...Y Contact  C2C      ..C72C     .       2.73 Ang.
                                       -x,y,3/2-z  =      2_556 Check
```

```
PLAT432_ALERT_2_G Short Inter X...Y Contact  C2C      ..C81C     .       2.93 Ang.
                                              -x,y,3/2-z  =     2_556 Check
PLAT432_ALERT_2_G Short Inter X...Y Contact  C2C      ..C7C      .       3.00 Ang.
                                              -x,y,3/2-z  =     2_556 Check
PLAT432_ALERT_2_G Short Inter X...Y Contact  C2C      ..C44C     .       3.06 Ang.
                                              -x,y,3/2-z  =     2_556 Check
PLAT432_ALERT_2_G Short Inter X...Y Contact  C3B      ..C8B      .       0.87 Ang.
                                             1-x,y,3/2-z  =     2_656 Check
PLAT432_ALERT_2_G Short Inter X...Y Contact  C3B      ..C9B      .       1.13 Ang.
                                             1-x,y,3/2-z  =     2_656 Check
PLAT432_ALERT_2_G Short Inter X...Y Contact  C3B      ..C13B     .       1.37 Ang.
                                             1-x,y,3/2-z  =     2_656 Check
PLAT432_ALERT_2_G Short Inter X...Y Contact  C3B      ..C10B     .       1.68 Ang.
                                             1-x,y,3/2-z  =     2_656 Check
PLAT432_ALERT_2_G Short Inter X...Y Contact  C3B      ..C12B     .       1.90 Ang.
                                             1-x,y,3/2-z  =     2_656 Check
PLAT432_ALERT_2_G Short Inter X...Y Contact  C3B      ..C11B     .       1.96 Ang.
                                             1-x,y,3/2-z  =     2_656 Check
PLAT432_ALERT_2_G Short Inter X...Y Contact  C3B      ..C7B      .       2.24 Ang.
                                             1-x,y,3/2-z  =     2_656 Check
PLAT432_ALERT_2_G Short Inter X...Y Contact  C3B      ..C14B     .       2.62 Ang.
                                             1-x,y,3/2-z  =     2_656 Check
PLAT432_ALERT_2_G Short Inter X...Y Contact  C3B      ..C80B     .       2.94 Ang.
                                             1-x,y,3/2-z  =     2_656 Check
PLAT432_ALERT_2_G Short Inter X...Y Contact  C3B      ..C71B     .       3.04 Ang.
                                             1-x,y,3/2-z  =     2_656 Check
PLAT432_ALERT_2_G Short Inter X...Y Contact  C3C      ..C46C     .       0.49 Ang.
                                              -x,y,3/2-z  =     2_556 Check
PLAT432_ALERT_2_G Short Inter X...Y Contact  C3C      ..C47C     .       0.99 Ang.
                                              -x,y,3/2-z  =     2_556 Check
PLAT432_ALERT_2_G Short Inter X...Y Contact  C3C      ..C45C     .       1.61 Ang.
                                              -x,y,3/2-z  =     2_556 Check
PLAT432_ALERT_2_G Short Inter X...Y Contact  C3C      ..C74C     .       1.79 Ang.
                                              -x,y,3/2-z  =     2_556 Check
PLAT432_ALERT_2_G Short Inter X...Y Contact  C3C      ..C72C     .       2.02 Ang.
                                              -x,y,3/2-z  =     2_556 Check
PLAT432_ALERT_2_G Short Inter X...Y Contact  C3C      ..C48C     .       2.05 Ang.
                                              -x,y,3/2-z  =     2_556 Check
PLAT432_ALERT_2_G Short Inter X...Y Contact  C3C      ..C73C     .       2.34 Ang.
                                              -x,y,3/2-z  =     2_556 Check
PLAT432_ALERT_2_G Short Inter X...Y Contact  C3C      ..C44C     .       2.43 Ang.
                                              -x,y,3/2-z  =     2_556 Check
PLAT432_ALERT_2_G Short Inter X...Y Contact  C3C      ..C71C     .       2.59 Ang.
                                              -x,y,3/2-z  =     2_556 Check
PLAT432_ALERT_2_G Short Inter X...Y Contact  C3C      ..C6C      .       3.07 Ang.
                                              -x,y,3/2-z  =     2_556 Check
PLAT432_ALERT_2_G Short Inter X...Y Contact  C4B      ..C10B     .       0.42 Ang.
                                             1-x,y,3/2-z  =     2_656 Check
PLAT432_ALERT_2_G Short Inter X...Y Contact  C4B      ..C11B     .       1.03 Ang.
                                             1-x,y,3/2-z  =     2_656 Check
PLAT432_ALERT_2_G Short Inter X...Y Contact  C4B      ..C9B      .       1.46 Ang.
                                             1-x,y,3/2-z  =     2_656 Check
PLAT432_ALERT_2_G Short Inter X...Y Contact  C4B      ..C71B     .       1.82 Ang.
                                             1-x,y,3/2-z  =     2_656 Check
PLAT432_ALERT_2_G Short Inter X...Y Contact  C4B      ..C12B     .       2.06 Ang.
                                             1-x,y,3/2-z  =     2_656 Check
PLAT432_ALERT_2_G Short Inter X...Y Contact  C4B      ..C72B     .       2.17 Ang.
```

```
                                           1-x,y,3/2-z  =      2_656 Check
PLAT432_ALERT_2_G Short Inter X...Y Contact  C4B      ..C8B      .       2.21 Ang.
                                           1-x,y,3/2-z  =      2_656 Check
PLAT432_ALERT_2_G Short Inter X...Y Contact  C4B      ..C13B     .       2.45 Ang.
                                           1-x,y,3/2-z  =      2_656 Check
PLAT432_ALERT_2_G Short Inter X...Y Contact  C4B      ..C70B     .       2.46 Ang.
                                           1-x,y,3/2-z  =      2_656 Check
PLAT432_ALERT_2_G Short Inter X...Y Contact  C4B      ..C66B     .       2.87 Ang.
                                           1-x,y,3/2-z  =      2_656 Check
PLAT432_ALERT_2_G Short Inter X...Y Contact  C4C      ..C44C     .       1.06 Ang.
                                            -x,y,3/2-z  =      2_556 Check
PLAT432_ALERT_2_G Short Inter X...Y Contact  C4C      ..C45C     .       1.13 Ang.
                                            -x,y,3/2-z  =      2_556 Check
PLAT432_ALERT_2_G Short Inter X...Y Contact  C4C      ..C71C     .       1.45 Ang.
                                            -x,y,3/2-z  =      2_556 Check
PLAT432_ALERT_2_G Short Inter X...Y Contact  C4C      ..C46C     .       1.55 Ang.
                                            -x,y,3/2-z  =      2_556 Check
PLAT432_ALERT_2_G Short Inter X...Y Contact  C4C      ..C48C     .       1.83 Ang.
                                            -x,y,3/2-z  =      2_556 Check
PLAT432_ALERT_2_G Short Inter X...Y Contact  C4C      ..C47C     .       1.84 Ang.
                                            -x,y,3/2-z  =      2_556 Check
PLAT432_ALERT_2_G Short Inter X...Y Contact  C4C      ..C43C     .       2.43 Ang.
                                            -x,y,3/2-z  =      2_556 Check
PLAT432_ALERT_2_G Short Inter X...Y Contact  C4C      ..C37C     .       2.74 Ang.
                                            -x,y,3/2-z  =      2_556 Check
PLAT432_ALERT_2_G Short Inter X...Y Contact  C4C      ..C74C     .       2.96 Ang.
                                            -x,y,3/2-z  =      2_556 Check
PLAT432_ALERT_2_G Short Inter X...Y Contact  C4C      ..C38C     .       3.10 Ang.
                                            -x,y,3/2-z  =      2_556 Check
PLAT432_ALERT_2_G Short Inter X...Y Contact  C4C      ..C72C     .       3.17 Ang.
                                            -x,y,3/2-z  =      2_556 Check
PLAT432_ALERT_2_G Short Inter X...Y Contact  C5B      ..C10B     .       1.03 Ang.
                                           1-x,y,3/2-z  =      2_656 Check
PLAT432_ALERT_2_G Short Inter X...Y Contact  C5B      ..C71B     .       1.13 Ang.
                                           1-x,y,3/2-z  =      2_656 Check
PLAT432_ALERT_2_G Short Inter X...Y Contact  C5B      ..C9B      .       1.50 Ang.
                                           1-x,y,3/2-z  =      2_656 Check
PLAT432_ALERT_2_G Short Inter X...Y Contact  C5B      ..C66B     .       1.64 Ang.
                                           1-x,y,3/2-z  =      2_656 Check
PLAT432_ALERT_2_G Short Inter X...Y Contact  C5B      ..C11B     .       2.28 Ang.
                                           1-x,y,3/2-z  =      2_656 Check
PLAT432_ALERT_2_G Short Inter X...Y Contact  C5B      ..C70B     .       2.40 Ang.
                                           1-x,y,3/2-z  =      2_656 Check
PLAT432_ALERT_2_G Short Inter X...Y Contact  C5B      ..C81B     .       2.75 Ang.
                                           1-x,y,3/2-z  =      2_656 Check
PLAT432_ALERT_2_G Short Inter X...Y Contact  C5B      ..C8B      .       2.84 Ang.
                                           1-x,y,3/2-z  =      2_656 Check
PLAT432_ALERT_2_G Short Inter X...Y Contact  C5B      ..C72B     .       2.89 Ang.
                                           1-x,y,3/2-z  =      2_656 Check
PLAT432_ALERT_2_G Short Inter X...Y Contact  C5B      ..C67B     .       3.06 Ang.
                                           1-x,y,3/2-z  =      2_656 Check
PLAT432_ALERT_2_G Short Inter X...Y Contact  C5C      ..C45C     .       0.76 Ang.
                                            -x,y,3/2-z  =      2_556 Check
PLAT432_ALERT_2_G Short Inter X...Y Contact  C5C      ..C44C     .       1.08 Ang.
                                            -x,y,3/2-z  =      2_556 Check
PLAT432_ALERT_2_G Short Inter X...Y Contact  C5C      ..C43C     .       1.95 Ang.
                                            -x,y,3/2-z  =      2_556 Check
```

```
PLAT432_ALERT_2_G Short Inter X...Y Contact  C5C       ..C46C     .       2.10 Ang.
                                             -x,y,3/2-z  =      2_556 Check
PLAT432_ALERT_2_G Short Inter X...Y Contact  C5C       ..C42C     .       2.24 Ang.
                                             -x,y,3/2-z  =      2_556 Check
PLAT432_ALERT_2_G Short Inter X...Y Contact  C5C       ..C71C     .       2.40 Ang.
                                             -x,y,3/2-z  =      2_556 Check
PLAT432_ALERT_2_G Short Inter X...Y Contact  C5C       ..C81C     .       2.51 Ang.
                                             -x,y,3/2-z  =      2_556 Check
PLAT432_ALERT_2_G Short Inter X...Y Contact  C5C       ..C5C      .       2.88 Ang.
                                             -x,y,3/2-z  =      2_556 Check
PLAT432_ALERT_2_G Short Inter X...Y Contact  C5C       ..C47C     .       3.03 Ang.
                                             -x,y,3/2-z  =      2_556 Check
PLAT432_ALERT_2_G Short Inter X...Y Contact  C5C       ..C38C     .       3.06 Ang.
                                             -x,y,3/2-z  =      2_556 Check
PLAT432_ALERT_2_G Short Inter X...Y Contact  C5C       ..C48C     .       3.16 Ang.
                                             -x,y,3/2-z  =      2_556 Check
PLAT432_ALERT_2_G Short Inter X...Y Contact  C5C       ..C74C     .       3.18 Ang.
                                             -x,y,3/2-z  =      2_556 Check
PLAT432_ALERT_2_G Short Inter X...Y Contact  C6B       ..C71B     .       0.73 Ang.
                                            1-x,y,3/2-z  =      2_656 Check
PLAT432_ALERT_2_G Short Inter X...Y Contact  C6B       ..C66B     .       0.75 Ang.
                                            1-x,y,3/2-z  =      2_656 Check
PLAT432_ALERT_2_G Short Inter X...Y Contact  C6B       ..C70B     .       1.71 Ang.
                                            1-x,y,3/2-z  =      2_656 Check
PLAT432_ALERT_2_G Short Inter X...Y Contact  C6B       ..C67B     .       1.80 Ang.
                                            1-x,y,3/2-z  =      2_656 Check
PLAT432_ALERT_2_G Short Inter X...Y Contact  C6B       ..C10B     .       2.06 Ang.
                                            1-x,y,3/2-z  =      2_656 Check
PLAT432_ALERT_2_G Short Inter X...Y Contact  C6B       ..C68B     .       2.38 Ang.
                                            1-x,y,3/2-z  =      2_656 Check
PLAT432_ALERT_2_G Short Inter X...Y Contact  C6B       ..C69B     .       2.39 Ang.
                                            1-x,y,3/2-z  =      2_656 Check
PLAT432_ALERT_2_G Short Inter X...Y Contact  C6B       ..C72B     .       2.82 Ang.
                                            1-x,y,3/2-z  =      2_656 Check
PLAT432_ALERT_2_G Short Inter X...Y Contact  C6B       ..C9B      .       2.85 Ang.
                                            1-x,y,3/2-z  =      2_656 Check
PLAT432_ALERT_2_G Short Inter X...Y Contact  C6B       ..C11B     .       2.91 Ang.
                                            1-x,y,3/2-z  =      2_656 Check
PLAT432_ALERT_2_G Short Inter X...Y Contact  C6B       ..C41B     .       3.08 Ang.
                                            1-x,y,3/2-z  =      2_656 Check
PLAT432_ALERT_2_G Short Inter X...Y Contact  C6C       ..C43C     .       0.94 Ang.
                                             -x,y,3/2-z  =      2_556 Check
PLAT432_ALERT_2_G Short Inter X...Y Contact  C6C       ..C42C     .       0.99 Ang.
                                             -x,y,3/2-z  =      2_556 Check
PLAT432_ALERT_2_G Short Inter X...Y Contact  C6C       ..C44C     .       1.50 Ang.
                                             -x,y,3/2-z  =      2_556 Check
PLAT432_ALERT_2_G Short Inter X...Y Contact  C6C       ..C45C     .       2.16 Ang.
                                             -x,y,3/2-z  =      2_556 Check
PLAT432_ALERT_2_G Short Inter X...Y Contact  C6C       ..C38C     .       2.18 Ang.
                                             -x,y,3/2-z  =      2_556 Check
PLAT432_ALERT_2_G Short Inter X...Y Contact  C6C       ..C41C     .       2.32 Ang.
                                             -x,y,3/2-z  =      2_556 Check
PLAT432_ALERT_2_G Short Inter X...Y Contact  C6C       ..C71C     .       2.74 Ang.
                                             -x,y,3/2-z  =      2_556 Check
PLAT432_ALERT_2_G Short Inter X...Y Contact  C6C       ..C39C     .       3.01 Ang.
                                             -x,y,3/2-z  =      2_556 Check
PLAT432_ALERT_2_G Short Inter X...Y Contact  C6C       ..C37C     .       3.03 Ang.
```

```
                                            -x,y,3/2-z  =      2_556 Check
PLAT432_ALERT_2_G Short Inter X...Y Contact  C6C      ..C40C     .       3.07 Ang.
                                            -x,y,3/2-z  =      2_556 Check
PLAT432_ALERT_2_G Short Inter X...Y Contact  C7B      ..C66B     .       0.85 Ang.
                                           1-x,y,3/2-z  =      2_656 Check
PLAT432_ALERT_2_G Short Inter X...Y Contact  C7B      ..C67B     .       0.87 Ang.
                                           1-x,y,3/2-z  =      2_656 Check
PLAT432_ALERT_2_G Short Inter X...Y Contact  C7B      ..C41B     .       1.77 Ang.
                                           1-x,y,3/2-z  =      2_656 Check
PLAT432_ALERT_2_G Short Inter X...Y Contact  C7B      ..C68B     .       2.17 Ang.
                                           1-x,y,3/2-z  =      2_656 Check
PLAT432_ALERT_2_G Short Inter X...Y Contact  C7B      ..C71B     .       2.17 Ang.
                                           1-x,y,3/2-z  =      2_656 Check
PLAT432_ALERT_2_G Short Inter X...Y Contact  C7B      ..C42B     .       2.33 Ang.
                                           1-x,y,3/2-z  =      2_656 Check
PLAT432_ALERT_2_G Short Inter X...Y Contact  C7B      ..C70B     .       2.91 Ang.
                                           1-x,y,3/2-z  =      2_656 Check
PLAT432_ALERT_2_G Short Inter X...Y Contact  C7B      ..C69B     .       2.94 Ang.
                                           1-x,y,3/2-z  =      2_656 Check
PLAT432_ALERT_2_G Short Inter X...Y Contact  C7B      ..C40B     .       2.98 Ang.
                                           1-x,y,3/2-z  =      2_656 Check
PLAT432_ALERT_2_G Short Inter X...Y Contact  C7B      ..C78B     .       3.17 Ang.
                                           1-x,y,3/2-z  =      2_656 Check
PLAT432_ALERT_2_G Short Inter X...Y Contact  C7C      ..C43C     .       0.48 Ang.
                                            -x,y,3/2-z  =      2_556 Check
PLAT432_ALERT_2_G Short Inter X...Y Contact  C7C      ..C38C     .       0.89 Ang.
                                            -x,y,3/2-z  =      2_556 Check
PLAT432_ALERT_2_G Short Inter X...Y Contact  C7C      ..C42C     .       1.70 Ang.
                                            -x,y,3/2-z  =      2_556 Check
PLAT432_ALERT_2_G Short Inter X...Y Contact  C7C      ..C44C     .       1.83 Ang.
                                            -x,y,3/2-z  =      2_556 Check
PLAT432_ALERT_2_G Short Inter X...Y Contact  C7C      ..C39C     .       1.83 Ang.
                                            -x,y,3/2-z  =      2_556 Check
PLAT432_ALERT_2_G Short Inter X...Y Contact  C7C      ..C37C     .       2.05 Ang.
                                            -x,y,3/2-z  =      2_556 Check
PLAT432_ALERT_2_G Short Inter X...Y Contact  C7C      ..C41C     .       2.35 Ang.
                                            -x,y,3/2-z  =      2_556 Check
PLAT432_ALERT_2_G Short Inter X...Y Contact  C7C      ..C71C     .       2.39 Ang.
                                            -x,y,3/2-z  =      2_556 Check
PLAT432_ALERT_2_G Short Inter X...Y Contact  C7C      ..C40C     .       2.45 Ang.
                                            -x,y,3/2-z  =      2_556 Check
PLAT432_ALERT_2_G Short Inter X...Y Contact  C7C      ..C34C     .       2.93 Ang.
                                            -x,y,3/2-z  =      2_556 Check
PLAT432_ALERT_2_G Short Inter X...Y Contact  C7C      ..C45C     .       3.07 Ang.
                                            -x,y,3/2-z  =      2_556 Check
PLAT432_ALERT_2_G Short Inter X...Y Contact  C8B      ..C42B     .       1.50 Ang.
                                           1-x,y,3/2-z  =      2_656 Check
PLAT432_ALERT_2_G Short Inter X...Y Contact  C8B      ..C66B     .       1.77 Ang.
                                           1-x,y,3/2-z  =      2_656 Check
PLAT432_ALERT_2_G Short Inter X...Y Contact  C8B      ..C41B     .       1.98 Ang.
                                           1-x,y,3/2-z  =      2_656 Check
PLAT432_ALERT_2_G Short Inter X...Y Contact  C8B      ..C67B     .       2.07 Ang.
                                           1-x,y,3/2-z  =      2_656 Check
PLAT432_ALERT_2_G Short Inter X...Y Contact  C8B      ..C43B     .       2.82 Ang.
                                           1-x,y,3/2-z  =      2_656 Check
PLAT432_ALERT_2_G Short Inter X...Y Contact  C8B      ..C71B     .       3.09 Ang.
                                           1-x,y,3/2-z  =      2_656 Check
```

```
PLAT432_ALERT_2_G Short Inter X...Y Contact  C8C      ..C37C     .       0.62 Ang.
                                                -x,y,3/2-z  =     2_556 Check
PLAT432_ALERT_2_G Short Inter X...Y Contact  C8C      ..C38C     .       0.99 Ang.
                                                -x,y,3/2-z  =     2_556 Check
PLAT432_ALERT_2_G Short Inter X...Y Contact  C8C      ..C71C     .       1.35 Ang.
                                                -x,y,3/2-z  =     2_556 Check
PLAT432_ALERT_2_G Short Inter X...Y Contact  C8C      ..C43C     .       1.57 Ang.
                                                -x,y,3/2-z  =     2_556 Check
PLAT432_ALERT_2_G Short Inter X...Y Contact  C8C      ..C44C     .       1.83 Ang.
                                                -x,y,3/2-z  =     2_556 Check
PLAT432_ALERT_2_G Short Inter X...Y Contact  C8C      ..C36C     .       1.93 Ang.
                                                -x,y,3/2-z  =     2_556 Check
PLAT432_ALERT_2_G Short Inter X...Y Contact  C8C      ..C39C     .       2.19 Ang.
                                                -x,y,3/2-z  =     2_556 Check
PLAT432_ALERT_2_G Short Inter X...Y Contact  C8C      ..C48C     .       2.65 Ang.
                                                -x,y,3/2-z  =     2_556 Check
PLAT432_ALERT_2_G Short Inter X...Y Contact  C8C      ..C34C     .       2.73 Ang.
                                                -x,y,3/2-z  =     2_556 Check
PLAT432_ALERT_2_G Short Inter X...Y Contact  C8C      ..C35C     .       2.73 Ang.
                                                -x,y,3/2-z  =     2_556 Check
PLAT432_ALERT_2_G Short Inter X...Y Contact  C8C      ..C57C     .       3.00 Ang.
                                                -x,y,3/2-z  =     2_556 Check
PLAT432_ALERT_2_G Short Inter X...Y Contact  C8C      ..C42C     .       3.02 Ang.
                                                -x,y,3/2-z  =     2_556 Check
PLAT432_ALERT_2_G Short Inter X...Y Contact  C9B      ..C66B     .       2.31 Ang.
                                               1-x,y,3/2-z  =     2_656 Check
PLAT432_ALERT_2_G Short Inter X...Y Contact  C9B      ..C81B     .       2.43 Ang.
                                               1-x,y,3/2-z  =     2_656 Check
PLAT432_ALERT_2_G Short Inter X...Y Contact  C9B      ..C42B     .       2.52 Ang.
                                               1-x,y,3/2-z  =     2_656 Check
PLAT432_ALERT_2_G Short Inter X...Y Contact  C9B      ..C9B      .       2.64 Ang.
                                               1-x,y,3/2-z  =     2_656 Check
PLAT432_ALERT_2_G Short Inter X...Y Contact  C9B      ..C45B     .       2.87 Ang.
                                               1-x,y,3/2-z  =     2_656 Check
PLAT432_ALERT_2_G Short Inter X...Y Contact  C9C      ..C71C     .       0.27 Ang.
                                                -x,y,3/2-z  =     2_556 Check
PLAT432_ALERT_2_G Short Inter X...Y Contact  C9C      ..C48C     .       1.30 Ang.
                                                -x,y,3/2-z  =     2_556 Check
PLAT432_ALERT_2_G Short Inter X...Y Contact  C9C      ..C37C     .       1.44 Ang.
                                                -x,y,3/2-z  =     2_556 Check
PLAT432_ALERT_2_G Short Inter X...Y Contact  C9C      ..C44C     .       1.53 Ang.
                                                -x,y,3/2-z  =     2_556 Check
PLAT432_ALERT_2_G Short Inter X...Y Contact  C9C      ..C49C     .       2.30 Ang.
                                                -x,y,3/2-z  =     2_556 Check
PLAT432_ALERT_2_G Short Inter X...Y Contact  C9C      ..C43C     .       2.32 Ang.
                                                -x,y,3/2-z  =     2_556 Check
PLAT432_ALERT_2_G Short Inter X...Y Contact  C9C      ..C38C     .       2.34 Ang.
                                                -x,y,3/2-z  =     2_556 Check
PLAT432_ALERT_2_G Short Inter X...Y Contact  C9C      ..C47C     .       2.34 Ang.
                                                -x,y,3/2-z  =     2_556 Check
PLAT432_ALERT_2_G Short Inter X...Y Contact  C9C      ..C36C     .       2.37 Ang.
                                                -x,y,3/2-z  =     2_556 Check
PLAT432_ALERT_2_G Short Inter X...Y Contact  C9C      ..C45C     .       2.50 Ang.
                                                -x,y,3/2-z  =     2_556 Check
PLAT432_ALERT_2_G Short Inter X...Y Contact  C9C      ..C57C     .       2.70 Ang.
                                                -x,y,3/2-z  =     2_556 Check
PLAT432_ALERT_2_G Short Inter X...Y Contact  C9C      ..C46C     .       2.79 Ang.
```

```
                                                   -x,y,3/2-z  =      2_556 Check
PLAT432_ALERT_2_G Short Inter X...Y Contact  C10B     ..C45B     .       1.64 Ang.
                                                  1-x,y,3/2-z  =      2_656 Check
PLAT432_ALERT_2_G Short Inter X...Y Contact  C10B     ..C46B     .       2.44 Ang.
                                                  1-x,y,3/2-z  =      2_656 Check
PLAT432_ALERT_2_G Short Inter X...Y Contact  C10B     ..C79B     .       2.58 Ang.
                                                  1-x,y,3/2-z  =      2_656 Check
PLAT432_ALERT_2_G Short Inter X...Y Contact  C10B     ..C44B     .       2.76 Ang.
                                                  1-x,y,3/2-z  =      2_656 Check
PLAT432_ALERT_2_G Short Inter X...Y Contact  C10B     ..C42B     .       2.84 Ang.
                                                  1-x,y,3/2-z  =      2_656 Check
PLAT432_ALERT_2_G Short Inter X...Y Contact  C10C     ..C48C     .       0.88 Ang.
                                                   -x,y,3/2-z  =      2_556 Check
PLAT432_ALERT_2_G Short Inter X...Y Contact  C10C     ..C49C     .       0.89 Ang.
                                                   -x,y,3/2-z  =      2_556 Check
PLAT432_ALERT_2_G Short Inter X...Y Contact  C10C     ..C71C     .       1.62 Ang.
                                                   -x,y,3/2-z  =      2_556 Check
PLAT432_ALERT_2_G Short Inter X...Y Contact  C10C     ..C57C     .       1.65 Ang.
                                                   -x,y,3/2-z  =      2_556 Check
PLAT432_ALERT_2_G Short Inter X...Y Contact  C10C     ..C37C     .       2.11 Ang.
                                                   -x,y,3/2-z  =      2_556 Check
PLAT432_ALERT_2_G Short Inter X...Y Contact  C10C     ..C50C     .       2.15 Ang.
                                                   -x,y,3/2-z  =      2_556 Check
PLAT432_ALERT_2_G Short Inter X...Y Contact  C10C     ..C36C     .       2.15 Ang.
                                                   -x,y,3/2-z  =      2_556 Check
PLAT432_ALERT_2_G Short Inter X...Y Contact  C10C     ..C47C     .       2.20 Ang.
                                                   -x,y,3/2-z  =      2_556 Check
PLAT432_ALERT_2_G Short Inter X...Y Contact  C10C     ..C56C     .       2.79 Ang.
                                                   -x,y,3/2-z  =      2_556 Check
PLAT432_ALERT_2_G Short Inter X...Y Contact  C10C     ..C44C     .       2.89 Ang.
                                                   -x,y,3/2-z  =      2_556 Check
PLAT432_ALERT_2_G Short Inter X...Y Contact  C10C     ..C55C     .       3.01 Ang.
                                                   -x,y,3/2-z  =      2_556 Check
PLAT432_ALERT_2_G Short Inter X...Y Contact  C10C     ..C51C     .       3.05 Ang.
                                                   -x,y,3/2-z  =      2_556 Check
PLAT432_ALERT_2_G Short Inter X...Y Contact  C10C     ..C72C     .       3.07 Ang.
                                                   -x,y,3/2-z  =      2_556 Check
PLAT432_ALERT_2_G Short Inter X...Y Contact  C11B     ..C45B     .       0.53 Ang.
                                                  1-x,y,3/2-z  =      2_656 Check
PLAT432_ALERT_2_G Short Inter X...Y Contact  C11B     ..C44B     .       1.49 Ang.
                                                  1-x,y,3/2-z  =      2_656 Check
PLAT432_ALERT_2_G Short Inter X...Y Contact  C11B     ..C46B     .       1.94 Ang.
                                                  1-x,y,3/2-z  =      2_656 Check
PLAT432_ALERT_2_G Short Inter X...Y Contact  C11B     ..C43B     .       2.24 Ang.
                                                  1-x,y,3/2-z  =      2_656 Check
PLAT432_ALERT_2_G Short Inter X...Y Contact  C11B     ..C42B     .       2.45 Ang.
                                                  1-x,y,3/2-z  =      2_656 Check
PLAT432_ALERT_2_G Short Inter X...Y Contact  C11B     ..C65B     .       2.74 Ang.
                                                  1-x,y,3/2-z  =      2_656 Check
PLAT432_ALERT_2_G Short Inter X...Y Contact  C11B     ..C79B     .       2.77 Ang.
                                                  1-x,y,3/2-z  =      2_656 Check
PLAT432_ALERT_2_G Short Inter X...Y Contact  C11B     ..C47B     .       3.03 Ang.
                                                  1-x,y,3/2-z  =      2_656 Check
PLAT432_ALERT_2_G Short Inter X...Y Contact  C11C     ..C57C     .       0.24 Ang.
                                                   -x,y,3/2-z  =      2_556 Check
PLAT432_ALERT_2_G Short Inter X...Y Contact  C11C     ..C49C     .       1.26 Ang.
                                                   -x,y,3/2-z  =      2_556 Check
```

```
PLAT432_ALERT_2_G Short Inter X...Y Contact  C11C     ..C36C     .       1.46 Ang.
                                              -x,y,3/2-z  =     2_556 Check
PLAT432_ALERT_2_G Short Inter X...Y Contact  C11C     ..C56C     .       1.58 Ang.
                                              -x,y,3/2-z  =     2_556 Check
PLAT432_ALERT_2_G Short Inter X...Y Contact  C11C     ..C50C     .       2.22 Ang.
                                              -x,y,3/2-z  =     2_556 Check
PLAT432_ALERT_2_G Short Inter X...Y Contact  C11C     ..C48C     .       2.29 Ang.
                                              -x,y,3/2-z  =     2_556 Check
PLAT432_ALERT_2_G Short Inter X...Y Contact  C11C     ..C37C     .       2.32 Ang.
                                              -x,y,3/2-z  =     2_556 Check
PLAT432_ALERT_2_G Short Inter X...Y Contact  C11C     ..C55C     .       2.34 Ang.
                                              -x,y,3/2-z  =     2_556 Check
PLAT432_ALERT_2_G Short Inter X...Y Contact  C11C     ..C35C     .       2.54 Ang.
                                              -x,y,3/2-z  =     2_556 Check
PLAT432_ALERT_2_G Short Inter X...Y Contact  C11C     ..C30C     .       2.64 Ang.
                                              -x,y,3/2-z  =     2_556 Check
PLAT432_ALERT_2_G Short Inter X...Y Contact  C11C     ..C71C     .       2.66 Ang.
                                              -x,y,3/2-z  =     2_556 Check
PLAT432_ALERT_2_G Short Inter X...Y Contact  C11C     ..C31C     .       2.98 Ang.
                                              -x,y,3/2-z  =     2_556 Check
PLAT432_ALERT_2_G Short Inter X...Y Contact  C12B     ..C43B     .       0.82 Ang.
                                             1-x,y,3/2-z  =     2_656 Check
PLAT432_ALERT_2_G Short Inter X...Y Contact  C12B     ..C44B     .       1.17 Ang.
                                             1-x,y,3/2-z  =     2_656 Check
PLAT432_ALERT_2_G Short Inter X...Y Contact  C12B     ..C42B     .       1.38 Ang.
                                             1-x,y,3/2-z  =     2_656 Check
PLAT432_ALERT_2_G Short Inter X...Y Contact  C12B     ..C45B     .       1.78 Ang.
                                             1-x,y,3/2-z  =     2_656 Check
PLAT432_ALERT_2_G Short Inter X...Y Contact  C12B     ..C38B     .       2.21 Ang.
                                             1-x,y,3/2-z  =     2_656 Check
PLAT432_ALERT_2_G Short Inter X...Y Contact  C12B     ..C65B     .       2.45 Ang.
                                             1-x,y,3/2-z  =     2_656 Check
PLAT432_ALERT_2_G Short Inter X...Y Contact  C12B     ..C41B     .       2.75 Ang.
                                             1-x,y,3/2-z  =     2_656 Check
PLAT432_ALERT_2_G Short Inter X...Y Contact  C12B     ..C37B     .       2.86 Ang.
                                             1-x,y,3/2-z  =     2_656 Check
PLAT432_ALERT_2_G Short Inter X...Y Contact  C12B     ..C46B     .       3.18 Ang.
                                             1-x,y,3/2-z  =     2_656 Check
PLAT432_ALERT_2_G Short Inter X...Y Contact  C12C     ..C36C     .       0.60 Ang.
                                              -x,y,3/2-z  =     2_556 Check
PLAT432_ALERT_2_G Short Inter X...Y Contact  C12C     ..C35C     .       1.13 Ang.
                                              -x,y,3/2-z  =     2_556 Check
PLAT432_ALERT_2_G Short Inter X...Y Contact  C12C     ..C57C     .       1.37 Ang.
                                              -x,y,3/2-z  =     2_556 Check
PLAT432_ALERT_2_G Short Inter X...Y Contact  C12C     ..C31C     .       1.93 Ang.
                                              -x,y,3/2-z  =     2_556 Check
PLAT432_ALERT_2_G Short Inter X...Y Contact  C12C     ..C37C     .       1.96 Ang.
                                              -x,y,3/2-z  =     2_556 Check
PLAT432_ALERT_2_G Short Inter X...Y Contact  C12C     ..C56C     .       2.07 Ang.
                                              -x,y,3/2-z  =     2_556 Check
PLAT432_ALERT_2_G Short Inter X...Y Contact  C12C     ..C30C     .       2.30 Ang.
                                              -x,y,3/2-z  =     2_556 Check
PLAT432_ALERT_2_G Short Inter X...Y Contact  C12C     ..C34C     .       2.39 Ang.
                                              -x,y,3/2-z  =     2_556 Check
PLAT432_ALERT_2_G Short Inter X...Y Contact  C12C     ..C49C     .       2.63 Ang.
                                              -x,y,3/2-z  =     2_556 Check
PLAT432_ALERT_2_G Short Inter X...Y Contact  C12C     ..C38C     .       2.87 Ang.
```

```
                                                  -x,y,3/2-z  =      2_556 Check
PLAT432_ALERT_2_G Short Inter X...Y Contact  C12C     ..C71C      .       3.00 Ang.
                                                  -x,y,3/2-z  =      2_556 Check
PLAT432_ALERT_2_G Short Inter X...Y Contact  C12C     ..C39C      .       3.07 Ang.
                                                  -x,y,3/2-z  =      2_556 Check
PLAT432_ALERT_2_G Short Inter X...Y Contact  C12C     ..C32C      .       3.15 Ang.
                                                  -x,y,3/2-z  =      2_556 Check
PLAT432_ALERT_2_G Short Inter X...Y Contact  C13B     ..C42B      .       0.24 Ang.
                                                 1-x,y,3/2-z  =      2_656 Check
PLAT432_ALERT_2_G Short Inter X...Y Contact  C13B     ..C41B      .       1.51 Ang.
                                                 1-x,y,3/2-z  =      2_656 Check
PLAT432_ALERT_2_G Short Inter X...Y Contact  C13B     ..C43B      .       1.55 Ang.
                                                 1-x,y,3/2-z  =      2_656 Check
PLAT432_ALERT_2_G Short Inter X...Y Contact  C13B     ..C67B      .       2.53 Ang.
                                                 1-x,y,3/2-z  =      2_656 Check
PLAT432_ALERT_2_G Short Inter X...Y Contact  C13B     ..C44B      .       2.59 Ang.
                                                 1-x,y,3/2-z  =      2_656 Check
PLAT432_ALERT_2_G Short Inter X...Y Contact  C13B     ..C40B      .       2.59 Ang.
                                                 1-x,y,3/2-z  =      2_656 Check
PLAT432_ALERT_2_G Short Inter X...Y Contact  C13B     ..C38B      .       2.64 Ang.
                                                 1-x,y,3/2-z  =      2_656 Check
PLAT432_ALERT_2_G Short Inter X...Y Contact  C13B     ..C66B      .       2.92 Ang.
                                                 1-x,y,3/2-z  =      2_656 Check
PLAT432_ALERT_2_G Short Inter X...Y Contact  C13B     ..C45B      .       2.94 Ang.
                                                 1-x,y,3/2-z  =      2_656 Check
PLAT432_ALERT_2_G Short Inter X...Y Contact  C13B     ..C39B      .       3.04 Ang.
                                                 1-x,y,3/2-z  =      2_656 Check
PLAT432_ALERT_2_G Short Inter X...Y Contact  C13C     ..C36C      .       1.10 Ang.
                                                  -x,y,3/2-z  =      2_556 Check
PLAT432_ALERT_2_G Short Inter X...Y Contact  C13C     ..C37C      .       1.16 Ang.
                                                  -x,y,3/2-z  =      2_556 Check
PLAT432_ALERT_2_G Short Inter X...Y Contact  C13C     ..C35C      .       1.34 Ang.
                                                  -x,y,3/2-z  =      2_556 Check
PLAT432_ALERT_2_G Short Inter X...Y Contact  C13C     ..C38C      .       1.50 Ang.
                                                  -x,y,3/2-z  =      2_556 Check
PLAT432_ALERT_2_G Short Inter X...Y Contact  C13C     ..C34C      .       1.59 Ang.
                                                  -x,y,3/2-z  =      2_556 Check
PLAT432_ALERT_2_G Short Inter X...Y Contact  C13C     ..C39C      .       1.76 Ang.
                                                  -x,y,3/2-z  =      2_556 Check
PLAT432_ALERT_2_G Short Inter X...Y Contact  C13C     ..C71C      .       2.52 Ang.
                                                  -x,y,3/2-z  =      2_556 Check
PLAT432_ALERT_2_G Short Inter X...Y Contact  C13C     ..C57C      .       2.54 Ang.
                                                  -x,y,3/2-z  =      2_556 Check
PLAT432_ALERT_2_G Short Inter X...Y Contact  C13C     ..C43C      .       2.64 Ang.
                                                  -x,y,3/2-z  =      2_556 Check
PLAT432_ALERT_2_G Short Inter X...Y Contact  C13C     ..C31C      .       2.69 Ang.
                                                  -x,y,3/2-z  =      2_556 Check
PLAT432_ALERT_2_G Short Inter X...Y Contact  C13C     ..C33C      .       3.03 Ang.
                                                  -x,y,3/2-z  =      2_556 Check
PLAT432_ALERT_2_G Short Inter X...Y Contact  C14B     ..C41B      .       0.57 Ang.
                                                 1-x,y,3/2-z  =      2_656 Check
PLAT432_ALERT_2_G Short Inter X...Y Contact  C14B     ..C40B      .       1.25 Ang.
                                                 1-x,y,3/2-z  =      2_656 Check
PLAT432_ALERT_2_G Short Inter X...Y Contact  C14B     ..C42B      .       1.33 Ang.
                                                 1-x,y,3/2-z  =      2_656 Check
PLAT432_ALERT_2_G Short Inter X...Y Contact  C14B     ..C67B      .       2.01 Ang.
                                                 1-x,y,3/2-z  =      2_656 Check
```

```
PLAT432_ALERT_2_G Short Inter X...Y Contact  C14B     ..C39B     .       2.08 Ang.
                                               1-x,y,3/2-z  =     2_656 Check
PLAT432_ALERT_2_G Short Inter X...Y Contact  C14B     ..C43B     .       2.09 Ang.
                                               1-x,y,3/2-z  =     2_656 Check
PLAT432_ALERT_2_G Short Inter X...Y Contact  C14B     ..C38B     .       2.39 Ang.
                                               1-x,y,3/2-z  =     2_656 Check
PLAT432_ALERT_2_G Short Inter X...Y Contact  C14B     ..C78B     .       2.48 Ang.
                                               1-x,y,3/2-z  =     2_656 Check
PLAT432_ALERT_2_G Short Inter X...Y Contact  C14B     ..C68B     .       2.72 Ang.
                                               1-x,y,3/2-z  =     2_656 Check
PLAT432_ALERT_2_G Short Inter X...Y Contact  C14B     ..C66B     .       3.07 Ang.
                                               1-x,y,3/2-z  =     2_656 Check
PLAT432_ALERT_2_G Short Inter X...Y Contact  C14C     ..C34C     .       0.61 Ang.
                                                -x,y,3/2-z  =     2_556 Check
PLAT432_ALERT_2_G Short Inter X...Y Contact  C14C     ..C39C     .       0.70 Ang.
                                                -x,y,3/2-z  =     2_556 Check
PLAT432_ALERT_2_G Short Inter X...Y Contact  C14C     ..C38C     .       1.70 Ang.
                                                -x,y,3/2-z  =     2_556 Check
PLAT432_ALERT_2_G Short Inter X...Y Contact  C14C     ..C35C     .       1.81 Ang.
                                                -x,y,3/2-z  =     2_556 Check
PLAT432_ALERT_2_G Short Inter X...Y Contact  C14C     ..C33C     .       2.03 Ang.
                                                -x,y,3/2-z  =     2_556 Check
PLAT432_ALERT_2_G Short Inter X...Y Contact  C14C     ..C40C     .       2.10 Ang.
                                                -x,y,3/2-z  =     2_556 Check
PLAT432_ALERT_2_G Short Inter X...Y Contact  C14C     ..C37C     .       2.42 Ang.
                                                -x,y,3/2-z  =     2_556 Check
PLAT432_ALERT_2_G Short Inter X...Y Contact  C14C     ..C36C     .       2.48 Ang.
                                                -x,y,3/2-z  =     2_556 Check
PLAT432_ALERT_2_G Short Inter X...Y Contact  C14C     ..C43C     .       2.75 Ang.
                                                -x,y,3/2-z  =     2_556 Check
PLAT432_ALERT_2_G Short Inter X...Y Contact  C14C     ..C70C     .       2.76 Ang.
                                                -x,y,3/2-z  =     2_556 Check
PLAT432_ALERT_2_G Short Inter X...Y Contact  C14C     ..C69C     .       2.78 Ang.
                                                -x,y,3/2-z  =     2_556 Check
PLAT432_ALERT_2_G Short Inter X...Y Contact  C14C     ..C31C     .       2.88 Ang.
                                                -x,y,3/2-z  =     2_556 Check
PLAT432_ALERT_2_G Short Inter X...Y Contact  C14C     ..C32C     .       2.93 Ang.
                                                -x,y,3/2-z  =     2_556 Check
PLAT432_ALERT_2_G Short Inter X...Y Contact  C14C     ..C41C     .       3.13 Ang.
                                                -x,y,3/2-z  =     2_556 Check
PLAT432_ALERT_2_G Short Inter X...Y Contact  C15B     ..C39B     .       0.68 Ang.
                                               1-x,y,3/2-z  =     2_656 Check
PLAT432_ALERT_2_G Short Inter X...Y Contact  C15B     ..C40B     .       1.03 Ang.
                                               1-x,y,3/2-z  =     2_656 Check
PLAT432_ALERT_2_G Short Inter X...Y Contact  C15B     ..C38B     .       1.54 Ang.
                                               1-x,y,3/2-z  =     2_656 Check
PLAT432_ALERT_2_G Short Inter X...Y Contact  C15B     ..C41B     .       1.89 Ang.
                                               1-x,y,3/2-z  =     2_656 Check
PLAT432_ALERT_2_G Short Inter X...Y Contact  C15B     ..C34B     .       2.07 Ang.
                                               1-x,y,3/2-z  =     2_656 Check
PLAT432_ALERT_2_G Short Inter X...Y Contact  C15B     ..C43B     .       2.19 Ang.
                                               1-x,y,3/2-z  =     2_656 Check
PLAT432_ALERT_2_G Short Inter X...Y Contact  C15B     ..C42B     .       2.34 Ang.
                                               1-x,y,3/2-z  =     2_656 Check
PLAT432_ALERT_2_G Short Inter X...Y Contact  C15B     ..C78B     .       2.44 Ang.
                                               1-x,y,3/2-z  =     2_656 Check
PLAT432_ALERT_2_G Short Inter X...Y Contact  C15B     ..C37B     .       2.87 Ang.
```

```
                                                     1-x,y,3/2-z  =      2_656 Check
PLAT432_ALERT_2_G Short Inter X...Y Contact  C15B     ..C33B     .       2.97 Ang.
                                                     1-x,y,3/2-z  =      2_656 Check
PLAT432_ALERT_2_G Short Inter X...Y Contact  C15B     ..C77B     .       3.12 Ang.
                                                     1-x,y,3/2-z  =      2_656 Check
PLAT432_ALERT_2_G Short Inter X...Y Contact  C15B     ..C67B     .       3.16 Ang.
                                                     1-x,y,3/2-z  =      2_656 Check
PLAT432_ALERT_2_G Short Inter X...Y Contact  C15B     ..C35B     .       3.19 Ang.
                                                     1-x,y,3/2-z  =      2_656 Check
PLAT432_ALERT_2_G Short Inter X...Y Contact  C15C     ..C33C     .       0.68 Ang.
                                                      -x,y,3/2-z  =      2_556 Check
PLAT432_ALERT_2_G Short Inter X...Y Contact  C15C     ..C34C     .       0.92 Ang.
                                                      -x,y,3/2-z  =      2_556 Check
PLAT432_ALERT_2_G Short Inter X...Y Contact  C15C     ..C69C     .       1.70 Ang.
                                                      -x,y,3/2-z  =      2_556 Check
PLAT432_ALERT_2_G Short Inter X...Y Contact  C15C     ..C32C     .       1.82 Ang.
                                                      -x,y,3/2-z  =      2_556 Check
PLAT432_ALERT_2_G Short Inter X...Y Contact  C15C     ..C39C     .       1.88 Ang.
                                                      -x,y,3/2-z  =      2_556 Check
PLAT432_ALERT_2_G Short Inter X...Y Contact  C15C     ..C35C     .       2.00 Ang.
                                                      -x,y,3/2-z  =      2_556 Check
PLAT432_ALERT_2_G Short Inter X...Y Contact  C15C     ..C31C     .       2.37 Ang.
                                                      -x,y,3/2-z  =      2_556 Check
PLAT432_ALERT_2_G Short Inter X...Y Contact  C15C     ..C70C     .       2.40 Ang.
                                                      -x,y,3/2-z  =      2_556 Check
PLAT432_ALERT_2_G Short Inter X...Y Contact  C15C     ..C40C     .       2.50 Ang.
                                                      -x,y,3/2-z  =      2_556 Check
PLAT432_ALERT_2_G Short Inter X...Y Contact  C15C     ..C68C     .       2.91 Ang.
                                                      -x,y,3/2-z  =      2_556 Check
PLAT432_ALERT_2_G Short Inter X...Y Contact  C15C     ..C27C     .       2.97 Ang.
                                                      -x,y,3/2-z  =      2_556 Check
PLAT432_ALERT_2_G Short Inter X...Y Contact  C15C     ..C38C     .       2.99 Ang.
                                                      -x,y,3/2-z  =      2_556 Check
PLAT432_ALERT_2_G Short Inter X...Y Contact  C15C     ..C36C     .       3.18 Ang.
                                                      -x,y,3/2-z  =      2_556 Check
PLAT432_ALERT_2_G Short Inter X...Y Contact  C16B     ..C38B     .       0.33 Ang.
                                                     1-x,y,3/2-z  =      2_656 Check
PLAT432_ALERT_2_G Short Inter X...Y Contact  C16B     ..C39B     .       1.24 Ang.
                                                     1-x,y,3/2-z  =      2_656 Check
PLAT432_ALERT_2_G Short Inter X...Y Contact  C16B     ..C37B     .       1.50 Ang.
                                                     1-x,y,3/2-z  =      2_656 Check
PLAT432_ALERT_2_G Short Inter X...Y Contact  C16B     ..C43B     .       1.73 Ang.
                                                     1-x,y,3/2-z  =      2_656 Check
PLAT432_ALERT_2_G Short Inter X...Y Contact  C16B     ..C34B     .       2.22 Ang.
                                                     1-x,y,3/2-z  =      2_656 Check
PLAT432_ALERT_2_G Short Inter X...Y Contact  C16B     ..C40B     .       2.43 Ang.
                                                     1-x,y,3/2-z  =      2_656 Check
PLAT432_ALERT_2_G Short Inter X...Y Contact  C16B     ..C36B     .       2.47 Ang.
                                                     1-x,y,3/2-z  =      2_656 Check
PLAT432_ALERT_2_G Short Inter X...Y Contact  C16B     ..C65B     .       2.55 Ang.
                                                     1-x,y,3/2-z  =      2_656 Check
PLAT432_ALERT_2_G Short Inter X...Y Contact  C16B     ..C44B     .       2.62 Ang.
                                                     1-x,y,3/2-z  =      2_656 Check
PLAT432_ALERT_2_G Short Inter X...Y Contact  C16B     ..C35B     .       2.67 Ang.
                                                     1-x,y,3/2-z  =      2_656 Check
PLAT432_ALERT_2_G Short Inter X...Y Contact  C16B     ..C42B     .       2.71 Ang.
                                                     1-x,y,3/2-z  =      2_656 Check
```

```
PLAT432_ALERT_2_G Short Inter X...Y Contact  C16B     ..C41B     .       2.98 Ang.
                                               1-x,y,3/2-z  =     2_656 Check
PLAT432_ALERT_2_G Short Inter X...Y Contact  C16C     ..C32C     .       0.69 Ang.
                                                -x,y,3/2-z  =     2_556 Check
PLAT432_ALERT_2_G Short Inter X...Y Contact  C16C     ..C31C     .       1.12 Ang.
                                                -x,y,3/2-z  =     2_556 Check
PLAT432_ALERT_2_G Short Inter X...Y Contact  C16C     ..C33C     .       1.15 Ang.
                                                -x,y,3/2-z  =     2_556 Check
PLAT432_ALERT_2_G Short Inter X...Y Contact  C16C     ..C35C     .       1.65 Ang.
                                                -x,y,3/2-z  =     2_556 Check
PLAT432_ALERT_2_G Short Inter X...Y Contact  C16C     ..C34C     .       1.73 Ang.
                                                -x,y,3/2-z  =     2_556 Check
PLAT432_ALERT_2_G Short Inter X...Y Contact  C16C     ..C27C     .       2.07 Ang.
                                                -x,y,3/2-z  =     2_556 Check
PLAT432_ALERT_2_G Short Inter X...Y Contact  C16C     ..C30C     .       2.38 Ang.
                                                -x,y,3/2-z  =     2_556 Check
PLAT432_ALERT_2_G Short Inter X...Y Contact  C16C     ..C69C     .       2.47 Ang.
                                                -x,y,3/2-z  =     2_556 Check
PLAT432_ALERT_2_G Short Inter X...Y Contact  C16C     ..C28C     .       2.95 Ang.
                                                -x,y,3/2-z  =     2_556 Check
PLAT432_ALERT_2_G Short Inter X...Y Contact  C16C     ..C36C     .       2.96 Ang.
                                                -x,y,3/2-z  =     2_556 Check
PLAT432_ALERT_2_G Short Inter X...Y Contact  C16C     ..C39C     .       2.98 Ang.
                                                -x,y,3/2-z  =     2_556 Check
PLAT432_ALERT_2_G Short Inter X...Y Contact  C16C     ..C26C     .       3.05 Ang.
                                                -x,y,3/2-z  =     2_556 Check
PLAT432_ALERT_2_G Short Inter X...Y Contact  C16C     ..C29C     .       3.07 Ang.
                                                -x,y,3/2-z  =     2_556 Check
PLAT432_ALERT_2_G Short Inter X...Y Contact  C16C     ..C68C     .       3.19 Ang.
                                                -x,y,3/2-z  =     2_556 Check
PLAT432_ALERT_2_G Short Inter X...Y Contact  C17B     ..C43B     .       0.95 Ang.
                                               1-x,y,3/2-z  =     2_656 Check
PLAT432_ALERT_2_G Short Inter X...Y Contact  C17B     ..C44B     .       1.17 Ang.
                                               1-x,y,3/2-z  =     2_656 Check
PLAT432_ALERT_2_G Short Inter X...Y Contact  C17B     ..C38B     .       1.19 Ang.
                                               1-x,y,3/2-z  =     2_656 Check
PLAT432_ALERT_2_G Short Inter X...Y Contact  C17B     ..C37B     .       1.48 Ang.
                                               1-x,y,3/2-z  =     2_656 Check
PLAT432_ALERT_2_G Short Inter X...Y Contact  C17B     ..C65B     .       1.48 Ang.
                                               1-x,y,3/2-z  =     2_656 Check
PLAT432_ALERT_2_G Short Inter X...Y Contact  C17B     ..C42B     .       2.35 Ang.
                                               1-x,y,3/2-z  =     2_656 Check
PLAT432_ALERT_2_G Short Inter X...Y Contact  C17B     ..C45B     .       2.53 Ang.
                                               1-x,y,3/2-z  =     2_656 Check
PLAT432_ALERT_2_G Short Inter X...Y Contact  C17B     ..C39B     .       2.57 Ang.
                                               1-x,y,3/2-z  =     2_656 Check
PLAT432_ALERT_2_G Short Inter X...Y Contact  C17B     ..C36B     .       2.90 Ang.
                                               1-x,y,3/2-z  =     2_656 Check
PLAT432_ALERT_2_G Short Inter X...Y Contact  C17B     ..C48B     .       2.92 Ang.
                                               1-x,y,3/2-z  =     2_656 Check
PLAT432_ALERT_2_G Short Inter X...Y Contact  C17C     ..C31C     .       0.51 Ang.
                                                -x,y,3/2-z  =     2_556 Check
PLAT432_ALERT_2_G Short Inter X...Y Contact  C17C     ..C35C     .       1.13 Ang.
                                                -x,y,3/2-z  =     2_556 Check
PLAT432_ALERT_2_G Short Inter X...Y Contact  C17C     ..C30C     .       1.36 Ang.
                                                -x,y,3/2-z  =     2_556 Check
PLAT432_ALERT_2_G Short Inter X...Y Contact  C17C     ..C32C     .       1.88 Ang.
```

```
                                                 -x,y,3/2-z  =      2_556 Check
PLAT432_ALERT_2_G Short Inter X...Y Contact  C17C     ..C36C      .       1.99 Ang.
                                                 -x,y,3/2-z  =      2_556 Check
PLAT432_ALERT_2_G Short Inter X...Y Contact  C17C     ..C56C      .       2.10 Ang.
                                                 -x,y,3/2-z  =      2_556 Check
PLAT432_ALERT_2_G Short Inter X...Y Contact  C17C     ..C34C      .       2.27 Ang.
                                                 -x,y,3/2-z  =      2_556 Check
PLAT432_ALERT_2_G Short Inter X...Y Contact  C17C     ..C57C      .       2.35 Ang.
                                                 -x,y,3/2-z  =      2_556 Check
PLAT432_ALERT_2_G Short Inter X...Y Contact  C17C     ..C33C      .       2.52 Ang.
                                                 -x,y,3/2-z  =      2_556 Check
PLAT432_ALERT_2_G Short Inter X...Y Contact  C17C     ..C29C      .       2.61 Ang.
                                                 -x,y,3/2-z  =      2_556 Check
PLAT432_ALERT_2_G Short Inter X...Y Contact  C17C     ..C27C      .       2.92 Ang.
                                                 -x,y,3/2-z  =      2_556 Check
PLAT432_ALERT_2_G Short Inter X...Y Contact  C17C     ..C28C      .       3.18 Ang.
                                                 -x,y,3/2-z  =      2_556 Check
PLAT432_ALERT_2_G Short Inter X...Y Contact  C18B     ..C65B      .       0.25 Ang.
                                                1-x,y,3/2-z  =      2_656 Check
PLAT432_ALERT_2_G Short Inter X...Y Contact  C18B     ..C37B      .       1.26 Ang.
                                                1-x,y,3/2-z  =      2_656 Check
PLAT432_ALERT_2_G Short Inter X...Y Contact  C18B     ..C48B      .       1.48 Ang.
                                                1-x,y,3/2-z  =      2_656 Check
PLAT432_ALERT_2_G Short Inter X...Y Contact  C18B     ..C44B      .       1.54 Ang.
                                                1-x,y,3/2-z  =      2_656 Check
PLAT432_ALERT_2_G Short Inter X...Y Contact  C18B     ..C38B      .       2.26 Ang.
                                                1-x,y,3/2-z  =      2_656 Check
PLAT432_ALERT_2_G Short Inter X...Y Contact  C18B     ..C36B      .       2.29 Ang.
                                                1-x,y,3/2-z  =      2_656 Check
PLAT432_ALERT_2_G Short Inter X...Y Contact  C18B     ..C43B      .       2.33 Ang.
                                                1-x,y,3/2-z  =      2_656 Check
PLAT432_ALERT_2_G Short Inter X...Y Contact  C18B     ..C49B      .       2.37 Ang.
                                                1-x,y,3/2-z  =      2_656 Check
PLAT432_ALERT_2_G Short Inter X...Y Contact  C18B     ..C47B      .       2.57 Ang.
                                                1-x,y,3/2-z  =      2_656 Check
PLAT432_ALERT_2_G Short Inter X...Y Contact  C18B     ..C45B      .       2.59 Ang.
                                                1-x,y,3/2-z  =      2_656 Check
PLAT432_ALERT_2_G Short Inter X...Y Contact  C18B     ..C64B      .       2.65 Ang.
                                                1-x,y,3/2-z  =      2_656 Check
PLAT432_ALERT_2_G Short Inter X...Y Contact  C18B     ..C46B      .       2.96 Ang.
                                                1-x,y,3/2-z  =      2_656 Check
PLAT432_ALERT_2_G Short Inter X...Y Contact  C18C     ..C30C      .       0.27 Ang.
                                                 -x,y,3/2-z  =      2_556 Check
PLAT432_ALERT_2_G Short Inter X...Y Contact  C18C     ..C29C      .       1.27 Ang.
                                                 -x,y,3/2-z  =      2_556 Check
PLAT432_ALERT_2_G Short Inter X...Y Contact  C18C     ..C31C      .       1.40 Ang.
                                                 -x,y,3/2-z  =      2_556 Check
PLAT432_ALERT_2_G Short Inter X...Y Contact  C18C     ..C56C      .       1.67 Ang.
                                                 -x,y,3/2-z  =      2_556 Check
PLAT432_ALERT_2_G Short Inter X...Y Contact  C18C     ..C28C      .       2.27 Ang.
                                                 -x,y,3/2-z  =      2_556 Check
PLAT432_ALERT_2_G Short Inter X...Y Contact  C18C     ..C32C      .       2.35 Ang.
                                                 -x,y,3/2-z  =      2_556 Check
PLAT432_ALERT_2_G Short Inter X...Y Contact  C18C     ..C80C      .       2.39 Ang.
                                                 -x,y,3/2-z  =      2_556 Check
PLAT432_ALERT_2_G Short Inter X...Y Contact  C18C     ..C35C      .       2.57 Ang.
                                                 -x,y,3/2-z  =      2_556 Check
```

```
PLAT432_ALERT_2_G Short Inter X...Y Contact  C18C     ..C55C     .       2.64 Ang.
                                              -x,y,3/2-z  =      2_556 Check
PLAT432_ALERT_2_G Short Inter X...Y Contact  C18C     ..C27C     .       2.68 Ang.
                                              -x,y,3/2-z  =      2_556 Check
PLAT432_ALERT_2_G Short Inter X...Y Contact  C18C     ..C57C     .       2.72 Ang.
                                              -x,y,3/2-z  =      2_556 Check
PLAT432_ALERT_2_G Short Inter X...Y Contact  C18C     ..C54C     .       2.92 Ang.
                                              -x,y,3/2-z  =      2_556 Check
PLAT432_ALERT_2_G Short Inter X...Y Contact  C18C     ..C36C     .       3.09 Ang.
                                              -x,y,3/2-z  =      2_556 Check
PLAT432_ALERT_2_G Short Inter X...Y Contact  C19B     ..C37B     .       0.95 Ang.
                                             1-x,y,3/2-z  =      2_656 Check
PLAT432_ALERT_2_G Short Inter X...Y Contact  C19B     ..C36B     .       0.95 Ang.
                                             1-x,y,3/2-z  =      2_656 Check
PLAT432_ALERT_2_G Short Inter X...Y Contact  C19B     ..C64B     .       1.59 Ang.
                                             1-x,y,3/2-z  =      2_656 Check
PLAT432_ALERT_2_G Short Inter X...Y Contact  C19B     ..C65B     .       1.60 Ang.
                                             1-x,y,3/2-z  =      2_656 Check
PLAT432_ALERT_2_G Short Inter X...Y Contact  C19B     ..C49B     .       2.07 Ang.
                                             1-x,y,3/2-z  =      2_656 Check
PLAT432_ALERT_2_G Short Inter X...Y Contact  C19B     ..C48B     .       2.12 Ang.
                                             1-x,y,3/2-z  =      2_656 Check
PLAT432_ALERT_2_G Short Inter X...Y Contact  C19B     ..C35B     .       2.34 Ang.
                                             1-x,y,3/2-z  =      2_656 Check
PLAT432_ALERT_2_G Short Inter X...Y Contact  C19B     ..C38B     .       2.34 Ang.
                                             1-x,y,3/2-z  =      2_656 Check
PLAT432_ALERT_2_G Short Inter X...Y Contact  C19B     ..C44B     .       2.79 Ang.
                                             1-x,y,3/2-z  =      2_656 Check
PLAT432_ALERT_2_G Short Inter X...Y Contact  C19B     ..C63B     .       2.92 Ang.
                                             1-x,y,3/2-z  =      2_656 Check
PLAT432_ALERT_2_G Short Inter X...Y Contact  C19B     ..C43B     .       3.10 Ang.
                                             1-x,y,3/2-z  =      2_656 Check
PLAT432_ALERT_2_G Short Inter X...Y Contact  C19B     ..C34B     .       3.15 Ang.
                                             1-x,y,3/2-z  =      2_656 Check
PLAT432_ALERT_2_G Short Inter X...Y Contact  C19B     ..C39B     .       3.17 Ang.
                                             1-x,y,3/2-z  =      2_656 Check
PLAT432_ALERT_2_G Short Inter X...Y Contact  C19C     ..C28C     .       0.90 Ang.
                                              -x,y,3/2-z  =      2_556 Check
PLAT432_ALERT_2_G Short Inter X...Y Contact  C19C     ..C29C     .       1.04 Ang.
                                              -x,y,3/2-z  =      2_556 Check
PLAT432_ALERT_2_G Short Inter X...Y Contact  C19C     ..C27C     .       1.45 Ang.
                                              -x,y,3/2-z  =      2_556 Check
PLAT432_ALERT_2_G Short Inter X...Y Contact  C19C     ..C30C     .       1.67 Ang.
                                              -x,y,3/2-z  =      2_556 Check
PLAT432_ALERT_2_G Short Inter X...Y Contact  C19C     ..C32C     .       1.90 Ang.
                                              -x,y,3/2-z  =      2_556 Check
PLAT432_ALERT_2_G Short Inter X...Y Contact  C19C     ..C31C     .       1.99 Ang.
                                              -x,y,3/2-z  =      2_556 Check
PLAT432_ALERT_2_G Short Inter X...Y Contact  C19C     ..C23C     .       2.24 Ang.
                                              -x,y,3/2-z  =      2_556 Check
PLAT432_ALERT_2_G Short Inter X...Y Contact  C19C     ..C80C     .       2.30 Ang.
                                              -x,y,3/2-z  =      2_556 Check
PLAT432_ALERT_2_G Short Inter X...Y Contact  C19C     ..C22C     .       2.79 Ang.
                                              -x,y,3/2-z  =      2_556 Check
PLAT432_ALERT_2_G Short Inter X...Y Contact  C19C     ..C26C     .       2.82 Ang.
                                              -x,y,3/2-z  =      2_556 Check
PLAT432_ALERT_2_G Short Inter X...Y Contact  C19C     ..C56C     .       2.99 Ang.
```

```
                                       -x,y,3/2-z  =      2_556 Check
PLAT432_ALERT_2_G Short Inter X...Y Contact  C19C     ..C33C     .       3.14 Ang.
                                       -x,y,3/2-z  =      2_556 Check
PLAT432_ALERT_2_G Short Inter X...Y Contact  C20B     ..C36B     .       1.10 Ang.
                                      1-x,y,3/2-z  =      2_656 Check
PLAT432_ALERT_2_G Short Inter X...Y Contact  C20B     ..C37B     .       1.13 Ang.
                                      1-x,y,3/2-z  =      2_656 Check
PLAT432_ALERT_2_G Short Inter X...Y Contact  C20B     ..C35B     .       1.48 Ang.
                                      1-x,y,3/2-z  =      2_656 Check
PLAT432_ALERT_2_G Short Inter X...Y Contact  C20B     ..C38B     .       1.62 Ang.
                                      1-x,y,3/2-z  =      2_656 Check
PLAT432_ALERT_2_G Short Inter X...Y Contact  C20B     ..C34B     .       1.87 Ang.
                                      1-x,y,3/2-z  =      2_656 Check
PLAT432_ALERT_2_G Short Inter X...Y Contact  C20B     ..C39B     .       1.95 Ang.
                                      1-x,y,3/2-z  =      2_656 Check
PLAT432_ALERT_2_G Short Inter X...Y Contact  C20B     ..C65B     .       2.51 Ang.
                                      1-x,y,3/2-z  =      2_656 Check
PLAT432_ALERT_2_G Short Inter X...Y Contact  C20B     ..C64B     .       2.51 Ang.
                                      1-x,y,3/2-z  =      2_656 Check
PLAT432_ALERT_2_G Short Inter X...Y Contact  C20B     ..C31B     .       2.85 Ang.
                                      1-x,y,3/2-z  =      2_656 Check
PLAT432_ALERT_2_G Short Inter X...Y Contact  C20B     ..C43B     .       2.87 Ang.
                                      1-x,y,3/2-z  =      2_656 Check
PLAT432_ALERT_2_G Short Inter X...Y Contact  C20B     ..C33B     .       3.20 Ang.
                                      1-x,y,3/2-z  =      2_656 Check
PLAT432_ALERT_2_G Short Inter X...Y Contact  C20C     ..C27C     .       0.65 Ang.
                                       -x,y,3/2-z  =      2_556 Check
PLAT432_ALERT_2_G Short Inter X...Y Contact  C20C     ..C32C     .       0.80 Ang.
                                       -x,y,3/2-z  =      2_556 Check
PLAT432_ALERT_2_G Short Inter X...Y Contact  C20C     ..C28C     .       1.74 Ang.
                                       -x,y,3/2-z  =      2_556 Check
PLAT432_ALERT_2_G Short Inter X...Y Contact  C20C     ..C31C     .       1.90 Ang.
                                       -x,y,3/2-z  =      2_556 Check
PLAT432_ALERT_2_G Short Inter X...Y Contact  C20C     ..C33C     .       1.91 Ang.
                                       -x,y,3/2-z  =      2_556 Check
PLAT432_ALERT_2_G Short Inter X...Y Contact  C20C     ..C26C     .       1.94 Ang.
                                       -x,y,3/2-z  =      2_556 Check
PLAT432_ALERT_2_G Short Inter X...Y Contact  C20C     ..C29C     .       2.42 Ang.
                                       -x,y,3/2-z  =      2_556 Check
PLAT432_ALERT_2_G Short Inter X...Y Contact  C20C     ..C30C     .       2.50 Ang.
                                       -x,y,3/2-z  =      2_556 Check
PLAT432_ALERT_2_G Short Inter X...Y Contact  C20C     ..C69C     .       2.63 Ang.
                                       -x,y,3/2-z  =      2_556 Check
PLAT432_ALERT_2_G Short Inter X...Y Contact  C20C     ..C68C     .       2.65 Ang.
                                       -x,y,3/2-z  =      2_556 Check
PLAT432_ALERT_2_G Short Inter X...Y Contact  C20C     ..C23C     .       2.94 Ang.
                                       -x,y,3/2-z  =      2_556 Check
PLAT432_ALERT_2_G Short Inter X...Y Contact  C20C     ..C35C     .       2.97 Ang.
                                       -x,y,3/2-z  =      2_556 Check
PLAT432_ALERT_2_G Short Inter X...Y Contact  C20C     ..C34C     .       3.05 Ang.
                                       -x,y,3/2-z  =      2_556 Check
PLAT432_ALERT_2_G Short Inter X...Y Contact  C20C     ..C25C     .       3.09 Ang.
                                       -x,y,3/2-z  =      2_556 Check
PLAT432_ALERT_2_G Short Inter X...Y Contact  C21B     ..C35B     .       0.34 Ang.
                                      1-x,y,3/2-z  =      2_656 Check
PLAT432_ALERT_2_G Short Inter X...Y Contact  C21B     ..C34B     .       1.12 Ang.
                                      1-x,y,3/2-z  =      2_656 Check
```

```
PLAT432_ALERT_2_G Short Inter X...Y Contact  C21B     ..C31B     .       1.58 Ang.
                                               1-x,y,3/2-z  =     2_656 Check
PLAT432_ALERT_2_G Short Inter X...Y Contact  C21B     ..C36B     .       1.67 Ang.
                                               1-x,y,3/2-z  =     2_656 Check
PLAT432_ALERT_2_G Short Inter X...Y Contact  C21B     ..C33B     .       2.07 Ang.
                                               1-x,y,3/2-z  =     2_656 Check
PLAT432_ALERT_2_G Short Inter X...Y Contact  C21B     ..C39B     .       2.24 Ang.
                                               1-x,y,3/2-z  =     2_656 Check
PLAT432_ALERT_2_G Short Inter X...Y Contact  C21B     ..C32B     .       2.24 Ang.
                                               1-x,y,3/2-z  =     2_656 Check
PLAT432_ALERT_2_G Short Inter X...Y Contact  C21B     ..C37B     .       2.53 Ang.
                                               1-x,y,3/2-z  =     2_656 Check
PLAT432_ALERT_2_G Short Inter X...Y Contact  C21B     ..C38B     .       2.73 Ang.
                                               1-x,y,3/2-z  =     2_656 Check
PLAT432_ALERT_2_G Short Inter X...Y Contact  C21B     ..C30B     .       2.78 Ang.
                                               1-x,y,3/2-z  =     2_656 Check
PLAT432_ALERT_2_G Short Inter X...Y Contact  C21B     ..C64B     .       2.78 Ang.
                                               1-x,y,3/2-z  =     2_656 Check
PLAT432_ALERT_2_G Short Inter X...Y Contact  C21B     ..C63B     .       3.18 Ang.
                                               1-x,y,3/2-z  =     2_656 Check
PLAT432_ALERT_2_G Short Inter X...Y Contact  C21C     ..C26C     .       0.56 Ang.
                                                -x,y,3/2-z  =     2_556 Check
PLAT432_ALERT_2_G Short Inter X...Y Contact  C21C     ..C27C     .       1.07 Ang.
                                                -x,y,3/2-z  =     2_556 Check
PLAT432_ALERT_2_G Short Inter X...Y Contact  C21C     ..C68C     .       1.44 Ang.
                                                -x,y,3/2-z  =     2_556 Check
PLAT432_ALERT_2_G Short Inter X...Y Contact  C21C     ..C25C     .       1.91 Ang.
                                                -x,y,3/2-z  =     2_556 Check
PLAT432_ALERT_2_G Short Inter X...Y Contact  C21C     ..C32C     .       1.95 Ang.
                                                -x,y,3/2-z  =     2_556 Check
PLAT432_ALERT_2_G Short Inter X...Y Contact  C21C     ..C69C     .       2.12 Ang.
                                                -x,y,3/2-z  =     2_556 Check
PLAT432_ALERT_2_G Short Inter X...Y Contact  C21C     ..C28C     .       2.29 Ang.
                                                -x,y,3/2-z  =     2_556 Check
PLAT432_ALERT_2_G Short Inter X...Y Contact  C21C     ..C33C     .       2.30 Ang.
                                                -x,y,3/2-z  =     2_556 Check
PLAT432_ALERT_2_G Short Inter X...Y Contact  C21C     ..C67C     .       2.59 Ang.
                                                -x,y,3/2-z  =     2_556 Check
PLAT432_ALERT_2_G Short Inter X...Y Contact  C21C     ..C66C     .       2.78 Ang.
                                                -x,y,3/2-z  =     2_556 Check
PLAT432_ALERT_2_G Short Inter X...Y Contact  C21C     ..C24C     .       2.80 Ang.
                                                -x,y,3/2-z  =     2_556 Check
PLAT432_ALERT_2_G Short Inter X...Y Contact  C21C     ..C23C     .       2.90 Ang.
                                                -x,y,3/2-z  =     2_556 Check
PLAT432_ALERT_2_G Short Inter X...Y Contact  C22B     ..C31B     .       0.66 Ang.
                                               1-x,y,3/2-z  =     2_656 Check
PLAT432_ALERT_2_G Short Inter X...Y Contact  C22B     ..C35B     .       1.18 Ang.
                                               1-x,y,3/2-z  =     2_656 Check
PLAT432_ALERT_2_G Short Inter X...Y Contact  C22B     ..C30B     .       1.40 Ang.
                                               1-x,y,3/2-z  =     2_656 Check
PLAT432_ALERT_2_G Short Inter X...Y Contact  C22B     ..C36B     .       1.96 Ang.
                                               1-x,y,3/2-z  =     2_656 Check
PLAT432_ALERT_2_G Short Inter X...Y Contact  C22B     ..C32B     .       2.03 Ang.
                                               1-x,y,3/2-z  =     2_656 Check
PLAT432_ALERT_2_G Short Inter X...Y Contact  C22B     ..C63B     .       2.04 Ang.
                                               1-x,y,3/2-z  =     2_656 Check
PLAT432_ALERT_2_G Short Inter X...Y Contact  C22B     ..C64B     .       2.26 Ang.
```

```
                                           1-x,y,3/2-z  =      2_656 Check
PLAT432_ALERT_2_G Short Inter X...Y Contact  C22B     ..C34B     .       2.41 Ang.
                                           1-x,y,3/2-z  =      2_656 Check
PLAT432_ALERT_2_G Short Inter X...Y Contact  C22B     ..C29B     .       2.71 Ang.
                                           1-x,y,3/2-z  =      2_656 Check
PLAT432_ALERT_2_G Short Inter X...Y Contact  C22B     ..C33B     .       2.73 Ang.
                                           1-x,y,3/2-z  =      2_656 Check
PLAT432_ALERT_2_G Short Inter X...Y Contact  C22B     ..C27B     .       3.13 Ang.
                                           1-x,y,3/2-z  =      2_656 Check
PLAT432_ALERT_2_G Short Inter X...Y Contact  C22C     ..C25C     .       0.71 Ang.
                                            -x,y,3/2-z  =      2_556 Check
PLAT432_ALERT_2_G Short Inter X...Y Contact  C22C     ..C26C     .       1.02 Ang.
                                            -x,y,3/2-z  =      2_556 Check
PLAT432_ALERT_2_G Short Inter X...Y Contact  C22C     ..C24C     .       1.47 Ang.
                                            -x,y,3/2-z  =      2_556 Check
PLAT432_ALERT_2_G Short Inter X...Y Contact  C22C     ..C27C     .       1.85 Ang.
                                            -x,y,3/2-z  =      2_556 Check
PLAT432_ALERT_2_G Short Inter X...Y Contact  C22C     ..C23C     .       2.06 Ang.
                                            -x,y,3/2-z  =      2_556 Check
PLAT432_ALERT_2_G Short Inter X...Y Contact  C22C     ..C66C     .       2.08 Ang.
                                            -x,y,3/2-z  =      2_556 Check
PLAT432_ALERT_2_G Short Inter X...Y Contact  C22C     ..C28C     .       2.22 Ang.
                                            -x,y,3/2-z  =      2_556 Check
PLAT432_ALERT_2_G Short Inter X...Y Contact  C22C     ..C68C     .       2.22 Ang.
                                            -x,y,3/2-z  =      2_556 Check
PLAT432_ALERT_2_G Short Inter X...Y Contact  C22C     ..C67C     .       2.65 Ang.
                                            -x,y,3/2-z  =      2_556 Check
PLAT432_ALERT_2_G Short Inter X...Y Contact  C22C     ..C32C     .       3.16 Ang.
                                            -x,y,3/2-z  =      2_556 Check
PLAT432_ALERT_2_G Short Inter X...Y Contact  C22C     ..C65C     .       3.19 Ang.
                                            -x,y,3/2-z  =      2_556 Check
PLAT432_ALERT_2_G Short Inter X...Y Contact  C23B     ..C63B     .       0.76 Ang.
                                           1-x,y,3/2-z  =      2_656 Check
PLAT432_ALERT_2_G Short Inter X...Y Contact  C23B     ..C64B     .       1.18 Ang.
                                           1-x,y,3/2-z  =      2_656 Check
PLAT432_ALERT_2_G Short Inter X...Y Contact  C23B     ..C30B     .       1.35 Ang.
                                           1-x,y,3/2-z  =      2_656 Check
PLAT432_ALERT_2_G Short Inter X...Y Contact  C23B     ..C36B     .       1.87 Ang.
                                           1-x,y,3/2-z  =      2_656 Check
PLAT432_ALERT_2_G Short Inter X...Y Contact  C23B     ..C31B     .       1.94 Ang.
                                           1-x,y,3/2-z  =      2_656 Check
PLAT432_ALERT_2_G Short Inter X...Y Contact  C23B     ..C62B     .       2.13 Ang.
                                           1-x,y,3/2-z  =      2_656 Check
PLAT432_ALERT_2_G Short Inter X...Y Contact  C23B     ..C35B     .       2.16 Ang.
                                           1-x,y,3/2-z  =      2_656 Check
PLAT432_ALERT_2_G Short Inter X...Y Contact  C23B     ..C49B     .       2.42 Ang.
                                           1-x,y,3/2-z  =      2_656 Check
PLAT432_ALERT_2_G Short Inter X...Y Contact  C23B     ..C29B     .       2.68 Ang.
                                           1-x,y,3/2-z  =      2_656 Check
PLAT432_ALERT_2_G Short Inter X...Y Contact  C23B     ..C50B     .       2.79 Ang.
                                           1-x,y,3/2-z  =      2_656 Check
PLAT432_ALERT_2_G Short Inter X...Y Contact  C23B     ..C61B     .       3.19 Ang.
                                           1-x,y,3/2-z  =      2_656 Check
PLAT432_ALERT_2_G Short Inter X...Y Contact  C23C     ..C23C     .       0.71 Ang.
                                            -x,y,3/2-z  =      2_556 Check
PLAT432_ALERT_2_G Short Inter X...Y Contact  C23C     ..C24C     .       0.92 Ang.
                                            -x,y,3/2-z  =      2_556 Check
```

```
PLAT432_ALERT_2_G Short Inter X...Y Contact  C23C     ..C28C     .       1.64 Ang.
                                               -x,y,3/2-z  =     2_556 Check
PLAT432_ALERT_2_G Short Inter X...Y Contact  C23C     ..C25C     .       1.77 Ang.
                                               -x,y,3/2-z  =     2_556 Check
PLAT432_ALERT_2_G Short Inter X...Y Contact  C23C     ..C27C     .       2.26 Ang.
                                               -x,y,3/2-z  =     2_556 Check
PLAT432_ALERT_2_G Short Inter X...Y Contact  C23C     ..C26C     .       2.27 Ang.
                                               -x,y,3/2-z  =     2_556 Check
PLAT432_ALERT_2_G Short Inter X...Y Contact  C23C     ..C29C     .       2.79 Ang.
                                               -x,y,3/2-z  =     2_556 Check
PLAT432_ALERT_2_G Short Inter X...Y Contact  C23C     ..C80C     .       2.95 Ang.
                                               -x,y,3/2-z  =     2_556 Check
PLAT432_ALERT_2_G Short Inter X...Y Contact  C23C     ..C66C     .       3.08 Ang.
                                               -x,y,3/2-z  =     2_556 Check
PLAT432_ALERT_2_G Short Inter X...Y Contact  C24B     ..C64B     .       0.33 Ang.
                                              1-x,y,3/2-z  =     2_656 Check
PLAT432_ALERT_2_G Short Inter X...Y Contact  C24B     ..C49B     .       1.20 Ang.
                                              1-x,y,3/2-z  =     2_656 Check
PLAT432_ALERT_2_G Short Inter X...Y Contact  C24B     ..C36B     .       1.43 Ang.
                                              1-x,y,3/2-z  =     2_656 Check
PLAT432_ALERT_2_G Short Inter X...Y Contact  C24B     ..C63B     .       1.66 Ang.
                                              1-x,y,3/2-z  =     2_656 Check
PLAT432_ALERT_2_G Short Inter X...Y Contact  C24B     ..C48B     .       2.18 Ang.
                                              1-x,y,3/2-z  =     2_656 Check
PLAT432_ALERT_2_G Short Inter X...Y Contact  C24B     ..C50B     .       2.21 Ang.
                                              1-x,y,3/2-z  =     2_656 Check
PLAT432_ALERT_2_G Short Inter X...Y Contact  C24B     ..C37B     .       2.32 Ang.
                                              1-x,y,3/2-z  =     2_656 Check
PLAT432_ALERT_2_G Short Inter X...Y Contact  C24B     ..C62B     .       2.38 Ang.
                                              1-x,y,3/2-z  =     2_656 Check
PLAT432_ALERT_2_G Short Inter X...Y Contact  C24B     ..C65B     .       2.57 Ang.
                                              1-x,y,3/2-z  =     2_656 Check
PLAT432_ALERT_2_G Short Inter X...Y Contact  C24B     ..C35B     .       2.60 Ang.
                                              1-x,y,3/2-z  =     2_656 Check
PLAT432_ALERT_2_G Short Inter X...Y Contact  C24B     ..C30B     .       2.73 Ang.
                                              1-x,y,3/2-z  =     2_656 Check
PLAT432_ALERT_2_G Short Inter X...Y Contact  C24B     ..C31B     .       3.05 Ang.
                                              1-x,y,3/2-z  =     2_656 Check
PLAT432_ALERT_2_G Short Inter X...Y Contact  C24C     ..C28C     .       0.80 Ang.
                                               -x,y,3/2-z  =     2_556 Check
PLAT432_ALERT_2_G Short Inter X...Y Contact  C24C     ..C29C     .       1.39 Ang.
                                               -x,y,3/2-z  =     2_556 Check
PLAT432_ALERT_2_G Short Inter X...Y Contact  C24C     ..C80C     .       1.69 Ang.
                                               -x,y,3/2-z  =     2_556 Check
PLAT432_ALERT_2_G Short Inter X...Y Contact  C24C     ..C27C     .       2.14 Ang.
                                               -x,y,3/2-z  =     2_556 Check
PLAT432_ALERT_2_G Short Inter X...Y Contact  C24C     ..C24C     .       2.28 Ang.
                                               -x,y,3/2-z  =     2_556 Check
PLAT432_ALERT_2_G Short Inter X...Y Contact  C24C     ..C30C     .       2.73 Ang.
                                               -x,y,3/2-z  =     2_556 Check
PLAT432_ALERT_2_G Short Inter X...Y Contact  C24C     ..C26C     .       2.97 Ang.
                                               -x,y,3/2-z  =     2_556 Check
PLAT432_ALERT_2_G Short Inter X...Y Contact  C24C     ..C25C     .       3.02 Ang.
                                               -x,y,3/2-z  =     2_556 Check
PLAT432_ALERT_2_G Short Inter X...Y Contact  C24C     ..C54C     .       3.04 Ang.
                                               -x,y,3/2-z  =     2_556 Check
PLAT432_ALERT_2_G Short Inter X...Y Contact  C24C     ..C32C     .       3.16 Ang.
```

```
                                              -x,y,3/2-z  =     2_556 Check
PLAT432_ALERT_2_G Short Inter X...Y Contact  C25B     ..C49B     .       0.47 Ang.
                                             1-x,y,3/2-z  =     2_656 Check
PLAT432_ALERT_2_G Short Inter X...Y Contact  C25B     ..C50B     .       0.98 Ang.
                                             1-x,y,3/2-z  =     2_656 Check
PLAT432_ALERT_2_G Short Inter X...Y Contact  C25B     ..C48B     .       1.63 Ang.
                                             1-x,y,3/2-z  =     2_656 Check
PLAT432_ALERT_2_G Short Inter X...Y Contact  C25B     ..C64B     .       1.67 Ang.
                                             1-x,y,3/2-z  =     2_656 Check
PLAT432_ALERT_2_G Short Inter X...Y Contact  C25B     ..C62B     .       1.98 Ang.
                                             1-x,y,3/2-z  =     2_656 Check
PLAT432_ALERT_2_G Short Inter X...Y Contact  C25B     ..C51B     .       2.04 Ang.
                                             1-x,y,3/2-z  =     2_656 Check
PLAT432_ALERT_2_G Short Inter X...Y Contact  C25B     ..C63B     .       2.25 Ang.
                                             1-x,y,3/2-z  =     2_656 Check
PLAT432_ALERT_2_G Short Inter X...Y Contact  C25B     ..C47B     .       2.41 Ang.
                                             1-x,y,3/2-z  =     2_656 Check
PLAT432_ALERT_2_G Short Inter X...Y Contact  C25B     ..C52B     .       2.57 Ang.
                                             1-x,y,3/2-z  =     2_656 Check
PLAT432_ALERT_2_G Short Inter X...Y Contact  C25B     ..C65B     .       2.82 Ang.
                                             1-x,y,3/2-z  =     2_656 Check
PLAT432_ALERT_2_G Short Inter X...Y Contact  C25B     ..C36B     .       2.84 Ang.
                                             1-x,y,3/2-z  =     2_656 Check
PLAT432_ALERT_2_G Short Inter X...Y Contact  C25C     ..C80C     .       0.72 Ang.
                                              -x,y,3/2-z  =     2_556 Check
PLAT432_ALERT_2_G Short Inter X...Y Contact  C25C     ..C29C     .       1.75 Ang.
                                              -x,y,3/2-z  =     2_556 Check
PLAT432_ALERT_2_G Short Inter X...Y Contact  C25C     ..C54C     .       1.92 Ang.
                                              -x,y,3/2-z  =     2_556 Check
PLAT432_ALERT_2_G Short Inter X...Y Contact  C25C     ..C28C     .       2.17 Ang.
                                              -x,y,3/2-z  =     2_556 Check
PLAT432_ALERT_2_G Short Inter X...Y Contact  C25C     ..C79C     .       2.61 Ang.
                                              -x,y,3/2-z  =     2_556 Check
PLAT432_ALERT_2_G Short Inter X...Y Contact  C25C     ..C53C     .       2.63 Ang.
                                              -x,y,3/2-z  =     2_556 Check
PLAT432_ALERT_2_G Short Inter X...Y Contact  C25C     ..C30C     .       3.01 Ang.
                                              -x,y,3/2-z  =     2_556 Check
PLAT432_ALERT_2_G Short Inter X...Y Contact  C25C     ..C55C     .       3.08 Ang.
                                              -x,y,3/2-z  =     2_556 Check
PLAT432_ALERT_2_G Short Inter X...Y Contact  C26B     ..C50B     .       0.51 Ang.
                                             1-x,y,3/2-z  =     2_656 Check
PLAT432_ALERT_2_G Short Inter X...Y Contact  C26B     ..C62B     .       1.13 Ang.
                                             1-x,y,3/2-z  =     2_656 Check
PLAT432_ALERT_2_G Short Inter X...Y Contact  C26B     ..C51B     .       1.38 Ang.
                                             1-x,y,3/2-z  =     2_656 Check
PLAT432_ALERT_2_G Short Inter X...Y Contact  C26B     ..C49B     .       1.87 Ang.
                                             1-x,y,3/2-z  =     2_656 Check
PLAT432_ALERT_2_G Short Inter X...Y Contact  C26B     ..C61B     .       1.99 Ang.
                                             1-x,y,3/2-z  =     2_656 Check
PLAT432_ALERT_2_G Short Inter X...Y Contact  C26B     ..C56B     .       2.12 Ang.
                                             1-x,y,3/2-z  =     2_656 Check
PLAT432_ALERT_2_G Short Inter X...Y Contact  C26B     ..C63B     .       2.25 Ang.
                                             1-x,y,3/2-z  =     2_656 Check
PLAT432_ALERT_2_G Short Inter X...Y Contact  C26B     ..C57B     .       2.34 Ang.
                                             1-x,y,3/2-z  =     2_656 Check
PLAT432_ALERT_2_G Short Inter X...Y Contact  C26B     ..C64B     .       2.56 Ang.
                                             1-x,y,3/2-z  =     2_656 Check
```

```
PLAT432_ALERT_2_G Short Inter X...Y Contact  C26B     ..C52B     .       2.63 Ang.
                                               1-x,y,3/2-z  =     2_656 Check
PLAT432_ALERT_2_G Short Inter X...Y Contact  C26B     ..C48B     .       2.91 Ang.
                                               1-x,y,3/2-z  =     2_656 Check
PLAT432_ALERT_2_G Short Inter X...Y Contact  C26B     ..C47B     .       3.17 Ang.
                                               1-x,y,3/2-z  =     2_656 Check
PLAT432_ALERT_2_G Short Inter X...Y Contact  C26C     ..C79C     .       1.56 Ang.
                                                -x,y,3/2-z  =     2_556 Check
PLAT432_ALERT_2_G Short Inter X...Y Contact  C26C     ..C80C     .       1.97 Ang.
                                                -x,y,3/2-z  =     2_556 Check
PLAT432_ALERT_2_G Short Inter X...Y Contact  C26C     ..C53C     .       2.31 Ang.
                                                -x,y,3/2-z  =     2_556 Check
PLAT432_ALERT_2_G Short Inter X...Y Contact  C26C     ..C54C     .       2.47 Ang.
                                                -x,y,3/2-z  =     2_556 Check
PLAT432_ALERT_2_G Short Inter X...Y Contact  C26C     ..C78C     .       2.81 Ang.
                                                -x,y,3/2-z  =     2_556 Check
PLAT432_ALERT_2_G Short Inter X...Y Contact  C26C     ..C29C     .       3.10 Ang.
                                                -x,y,3/2-z  =     2_556 Check
PLAT432_ALERT_2_G Short Inter X...Y Contact  C27B     ..C62B     .       0.62 Ang.
                                               1-x,y,3/2-z  =     2_656 Check
PLAT432_ALERT_2_G Short Inter X...Y Contact  C27B     ..C61B     .       0.85 Ang.
                                               1-x,y,3/2-z  =     2_656 Check
PLAT432_ALERT_2_G Short Inter X...Y Contact  C27B     ..C63B     .       1.76 Ang.
                                               1-x,y,3/2-z  =     2_656 Check
PLAT432_ALERT_2_G Short Inter X...Y Contact  C27B     ..C50B     .       1.82 Ang.
                                               1-x,y,3/2-z  =     2_656 Check
PLAT432_ALERT_2_G Short Inter X...Y Contact  C27B     ..C60B     .       1.93 Ang.
                                               1-x,y,3/2-z  =     2_656 Check
PLAT432_ALERT_2_G Short Inter X...Y Contact  C27B     ..C57B     .       1.99 Ang.
                                               1-x,y,3/2-z  =     2_656 Check
PLAT432_ALERT_2_G Short Inter X...Y Contact  C27B     ..C30B     .       2.50 Ang.
                                               1-x,y,3/2-z  =     2_656 Check
PLAT432_ALERT_2_G Short Inter X...Y Contact  C27B     ..C29B     .       2.52 Ang.
                                               1-x,y,3/2-z  =     2_656 Check
PLAT432_ALERT_2_G Short Inter X...Y Contact  C27B     ..C51B     .       2.58 Ang.
                                               1-x,y,3/2-z  =     2_656 Check
PLAT432_ALERT_2_G Short Inter X...Y Contact  C27B     ..C56B     .       2.63 Ang.
                                               1-x,y,3/2-z  =     2_656 Check
PLAT432_ALERT_2_G Short Inter X...Y Contact  C27B     ..C64B     .       2.81 Ang.
                                               1-x,y,3/2-z  =     2_656 Check
PLAT432_ALERT_2_G Short Inter X...Y Contact  C27B     ..C49B     .       2.82 Ang.
                                               1-x,y,3/2-z  =     2_656 Check
PLAT432_ALERT_2_G Short Inter X...Y Contact  C27B     ..C58B     .       3.19 Ang.
                                               1-x,y,3/2-z  =     2_656 Check
PLAT432_ALERT_2_G Short Inter X...Y Contact  C27C     ..C79C     .       2.39 Ang.
                                                -x,y,3/2-z  =     2_556 Check
PLAT432_ALERT_2_G Short Inter X...Y Contact  C27C     ..C78C     .       3.08 Ang.
                                                -x,y,3/2-z  =     2_556 Check
PLAT432_ALERT_2_G Short Inter X...Y Contact  C27C     ..C80C     .       3.16 Ang.
                                                -x,y,3/2-z  =     2_556 Check
PLAT432_ALERT_2_G Short Inter X...Y Contact  C28B     ..C63B     .       0.87 Ang.
                                               1-x,y,3/2-z  =     2_656 Check
PLAT432_ALERT_2_G Short Inter X...Y Contact  C28B     ..C30B     .       1.12 Ang.
                                               1-x,y,3/2-z  =     2_656 Check
PLAT432_ALERT_2_G Short Inter X...Y Contact  C28B     ..C62B     .       1.39 Ang.
                                               1-x,y,3/2-z  =     2_656 Check
PLAT432_ALERT_2_G Short Inter X...Y Contact  C28B     ..C29B     .       1.68 Ang.
```

```
                                             1-x,y,3/2-z  =      2_656 Check
PLAT432_ALERT_2_G Short Inter X...Y Contact  C28B     ..C61B     .       1.96 Ang.
                                             1-x,y,3/2-z  =      2_656 Check
PLAT432_ALERT_2_G Short Inter X...Y Contact  C28B     ..C60B     .       2.03 Ang.
                                             1-x,y,3/2-z  =      2_656 Check
PLAT432_ALERT_2_G Short Inter X...Y Contact  C28B     ..C64B     .       2.29 Ang.
                                             1-x,y,3/2-z  =      2_656 Check
PLAT432_ALERT_2_G Short Inter X...Y Contact  C28B     ..C31B     .       2.52 Ang.
                                             1-x,y,3/2-z  =      2_656 Check
PLAT432_ALERT_2_G Short Inter X...Y Contact  C28B     ..C50B     .       2.65 Ang.
                                             1-x,y,3/2-z  =      2_656 Check
PLAT432_ALERT_2_G Short Inter X...Y Contact  C28B     ..C49B     .       3.01 Ang.
                                             1-x,y,3/2-z  =      2_656 Check
PLAT432_ALERT_2_G Short Inter X...Y Contact  C28B     ..C28B     .       3.01 Ang.
                                             1-x,y,3/2-z  =      2_656 Check
PLAT432_ALERT_2_G Short Inter X...Y Contact  C28C     ..C28C     .       2.95 Ang.
                                              -x,y,3/2-z  =      2_556 Check
PLAT432_ALERT_2_G Short Inter X...Y Contact  C28C     ..C66C     .       2.98 Ang.
                                              -x,y,3/2-z  =      2_556 Check
PLAT432_ALERT_2_G Short Inter X...Y Contact  C28C     ..C65C     .       3.08 Ang.
                                              -x,y,3/2-z  =      2_556 Check
PLAT432_ALERT_2_G Short Inter X...Y Contact  C29B     ..C29B     .       0.44 Ang.
                                             1-x,y,3/2-z  =      2_656 Check
PLAT432_ALERT_2_G Short Inter X...Y Contact  C29B     ..C30B     .       1.02 Ang.
                                             1-x,y,3/2-z  =      2_656 Check
PLAT432_ALERT_2_G Short Inter X...Y Contact  C29B     ..C60B     .       1.72 Ang.
                                             1-x,y,3/2-z  =      2_656 Check
PLAT432_ALERT_2_G Short Inter X...Y Contact  C29B     ..C63B     .       2.12 Ang.
                                             1-x,y,3/2-z  =      2_656 Check
PLAT432_ALERT_2_G Short Inter X...Y Contact  C29B     ..C31B     .       2.15 Ang.
                                             1-x,y,3/2-z  =      2_656 Check
PLAT432_ALERT_2_G Short Inter X...Y Contact  C29B     ..C61B     .       2.57 Ang.
                                             1-x,y,3/2-z  =      2_656 Check
PLAT432_ALERT_2_G Short Inter X...Y Contact  C29B     ..C32B     .       2.67 Ang.
                                             1-x,y,3/2-z  =      2_656 Check
PLAT432_ALERT_2_G Short Inter X...Y Contact  C29B     ..C62B     .       2.68 Ang.
                                             1-x,y,3/2-z  =      2_656 Check
PLAT432_ALERT_2_G Short Inter X...Y Contact  C29C     ..C65C     .       1.73 Ang.
                                              -x,y,3/2-z  =      2_556 Check
PLAT432_ALERT_2_G Short Inter X...Y Contact  C29C     ..C66C     .       1.87 Ang.
                                              -x,y,3/2-z  =      2_556 Check
PLAT432_ALERT_2_G Short Inter X...Y Contact  C29C     ..C64C     .       3.11 Ang.
                                              -x,y,3/2-z  =      2_556 Check
PLAT432_ALERT_2_G Short Inter X...Y Contact  C30B     ..C60B     .       1.19 Ang.
                                             1-x,y,3/2-z  =      2_656 Check
PLAT432_ALERT_2_G Short Inter X...Y Contact  C30B     ..C30B     .       2.42 Ang.
                                             1-x,y,3/2-z  =      2_656 Check
PLAT432_ALERT_2_G Short Inter X...Y Contact  C30B     ..C61B     .       2.56 Ang.
                                             1-x,y,3/2-z  =      2_656 Check
PLAT432_ALERT_2_G Short Inter X...Y Contact  C30C     ..C65C     .       1.37 Ang.
                                              -x,y,3/2-z  =      2_556 Check
PLAT432_ALERT_2_G Short Inter X...Y Contact  C30C     ..C64C     .       2.29 Ang.
                                              -x,y,3/2-z  =      2_556 Check
PLAT432_ALERT_2_G Short Inter X...Y Contact  C30C     ..C66C     .       2.49 Ang.
                                              -x,y,3/2-z  =      2_556 Check
PLAT432_ALERT_2_G Short Inter X...Y Contact  C31B     ..C60B     .       0.94 Ang.
                                             1-x,y,3/2-z  =      2_656 Check
```

```
PLAT432_ALERT_2_G Short Inter X...Y Contact  C31B     ..C61B     .       1.81 Ang.
                                               1-x,y,3/2-z  =     2_656 Check
PLAT432_ALERT_2_G Short Inter X...Y Contact  C31B     ..C58B     .       2.18 Ang.
                                               1-x,y,3/2-z  =     2_656 Check
PLAT432_ALERT_2_G Short Inter X...Y Contact  C31B     ..C57B     .       2.25 Ang.
                                               1-x,y,3/2-z  =     2_656 Check
PLAT432_ALERT_2_G Short Inter X...Y Contact  C31B     ..C62B     .       3.10 Ang.
                                               1-x,y,3/2-z  =     2_656 Check
PLAT432_ALERT_2_G Short Inter X...Y Contact  C31C     ..C65C     .       2.71 Ang.
                                                -x,y,3/2-z  =     2_556 Check
PLAT432_ALERT_2_G Short Inter X...Y Contact  C32B     ..C61B     .       0.68 Ang.
                                               1-x,y,3/2-z  =     2_656 Check
PLAT432_ALERT_2_G Short Inter X...Y Contact  C32B     ..C57B     .       1.05 Ang.
                                               1-x,y,3/2-z  =     2_656 Check
PLAT432_ALERT_2_G Short Inter X...Y Contact  C32B     ..C60B     .       1.40 Ang.
                                               1-x,y,3/2-z  =     2_656 Check
PLAT432_ALERT_2_G Short Inter X...Y Contact  C32B     ..C58B     .       1.84 Ang.
                                               1-x,y,3/2-z  =     2_656 Check
PLAT432_ALERT_2_G Short Inter X...Y Contact  C32B     ..C62B     .       2.09 Ang.
                                               1-x,y,3/2-z  =     2_656 Check
PLAT432_ALERT_2_G Short Inter X...Y Contact  C32B     ..C56B     .       2.33 Ang.
                                               1-x,y,3/2-z  =     2_656 Check
PLAT432_ALERT_2_G Short Inter X...Y Contact  C32B     ..C50B     .       2.95 Ang.
                                               1-x,y,3/2-z  =     2_656 Check
PLAT432_ALERT_2_G Short Inter X...Y Contact  C32B     ..C59B     .       3.03 Ang.
                                               1-x,y,3/2-z  =     2_656 Check
PLAT432_ALERT_2_G Short Inter X...Y Contact  C32B     ..C51B     .       3.05 Ang.
                                               1-x,y,3/2-z  =     2_656 Check
PLAT432_ALERT_2_G Short Inter X...Y Contact  C32B     ..C63B     .       3.09 Ang.
                                               1-x,y,3/2-z  =     2_656 Check
PLAT432_ALERT_2_G Short Inter X...Y Contact  C32C     ..C78C     .       2.51 Ang.
                                                -x,y,3/2-z  =     2_556 Check
PLAT432_ALERT_2_G Short Inter X...Y Contact  C32C     ..C79C     .       2.55 Ang.
                                                -x,y,3/2-z  =     2_556 Check
PLAT432_ALERT_2_G Short Inter X...Y Contact  C33B     ..C57B     .       0.66 Ang.
                                               1-x,y,3/2-z  =     2_656 Check
PLAT432_ALERT_2_G Short Inter X...Y Contact  C33B     ..C58B     .       0.82 Ang.
                                               1-x,y,3/2-z  =     2_656 Check
PLAT432_ALERT_2_G Short Inter X...Y Contact  C33B     ..C56B     .       1.53 Ang.
                                               1-x,y,3/2-z  =     2_656 Check
PLAT432_ALERT_2_G Short Inter X...Y Contact  C33B     ..C59B     .       1.66 Ang.
                                               1-x,y,3/2-z  =     2_656 Check
PLAT432_ALERT_2_G Short Inter X...Y Contact  C33B     ..C55B     .       1.94 Ang.
                                               1-x,y,3/2-z  =     2_656 Check
PLAT432_ALERT_2_G Short Inter X...Y Contact  C33B     ..C61B     .       1.95 Ang.
                                               1-x,y,3/2-z  =     2_656 Check
PLAT432_ALERT_2_G Short Inter X...Y Contact  C33B     ..C60B     .       2.71 Ang.
                                               1-x,y,3/2-z  =     2_656 Check
PLAT432_ALERT_2_G Short Inter X...Y Contact  C33B     ..C51B     .       2.78 Ang.
                                               1-x,y,3/2-z  =     2_656 Check
PLAT432_ALERT_2_G Short Inter X...Y Contact  C33B     ..C62B     .       3.06 Ang.
                                               1-x,y,3/2-z  =     2_656 Check
PLAT432_ALERT_2_G Short Inter X...Y Contact  C33C     ..C78C     .       1.35 Ang.
                                                -x,y,3/2-z  =     2_556 Check
PLAT432_ALERT_2_G Short Inter X...Y Contact  C33C     ..C79C     .       2.04 Ang.
                                                -x,y,3/2-z  =     2_556 Check
PLAT432_ALERT_2_G Short Inter X...Y Contact  C33C     ..C77C     .       2.60 Ang.
```

```
                                             -x,y,3/2-z  =      2_556 Check
PLAT432_ALERT_2_G Short Inter X...Y Contact  C33C     ..C75C     .       3.08 Ang.
                                             -x,y,3/2-z  =      2_556 Check
PLAT432_ALERT_2_G Short Inter X...Y Contact  C34B     ..C58B     .       0.66 Ang.
                                            1-x,y,3/2-z  =      2_656 Check
PLAT432_ALERT_2_G Short Inter X...Y Contact  C34B     ..C59B     .       1.37 Ang.
                                            1-x,y,3/2-z  =      2_656 Check
PLAT432_ALERT_2_G Short Inter X...Y Contact  C34B     ..C57B     .       2.04 Ang.
                                            1-x,y,3/2-z  =      2_656 Check
PLAT432_ALERT_2_G Short Inter X...Y Contact  C34B     ..C55B     .       2.53 Ang.
                                            1-x,y,3/2-z  =      2_656 Check
PLAT432_ALERT_2_G Short Inter X...Y Contact  C34B     ..C56B     .       2.81 Ang.
                                            1-x,y,3/2-z  =      2_656 Check
PLAT432_ALERT_2_G Short Inter X...Y Contact  C34B     ..C61B     .       2.98 Ang.
                                            1-x,y,3/2-z  =      2_656 Check
PLAT432_ALERT_2_G Short Inter X...Y Contact  C34B     ..C60B     .       3.12 Ang.
                                            1-x,y,3/2-z  =      2_656 Check
PLAT432_ALERT_2_G Short Inter X...Y Contact  C34C     ..C75C     .       2.03 Ang.
                                             -x,y,3/2-z  =      2_556 Check
PLAT432_ALERT_2_G Short Inter X...Y Contact  C34C     ..C78C     .       2.25 Ang.
                                             -x,y,3/2-z  =      2_556 Check
PLAT432_ALERT_2_G Short Inter X...Y Contact  C34C     ..C76C     .       2.85 Ang.
                                             -x,y,3/2-z  =      2_556 Check
PLAT432_ALERT_2_G Short Inter X...Y Contact  C34C     ..C77C     .       2.91 Ang.
                                             -x,y,3/2-z  =      2_556 Check
PLAT432_ALERT_2_G Short Inter X...Y Contact  C35B     ..C58B     .       1.74 Ang.
                                            1-x,y,3/2-z  =      2_656 Check
PLAT432_ALERT_2_G Short Inter X...Y Contact  C35B     ..C60B     .       2.37 Ang.
                                            1-x,y,3/2-z  =      2_656 Check
PLAT432_ALERT_2_G Short Inter X...Y Contact  C35B     ..C57B     .       2.67 Ang.
                                            1-x,y,3/2-z  =      2_656 Check
PLAT432_ALERT_2_G Short Inter X...Y Contact  C35B     ..C59B     .       2.81 Ang.
                                            1-x,y,3/2-z  =      2_656 Check
PLAT432_ALERT_2_G Short Inter X...Y Contact  C35B     ..C61B     .       2.94 Ang.
                                            1-x,y,3/2-z  =      2_656 Check
PLAT432_ALERT_2_G Short Inter X...Y Contact  C35C     ..C75C     .       3.15 Ang.
                                             -x,y,3/2-z  =      2_556 Check
PLAT432_ALERT_2_G Short Inter X...Y Contact  C36B     ..C58B     .       3.03 Ang.
                                            1-x,y,3/2-z  =      2_656 Check
PLAT432_ALERT_2_G Short Inter X...Y Contact  C36C     ..C64C     .       2.76 Ang.
                                             -x,y,3/2-z  =      2_556 Check
PLAT432_ALERT_2_G Short Inter X...Y Contact  C37B     ..C73B     .       2.64 Ang.
                                            1-x,y,3/2-z  =      2_656 Check
PLAT432_ALERT_2_G Short Inter X...Y Contact  C37C     ..C75C     .       2.73 Ang.
                                             -x,y,3/2-z  =      2_556 Check
PLAT432_ALERT_2_G Short Inter X...Y Contact  C38B     ..C59B     .       2.68 Ang.
                                            1-x,y,3/2-z  =      2_656 Check
PLAT432_ALERT_2_G Short Inter X...Y Contact  C38B     ..C58B     .       3.11 Ang.
                                            1-x,y,3/2-z  =      2_656 Check
PLAT432_ALERT_2_G Short Inter X...Y Contact  C38C     ..C75C     .       1.37 Ang.
                                             -x,y,3/2-z  =      2_556 Check
PLAT432_ALERT_2_G Short Inter X...Y Contact  C38C     ..C76C     .       2.64 Ang.
                                             -x,y,3/2-z  =      2_556 Check
PLAT432_ALERT_2_G Short Inter X...Y Contact  C39B     ..C59B     .       1.27 Ang.
                                            1-x,y,3/2-z  =      2_656 Check
PLAT432_ALERT_2_G Short Inter X...Y Contact  C39B     ..C58B     .       1.95 Ang.
                                            1-x,y,3/2-z  =      2_656 Check
```

```
PLAT432_ALERT_2_G Short Inter X...Y Contact  C39B     ..C55B      .       2.55 Ang.
                                               1-x,y,3/2-z  =      2_656 Check
PLAT432_ALERT_2_G Short Inter X...Y Contact  C39B     ..C80B      .       3.04 Ang.
                                               1-x,y,3/2-z  =      2_656 Check
PLAT432_ALERT_2_G Short Inter X...Y Contact  C39B     ..C57B      .       3.19 Ang.
                                               1-x,y,3/2-z  =      2_656 Check
PLAT432_ALERT_2_G Short Inter X...Y Contact  C39C     ..C75C      .       0.79 Ang.
                                                -x,y,3/2-z  =      2_556 Check
PLAT432_ALERT_2_G Short Inter X...Y Contact  C39C     ..C76C      .       1.95 Ang.
                                                -x,y,3/2-z  =      2_556 Check
PLAT432_ALERT_2_G Short Inter X...Y Contact  C39C     ..C78C      .       2.62 Ang.
                                                -x,y,3/2-z  =      2_556 Check
PLAT432_ALERT_2_G Short Inter X...Y Contact  C39C     ..C77C      .       2.62 Ang.
                                                -x,y,3/2-z  =      2_556 Check
PLAT432_ALERT_2_G Short Inter X...Y Contact  C39C     ..C73C      .       3.17 Ang.
                                                -x,y,3/2-z  =      2_556 Check
PLAT432_ALERT_2_G Short Inter X...Y Contact  C40B     ..C59B      .       1.27 Ang.
                                               1-x,y,3/2-z  =      2_656 Check
PLAT432_ALERT_2_G Short Inter X...Y Contact  C40B     ..C80B      .       1.68 Ang.
                                               1-x,y,3/2-z  =      2_656 Check
PLAT432_ALERT_2_G Short Inter X...Y Contact  C40B     ..C55B      .       1.70 Ang.
                                               1-x,y,3/2-z  =      2_656 Check
PLAT432_ALERT_2_G Short Inter X...Y Contact  C40B     ..C54B      .       1.90 Ang.
                                               1-x,y,3/2-z  =      2_656 Check
PLAT432_ALERT_2_G Short Inter X...Y Contact  C40B     ..C58B      .       2.59 Ang.
                                               1-x,y,3/2-z  =      2_656 Check
PLAT432_ALERT_2_G Short Inter X...Y Contact  C40B     ..C56B      .       2.99 Ang.
                                               1-x,y,3/2-z  =      2_656 Check
PLAT432_ALERT_2_G Short Inter X...Y Contact  C40C     ..C76C      .       0.49 Ang.
                                                -x,y,3/2-z  =      2_556 Check
PLAT432_ALERT_2_G Short Inter X...Y Contact  C40C     ..C75C      .       1.16 Ang.
                                                -x,y,3/2-z  =      2_556 Check
PLAT432_ALERT_2_G Short Inter X...Y Contact  C40C     ..C77C      .       1.41 Ang.
                                                -x,y,3/2-z  =      2_556 Check
PLAT432_ALERT_2_G Short Inter X...Y Contact  C40C     ..C73C      .       1.95 Ang.
                                                -x,y,3/2-z  =      2_556 Check
PLAT432_ALERT_2_G Short Inter X...Y Contact  C40C     ..C78C      .       2.23 Ang.
                                                -x,y,3/2-z  =      2_556 Check
PLAT432_ALERT_2_G Short Inter X...Y Contact  C40C     ..C52C      .       2.66 Ang.
                                                -x,y,3/2-z  =      2_556 Check
PLAT432_ALERT_2_G Short Inter X...Y Contact  C40C     ..C74C      .       2.92 Ang.
                                                -x,y,3/2-z  =      2_556 Check
PLAT432_ALERT_2_G Short Inter X...Y Contact  C40C     ..C72C      .       2.97 Ang.
                                                -x,y,3/2-z  =      2_556 Check
PLAT432_ALERT_2_G Short Inter X...Y Contact  C41B     ..C80B      .       0.89 Ang.
                                               1-x,y,3/2-z  =      2_656 Check
PLAT432_ALERT_2_G Short Inter X...Y Contact  C41B     ..C54B      .       2.11 Ang.
                                               1-x,y,3/2-z  =      2_656 Check
PLAT432_ALERT_2_G Short Inter X...Y Contact  C41B     ..C59B      .       2.65 Ang.
                                               1-x,y,3/2-z  =      2_656 Check
PLAT432_ALERT_2_G Short Inter X...Y Contact  C41B     ..C55B      .       2.73 Ang.
                                               1-x,y,3/2-z  =      2_656 Check
PLAT432_ALERT_2_G Short Inter X...Y Contact  C41C     ..C73C      .       0.66 Ang.
                                                -x,y,3/2-z  =      2_556 Check
PLAT432_ALERT_2_G Short Inter X...Y Contact  C41C     ..C76C      .       0.91 Ang.
                                                -x,y,3/2-z  =      2_556 Check
PLAT432_ALERT_2_G Short Inter X...Y Contact  C41C     ..C74C      .       1.67 Ang.
```

```
                                              -x,y,3/2-z  =      2_556 Check
PLAT432_ALERT_2_G Short Inter X...Y Contact  C41C     ..C75C     .       1.81 Ang.
                                              -x,y,3/2-z  =      2_556 Check
PLAT432_ALERT_2_G Short Inter X...Y Contact  C41C     ..C72C     .       2.02 Ang.
                                              -x,y,3/2-z  =      2_556 Check
PLAT432_ALERT_2_G Short Inter X...Y Contact  C41C     ..C77C     .       2.23 Ang.
                                              -x,y,3/2-z  =      2_556 Check
PLAT432_ALERT_2_G Short Inter X...Y Contact  C41C     ..C46C     .       2.83 Ang.
                                              -x,y,3/2-z  =      2_556 Check
PLAT432_ALERT_2_G Short Inter X...Y Contact  C41C     ..C51C     .       2.86 Ang.
                                              -x,y,3/2-z  =      2_556 Check
PLAT432_ALERT_2_G Short Inter X...Y Contact  C41C     ..C52C     .       2.91 Ang.
                                              -x,y,3/2-z  =      2_556 Check
PLAT432_ALERT_2_G Short Inter X...Y Contact  C41C     ..C47C     .       3.00 Ang.
                                              -x,y,3/2-z  =      2_556 Check
PLAT432_ALERT_2_G Short Inter X...Y Contact  C42B     ..C80B     .       2.23 Ang.
                                             1-x,y,3/2-z  =      2_656 Check
PLAT432_ALERT_2_G Short Inter X...Y Contact  C42C     ..C74C     .       0.80 Ang.
                                              -x,y,3/2-z  =      2_556 Check
PLAT432_ALERT_2_G Short Inter X...Y Contact  C42C     ..C73C     .       1.50 Ang.
                                              -x,y,3/2-z  =      2_556 Check
PLAT432_ALERT_2_G Short Inter X...Y Contact  C42C     ..C76C     .       2.11 Ang.
                                              -x,y,3/2-z  =      2_556 Check
PLAT432_ALERT_2_G Short Inter X...Y Contact  C42C     ..C75C     .       2.16 Ang.
                                              -x,y,3/2-z  =      2_556 Check
PLAT432_ALERT_2_G Short Inter X...Y Contact  C42C     ..C46C     .       2.25 Ang.
                                              -x,y,3/2-z  =      2_556 Check
PLAT432_ALERT_2_G Short Inter X...Y Contact  C42C     ..C72C     .       2.75 Ang.
                                              -x,y,3/2-z  =      2_556 Check
PLAT432_ALERT_2_G Short Inter X...Y Contact  C42C     ..C47C     .       3.10 Ang.
                                              -x,y,3/2-z  =      2_556 Check
PLAT432_ALERT_2_G Short Inter X...Y Contact  C42C     ..C45C     .       3.13 Ang.
                                              -x,y,3/2-z  =      2_556 Check
PLAT432_ALERT_2_G Short Inter X...Y Contact  C43B     ..C72B     .       3.05 Ang.
                                             1-x,y,3/2-z  =      2_656 Check
PLAT432_ALERT_2_G Short Inter X...Y Contact  C43B     ..C73B     .       3.10 Ang.
                                             1-x,y,3/2-z  =      2_656 Check
PLAT432_ALERT_2_G Short Inter X...Y Contact  C43C     ..C75C     .       1.81 Ang.
                                              -x,y,3/2-z  =      2_556 Check
PLAT432_ALERT_2_G Short Inter X...Y Contact  C43C     ..C74C     .       2.20 Ang.
                                              -x,y,3/2-z  =      2_556 Check
PLAT432_ALERT_2_G Short Inter X...Y Contact  C43C     ..C76C     .       2.62 Ang.
                                              -x,y,3/2-z  =      2_556 Check
PLAT432_ALERT_2_G Short Inter X...Y Contact  C43C     ..C73C     .       2.76 Ang.
                                              -x,y,3/2-z  =      2_556 Check
PLAT432_ALERT_2_G Short Inter X...Y Contact  C44B     ..C72B     .       1.77 Ang.
                                             1-x,y,3/2-z  =      2_656 Check
PLAT432_ALERT_2_G Short Inter X...Y Contact  C44B     ..C73B     .       1.80 Ang.
                                             1-x,y,3/2-z  =      2_656 Check
PLAT432_ALERT_2_G Short Inter X...Y Contact  C44B     ..C70B     .       3.08 Ang.
                                             1-x,y,3/2-z  =      2_656 Check
PLAT432_ALERT_2_G Short Inter X...Y Contact  C44B     ..C74B     .       3.17 Ang.
                                             1-x,y,3/2-z  =      2_656 Check
PLAT432_ALERT_2_G Short Inter X...Y Contact  C44C     ..C74C     .       2.88 Ang.
                                              -x,y,3/2-z  =      2_556 Check
PLAT432_ALERT_2_G Short Inter X...Y Contact  C44C     ..C75C     .       3.20 Ang.
                                              -x,y,3/2-z  =      2_556 Check
```

```
PLAT432_ALERT_2_G Short Inter X...Y Contact  C45B     ..C72B     .       0.87 Ang.
                                              1-x,y,3/2-z  =     2_656 Check
PLAT432_ALERT_2_G Short Inter X...Y Contact  C45B     ..C70B     .       1.91 Ang.
                                              1-x,y,3/2-z  =     2_656 Check
PLAT432_ALERT_2_G Short Inter X...Y Contact  C45B     ..C73B     .       2.02 Ang.
                                              1-x,y,3/2-z  =     2_656 Check
PLAT432_ALERT_2_G Short Inter X...Y Contact  C45B     ..C71B     .       2.20 Ang.
                                              1-x,y,3/2-z  =     2_656 Check
PLAT432_ALERT_2_G Short Inter X...Y Contact  C45B     ..C69B     .       3.14 Ang.
                                              1-x,y,3/2-z  =     2_656 Check
PLAT432_ALERT_2_G Short Inter X...Y Contact  C45B     ..C74B     .       3.20 Ang.
                                              1-x,y,3/2-z  =     2_656 Check
PLAT432_ALERT_2_G Short Inter X...Y Contact  C45C     ..C81C     .       2.38 Ang.
                                               -x,y,3/2-z  =     2_556 Check
PLAT432_ALERT_2_G Short Inter X...Y Contact  C45C     ..C45C     .       2.65 Ang.
                                               -x,y,3/2-z  =     2_556 Check
PLAT432_ALERT_2_G Short Inter X...Y Contact  C45C     ..C74C     .       2.98 Ang.
                                               -x,y,3/2-z  =     2_556 Check
PLAT432_ALERT_2_G Short Inter X...Y Contact  C45C     ..C58C     .       3.02 Ang.
                                               -x,y,3/2-z  =     2_556 Check
PLAT432_ALERT_2_G Short Inter X...Y Contact  C45C     ..C46C     .       3.18 Ang.
                                               -x,y,3/2-z  =     2_556 Check
PLAT432_ALERT_2_G Short Inter X...Y Contact  C46B     ..C72B     .       0.70 Ang.
                                              1-x,y,3/2-z  =     2_656 Check
PLAT432_ALERT_2_G Short Inter X...Y Contact  C46B     ..C70B     .       0.84 Ang.
                                              1-x,y,3/2-z  =     2_656 Check
PLAT432_ALERT_2_G Short Inter X...Y Contact  C46B     ..C73B     .       1.75 Ang.
                                              1-x,y,3/2-z  =     2_656 Check
PLAT432_ALERT_2_G Short Inter X...Y Contact  C46B     ..C69B     .       1.82 Ang.
                                              1-x,y,3/2-z  =     2_656 Check
PLAT432_ALERT_2_G Short Inter X...Y Contact  C46B     ..C71B     .       2.00 Ang.
                                              1-x,y,3/2-z  =     2_656 Check
PLAT432_ALERT_2_G Short Inter X...Y Contact  C46B     ..C74B     .       2.32 Ang.
                                              1-x,y,3/2-z  =     2_656 Check
PLAT432_ALERT_2_G Short Inter X...Y Contact  C46B     ..C75B     .       2.46 Ang.
                                              1-x,y,3/2-z  =     2_656 Check
PLAT432_ALERT_2_G Short Inter X...Y Contact  C46B     ..C68B     .       3.04 Ang.
                                              1-x,y,3/2-z  =     2_656 Check
PLAT432_ALERT_2_G Short Inter X...Y Contact  C46B     ..C66B     .       3.15 Ang.
                                              1-x,y,3/2-z  =     2_656 Check
PLAT432_ALERT_2_G Short Inter X...Y Contact  C46C     ..C58C     .       1.88 Ang.
                                               -x,y,3/2-z  =     2_556 Check
PLAT432_ALERT_2_G Short Inter X...Y Contact  C46C     ..C59C     .       2.72 Ang.
                                               -x,y,3/2-z  =     2_556 Check
PLAT432_ALERT_2_G Short Inter X...Y Contact  C46C     ..C63C     .       2.92 Ang.
                                               -x,y,3/2-z  =     2_556 Check
PLAT432_ALERT_2_G Short Inter X...Y Contact  C46C     ..C81C     .       2.98 Ang.
                                               -x,y,3/2-z  =     2_556 Check
PLAT432_ALERT_2_G Short Inter X...Y Contact  C47B     ..C74B     .       0.97 Ang.
                                              1-x,y,3/2-z  =     2_656 Check
PLAT432_ALERT_2_G Short Inter X...Y Contact  C47B     ..C73B     .       1.24 Ang.
                                              1-x,y,3/2-z  =     2_656 Check
PLAT432_ALERT_2_G Short Inter X...Y Contact  C47B     ..C75B     .       1.49 Ang.
                                              1-x,y,3/2-z  =     2_656 Check
PLAT432_ALERT_2_G Short Inter X...Y Contact  C47B     ..C72B     .       1.69 Ang.
                                              1-x,y,3/2-z  =     2_656 Check
PLAT432_ALERT_2_G Short Inter X...Y Contact  C47B     ..C69B     .       1.88 Ang.
```

```
                                               1-x,y,3/2-z  =      2_656 Check
PLAT432_ALERT_2_G Short Inter X...Y Contact  C47B     ..C70B     .       1.98 Ang.
                                               1-x,y,3/2-z  =      2_656 Check
PLAT432_ALERT_2_G Short Inter X...Y Contact  C47B     ..C76B     .       2.77 Ang.
                                               1-x,y,3/2-z  =      2_656 Check
PLAT432_ALERT_2_G Short Inter X...Y Contact  C47C     ..C58C     .       0.75 Ang.
                                                -x,y,3/2-z  =      2_556 Check
PLAT432_ALERT_2_G Short Inter X...Y Contact  C47C     ..C63C     .       1.55 Ang.
                                                -x,y,3/2-z  =      2_556 Check
PLAT432_ALERT_2_G Short Inter X...Y Contact  C47C     ..C59C     .       2.15 Ang.
                                                -x,y,3/2-z  =      2_556 Check
PLAT432_ALERT_2_G Short Inter X...Y Contact  C47C     ..C62C     .       2.85 Ang.
                                                -x,y,3/2-z  =      2_556 Check
PLAT432_ALERT_2_G Short Inter X...Y Contact  C48B     ..C73B     .       0.91 Ang.
                                               1-x,y,3/2-z  =      2_656 Check
PLAT432_ALERT_2_G Short Inter X...Y Contact  C48B     ..C74B     .       0.98 Ang.
                                               1-x,y,3/2-z  =      2_656 Check
PLAT432_ALERT_2_G Short Inter X...Y Contact  C48B     ..C72B     .       2.33 Ang.
                                               1-x,y,3/2-z  =      2_656 Check
PLAT432_ALERT_2_G Short Inter X...Y Contact  C48B     ..C75B     .       2.41 Ang.
                                               1-x,y,3/2-z  =      2_656 Check
PLAT432_ALERT_2_G Short Inter X...Y Contact  C48B     ..C70B     .       3.17 Ang.
                                               1-x,y,3/2-z  =      2_656 Check
PLAT432_ALERT_2_G Short Inter X...Y Contact  C48B     ..C69B     .       3.18 Ang.
                                               1-x,y,3/2-z  =      2_656 Check
PLAT432_ALERT_2_G Short Inter X...Y Contact  C48C     ..C63C     .       1.30 Ang.
                                                -x,y,3/2-z  =      2_556 Check
PLAT432_ALERT_2_G Short Inter X...Y Contact  C48C     ..C58C     .       1.86 Ang.
                                                -x,y,3/2-z  =      2_556 Check
PLAT432_ALERT_2_G Short Inter X...Y Contact  C48C     ..C62C     .       2.59 Ang.
                                                -x,y,3/2-z  =      2_556 Check
PLAT432_ALERT_2_G Short Inter X...Y Contact  C48C     ..C64C     .       2.99 Ang.
                                                -x,y,3/2-z  =      2_556 Check
PLAT432_ALERT_2_G Short Inter X...Y Contact  C49B     ..C74B     .       1.54 Ang.
                                               1-x,y,3/2-z  =      2_656 Check
PLAT432_ALERT_2_G Short Inter X...Y Contact  C49B     ..C73B     .       2.31 Ang.
                                               1-x,y,3/2-z  =      2_656 Check
PLAT432_ALERT_2_G Short Inter X...Y Contact  C49B     ..C75B     .       2.66 Ang.
                                               1-x,y,3/2-z  =      2_656 Check
PLAT432_ALERT_2_G Short Inter X...Y Contact  C49B     ..C76B     .       2.80 Ang.
                                               1-x,y,3/2-z  =      2_656 Check
PLAT432_ALERT_2_G Short Inter X...Y Contact  C49C     ..C63C     .       1.12 Ang.
                                                -x,y,3/2-z  =      2_556 Check
PLAT432_ALERT_2_G Short Inter X...Y Contact  C49C     ..C62C     .       1.55 Ang.
                                                -x,y,3/2-z  =      2_556 Check
PLAT432_ALERT_2_G Short Inter X...Y Contact  C49C     ..C64C     .       1.61 Ang.
                                                -x,y,3/2-z  =      2_556 Check
PLAT432_ALERT_2_G Short Inter X...Y Contact  C49C     ..C58C     .       2.47 Ang.
                                                -x,y,3/2-z  =      2_556 Check
PLAT432_ALERT_2_G Short Inter X...Y Contact  C49C     ..N36      .       2.88 Ang.
                                                     x,y,z  =      1_555 Check
PLAT432_ALERT_2_G Short Inter X...Y Contact  C49C     ..C61C     .       2.93 Ang.
                                                -x,y,3/2-z  =      2_556 Check
PLAT432_ALERT_2_G Short Inter X...Y Contact  C49C     ..C65C     .       2.99 Ang.
                                                -x,y,3/2-z  =      2_556 Check
PLAT432_ALERT_2_G Short Inter X...Y Contact  C49C     ..C127     .       3.17 Ang.
                                                     x,y,z  =      1_555 Check
```

```
PLAT432_ALERT_2_G Short Inter X...Y Contact  C50B     ..C76B     .       1.67 Ang.
                                               1-x,y,3/2-z  =     2_656 Check
PLAT432_ALERT_2_G Short Inter X...Y Contact  C50B     ..C74B     .       1.97 Ang.
                                               1-x,y,3/2-z  =     2_656 Check
PLAT432_ALERT_2_G Short Inter X...Y Contact  C50B     ..C75B     .       2.22 Ang.
                                               1-x,y,3/2-z  =     2_656 Check
PLAT432_ALERT_2_G Short Inter X...Y Contact  C50B     ..C77B     .       2.88 Ang.
                                               1-x,y,3/2-z  =     2_656 Check
PLAT432_ALERT_2_G Short Inter X...Y Contact  C50C     ..C62C     .       0.17 Ang.
                                                -x,y,3/2-z  =     2_556 Check
PLAT432_ALERT_2_G Short Inter X...Y Contact  C50C     ..C63C     .       1.27 Ang.
                                                -x,y,3/2-z  =     2_556 Check
PLAT432_ALERT_2_G Short Inter X...Y Contact  C50C     ..C64C     .       1.41 Ang.
                                                -x,y,3/2-z  =     2_556 Check
PLAT432_ALERT_2_G Short Inter X...Y Contact  C50C     ..C61C     .       1.58 Ang.
                                                -x,y,3/2-z  =     2_556 Check
PLAT432_ALERT_2_G Short Inter X...Y Contact  C50C     ..C58C     .       2.37 Ang.
                                                -x,y,3/2-z  =     2_556 Check
PLAT432_ALERT_2_G Short Inter X...Y Contact  C50C     ..C65C     .       2.54 Ang.
                                                -x,y,3/2-z  =     2_556 Check
PLAT432_ALERT_2_G Short Inter X...Y Contact  C50C     ..C60C     .       2.57 Ang.
                                                -x,y,3/2-z  =     2_556 Check
PLAT432_ALERT_2_G Short Inter X...Y Contact  C50C     ..C67C     .       2.62 Ang.
                                                -x,y,3/2-z  =     2_556 Check
PLAT432_ALERT_2_G Short Inter X...Y Contact  C50C     ..C59C     .       2.82 Ang.
                                                -x,y,3/2-z  =     2_556 Check
PLAT432_ALERT_2_G Short Inter X...Y Contact  C50C     ..C66C     .       2.94 Ang.
                                                -x,y,3/2-z  =     2_556 Check
PLAT432_ALERT_2_G Short Inter X...Y Contact  C51A     ..N30      .       2.86 Ang.
                                                     x,y,z  =     1_555 Check
PLAT432_ALERT_2_G Short Inter X...Y Contact  C51B     ..C76B     .       0.38 Ang.
                                               1-x,y,3/2-z  =     2_656 Check
PLAT432_ALERT_2_G Short Inter X...Y Contact  C51B     ..C75B     .       1.18 Ang.
                                               1-x,y,3/2-z  =     2_656 Check
PLAT432_ALERT_2_G Short Inter X...Y Contact  C51B     ..C77B     .       1.79 Ang.
                                               1-x,y,3/2-z  =     2_656 Check
PLAT432_ALERT_2_G Short Inter X...Y Contact  C51B     ..C74B     .       1.99 Ang.
                                               1-x,y,3/2-z  =     2_656 Check
PLAT432_ALERT_2_G Short Inter X...Y Contact  C51B     ..C69B     .       2.48 Ang.
                                               1-x,y,3/2-z  =     2_656 Check
PLAT432_ALERT_2_G Short Inter X...Y Contact  C51B     ..C78B     .       2.66 Ang.
                                               1-x,y,3/2-z  =     2_656 Check
PLAT432_ALERT_2_G Short Inter X...Y Contact  C51B     ..C68B     .       2.98 Ang.
                                               1-x,y,3/2-z  =     2_656 Check
PLAT432_ALERT_2_G Short Inter X...Y Contact  C51C     ..C61C     .       1.16 Ang.
                                                -x,y,3/2-z  =     2_556 Check
PLAT432_ALERT_2_G Short Inter X...Y Contact  C51C     ..C60C     .       1.24 Ang.
                                                -x,y,3/2-z  =     2_556 Check
PLAT432_ALERT_2_G Short Inter X...Y Contact  C51C     ..C62C     .       1.37 Ang.
                                                -x,y,3/2-z  =     2_556 Check
PLAT432_ALERT_2_G Short Inter X...Y Contact  C51C     ..C59C     .       1.53 Ang.
                                                -x,y,3/2-z  =     2_556 Check
PLAT432_ALERT_2_G Short Inter X...Y Contact  C51C     ..C63C     .       1.69 Ang.
                                                -x,y,3/2-z  =     2_556 Check
PLAT432_ALERT_2_G Short Inter X...Y Contact  C51C     ..C58C     .       1.79 Ang.
                                                -x,y,3/2-z  =     2_556 Check
PLAT432_ALERT_2_G Short Inter X...Y Contact  C51C     ..C70C     .       2.56 Ang.
```

```
                                                   -x,y,3/2-z  =      2_556 Check
PLAT432_ALERT_2_G Short Inter X...Y Contact  C51C     ..C67C     .        2.58 Ang.
                                                   -x,y,3/2-z  =      2_556 Check
PLAT432_ALERT_2_G Short Inter X...Y Contact  C51C     ..C64C     .        2.79 Ang.
                                                   -x,y,3/2-z  =      2_556 Check
PLAT432_ALERT_2_G Short Inter X...Y Contact  C52B     ..C75B     .        0.28 Ang.
                                                  1-x,y,3/2-z  =      2_656 Check
PLAT432_ALERT_2_G Short Inter X...Y Contact  C52B     ..C69B     .        1.27 Ang.
                                                  1-x,y,3/2-z  =      2_656 Check
PLAT432_ALERT_2_G Short Inter X...Y Contact  C52B     ..C74B     .        1.56 Ang.
                                                  1-x,y,3/2-z  =      2_656 Check
PLAT432_ALERT_2_G Short Inter X...Y Contact  C52B     ..C76B     .        1.65 Ang.
                                                  1-x,y,3/2-z  =      2_656 Check
PLAT432_ALERT_2_G Short Inter X...Y Contact  C52B     ..C68B     .        2.29 Ang.
                                                  1-x,y,3/2-z  =      2_656 Check
PLAT432_ALERT_2_G Short Inter X...Y Contact  C52B     ..C70B     .        2.35 Ang.
                                                  1-x,y,3/2-z  =      2_656 Check
PLAT432_ALERT_2_G Short Inter X...Y Contact  C52B     ..C73B     .        2.56 Ang.
                                                  1-x,y,3/2-z  =      2_656 Check
PLAT432_ALERT_2_G Short Inter X...Y Contact  C52B     ..C77B     .        2.60 Ang.
                                                  1-x,y,3/2-z  =      2_656 Check
PLAT432_ALERT_2_G Short Inter X...Y Contact  C52B     ..C78B     .        2.74 Ang.
                                                  1-x,y,3/2-z  =      2_656 Check
PLAT432_ALERT_2_G Short Inter X...Y Contact  C52B     ..C72B     .        2.79 Ang.
                                                  1-x,y,3/2-z  =      2_656 Check
PLAT432_ALERT_2_G Short Inter X...Y Contact  C52C     ..C60C     .        0.71 Ang.
                                                   -x,y,3/2-z  =      2_556 Check
PLAT432_ALERT_2_G Short Inter X...Y Contact  C52C     ..C61C     .        1.02 Ang.
                                                   -x,y,3/2-z  =      2_556 Check
PLAT432_ALERT_2_G Short Inter X...Y Contact  C52C     ..C70C     .        1.49 Ang.
                                                   -x,y,3/2-z  =      2_556 Check
PLAT432_ALERT_2_G Short Inter X...Y Contact  C52C     ..C67C     .        1.82 Ang.
                                                   -x,y,3/2-z  =      2_556 Check
PLAT432_ALERT_2_G Short Inter X...Y Contact  C52C     ..C59C     .        2.08 Ang.
                                                   -x,y,3/2-z  =      2_556 Check
PLAT432_ALERT_2_G Short Inter X...Y Contact  C52C     ..C69C     .        2.22 Ang.
                                                   -x,y,3/2-z  =      2_556 Check
PLAT432_ALERT_2_G Short Inter X...Y Contact  C52C     ..C68C     .        2.26 Ang.
                                                   -x,y,3/2-z  =      2_556 Check
PLAT432_ALERT_2_G Short Inter X...Y Contact  C52C     ..C62C     .        2.33 Ang.
                                                   -x,y,3/2-z  =      2_556 Check
PLAT432_ALERT_2_G Short Inter X...Y Contact  C52C     ..C58C     .        2.99 Ang.
                                                   -x,y,3/2-z  =      2_556 Check
PLAT432_ALERT_2_G Short Inter X...Y Contact  C52C     ..C63C     .        3.04 Ang.
                                                   -x,y,3/2-z  =      2_556 Check
PLAT432_ALERT_2_G Short Inter X...Y Contact  C52C     ..C66C     .        3.11 Ang.
                                                   -x,y,3/2-z  =      2_556 Check
PLAT432_ALERT_2_G Short Inter X...Y Contact  C53B     ..C69B     .        0.48 Ang.
                                                  1-x,y,3/2-z  =      2_656 Check
PLAT432_ALERT_2_G Short Inter X...Y Contact  C53B     ..C68B     .        1.03 Ang.
                                                  1-x,y,3/2-z  =      2_656 Check
PLAT432_ALERT_2_G Short Inter X...Y Contact  C53B     ..C75B     .        1.61 Ang.
                                                  1-x,y,3/2-z  =      2_656 Check
PLAT432_ALERT_2_G Short Inter X...Y Contact  C53B     ..C70B     .        1.83 Ang.
                                                  1-x,y,3/2-z  =      2_656 Check
PLAT432_ALERT_2_G Short Inter X...Y Contact  C53B     ..C78B     .        2.12 Ang.
                                                  1-x,y,3/2-z  =      2_656 Check
```

```
PLAT432_ALERT_2_G Short Inter X...Y Contact  C53B     ..C67B     .       2.21 Ang.
                                               1-x,y,3/2-z  =     2_656 Check
PLAT432_ALERT_2_G Short Inter X...Y Contact  C53B     ..C76B     .       2.50 Ang.
                                               1-x,y,3/2-z  =     2_656 Check
PLAT432_ALERT_2_G Short Inter X...Y Contact  C53B     ..C71B     .       2.69 Ang.
                                               1-x,y,3/2-z  =     2_656 Check
PLAT432_ALERT_2_G Short Inter X...Y Contact  C53B     ..C77B     .       2.72 Ang.
                                               1-x,y,3/2-z  =     2_656 Check
PLAT432_ALERT_2_G Short Inter X...Y Contact  C53B     ..C74B     .       2.80 Ang.
                                               1-x,y,3/2-z  =     2_656 Check
PLAT432_ALERT_2_G Short Inter X...Y Contact  C53B     ..C66B     .       2.85 Ang.
                                               1-x,y,3/2-z  =     2_656 Check
PLAT432_ALERT_2_G Short Inter X...Y Contact  C53B     ..C72B     .       2.92 Ang.
                                               1-x,y,3/2-z  =     2_656 Check
PLAT432_ALERT_2_G Short Inter X...Y Contact  C53C     ..C67C     .       0.61 Ang.
                                                -x,y,3/2-z  =     2_556 Check
PLAT432_ALERT_2_G Short Inter X...Y Contact  C53C     ..C68C     .       1.14 Ang.
                                                -x,y,3/2-z  =     2_556 Check
PLAT432_ALERT_2_G Short Inter X...Y Contact  C53C     ..C61C     .       1.40 Ang.
                                                -x,y,3/2-z  =     2_556 Check
PLAT432_ALERT_2_G Short Inter X...Y Contact  C53C     ..C69C     .       1.97 Ang.
                                                -x,y,3/2-z  =     2_556 Check
PLAT432_ALERT_2_G Short Inter X...Y Contact  C53C     ..C66C     .       1.98 Ang.
                                                -x,y,3/2-z  =     2_556 Check
PLAT432_ALERT_2_G Short Inter X...Y Contact  C53C     ..C60C     .       2.10 Ang.
                                                -x,y,3/2-z  =     2_556 Check
PLAT432_ALERT_2_G Short Inter X...Y Contact  C53C     ..C70C     .       2.32 Ang.
                                                -x,y,3/2-z  =     2_556 Check
PLAT432_ALERT_2_G Short Inter X...Y Contact  C53C     ..C62C     .       2.67 Ang.
                                                -x,y,3/2-z  =     2_556 Check
PLAT432_ALERT_2_G Short Inter X...Y Contact  C53C     ..C65C     .       3.06 Ang.
                                                -x,y,3/2-z  =     2_556 Check
PLAT432_ALERT_2_G Short Inter X...Y Contact  C54B     ..C68B     .       0.61 Ang.
                                               1-x,y,3/2-z  =     2_656 Check
PLAT432_ALERT_2_G Short Inter X...Y Contact  C54B     ..C78B     .       0.92 Ang.
                                               1-x,y,3/2-z  =     2_656 Check
PLAT432_ALERT_2_G Short Inter X...Y Contact  C54B     ..C67B     .       1.63 Ang.
                                               1-x,y,3/2-z  =     2_656 Check
PLAT432_ALERT_2_G Short Inter X...Y Contact  C54B     ..C69B     .       1.83 Ang.
                                               1-x,y,3/2-z  =     2_656 Check
PLAT432_ALERT_2_G Short Inter X...Y Contact  C54B     ..C77B     .       2.12 Ang.
                                               1-x,y,3/2-z  =     2_656 Check
PLAT432_ALERT_2_G Short Inter X...Y Contact  C54B     ..C75B     .       2.55 Ang.
                                               1-x,y,3/2-z  =     2_656 Check
PLAT432_ALERT_2_G Short Inter X...Y Contact  C54B     ..C76B     .       2.70 Ang.
                                               1-x,y,3/2-z  =     2_656 Check
PLAT432_ALERT_2_G Short Inter X...Y Contact  C54B     ..C66B     .       2.89 Ang.
                                               1-x,y,3/2-z  =     2_656 Check
PLAT432_ALERT_2_G Short Inter X...Y Contact  C54B     ..C70B     .       2.99 Ang.
                                               1-x,y,3/2-z  =     2_656 Check
PLAT432_ALERT_2_G Short Inter X...Y Contact  C54C     ..C66C     .       0.64 Ang.
                                                -x,y,3/2-z  =     2_556 Check
PLAT432_ALERT_2_G Short Inter X...Y Contact  C54C     ..C67C     .       0.88 Ang.
                                                -x,y,3/2-z  =     2_556 Check
PLAT432_ALERT_2_G Short Inter X...Y Contact  C54C     ..C65C     .       1.64 Ang.
                                                -x,y,3/2-z  =     2_556 Check
PLAT432_ALERT_2_G Short Inter X...Y Contact  C54C     ..C61C     .       1.84 Ang.
```

```
                                              -x,y,3/2-z  =      2_556 Check
PLAT432_ALERT_2_G Short Inter X...Y Contact  C54C     ..C68C     .       2.08 Ang.
                                              -x,y,3/2-z  =      2_556 Check
PLAT432_ALERT_2_G Short Inter X...Y Contact  C54C     ..C64C     .       2.28 Ang.
                                              -x,y,3/2-z  =      2_556 Check
PLAT432_ALERT_2_G Short Inter X...Y Contact  C54C     ..C62C     .       2.34 Ang.
                                              -x,y,3/2-z  =      2_556 Check
PLAT432_ALERT_2_G Short Inter X...Y Contact  C54C     ..C60C     .       3.13 Ang.
                                              -x,y,3/2-z  =      2_556 Check
PLAT432_ALERT_2_G Short Inter X...Y Contact  C55B     ..C78B     .       0.53 Ang.
                                             1-x,y,3/2-z  =      2_656 Check
PLAT432_ALERT_2_G Short Inter X...Y Contact  C55B     ..C77B     .       0.95 Ang.
                                             1-x,y,3/2-z  =      2_656 Check
PLAT432_ALERT_2_G Short Inter X...Y Contact  C55B     ..C68B     .       1.97 Ang.
                                             1-x,y,3/2-z  =      2_656 Check
PLAT432_ALERT_2_G Short Inter X...Y Contact  C55B     ..C76B     .       2.14 Ang.
                                             1-x,y,3/2-z  =      2_656 Check
PLAT432_ALERT_2_G Short Inter X...Y Contact  C55B     ..C75B     .       2.77 Ang.
                                             1-x,y,3/2-z  =      2_656 Check
PLAT432_ALERT_2_G Short Inter X...Y Contact  C55B     ..C69B     .       2.78 Ang.
                                             1-x,y,3/2-z  =      2_656 Check
PLAT432_ALERT_2_G Short Inter X...Y Contact  C55B     ..C67B     .       2.87 Ang.
                                             1-x,y,3/2-z  =      2_656 Check
PLAT432_ALERT_2_G Short Inter X...Y Contact  C55C     ..C64C     .       0.92 Ang.
                                              -x,y,3/2-z  =      2_556 Check
PLAT432_ALERT_2_G Short Inter X...Y Contact  C55C     ..C65C     .       1.21 Ang.
                                              -x,y,3/2-z  =      2_556 Check
PLAT432_ALERT_2_G Short Inter X...Y Contact  C55C     ..C62C     .       1.36 Ang.
                                              -x,y,3/2-z  =      2_556 Check
PLAT432_ALERT_2_G Short Inter X...Y Contact  C55C     ..C66C     .       1.71 Ang.
                                              -x,y,3/2-z  =      2_556 Check
PLAT432_ALERT_2_G Short Inter X...Y Contact  C55C     ..C61C     .       1.88 Ang.
                                              -x,y,3/2-z  =      2_556 Check
PLAT432_ALERT_2_G Short Inter X...Y Contact  C55C     ..C67C     .       1.99 Ang.
                                              -x,y,3/2-z  =      2_556 Check
PLAT432_ALERT_2_G Short Inter X...Y Contact  C55C     ..C63C     .       2.61 Ang.
                                              -x,y,3/2-z  =      2_556 Check
PLAT432_ALERT_2_G Short Inter X...Y Contact  C56B     ..C77B     .       0.52 Ang.
                                             1-x,y,3/2-z  =      2_656 Check
PLAT432_ALERT_2_G Short Inter X...Y Contact  C56B     ..C76B     .       1.04 Ang.
                                             1-x,y,3/2-z  =      2_656 Check
PLAT432_ALERT_2_G Short Inter X...Y Contact  C56B     ..C78B     .       1.83 Ang.
                                             1-x,y,3/2-z  =      2_656 Check
PLAT432_ALERT_2_G Short Inter X...Y Contact  C56B     ..C75B     .       2.27 Ang.
                                             1-x,y,3/2-z  =      2_656 Check
PLAT432_ALERT_2_G Short Inter X...Y Contact  C56B     ..C68B     .       2.85 Ang.
                                             1-x,y,3/2-z  =      2_656 Check
PLAT432_ALERT_2_G Short Inter X...Y Contact  C56B     ..C69B     .       3.03 Ang.
                                             1-x,y,3/2-z  =      2_656 Check
PLAT432_ALERT_2_G Short Inter X...Y Contact  C56C     ..C64C     .       0.92 Ang.
                                              -x,y,3/2-z  =      2_556 Check
PLAT432_ALERT_2_G Short Inter X...Y Contact  C56C     ..C65C     .       1.00 Ang.
                                              -x,y,3/2-z  =      2_556 Check
PLAT432_ALERT_2_G Short Inter X...Y Contact  C56C     ..C62C     .       2.32 Ang.
                                              -x,y,3/2-z  =      2_556 Check
PLAT432_ALERT_2_G Short Inter X...Y Contact  C56C     ..C66C     .       2.39 Ang.
                                              -x,y,3/2-z  =      2_556 Check
```

```
PLAT432_ALERT_2_G Short Inter X...Y Contact  C56C     ..C63C     .       3.08 Ang.
                                                -x,y,3/2-z  =      2_556 Check
PLAT432_ALERT_2_G Short Inter X...Y Contact  C56C     ..C61C     .       3.19 Ang.
                                                -x,y,3/2-z  =      2_556 Check
PLAT432_ALERT_2_G Short Inter X...Y Contact  C56C     ..C67C     .       3.20 Ang.
                                                -x,y,3/2-z  =      2_556 Check
PLAT432_ALERT_2_G Short Inter X...Y Contact  C57B     ..C77B     .       1.48 Ang.
                                               1-x,y,3/2-z  =      2_656 Check
PLAT432_ALERT_2_G Short Inter X...Y Contact  C57B     ..C76B     .       2.13 Ang.
                                               1-x,y,3/2-z  =      2_656 Check
PLAT432_ALERT_2_G Short Inter X...Y Contact  C57B     ..C78B     .       2.74 Ang.
                                               1-x,y,3/2-z  =      2_656 Check
PLAT432_ALERT_2_G Short Inter X...Y Contact  C57C     ..C64C     .       1.37 Ang.
                                                -x,y,3/2-z  =      2_556 Check
PLAT432_ALERT_2_G Short Inter X...Y Contact  C57C     ..C65C     .       2.37 Ang.
                                                -x,y,3/2-z  =      2_556 Check
PLAT432_ALERT_2_G Short Inter X...Y Contact  C57C     ..C62C     .       2.40 Ang.
                                                -x,y,3/2-z  =      2_556 Check
PLAT432_ALERT_2_G Short Inter X...Y Contact  C57C     ..C63C     .       2.48 Ang.
                                                -x,y,3/2-z  =      2_556 Check
PLAT432_ALERT_2_G Short Inter X...Y Contact  C58B     ..C77B     .       2.00 Ang.
                                               1-x,y,3/2-z  =      2_656 Check
PLAT432_ALERT_2_G Short Inter X...Y Contact  C58B     ..C78B     .       2.65 Ang.
                                               1-x,y,3/2-z  =      2_656 Check
PLAT432_ALERT_2_G Short Inter X...Y Contact  C58B     ..C76B     .       3.20 Ang.
                                               1-x,y,3/2-z  =      2_656 Check
PLAT432_ALERT_2_G Short Inter X...Y Contact  C58C     ..C72C     .       0.73 Ang.
                                                -x,y,3/2-z  =      2_556 Check
PLAT432_ALERT_2_G Short Inter X...Y Contact  C58C     ..C73C     .       1.89 Ang.
                                                -x,y,3/2-z  =      2_556 Check
PLAT432_ALERT_2_G Short Inter X...Y Contact  C58C     ..C74C     .       2.35 Ang.
                                                -x,y,3/2-z  =      2_556 Check
PLAT432_ALERT_2_G Short Inter X...Y Contact  C58C     ..C76C     .       3.07 Ang.
                                                -x,y,3/2-z  =      2_556 Check
PLAT432_ALERT_2_G Short Inter X...Y Contact  C58C     ..C71C     .       3.08 Ang.
                                                -x,y,3/2-z  =      2_556 Check
PLAT432_ALERT_2_G Short Inter X...Y Contact  C59B     ..C78B     .       1.63 Ang.
                                               1-x,y,3/2-z  =      2_656 Check
PLAT432_ALERT_2_G Short Inter X...Y Contact  C59B     ..C77B     .       1.81 Ang.
                                               1-x,y,3/2-z  =      2_656 Check
PLAT432_ALERT_2_G Short Inter X...Y Contact  C59B     ..C68B     .       3.01 Ang.
                                               1-x,y,3/2-z  =      2_656 Check
PLAT432_ALERT_2_G Short Inter X...Y Contact  C59C     ..C72C     .       0.77 Ang.
                                                -x,y,3/2-z  =      2_556 Check
PLAT432_ALERT_2_G Short Inter X...Y Contact  C59C     ..C73C     .       1.04 Ang.
                                                -x,y,3/2-z  =      2_556 Check
PLAT432_ALERT_2_G Short Inter X...Y Contact  C59C     ..C76C     .       1.80 Ang.
                                                -x,y,3/2-z  =      2_556 Check
PLAT432_ALERT_2_G Short Inter X...Y Contact  C59C     ..C77C     .       2.20 Ang.
                                                -x,y,3/2-z  =      2_556 Check
PLAT432_ALERT_2_G Short Inter X...Y Contact  C59C     ..C74C     .       2.29 Ang.
                                                -x,y,3/2-z  =      2_556 Check
PLAT432_ALERT_2_G Short Inter X...Y Contact  C59C     ..C75C     .       3.05 Ang.
                                                -x,y,3/2-z  =      2_556 Check
PLAT432_ALERT_2_G Short Inter X...Y Contact  C60B     ..C63B     .       2.56 Ang.
                                               1-x,y,3/2-z  =      2_656 Check
PLAT432_ALERT_2_G Short Inter X...Y Contact  C60B     ..C60B     .       3.08 Ang.
```

```
                                            1-x,y,3/2-z  =      2_656 Check
PLAT432_ALERT_2_G Short Inter X...Y Contact  C60C     ..C77C     .       1.31 Ang.
                                             -x,y,3/2-z  =      2_556 Check
PLAT432_ALERT_2_G Short Inter X...Y Contact  C60C     ..C72C     .       1.91 Ang.
                                             -x,y,3/2-z  =      2_556 Check
PLAT432_ALERT_2_G Short Inter X...Y Contact  C60C     ..C76C     .       2.02 Ang.
                                             -x,y,3/2-z  =      2_556 Check
PLAT432_ALERT_2_G Short Inter X...Y Contact  C60C     ..C73C     .       2.24 Ang.
                                             -x,y,3/2-z  =      2_556 Check
PLAT432_ALERT_2_G Short Inter X...Y Contact  C60C     ..C78C     .       2.49 Ang.
                                             -x,y,3/2-z  =      2_556 Check
PLAT432_ALERT_2_G Short Inter X...Y Contact  C60C     ..C79C     .       2.82 Ang.
                                             -x,y,3/2-z  =      2_556 Check
PLAT432_ALERT_2_G Short Inter X...Y Contact  C61B     ..C77B     .       2.67 Ang.
                                            1-x,y,3/2-z  =      2_656 Check
PLAT432_ALERT_2_G Short Inter X...Y Contact  C61B     ..C76B     .       2.69 Ang.
                                            1-x,y,3/2-z  =      2_656 Check
PLAT432_ALERT_2_G Short Inter X...Y Contact  C61C     ..C77C     .       2.42 Ang.
                                             -x,y,3/2-z  =      2_556 Check
PLAT432_ALERT_2_G Short Inter X...Y Contact  C61C     ..C72C     .       2.57 Ang.
                                             -x,y,3/2-z  =      2_556 Check
PLAT432_ALERT_2_G Short Inter X...Y Contact  C61C     ..C79C     .       2.68 Ang.
                                             -x,y,3/2-z  =      2_556 Check
PLAT432_ALERT_2_G Short Inter X...Y Contact  C61C     ..C78C     .       3.08 Ang.
                                             -x,y,3/2-z  =      2_556 Check
PLAT432_ALERT_2_G Short Inter X...Y Contact  C62B     ..C76B     .       2.51 Ang.
                                            1-x,y,3/2-z  =      2_656 Check
PLAT432_ALERT_2_G Short Inter X...Y Contact  C62C     ..C72C     .       2.48 Ang.
                                             -x,y,3/2-z  =      2_556 Check
PLAT432_ALERT_2_G Short Inter X...Y Contact  C63C     ..C72C     .       1.80 Ang.
                                             -x,y,3/2-z  =      2_556 Check
PLAT432_ALERT_2_G Short Inter X...Y Contact  C63C     ..C71C     .       2.69 Ang.
                                             -x,y,3/2-z  =      2_556 Check
PLAT432_ALERT_2_G Short Inter X...Y Contact  C63C     ..C73C     .       3.14 Ang.
                                             -x,y,3/2-z  =      2_556 Check
PLAT432_ALERT_2_G Short Inter X...Y Contact  C64B     ..C74B     .       2.86 Ang.
                                            1-x,y,3/2-z  =      2_656 Check
PLAT432_ALERT_2_G Short Inter X...Y Contact  C64C     ..C80C     .       3.10 Ang.
                                             -x,y,3/2-z  =      2_556 Check
PLAT432_ALERT_2_G Short Inter X...Y Contact  C65B     ..C73B     .       1.29 Ang.
                                            1-x,y,3/2-z  =      2_656 Check
PLAT432_ALERT_2_G Short Inter X...Y Contact  C65B     ..C72B     .       2.33 Ang.
                                            1-x,y,3/2-z  =      2_656 Check
PLAT432_ALERT_2_G Short Inter X...Y Contact  C65B     ..C74B     .       2.41 Ang.
                                            1-x,y,3/2-z  =      2_656 Check
PLAT432_ALERT_2_G Short Inter X...Y Contact  C65C     ..C80C     .       1.83 Ang.
                                             -x,y,3/2-z  =      2_556 Check
PLAT432_ALERT_2_G Short Inter X...Y Contact  C66B     ..C79B     .       2.06 Ang.
                                            1-x,y,3/2-z  =      2_656 Check
PLAT432_ALERT_2_G Short Inter X...Y Contact  C66B     ..C80B     .       2.13 Ang.
                                            1-x,y,3/2-z  =      2_656 Check
PLAT432_ALERT_2_G Short Inter X...Y Contact  C66C     ..C80C     .       0.84 Ang.
                                             -x,y,3/2-z  =      2_556 Check
PLAT432_ALERT_2_G Short Inter X...Y Contact  C66C     ..C79C     .       2.72 Ang.
                                             -x,y,3/2-z  =      2_556 Check
PLAT432_ALERT_2_G Short Inter X...Y Contact  C67B     ..C80B     .       0.73 Ang.
                                            1-x,y,3/2-z  =      2_656 Check
```

```
PLAT432_ALERT_2_G Short Inter X...Y Contact  C67B     ..C79B     .        2.27 Ang.
                                               1-x,y,3/2-z  =      2_656 Check
PLAT432_ALERT_2_G Short Inter X...Y Contact  C67C     ..C79C     .        1.79 Ang.
                                                -x,y,3/2-z  =      2_556 Check
PLAT432_ALERT_2_G Short Inter X...Y Contact  C67C     ..C80C     .        2.03 Ang.
                                                -x,y,3/2-z  =      2_556 Check
PLAT432_ALERT_2_G Short Inter X...Y Contact  C67C     ..C78C     .        2.82 Ang.
                                                -x,y,3/2-z  =      2_556 Check
PLAT432_ALERT_2_G Short Inter X...Y Contact  C67C     ..C77C     .        2.84 Ang.
                                                -x,y,3/2-z  =      2_556 Check
PLAT432_ALERT_2_G Short Inter X...Y Contact  C68B     ..C80B     .        1.47 Ang.
                                               1-x,y,3/2-z  =      2_656 Check
PLAT432_ALERT_2_G Short Inter X...Y Contact  C68B     ..C79B     .        1.91 Ang.
                                               1-x,y,3/2-z  =      2_656 Check
PLAT432_ALERT_2_G Short Inter X...Y Contact  C68C     ..C79C     .        0.44 Ang.
                                                -x,y,3/2-z  =      2_556 Check
PLAT432_ALERT_2_G Short Inter X...Y Contact  C68C     ..C78C     .        1.82 Ang.
                                                -x,y,3/2-z  =      2_556 Check
PLAT432_ALERT_2_G Short Inter X...Y Contact  C68C     ..C80C     .        2.46 Ang.
                                                -x,y,3/2-z  =      2_556 Check
PLAT432_ALERT_2_G Short Inter X...Y Contact  C68C     ..C77C     .        2.51 Ang.
                                                -x,y,3/2-z  =      2_556 Check
PLAT432_ALERT_2_G Short Inter X...Y Contact  C69B     ..C79B     .        1.16 Ang.
                                               1-x,y,3/2-z  =      2_656 Check
PLAT432_ALERT_2_G Short Inter X...Y Contact  C69B     ..C80B     .        2.75 Ang.
                                               1-x,y,3/2-z  =      2_656 Check
PLAT432_ALERT_2_G Short Inter X...Y Contact  C69C     ..C78C     .        0.43 Ang.
                                                -x,y,3/2-z  =      2_556 Check
PLAT432_ALERT_2_G Short Inter X...Y Contact  C69C     ..C79C     .        0.99 Ang.
                                                -x,y,3/2-z  =      2_556 Check
PLAT432_ALERT_2_G Short Inter X...Y Contact  C69C     ..C77C     .        1.60 Ang.
                                                -x,y,3/2-z  =      2_556 Check
PLAT432_ALERT_2_G Short Inter X...Y Contact  C69C     ..C76C     .        2.83 Ang.
                                                -x,y,3/2-z  =      2_556 Check
PLAT432_ALERT_2_G Short Inter X...Y Contact  C70B     ..C79B     .        0.61 Ang.
                                               1-x,y,3/2-z  =      2_656 Check
PLAT432_ALERT_2_G Short Inter X...Y Contact  C70C     ..C77C     .        0.06 Ang.
                                                -x,y,3/2-z  =      2_556 Check
PLAT432_ALERT_2_G Short Inter X...Y Contact  C70C     ..C78C     .        1.34 Ang.
                                                -x,y,3/2-z  =      2_556 Check
PLAT432_ALERT_2_G Short Inter X...Y Contact  C70C     ..C76C     .        1.49 Ang.
                                                -x,y,3/2-z  =      2_556 Check
PLAT432_ALERT_2_G Short Inter X...Y Contact  C70C     ..C79C     .        2.27 Ang.
                                                -x,y,3/2-z  =      2_556 Check
PLAT432_ALERT_2_G Short Inter X...Y Contact  C70C     ..C75C     .        2.42 Ang.
                                                -x,y,3/2-z  =      2_556 Check
PLAT432_ALERT_2_G Short Inter X...Y Contact  C70C     ..C73C     .        2.58 Ang.
                                                -x,y,3/2-z  =      2_556 Check
PLAT432_ALERT_2_G Short Inter X...Y Contact  C70C     ..C72C     .        2.94 Ang.
                                                -x,y,3/2-z  =      2_556 Check
PLAT432_ALERT_2_G Short Inter X...Y Contact  C71B     ..C79B     .        1.37 Ang.
                                               1-x,y,3/2-z  =      2_656 Check
PLAT432_ALERT_2_G Short Inter X...Y Contact  C71B     ..C80B     .        3.16 Ang.
                                               1-x,y,3/2-z  =      2_656 Check
PLAT432_ALERT_2_G Short Inter X...Y Contact  C72B     ..C79B     .        2.00 Ang.
                                               1-x,y,3/2-z  =      2_656 Check
PLAT432_ALERT_2_G Short Inter X...Y Contact  C73B     ..C79B     .        3.01 Ang.
```

```
                                                  1-x,y,3/2-z  =      2_656 Check
PLAT432_ALERT_2_G Short Inter X...Y Contact  C74B     ..C79B     .       3.11 Ang.
                                                  1-x,y,3/2-z  =      2_656 Check
PLAT432_ALERT_2_G Short Inter X...Y Contact  C75B     ..C79B     .       2.52 Ang.
                                                  1-x,y,3/2-z  =      2_656 Check
PLAT432_ALERT_2_G Short Inter X...Y Contact  C78B     ..C80B     .       1.94 Ang.
                                                  1-x,y,3/2-z  =      2_656 Check
PLAT432_ALERT_2_G Short Inter X...Y Contact  C81B     ..C81B     .       0.39 Ang.
                                                  1-x,y,3/2-z  =      2_656 Check
PLAT432_ALERT_2_G Short Inter X...Y Contact  C81C     ..C81C     .       0.59 Ang.
                                                   -x,y,3/2-z  =      2_556 Check
PLAT720_ALERT_4_G Number of Unusual/Non-Standard Labels .........          3 Note
PLAT764_ALERT_4_G Overcomplete CIF Bond List Detected (Rep/Expd) .       1.65 Ratio
PLAT773_ALERT_2_G Check long C-C Bond in CIF: C1C      --C46C            1.96 Ang.
PLAT773_ALERT_2_G Check long C-C Bond in CIF: C2C      --C6C             1.78 Ang.
PLAT773_ALERT_2_G Check long C-C Bond in CIF: C3C      --C74C            1.79 Ang.
PLAT773_ALERT_2_G Check long C-C Bond in CIF: C3C      --C72C            2.02 Ang.
PLAT773_ALERT_2_G Check long C-C Bond in CIF: C4C      --C48C            1.83 Ang.
PLAT773_ALERT_2_G Check long C-C Bond in CIF: C4C      --C47C            1.84 Ang.
PLAT773_ALERT_2_G Check long C-C Bond in CIF: C5C      --C43C            1.95 Ang.
PLAT773_ALERT_2_G Check long C-C Bond in CIF: C7C      --C44C            1.83 Ang.
PLAT773_ALERT_2_G Check long C-C Bond in CIF: C7C      --C39C            1.83 Ang.
PLAT773_ALERT_2_G Check long C-C Bond in CIF: C8C      --C44C            1.83 Ang.
PLAT773_ALERT_2_G Check long C-C Bond in CIF: C8C      --C36C            1.93 Ang.
PLAT773_ALERT_2_G Check long C-C Bond in CIF: C12C     --C31C            1.93 Ang.
PLAT773_ALERT_2_G Check long C-C Bond in CIF: C12C     --C37C            1.96 Ang.
PLAT773_ALERT_2_G Check long C-C Bond in CIF: C13C     --C39C            1.76 Ang.
PLAT773_ALERT_2_G Check long C-C Bond in CIF: C14C     --C35C            1.81 Ang.
PLAT773_ALERT_2_G Check long C-C Bond in CIF: C14C     --C33C            2.04 Ang.
PLAT773_ALERT_2_G Check long C-C Bond in CIF: C15C     --C32C            1.82 Ang.
PLAT773_ALERT_2_G Check long C-C Bond in CIF: C15C     --C39C            1.88 Ang.
PLAT773_ALERT_2_G Check long C-C Bond in CIF: C15C     --C35C            2.00 Ang.
PLAT773_ALERT_2_G Check long C-C Bond in CIF: C16C     --C34C            1.73 Ang.
PLAT773_ALERT_2_G Check long C-C Bond in CIF: C17C     --C32C            1.88 Ang.
PLAT773_ALERT_2_G Check long C-C Bond in CIF: C17C     --C36C            1.99 Ang.
PLAT773_ALERT_2_G Check long C-C Bond in CIF: C19C     --C32C            1.90 Ang.
PLAT773_ALERT_2_G Check long C-C Bond in CIF: C19C     --C31C            1.99 Ang.
PLAT773_ALERT_2_G Check long C-C Bond in CIF: C20C     --C28C            1.74 Ang.
PLAT773_ALERT_2_G Check long C-C Bond in CIF: C20C     --C31C            1.90 Ang.
PLAT773_ALERT_2_G Check long C-C Bond in CIF: C20C     --C33C            1.91 Ang.
PLAT773_ALERT_2_G Check long C-C Bond in CIF: C20C     --C26C            1.94 Ang.
PLAT773_ALERT_2_G Check long C-C Bond in CIF: C21C     --C25C            1.91 Ang.
PLAT773_ALERT_2_G Check long C-C Bond in CIF: C21C     --C32C            1.95 Ang.
PLAT773_ALERT_2_G Check long C-C Bond in CIF: C22C     --C27C            1.85 Ang.
PLAT773_ALERT_2_G Check long C-C Bond in CIF: C23C     --C25C            1.77 Ang.
PLAT773_ALERT_2_G Check long C-C Bond in CIF: C25C     --C29C            1.75 Ang.
PLAT773_ALERT_2_G Check long C-C Bond in CIF: C25C     --C54C            1.93 Ang.
PLAT773_ALERT_2_G Check long C-C Bond in CIF: C26C     --C80C            1.97 Ang.
PLAT773_ALERT_2_G Check long C-C Bond in CIF: C29C     --C65C            1.73 Ang.
PLAT773_ALERT_2_G Check long C-C Bond in CIF: C29C     --C66C            1.87 Ang.
PLAT773_ALERT_2_G Check long C-C Bond in CIF: C33C     --C79C            2.04 Ang.
PLAT773_ALERT_2_G Check long C-C Bond in CIF: C34C     --C75C            2.03 Ang.
PLAT773_ALERT_2_G Check long C-C Bond in CIF: C39C     --C76C            1.95 Ang.
PLAT773_ALERT_2_G Check long C-C Bond in CIF: C40C     --C73C            1.95 Ang.
PLAT773_ALERT_2_G Check long C-C Bond in CIF: C41C     --C75C            1.81 Ang.
PLAT773_ALERT_2_G Check long C-C Bond in CIF: C41C     --C72C            2.02 Ang.
PLAT773_ALERT_2_G Check long C-C Bond in CIF: C43C     --C75C            1.81 Ang.
```

PLAT773_ALERT_2_G Check long C-C Bond in CIF: C46C --C58C 1.88 Ang.
PLAT773_ALERT_2_G Check long C-C Bond in CIF: C48C --C58C 1.86 Ang.
PLAT773_ALERT_2_G Check long C-C Bond in CIF: C51C --C58C 1.79 Ang.
PLAT773_ALERT_2_G Check long C-C Bond in CIF: C52C --C67C 1.82 Ang.
PLAT773_ALERT_2_G Check long C-C Bond in CIF: C53C --C69C 1.97 Ang.
PLAT773_ALERT_2_G Check long C-C Bond in CIF: C53C --C66C 1.98 Ang.
PLAT773_ALERT_2_G Check long C-C Bond in CIF: C54C --C61C 1.84 Ang.
PLAT773_ALERT_2_G Check long C-C Bond in CIF: C55C --C66C 1.71 Ang.
PLAT773_ALERT_2_G Check long C-C Bond in CIF: C55C --C61C 1.88 Ang.
PLAT773_ALERT_2_G Check long C-C Bond in CIF: C55C --C67C 1.99 Ang.
PLAT773_ALERT_2_G Check long C-C Bond in CIF: C58C --C73C 1.89 Ang.
PLAT773_ALERT_2_G Check long C-C Bond in CIF: C59C --C76C 1.80 Ang.
PLAT773_ALERT_2_G Check long C-C Bond in CIF: C60C --C72C 1.91 Ang.
PLAT773_ALERT_2_G Check long C-C Bond in CIF: C60C --C76C 2.02 Ang.
PLAT773_ALERT_2_G Check long C-C Bond in CIF: C63C --C72C 1.80 Ang.
PLAT773_ALERT_2_G Check long C-C Bond in CIF: C65C --C80C 1.83 Ang.
PLAT773_ALERT_2_G Check long C-C Bond in CIF: C67C --C79C 1.79 Ang.
PLAT773_ALERT_2_G Check long C-C Bond in CIF: C67C --C80C 2.03 Ang.
PLAT773_ALERT_2_G Check long C-C Bond in CIF: C68C --C78C 1.83 Ang.
PLAT773_ALERT_2_G Check long C-C Bond in CIF: C1B --C5B 1.91 Ang.
PLAT773_ALERT_2_G Check long C-C Bond in CIF: C2B --C5B 2.00 Ang.
PLAT773_ALERT_2_G Check long C-C Bond in CIF: C3B --C12B 1.90 Ang.
PLAT773_ALERT_2_G Check long C-C Bond in CIF: C3B --C11B 1.96 Ang.
PLAT773_ALERT_2_G Check long C-C Bond in CIF: C4B --C71B 1.82 Ang.
PLAT773_ALERT_2_G Check long C-C Bond in CIF: C6B --C70B 1.71 Ang.
PLAT773_ALERT_2_G Check long C-C Bond in CIF: C6B --C67B 1.80 Ang.
PLAT773_ALERT_2_G Check long C-C Bond in CIF: C7B --C41B 1.77 Ang.
PLAT773_ALERT_2_G Check long C-C Bond in CIF: C8B --C66B 1.77 Ang.
PLAT773_ALERT_2_G Check long C-C Bond in CIF: C8B --C41B 1.98 Ang.
PLAT773_ALERT_2_G Check long C-C Bond in CIF: C11B --C46B 1.94 Ang.
PLAT773_ALERT_2_G Check long C-C Bond in CIF: C12B --C45B 1.79 Ang.
PLAT773_ALERT_2_G Check long C-C Bond in CIF: C14B --C67B 2.01 Ang.
PLAT773_ALERT_2_G Check long C-C Bond in CIF: C15B --C41B 1.89 Ang.
PLAT773_ALERT_2_G Check long C-C Bond in CIF: C16B --C43B 1.73 Ang.
PLAT773_ALERT_2_G Check long C-C Bond in CIF: C20B --C34B 1.87 Ang.
PLAT773_ALERT_2_G Check long C-C Bond in CIF: C20B --C39B 1.95 Ang.
PLAT773_ALERT_2_G Check long C-C Bond in CIF: C22B --C36B 1.96 Ang.
PLAT773_ALERT_2_G Check long C-C Bond in CIF: C22B --C32B 2.03 Ang.
PLAT773_ALERT_2_G Check long C-C Bond in CIF: C23B --C36B 1.87 Ang.
PLAT773_ALERT_2_G Check long C-C Bond in CIF: C23B --C31B 1.94 Ang.
PLAT773_ALERT_2_G Check long C-C Bond in CIF: C25B --C62B 1.99 Ang.
PLAT773_ALERT_2_G Check long C-C Bond in CIF: C26B --C49B 1.87 Ang.
PLAT773_ALERT_2_G Check long C-C Bond in CIF: C26B --C61B 1.99 Ang.
PLAT773_ALERT_2_G Check long C-C Bond in CIF: C27B --C63B 1.76 Ang.
PLAT773_ALERT_2_G Check long C-C Bond in CIF: C27B --C50B 1.82 Ang.
PLAT773_ALERT_2_G Check long C-C Bond in CIF: C27B --C60B 1.93 Ang.
PLAT773_ALERT_2_G Check long C-C Bond in CIF: C27B --C57B 2.00 Ang.
PLAT773_ALERT_2_G Check long C-C Bond in CIF: C28B --C61B 1.96 Ang.
PLAT773_ALERT_2_G Check long C-C Bond in CIF: C28B --C60B 2.03 Ang.
PLAT773_ALERT_2_G Check long C-C Bond in CIF: C29B --C60B 1.72 Ang.
PLAT773_ALERT_2_G Check long C-C Bond in CIF: C31B --C61B 1.81 Ang.
PLAT773_ALERT_2_G Check long C-C Bond in CIF: C32B --C58B 1.85 Ang.
PLAT773_ALERT_2_G Check long C-C Bond in CIF: C33B --C55B 1.94 Ang.
PLAT773_ALERT_2_G Check long C-C Bond in CIF: C33B --C61B 1.95 Ang.
PLAT773_ALERT_2_G Check long C-C Bond in CIF: C35B --C58B 1.74 Ang.
PLAT773_ALERT_2_G Check long C-C Bond in CIF: C39B --C58B 1.95 Ang.
PLAT773_ALERT_2_G Check long C-C Bond in CIF: C40B --C54B 1.89 Ang.

```
PLAT773_ALERT_2_G Check long C-C Bond in CIF: C44B     --C72B           1.77 Ang.
PLAT773_ALERT_2_G Check long C-C Bond in CIF: C44B     --C73B           1.80 Ang.
PLAT773_ALERT_2_G Check long C-C Bond in CIF: C45B     --C70B           1.91 Ang.
PLAT773_ALERT_2_G Check long C-C Bond in CIF: C45B     --C73B           2.02 Ang.
PLAT773_ALERT_2_G Check long C-C Bond in CIF: C46B     --C73B           1.75 Ang.
PLAT773_ALERT_2_G Check long C-C Bond in CIF: C46B     --C69B           1.82 Ang.
PLAT773_ALERT_2_G Check long C-C Bond in CIF: C46B     --C71B           2.00 Ang.
PLAT773_ALERT_2_G Check long C-C Bond in CIF: C47B     --C69B           1.88 Ang.
PLAT773_ALERT_2_G Check long C-C Bond in CIF: C47B     --C70B           1.98 Ang.
PLAT773_ALERT_2_G Check long C-C Bond in CIF: C50B     --C74B           1.97 Ang.
PLAT773_ALERT_2_G Check long C-C Bond in CIF: C51B     --C77B           1.79 Ang.
PLAT773_ALERT_2_G Check long C-C Bond in CIF: C51B     --C74B           1.99 Ang.
PLAT773_ALERT_2_G Check long C-C Bond in CIF: C53B     --C70B           1.83 Ang.
PLAT773_ALERT_2_G Check long C-C Bond in CIF: C54B     --C69B           1.84 Ang.
PLAT773_ALERT_2_G Check long C-C Bond in CIF: C55B     --C68B           1.97 Ang.
PLAT773_ALERT_2_G Check long C-C Bond in CIF: C56B     --C78B           1.83 Ang.
PLAT773_ALERT_2_G Check long C-C Bond in CIF: C58B     --C77B           2.00 Ang.
PLAT773_ALERT_2_G Check long C-C Bond in CIF: C59B     --C77B           1.81 Ang.
PLAT773_ALERT_2_G Check long C-C Bond in CIF: C68B     --C79B           1.90 Ang.
PLAT773_ALERT_2_G Check long C-C Bond in CIF: C72B     --C79B           2.00 Ang.
PLAT773_ALERT_2_G Check long C-C Bond in CIF: C78B     --C80B           1.94 Ang.
PLAT779_ALERT_4_G Suspect or Irrelevant (Bond) Angle(s) in CIF ...       8.20 Deg.
              ND6A -C10A -ND1A    1_555   1_555   1_555 ......     #  442 Check
PLAT779_ALERT_4_G Suspect or Irrelevant (Bond) Angle(s) in CIF ...      11.60 Deg.
              ND6A -C11A -ND1A    1_555   1_555   1_555 ......     #  452 Check
PLAT779_ALERT_4_G Suspect or Irrelevant (Bond) Angle(s) in CIF ...      11.30 Deg.
              ND6A -C12A -ND1A    1_555   1_555   1_555 ......     #  462 Check
PLAT779_ALERT_4_G Suspect or Irrelevant (Bond) Angle(s) in CIF ...       8.00 Deg.
              ND6A -C13A -ND1A    1_555   1_555   1_555 ......     #  472 Check
PLAT779_ALERT_4_G Suspect or Irrelevant (Bond) Angle(s) in CIF ...      10.70 Deg.
              ND1A -C17A -ND6A    1_555   1_555   1_555 ......     #  494 Check
PLAT779_ALERT_4_G Suspect or Irrelevant (Bond) Angle(s) in CIF ...       7.90 Deg.
              ND1A -C18A -ND6A    1_555   1_555   1_555 ......     #  504 Check
PLAT779_ALERT_4_G Suspect or Irrelevant (Bond) Angle(s) in CIF ...       8.51 Deg.
              ND5A -C30A -ND2A    1_555   1_555   1_555 ......     #  556 Check
PLAT779_ALERT_4_G Suspect or Irrelevant (Bond) Angle(s) in CIF ...       9.01 Deg.
              ND5A -C31A -ND2A    1_555   1_555   1_555 ......     #  566 Check
PLAT779_ALERT_4_G Suspect or Irrelevant (Bond) Angle(s) in CIF ...      11.80 Deg.
              ND5A -C35A -ND2A    1_555   1_555   1_555 ......     #  585 Check
PLAT779_ALERT_4_G Suspect or Irrelevant (Bond) Angle(s) in CIF ...      12.25 Deg.
              ND2A -C36A -ND5A    1_555   1_555   1_555 ......     #  595 Check
PLAT779_ALERT_4_G Suspect or Irrelevant (Bond) Angle(s) in CIF ...       8.59 Deg.
              ND2A -C37A -ND5A    1_555   1_555   1_555 ......     #  605 Check
PLAT779_ALERT_4_G Suspect or Irrelevant (Bond) Angle(s) in CIF ...      12.74 Deg.
              ND2A -C50A -ND5A    1_555   1_555   1_555 ......     #  663 Check
PLAT779_ALERT_4_G Suspect or Irrelevant (Bond) Angle(s) in CIF ...       9.83 Deg.
              ND2A -C51A -ND5A    1_555   1_555   1_555 ......     #  673 Check
PLAT779_ALERT_4_G Suspect or Irrelevant (Bond) Angle(s) in CIF ...      10.85 Deg.
              ND2A -C52A -ND5A    1_555   1_555   1_555 ......     #  687 Check
PLAT779_ALERT_4_G Suspect or Irrelevant (Bond) Angle(s) in CIF ...      11.49 Deg.
              ND5A -C53A -ND2A    1_555   1_555   1_555 ......     #  698 Check
PLAT779_ALERT_4_G Suspect or Irrelevant (Bond) Angle(s) in CIF ...      12.42 Deg.
              ND5A -C54A -ND2A    1_555   1_555   1_555 ......     #  713 Check
PLAT779_ALERT_4_G Suspect or Irrelevant (Bond) Angle(s) in CIF ...      10.20 Deg.
              ND1A -C75A -ND6A    1_555   1_555   1_555 ......     #  834 Check
PLAT779_ALERT_4_G Suspect or Irrelevant (Bond) Angle(s) in CIF ...      11.20 Deg.
              ND1A -C76A -ND6A    1_555   1_555   1_555 ......     #  849 Check
```

```
PLAT779_ALERT_4_G Suspect or Irrelevant (Bond) Angle(s) in CIF ...       7.90 Deg.
              ND1A -C77A -ND6A    1_555   1_555   1_555 ......     #  859 Check
PLAT779_ALERT_4_G Suspect or Irrelevant (Bond) Angle(s) in CIF ...       9.10 Deg.
              ND6A -C79A -ND1A    1_555   1_555   1_555 ......     #  875 Check
PLAT779_ALERT_4_G Suspect or Irrelevant (Bond) Angle(s) in CIF ...      28.30 Deg.
              C5C  -C1C  -C45C    2_556   1_555   1_555 ......     # 1162 Check
PLAT779_ALERT_4_G Suspect or Irrelevant (Bond) Angle(s) in CIF ...      28.40 Deg.
              C45C -C1C  -C5C     2_556   1_555   1_555 ......     # 1173 Check
PLAT779_ALERT_4_G Suspect or Irrelevant (Bond) Angle(s) in CIF ...      21.20 Deg.
              C81C -C1C  -C81C    1_555   1_555   2_556 ......     # 1183 Check
PLAT779_ALERT_4_G Suspect or Irrelevant (Bond) Angle(s) in CIF ...      44.30 Deg.
              C45C -C1C  -C46C    2_556   1_555   2_556 ......     # 1186 Check
PLAT779_ALERT_4_G Suspect or Irrelevant (Bond) Angle(s) in CIF ...      28.40 Deg.
              C2C  -C1C  -C46C    1_555   1_555   2_556 ......     # 1189 Check
PLAT779_ALERT_4_G Suspect or Irrelevant (Bond) Angle(s) in CIF ...       8.40 Deg.
              C46C -C2C  -C3C     2_556   1_555   1_555 ......     # 1195 Check
PLAT779_ALERT_4_G Suspect or Irrelevant (Bond) Angle(s) in CIF ...      23.20 Deg.
              C74C -C2C  -C42C    2_556   1_555   1_555 ......     # 1196 Check
PLAT779_ALERT_4_G Suspect or Irrelevant (Bond) Angle(s) in CIF ...      33.50 Deg.
              C42C -C2C  -C6C     1_555   1_555   2_556 ......     # 1211 Check
PLAT779_ALERT_4_G Suspect or Irrelevant (Bond) Angle(s) in CIF ...      16.30 Deg.
              C46C -C3C  -C2C     2_556   1_555   1_555 ......     # 1217 Check
PLAT779_ALERT_4_G Suspect or Irrelevant (Bond) Angle(s) in CIF ...      26.30 Deg.
              C47C -C3C  -C58C    2_556   1_555   1_555 ......     # 1221 Check
PLAT779_ALERT_4_G Suspect or Irrelevant (Bond) Angle(s) in CIF ...      43.30 Deg.
              C4C  -C3C  -C45C    1_555   1_555   2_556 ......     # 1226 Check
PLAT779_ALERT_4_G Suspect or Irrelevant (Bond) Angle(s) in CIF ...      28.90 Deg.
              C2C  -C3C  -C74C    1_555   1_555   2_556 ......     # 1232 Check
PLAT779_ALERT_4_G Suspect or Irrelevant (Bond) Angle(s) in CIF ...      41.90 Deg.
              C47C -C3C  -C72C    2_556   1_555   2_556 ......     # 1236 Check
PLAT779_ALERT_4_G Suspect or Irrelevant (Bond) Angle(s) in CIF ...      16.50 Deg.
              C58C -C3C  -C72C    1_555   1_555   2_556 ......     # 1239 Check
PLAT779_ALERT_4_G Suspect or Irrelevant (Bond) Angle(s) in CIF ...      33.40 Deg.
              C45C -C4C  -C5C     2_556   1_555   1_555 ......     # 1244 Check
PLAT779_ALERT_4_G Suspect or Irrelevant (Bond) Angle(s) in CIF ...      10.90 Deg.
              C9C  -C4C  -C71C    1_555   1_555   2_556 ......     # 1256 Check
PLAT779_ALERT_4_G Suspect or Irrelevant (Bond) Angle(s) in CIF ...      18.50 Deg.
              C3C  -C4C  -C46C    1_555   1_555   2_556 ......     # 1260 Check
PLAT779_ALERT_4_G Suspect or Irrelevant (Bond) Angle(s) in CIF ...      44.90 Deg.
              C9C  -C4C  -C48C    1_555   1_555   2_556 ......     # 1267 Check
PLAT779_ALERT_4_G Suspect or Irrelevant (Bond) Angle(s) in CIF ...      31.90 Deg.
              C3C  -C4C  -C47C    1_555   1_555   2_556 ......     # 1273 Check
PLAT779_ALERT_4_G Suspect or Irrelevant (Bond) Angle(s) in CIF ...      20.50 Deg.
              C1C  -C5C  -C1C     2_556   1_555   1_555 ......     # 1292 Check
PLAT779_ALERT_4_G Suspect or Irrelevant (Bond) Angle(s) in CIF ...      26.80 Deg.
              C6C  -C5C  -C43C    1_555   1_555   2_556 ......     # 1296 Check
PLAT779_ALERT_4_G Suspect or Irrelevant (Bond) Angle(s) in CIF ...      12.60 Deg.
              C43C -C6C  -C7C     2_556   1_555   1_555 ......     # 1300 Check
PLAT779_ALERT_4_G Suspect or Irrelevant (Bond) Angle(s) in CIF ...      29.50 Deg.
              C42C -C6C  -C74C    2_556   1_555   1_555 ......     # 1306 Check
PLAT779_ALERT_4_G Suspect or Irrelevant (Bond) Angle(s) in CIF ...      43.30 Deg.
              C5C  -C6C  -C44C    1_555   1_555   2_556 ......     # 1312 Check
PLAT779_ALERT_4_G Suspect or Irrelevant (Bond) Angle(s) in CIF ...      29.40 Deg.
              C74C -C6C  -C2C     1_555   1_555   2_556 ......     # 1318 Check
PLAT779_ALERT_4_G Suspect or Irrelevant (Bond) Angle(s) in CIF ...      25.40 Deg.
              C43C -C7C  -C6C     2_556   1_555   1_555 ......     # 1321 Check
PLAT779_ALERT_4_G Suspect or Irrelevant (Bond) Angle(s) in CIF ...      41.60 Deg.
```

```
              C38C -C7C  -C8C     2_556   1_555   1_555 ......     # 1327 Check
PLAT779_ALERT_4_G Suspect or Irrelevant (Bond) Angle(s) in CIF ...      35.40 Deg.
              C6C  -C7C  -C42C    1_555   1_555   2_556 ......     # 1332 Check
PLAT779_ALERT_4_G Suspect or Irrelevant (Bond) Angle(s) in CIF ...      32.10 Deg.
              C43C -C7C  -C44C    2_556   1_555   2_556 ......     # 1335 Check
PLAT779_ALERT_4_G Suspect or Irrelevant (Bond) Angle(s) in CIF ...      42.50 Deg.
              C38C -C7C  -C39C    2_556   1_555   2_556 ......     # 1342 Check
PLAT779_ALERT_4_G Suspect or Irrelevant (Bond) Angle(s) in CIF ...      24.50 Deg.
              C75C -C7C  -C39C    1_555   1_555   2_556 ......     # 1344 Check
PLAT779_ALERT_4_G Suspect or Irrelevant (Bond) Angle(s) in CIF ...      11.10 Deg.
              C71C -C8C  -C9C     2_556   1_555   1_555 ......     # 1353 Check
PLAT779_ALERT_4_G Suspect or Irrelevant (Bond) Angle(s) in CIF ...      36.60 Deg.
              C38C -C8C  -C7C     2_556   1_555   1_555 ......     # 1359 Check
PLAT779_ALERT_4_G Suspect or Irrelevant (Bond) Angle(s) in CIF ...      17.60 Deg.
              C7C  -C8C  -C43C    1_555   1_555   2_556 ......     # 1368 Check
PLAT779_ALERT_4_G Suspect or Irrelevant (Bond) Angle(s) in CIF ...      22.90 Deg.
              C37C -C8C  -C36C    2_556   1_555   2_556 ......     # 1376 Check
PLAT779_ALERT_4_G Suspect or Irrelevant (Bond) Angle(s) in CIF ...      34.30 Deg.
              C13C -C8C  -C36C    1_555   1_555   2_556 ......     # 1380 Check
PLAT779_ALERT_4_G Suspect or Irrelevant (Bond) Angle(s) in CIF ...      37.50 Deg.
              C48C -C9C  -C10C    2_556   1_555   1_555 ......     # 1395 Check
PLAT779_ALERT_4_G Suspect or Irrelevant (Bond) Angle(s) in CIF ...      25.10 Deg.
              C8C  -C9C  -C37C    1_555   1_555   2_556 ......     # 1402 Check
PLAT779_ALERT_4_G Suspect or Irrelevant (Bond) Angle(s) in CIF ...      41.80 Deg.
              C4C  -C9C  -C44C    1_555   1_555   2_556 ......     # 1413 Check
PLAT779_ALERT_4_G Suspect or Irrelevant (Bond) Angle(s) in CIF ...       6.80 Deg.
              C9C  -C10C -C71C    1_555   1_555   2_556 ......     # 1434 Check
PLAT779_ALERT_4_G Suspect or Irrelevant (Bond) Angle(s) in CIF ...       1.40 Deg.
              C11C -C10C -C57C    1_555   1_555   2_556 ......     # 1438 Check
PLAT779_ALERT_4_G Suspect or Irrelevant (Bond) Angle(s) in CIF ...      10.59 Deg.
              ND2C -C10C -ND1C    2_556   1_555   1_555 ......     # 1456 Check
PLAT779_ALERT_4_G Suspect or Irrelevant (Bond) Angle(s) in CIF ...      38.40 Deg.
              C49C -C11C -C10C    2_556   1_555   1_555 ......     # 1459 Check
PLAT779_ALERT_4_G Suspect or Irrelevant (Bond) Angle(s) in CIF ...      24.00 Deg.
              C12C -C11C -C36C    1_555   1_555   2_556 ......     # 1470 Check
PLAT779_ALERT_4_G Suspect or Irrelevant (Bond) Angle(s) in CIF ...      37.00 Deg.
              C57C -C11C -C56C    2_556   1_555   2_556 ......     # 1472 Check
PLAT779_ALERT_4_G Suspect or Irrelevant (Bond) Angle(s) in CIF ...      34.80 Deg.
              C64C -C11C -C56C    1_555   1_555   2_556 ......     # 1476 Check
PLAT779_ALERT_4_G Suspect or Irrelevant (Bond) Angle(s) in CIF ...      15.65 Deg.
              ND2C -C11C -ND1C    2_556   1_555   1_555 ......     # 1492 Check
PLAT779_ALERT_4_G Suspect or Irrelevant (Bond) Angle(s) in CIF ...       9.40 Deg.
              C57C -C12C -C11C    2_556   1_555   1_555 ......     # 1498 Check
PLAT779_ALERT_4_G Suspect or Irrelevant (Bond) Angle(s) in CIF ...      44.60 Deg.
              C36C -C12C -C13C    2_556   1_555   1_555 ......     # 1503 Check
PLAT779_ALERT_4_G Suspect or Irrelevant (Bond) Angle(s) in CIF ...       3.70 Deg.
              C17C -C12C -C31C    1_555   1_555   2_556 ......     # 1512 Check
PLAT779_ALERT_4_G Suspect or Irrelevant (Bond) Angle(s) in CIF ...      13.20 Deg.
              C36C -C12C -C37C    2_556   1_555   2_556 ......     # 1514 Check
PLAT779_ALERT_4_G Suspect or Irrelevant (Bond) Angle(s) in CIF ...      35.80 Deg.
              C13C -C12C -C37C    1_555   1_555   2_556 ......     # 1519 Check
PLAT779_ALERT_4_G Suspect or Irrelevant (Bond) Angle(s) in CIF ...      15.66 Deg.
              ND2C -C12C -ND1C    2_556   1_555   1_555 ......     # 1537 Check
PLAT779_ALERT_4_G Suspect or Irrelevant (Bond) Angle(s) in CIF ...      24.90 Deg.
              C37C -C13C -C8C     2_556   1_555   1_555 ......     # 1542 Check
PLAT779_ALERT_4_G Suspect or Irrelevant (Bond) Angle(s) in CIF ...      22.50 Deg.
              C36C -C13C -C12C    2_556   1_555   1_555 ......     # 1544 Check
```

```
PLAT779_ALERT_4_G Suspect or Irrelevant (Bond) Angle(s) in CIF ...      39.40 Deg.
              C8C  -C13C -C38C    1_555   1_555   2_556 ......     # 1556 Check
PLAT779_ALERT_4_G Suspect or Irrelevant (Bond) Angle(s) in CIF ...      22.70 Deg.
              C14C -C13C -C34C    1_555   1_555   2_556 ......     # 1564 Check
PLAT779_ALERT_4_G Suspect or Irrelevant (Bond) Angle(s) in CIF ...      22.70 Deg.
              C14C -C13C -C39C    1_555   1_555   2_556 ......     # 1571 Check
PLAT779_ALERT_4_G Suspect or Irrelevant (Bond) Angle(s) in CIF ...      11.81 Deg.
              ND2C -C13C -ND1C    2_556   1_555   1_555 ......     # 1592 Check
PLAT779_ALERT_4_G Suspect or Irrelevant (Bond) Angle(s) in CIF ...      34.70 Deg.
              C34C -C14C -C15C    2_556   1_555   1_555 ......     # 1594 Check
PLAT779_ALERT_4_G Suspect or Irrelevant (Bond) Angle(s) in CIF ...      10.60 Deg.
              C39C -C14C -C75C    2_556   1_555   1_555 ......     # 1597 Check
PLAT779_ALERT_4_G Suspect or Irrelevant (Bond) Angle(s) in CIF ...      43.00 Deg.
              C34C -C14C -C35C    2_556   1_555   2_556 ......     # 1608 Check
PLAT779_ALERT_4_G Suspect or Irrelevant (Bond) Angle(s) in CIF ...      32.10 Deg.
              C34C -C14C -C33C    2_556   1_555   2_556 ......     # 1614 Check
PLAT779_ALERT_4_G Suspect or Irrelevant (Bond) Angle(s) in CIF ...       3.40 Deg.
              C15C -C14C -C33C    1_555   1_555   2_556 ......     # 1616 Check
PLAT779_ALERT_4_G Suspect or Irrelevant (Bond) Angle(s) in CIF ...      22.30 Deg.
              C34C -C15C -C14C    2_556   1_555   1_555 ......     # 1623 Check
PLAT779_ALERT_4_G Suspect or Irrelevant (Bond) Angle(s) in CIF ...      13.20 Deg.
              C78C -C15C -C69C    1_555   1_555   2_556 ......     # 1635 Check
PLAT779_ALERT_4_G Suspect or Irrelevant (Bond) Angle(s) in CIF ...      35.60 Deg.
              C33C -C15C -C32C    2_556   1_555   2_556 ......     # 1636 Check
PLAT779_ALERT_4_G Suspect or Irrelevant (Bond) Angle(s) in CIF ...      19.70 Deg.
              C16C -C15C -C32C    1_555   1_555   2_556 ......     # 1639 Check
PLAT779_ALERT_4_G Suspect or Irrelevant (Bond) Angle(s) in CIF ...      38.90 Deg.
              C34C -C15C -C39C    2_556   1_555   2_556 ......     # 1643 Check
PLAT779_ALERT_4_G Suspect or Irrelevant (Bond) Angle(s) in CIF ...      16.70 Deg.
              C14C -C15C -C39C    1_555   1_555   2_556 ......     # 1644 Check
PLAT779_ALERT_4_G Suspect or Irrelevant (Bond) Angle(s) in CIF ...      39.80 Deg.
              C34C -C15C -C35C    2_556   1_555   2_556 ......     # 1650 Check
PLAT779_ALERT_4_G Suspect or Irrelevant (Bond) Angle(s) in CIF ...      29.10 Deg.
              C33C -C16C -C15C    2_556   1_555   1_555 ......     # 1662 Check
PLAT779_ALERT_4_G Suspect or Irrelevant (Bond) Angle(s) in CIF ...      16.30 Deg.
              C32C -C16C -C20C    2_556   1_555   1_555 ......     # 1663 Check
PLAT779_ALERT_4_G Suspect or Irrelevant (Bond) Angle(s) in CIF ...      17.50 Deg.
              C31C -C16C -C17C    2_556   1_555   1_555 ......     # 1668 Check
PLAT779_ALERT_4_G Suspect or Irrelevant (Bond) Angle(s) in CIF ...      42.10 Deg.
              C17C -C16C -C35C    1_555   1_555   2_556 ......     # 1677 Check
PLAT779_ALERT_4_G Suspect or Irrelevant (Bond) Angle(s) in CIF ...      32.10 Deg.
              C15C -C16C -C34C    1_555   1_555   2_556 ......     # 1681 Check
PLAT779_ALERT_4_G Suspect or Irrelevant (Bond) Angle(s) in CIF ...      42.00 Deg.
              C31C -C17C -C16C    2_556   1_555   1_555 ......     # 1699 Check
PLAT779_ALERT_4_G Suspect or Irrelevant (Bond) Angle(s) in CIF ...      10.50 Deg.
              C30C -C17C -C18C    2_556   1_555   1_555 ......     # 1705 Check
PLAT779_ALERT_4_G Suspect or Irrelevant (Bond) Angle(s) in CIF ...      27.00 Deg.
              C31C -C17C -C32C    2_556   1_555   2_556 ......     # 1708 Check
PLAT779_ALERT_4_G Suspect or Irrelevant (Bond) Angle(s) in CIF ...      18.80 Deg.
              C16C -C17C -C32C    1_555   1_555   2_556 ......     # 1712 Check
PLAT779_ALERT_4_G Suspect or Irrelevant (Bond) Angle(s) in CIF ...      43.50 Deg.
              C35C -C17C -C36C    2_556   1_555   2_556 ......     # 1715 Check
PLAT779_ALERT_4_G Suspect or Irrelevant (Bond) Angle(s) in CIF ...       8.10 Deg.
              C12C -C17C -C36C    1_555   1_555   2_556 ......     # 1717 Check
PLAT779_ALERT_4_G Suspect or Irrelevant (Bond) Angle(s) in CIF ...      14.57 Deg.
              ND1C -C17C -ND2C    1_555   1_555   2_556 ......     # 1737 Check
PLAT779_ALERT_4_G Suspect or Irrelevant (Bond) Angle(s) in CIF ...      44.80 Deg.
```

```
             C29C -C18C -C19C    2_556   1_555   1_555 ......     # 1742 Check
PLAT779_ALERT_4_G Suspect or Irrelevant (Bond) Angle(s) in CIF ...      20.30 Deg.
             C31C -C18C -C17C    2_556   1_555   1_555 ......     # 1746 Check
PLAT779_ALERT_4_G Suspect or Irrelevant (Bond) Angle(s) in CIF ...      20.00 Deg.
             C30C -C18C -C56C    2_556   1_555   2_556 ......     # 1753 Check
PLAT779_ALERT_4_G Suspect or Irrelevant (Bond) Angle(s) in CIF ...      36.40 Deg.
             C65C -C18C -C56C    1_555   1_555   2_556 ......     # 1758 Check
PLAT779_ALERT_4_G Suspect or Irrelevant (Bond) Angle(s) in CIF ...      10.33 Deg.
             ND1C -C18C -ND2C    1_555   1_555   2_556 ......     # 1773 Check
PLAT779_ALERT_4_G Suspect or Irrelevant (Bond) Angle(s) in CIF ...      26.30 Deg.
             C20C -C19C -C27C    1_555   1_555   2_556 ......     # 1782 Check
PLAT779_ALERT_4_G Suspect or Irrelevant (Bond) Angle(s) in CIF ...      29.30 Deg.
             C28C -C19C -C24C    2_556   1_555   1_555 ......     # 1784 Check
PLAT779_ALERT_4_G Suspect or Irrelevant (Bond) Angle(s) in CIF ...       5.00 Deg.
             C18C -C19C -C30C    1_555   1_555   2_556 ......     # 1792 Check
PLAT779_ALERT_4_G Suspect or Irrelevant (Bond) Angle(s) in CIF ...      22.10 Deg.
             C20C -C19C -C32C    1_555   1_555   2_556 ......     # 1797 Check
PLAT779_ALERT_4_G Suspect or Irrelevant (Bond) Angle(s) in CIF ...      44.60 Deg.
             C18C -C19C -C31C    1_555   1_555   2_556 ......     # 1805 Check
PLAT779_ALERT_4_G Suspect or Irrelevant (Bond) Angle(s) in CIF ...      43.80 Deg.
             C30C -C19C -C31C    2_556   1_555   2_556 ......     # 1808 Check
PLAT779_ALERT_4_G Suspect or Irrelevant (Bond) Angle(s) in CIF ...      43.40 Deg.
             C32C -C19C -C31C    2_556   1_555   2_556 ......     # 1809 Check
PLAT779_ALERT_4_G Suspect or Irrelevant (Bond) Angle(s) in CIF ...      43.90 Deg.
             C27C -C20C -C21C    2_556   1_555   1_555 ......     # 1822 Check
PLAT779_ALERT_4_G Suspect or Irrelevant (Bond) Angle(s) in CIF ...      14.00 Deg.
             C32C -C20C -C16C    2_556   1_555   1_555 ......     # 1826 Check
PLAT779_ALERT_4_G Suspect or Irrelevant (Bond) Angle(s) in CIF ...      31.00 Deg.
             C19C -C20C -C28C    1_555   1_555   2_556 ......     # 1831 Check
PLAT779_ALERT_4_G Suspect or Irrelevant (Bond) Angle(s) in CIF ...      44.40 Deg.
             C32C -C20C -C31C    2_556   1_555   2_556 ......     # 1835 Check
PLAT779_ALERT_4_G Suspect or Irrelevant (Bond) Angle(s) in CIF ...      36.10 Deg.
             C16C -C20C -C31C    1_555   1_555   2_556 ......     # 1838 Check
PLAT779_ALERT_4_G Suspect or Irrelevant (Bond) Angle(s) in CIF ...      33.70 Deg.
             C32C -C20C -C33C    2_556   1_555   2_556 ......     # 1841 Check
PLAT779_ALERT_4_G Suspect or Irrelevant (Bond) Angle(s) in CIF ...      36.90 Deg.
             C16C -C20C -C33C    1_555   1_555   2_556 ......     # 1844 Check
PLAT779_ALERT_4_G Suspect or Irrelevant (Bond) Angle(s) in CIF ...      36.30 Deg.
             C27C -C20C -C26C    2_556   1_555   2_556 ......     # 1847 Check
PLAT779_ALERT_4_G Suspect or Irrelevant (Bond) Angle(s) in CIF ...       7.90 Deg.
             C21C -C20C -C26C    1_555   1_555   2_556 ......     # 1850 Check
PLAT779_ALERT_4_G Suspect or Irrelevant (Bond) Angle(s) in CIF ...      33.30 Deg.
             C26C -C21C -C22C    2_556   1_555   1_555 ......     # 1856 Check
PLAT779_ALERT_4_G Suspect or Irrelevant (Bond) Angle(s) in CIF ...      25.10 Deg.
             C27C -C21C -C20C    2_556   1_555   1_555 ......     # 1859 Check
PLAT779_ALERT_4_G Suspect or Irrelevant (Bond) Angle(s) in CIF ...      17.80 Deg.
             C68C -C21C -C79C    2_556   1_555   1_555 ......     # 1869 Check
PLAT779_ALERT_4_G Suspect or Irrelevant (Bond) Angle(s) in CIF ...      20.80 Deg.
             C26C -C21C -C25C    2_556   1_555   2_556 ......     # 1870 Check
PLAT779_ALERT_4_G Suspect or Irrelevant (Bond) Angle(s) in CIF ...      18.30 Deg.
             C22C -C21C -C25C    1_555   1_555   2_556 ......     # 1872 Check
PLAT779_ALERT_4_G Suspect or Irrelevant (Bond) Angle(s) in CIF ...      21.20 Deg.
             C20C -C21C -C32C    1_555   1_555   2_556 ......     # 1879 Check
PLAT779_ALERT_4_G Suspect or Irrelevant (Bond) Angle(s) in CIF ...       5.40 Deg.
             C25C -C22C -C80C    2_556   1_555   1_555 ......     # 1894 Check
PLAT779_ALERT_4_G Suspect or Irrelevant (Bond) Angle(s) in CIF ...      17.50 Deg.
             C26C -C22C -C21C    2_556   1_555   1_555 ......     # 1898 Check
```

```
PLAT779_ALERT_4_G Suspect or Irrelevant (Bond) Angle(s) in CIF ...      37.00 Deg.
               C23C -C22C -C24C    1_555   1_555   2_556 ......     # 1903 Check
PLAT779_ALERT_4_G Suspect or Irrelevant (Bond) Angle(s) in CIF ...      35.00 Deg.
               C21C -C22C -C27C    1_555   1_555   2_556 ......     # 1910 Check
PLAT779_ALERT_4_G Suspect or Irrelevant (Bond) Angle(s) in CIF ...      31.70 Deg.
               C24C -C23C -C28C    2_556   1_555   1_555 ......     # 1916 Check
PLAT779_ALERT_4_G Suspect or Irrelevant (Bond) Angle(s) in CIF ...      31.20 Deg.
               C23C -C23C -C24C    2_556   1_555   1_555 ......     # 1918 Check
PLAT779_ALERT_4_G Suspect or Irrelevant (Bond) Angle(s) in CIF ...      29.50 Deg.
               C24C -C23C -C28C    1_555   1_555   2_556 ......     # 1926 Check
PLAT779_ALERT_4_G Suspect or Irrelevant (Bond) Angle(s) in CIF ...      22.40 Deg.
               C22C -C23C -C25C    1_555   1_555   2_556 ......     # 1929 Check
PLAT779_ALERT_4_G Suspect or Irrelevant (Bond) Angle(s) in CIF ...      23.60 Deg.
               C23C -C24C -C23C    2_556   1_555   1_555 ......     # 1940 Check
PLAT779_ALERT_4_G Suspect or Irrelevant (Bond) Angle(s) in CIF ...      33.00 Deg.
               C28C -C24C -C19C    2_556   1_555   1_555 ......     # 1943 Check
PLAT779_ALERT_4_G Suspect or Irrelevant (Bond) Angle(s) in CIF ...      42.80 Deg.
               C29C -C24C -C19C    2_556   1_555   1_555 ......     # 1945 Check
PLAT779_ALERT_4_G Suspect or Irrelevant (Bond) Angle(s) in CIF ...      28.40 Deg.
               C25C -C24C -C22C    1_555   1_555   2_556 ......     # 1951 Check
PLAT779_ALERT_4_G Suspect or Irrelevant (Bond) Angle(s) in CIF ...      25.00 Deg.
               C25C -C24C -C80C    1_555   1_555   2_556 ......     # 1957 Check
PLAT779_ALERT_4_G Suspect or Irrelevant (Bond) Angle(s) in CIF ...      43.40 Deg.
               C22C -C25C -C26C    2_556   1_555   1_555 ......     # 1962 Check
PLAT779_ALERT_4_G Suspect or Irrelevant (Bond) Angle(s) in CIF ...      25.00 Deg.
               C80C -C25C -C66C    2_556   1_555   1_555 ......     # 1968 Check
PLAT779_ALERT_4_G Suspect or Irrelevant (Bond) Angle(s) in CIF ...      31.00 Deg.
               C24C -C25C -C23C    1_555   1_555   2_556 ......     # 1979 Check
PLAT779_ALERT_4_G Suspect or Irrelevant (Bond) Angle(s) in CIF ...      39.20 Deg.
               C22C -C25C -C21C    2_556   1_555   2_556 ......     # 1982 Check
PLAT779_ALERT_4_G Suspect or Irrelevant (Bond) Angle(s) in CIF ...       8.00 Deg.
               C26C -C25C -C21C    1_555   1_555   2_556 ......     # 1984 Check
PLAT779_ALERT_4_G Suspect or Irrelevant (Bond) Angle(s) in CIF ...      39.00 Deg.
               C80C -C25C -C54C    2_556   1_555   2_556 ......     # 1990 Check
PLAT779_ALERT_4_G Suspect or Irrelevant (Bond) Angle(s) in CIF ...      14.30 Deg.
               C66C -C25C -C54C    1_555   1_555   2_556 ......     # 1993 Check
PLAT779_ALERT_4_G Suspect or Irrelevant (Bond) Angle(s) in CIF ...      28.80 Deg.
               C22C -C26C -C25C    2_556   1_555   1_555 ......     # 2001 Check
PLAT779_ALERT_4_G Suspect or Irrelevant (Bond) Angle(s) in CIF ...      35.60 Deg.
               C21C -C26C -C27C    2_556   1_555   1_555 ......     # 2003 Check
PLAT779_ALERT_4_G Suspect or Irrelevant (Bond) Angle(s) in CIF ...      16.00 Deg.
               C68C -C26C -C79C    1_555   1_555   2_556 ......     # 2009 Check
PLAT779_ALERT_4_G Suspect or Irrelevant (Bond) Angle(s) in CIF ...      20.70 Deg.
               C21C -C26C -C20C    2_556   1_555   2_556 ......     # 2012 Check
PLAT779_ALERT_4_G Suspect or Irrelevant (Bond) Angle(s) in CIF ...      15.20 Deg.
               C27C -C26C -C20C    1_555   1_555   2_556 ......     # 2016 Check
PLAT779_ALERT_4_G Suspect or Irrelevant (Bond) Angle(s) in CIF ...      43.80 Deg.
               C22C -C26C -C80C    2_556   1_555   2_556 ......     # 2019 Check
PLAT779_ALERT_4_G Suspect or Irrelevant (Bond) Angle(s) in CIF ...      15.30 Deg.
               C25C -C26C -C80C    1_555   1_555   2_556 ......     # 2021 Check
PLAT779_ALERT_4_G Suspect or Irrelevant (Bond) Angle(s) in CIF ...      10.00 Deg.
               C20C -C27C -C32C    2_556   1_555   1_555 ......     # 2036 Check
PLAT779_ALERT_4_G Suspect or Irrelevant (Bond) Angle(s) in CIF ...      36.40 Deg.
               C28C -C27C -C19C    1_555   1_555   2_556 ......     # 2041 Check
PLAT779_ALERT_4_G Suspect or Irrelevant (Bond) Angle(s) in CIF ...      17.70 Deg.
               C21C -C27C -C26C    2_556   1_555   1_555 ......     # 2044 Check
PLAT779_ALERT_4_G Suspect or Irrelevant (Bond) Angle(s) in CIF ...      33.10 Deg.
```

```
              C26C -C27C -C22C    1_555   1_555   2_556 ......     # 2053 Check
PLAT779_ALERT_4_G Suspect or Irrelevant (Bond) Angle(s) in CIF ...      36.70 Deg.
              C24C -C28C -C23C    2_556   1_555   1_555 ......     # 2060 Check
PLAT779_ALERT_4_G Suspect or Irrelevant (Bond) Angle(s) in CIF ...      25.60 Deg.
              C23C -C28C -C23C    1_555   1_555   2_556 ......     # 2068 Check
PLAT779_ALERT_4_G Suspect or Irrelevant (Bond) Angle(s) in CIF ...      20.80 Deg.
              C27C -C28C -C20C    1_555   1_555   2_556 ......     # 2071 Check
PLAT779_ALERT_4_G Suspect or Irrelevant (Bond) Angle(s) in CIF ...      39.30 Deg.
              C19C -C29C -C28C    2_556   1_555   1_555 ......     # 2081 Check
PLAT779_ALERT_4_G Suspect or Irrelevant (Bond) Angle(s) in CIF ...      33.40 Deg.
              C24C -C29C -C28C    2_556   1_555   1_555 ......     # 2084 Check
PLAT779_ALERT_4_G Suspect or Irrelevant (Bond) Angle(s) in CIF ...       8.60 Deg.
              C18C -C29C -C30C    2_556   1_555   1_555 ......     # 2086 Check
PLAT779_ALERT_4_G Suspect or Irrelevant (Bond) Angle(s) in CIF ...      22.80 Deg.
              C80C -C29C -C25C    1_555   1_555   2_556 ......     # 2098 Check
PLAT779_ALERT_4_G Suspect or Irrelevant (Bond) Angle(s) in CIF ...      24.40 Deg.
              C80C -C29C -C66C    1_555   1_555   2_556 ......     # 2105 Check
PLAT779_ALERT_4_G Suspect or Irrelevant (Bond) Angle(s) in CIF ...      21.00 Deg.
              C17C -C30C -C31C    2_556   1_555   1_555 ......     # 2124 Check
PLAT779_ALERT_4_G Suspect or Irrelevant (Bond) Angle(s) in CIF ...      42.30 Deg.
              C65C -C30C -C56C    2_556   1_555   1_555 ......     # 2128 Check
PLAT779_ALERT_4_G Suspect or Irrelevant (Bond) Angle(s) in CIF ...      44.00 Deg.
              C18C -C30C -C29C    2_556   1_555   1_555 ......     # 2130 Check
PLAT779_ALERT_4_G Suspect or Irrelevant (Bond) Angle(s) in CIF ...      27.00 Deg.
              C18C -C30C -C19C    2_556   1_555   2_556 ......     # 2135 Check
PLAT779_ALERT_4_G Suspect or Irrelevant (Bond) Angle(s) in CIF ...      38.10 Deg.
              C29C -C30C -C19C    1_555   1_555   2_556 ......     # 2140 Check
PLAT779_ALERT_4_G Suspect or Irrelevant (Bond) Angle(s) in CIF ...      11.60 Deg.
              ND1C -C30C -ND2C    2_556   1_555   1_555 ......     # 2155 Check
PLAT779_ALERT_4_G Suspect or Irrelevant (Bond) Angle(s) in CIF ...      11.20 Deg.
              C18C -C31C -C30C    2_556   1_555   1_555 ......     # 2161 Check
PLAT779_ALERT_4_G Suspect or Irrelevant (Bond) Angle(s) in CIF ...      27.80 Deg.
              C16C -C31C -C32C    2_556   1_555   1_555 ......     # 2167 Check
PLAT779_ALERT_4_G Suspect or Irrelevant (Bond) Angle(s) in CIF ...      22.90 Deg.
              C32C -C31C -C20C    1_555   1_555   2_556 ......     # 2176 Check
PLAT779_ALERT_4_G Suspect or Irrelevant (Bond) Angle(s) in CIF ...      10.60 Deg.
              C17C -C31C -C12C    2_556   1_555   2_556 ......     # 2177 Check
PLAT779_ALERT_4_G Suspect or Irrelevant (Bond) Angle(s) in CIF ...      35.30 Deg.
              C35C -C31C -C12C    1_555   1_555   2_556 ......     # 2181 Check
PLAT779_ALERT_4_G Suspect or Irrelevant (Bond) Angle(s) in CIF ...      42.20 Deg.
              C20C -C31C -C19C    2_556   1_555   2_556 ......     # 2190 Check
PLAT779_ALERT_4_G Suspect or Irrelevant (Bond) Angle(s) in CIF ...      12.64 Deg.
              ND1C -C31C -ND2C    2_556   1_555   1_555 ......     # 2210 Check
PLAT779_ALERT_4_G Suspect or Irrelevant (Bond) Angle(s) in CIF ...       8.10 Deg.
              C20C -C32C -C27C    2_556   1_555   1_555 ......     # 2215 Check
PLAT779_ALERT_4_G Suspect or Irrelevant (Bond) Angle(s) in CIF ...      43.30 Deg.
              C16C -C32C -C15C    2_556   1_555   2_556 ......     # 2221 Check
PLAT779_ALERT_4_G Suspect or Irrelevant (Bond) Angle(s) in CIF ...      17.50 Deg.
              C33C -C32C -C15C    1_555   1_555   2_556 ......     # 2223 Check
PLAT779_ALERT_4_G Suspect or Irrelevant (Bond) Angle(s) in CIF ...      42.80 Deg.
              C16C -C32C -C17C    2_556   1_555   2_556 ......     # 2226 Check
PLAT779_ALERT_4_G Suspect or Irrelevant (Bond) Angle(s) in CIF ...       9.00 Deg.
              C31C -C32C -C17C    1_555   1_555   2_556 ......     # 2230 Check
PLAT779_ALERT_4_G Suspect or Irrelevant (Bond) Angle(s) in CIF ...      41.30 Deg.
              C20C -C32C -C19C    2_556   1_555   2_556 ......     # 2233 Check
PLAT779_ALERT_4_G Suspect or Irrelevant (Bond) Angle(s) in CIF ...      40.50 Deg.
              C20C -C32C -C21C    2_556   1_555   2_556 ......     # 2240 Check
```

```
PLAT779_ALERT_4_G Suspect or Irrelevant (Bond) Angle(s) in CIF ...      32.50 Deg.
              C27C -C32C -C21C    1_555   1_555   2_556 ......     # 2242 Check
PLAT779_ALERT_4_G Suspect or Irrelevant (Bond) Angle(s) in CIF ...      31.30 Deg.
              C16C -C33C -C32C    2_556   1_555   1_555 ......     # 2249 Check
PLAT779_ALERT_4_G Suspect or Irrelevant (Bond) Angle(s) in CIF ...      17.70 Deg.
              C78C -C33C -C69C    2_556   1_555   1_555 ......     # 2256 Check
PLAT779_ALERT_4_G Suspect or Irrelevant (Bond) Angle(s) in CIF ...      17.80 Deg.
              C15C -C33C -C34C    2_556   1_555   1_555 ......     # 2257 Check
PLAT779_ALERT_4_G Suspect or Irrelevant (Bond) Angle(s) in CIF ...      19.60 Deg.
              C32C -C33C -C20C    1_555   1_555   2_556 ......     # 2264 Check
PLAT779_ALERT_4_G Suspect or Irrelevant (Bond) Angle(s) in CIF ...       6.80 Deg.
              C15C -C33C -C14C    2_556   1_555   2_556 ......     # 2268 Check
PLAT779_ALERT_4_G Suspect or Irrelevant (Bond) Angle(s) in CIF ...      12.20 Deg.
              C34C -C33C -C14C    1_555   1_555   2_556 ......     # 2273 Check
PLAT779_ALERT_4_G Suspect or Irrelevant (Bond) Angle(s) in CIF ...      43.80 Deg.
              C78C -C33C -C79C    2_556   1_555   2_556 ......     # 2278 Check
PLAT779_ALERT_4_G Suspect or Irrelevant (Bond) Angle(s) in CIF ...      26.10 Deg.
              C69C -C33C -C79C    1_555   1_555   2_556 ......     # 2279 Check
PLAT779_ALERT_4_G Suspect or Irrelevant (Bond) Angle(s) in CIF ...       9.20 Deg.
              C14C -C34C -C39C    2_556   1_555   1_555 ......     # 2284 Check
PLAT779_ALERT_4_G Suspect or Irrelevant (Bond) Angle(s) in CIF ...      13.20 Deg.
              C15C -C34C -C33C    2_556   1_555   1_555 ......     # 2290 Check
PLAT779_ALERT_4_G Suspect or Irrelevant (Bond) Angle(s) in CIF ...      40.60 Deg.
              C33C -C34C -C16C    1_555   1_555   2_556 ......     # 2302 Check
PLAT779_ALERT_4_G Suspect or Irrelevant (Bond) Angle(s) in CIF ...      19.30 Deg.
              C14C -C34C -C75C    2_556   1_555   2_556 ......     # 2304 Check
PLAT779_ALERT_4_G Suspect or Irrelevant (Bond) Angle(s) in CIF ...      10.40 Deg.
              C39C -C34C -C75C    1_555   1_555   2_556 ......     # 2306 Check
PLAT779_ALERT_4_G Suspect or Irrelevant (Bond) Angle(s) in CIF ...      24.40 Deg.
              C12C -C35C -C36C    2_556   1_555   1_555 ......     # 2315 Check
PLAT779_ALERT_4_G Suspect or Irrelevant (Bond) Angle(s) in CIF ...      18.80 Deg.
              C17C -C35C -C31C    2_556   1_555   1_555 ......     # 2317 Check
PLAT779_ALERT_4_G Suspect or Irrelevant (Bond) Angle(s) in CIF ...      42.30 Deg.
              C31C -C35C -C16C    1_555   1_555   2_556 ......     # 2330 Check
PLAT779_ALERT_4_G Suspect or Irrelevant (Bond) Angle(s) in CIF ...      17.20 Deg.
              C34C -C35C -C14C    1_555   1_555   2_556 ......     # 2337 Check
PLAT779_ALERT_4_G Suspect or Irrelevant (Bond) Angle(s) in CIF ...      24.50 Deg.
              C34C -C35C -C15C    1_555   1_555   2_556 ......     # 2344 Check
PLAT779_ALERT_4_G Suspect or Irrelevant (Bond) Angle(s) in CIF ...      43.80 Deg.
              C16C -C35C -C15C    2_556   1_555   2_556 ......     # 2345 Check
PLAT779_ALERT_4_G Suspect or Irrelevant (Bond) Angle(s) in CIF ...      41.30 Deg.
              C14C -C35C -C15C    2_556   1_555   2_556 ......     # 2346 Check
PLAT779_ALERT_4_G Suspect or Irrelevant (Bond) Angle(s) in CIF ...      14.14 Deg.
              ND2C -C35C -ND1C    1_555   1_555   2_556 ......     # 2365 Check
PLAT779_ALERT_4_G Suspect or Irrelevant (Bond) Angle(s) in CIF ...       9.30 Deg.
              C11C -C36C -C57C    2_556   1_555   1_555 ......     # 2380 Check
PLAT779_ALERT_4_G Suspect or Irrelevant (Bond) Angle(s) in CIF ...      10.10 Deg.
              C37C -C36C -C8C     1_555   1_555   2_556 ......     # 2383 Check
PLAT779_ALERT_4_G Suspect or Irrelevant (Bond) Angle(s) in CIF ...      19.60 Deg.
              C12C -C36C -C17C    2_556   1_555   2_556 ......     # 2387 Check
PLAT779_ALERT_4_G Suspect or Irrelevant (Bond) Angle(s) in CIF ...      33.40 Deg.
              C35C -C36C -C17C    1_555   1_555   2_556 ......     # 2390 Check
PLAT779_ALERT_4_G Suspect or Irrelevant (Bond) Angle(s) in CIF ...      13.74 Deg.
              ND2C -C36C -ND1C    1_555   1_555   2_556 ......     # 2410 Check
PLAT779_ALERT_4_G Suspect or Irrelevant (Bond) Angle(s) in CIF ...      35.30 Deg.
              C8C  -C37C -C38C    2_556   1_555   1_555 ......     # 2417 Check
PLAT779_ALERT_4_G Suspect or Irrelevant (Bond) Angle(s) in CIF ...      11.00 Deg.
```

```
             C71C -C37C -C9C     1_555   1_555   2_556 ......     # 2424 Check
PLAT779_ALERT_4_G Suspect or Irrelevant (Bond) Angle(s) in CIF ...       5.70 Deg.
             C36C -C37C -C12C    1_555   1_555   2_556 ......     # 2428 Check
PLAT779_ALERT_4_G Suspect or Irrelevant (Bond) Angle(s) in CIF ...      16.40 Deg.
             C7C  -C38C -C43C    2_556   1_555   1_555 ......     # 2440 Check
PLAT779_ALERT_4_G Suspect or Irrelevant (Bond) Angle(s) in CIF ...      34.00 Deg.
             C39C -C38C -C75C    1_555   1_555   2_556 ......     # 2448 Check
PLAT779_ALERT_4_G Suspect or Irrelevant (Bond) Angle(s) in CIF ...      21.30 Deg.
             C8C  -C38C -C37C    2_556   1_555   1_555 ......     # 2450 Check
PLAT779_ALERT_4_G Suspect or Irrelevant (Bond) Angle(s) in CIF ...      22.70 Deg.
             C39C -C38C -C14C    1_555   1_555   2_556 ......     # 2463 Check
PLAT779_ALERT_4_G Suspect or Irrelevant (Bond) Angle(s) in CIF ...       8.10 Deg.
             C14C -C39C -C34C    2_556   1_555   1_555 ......     # 2468 Check
PLAT779_ALERT_4_G Suspect or Irrelevant (Bond) Angle(s) in CIF ...      41.70 Deg.
             C75C -C39C -C40C    2_556   1_555   1_555 ......     # 2474 Check
PLAT779_ALERT_4_G Suspect or Irrelevant (Bond) Angle(s) in CIF ...      27.10 Deg.
             C38C -C39C -C7C     1_555   1_555   2_556 ......     # 2485 Check
PLAT779_ALERT_4_G Suspect or Irrelevant (Bond) Angle(s) in CIF ...      34.00 Deg.
             C14C -C39C -C15C    2_556   1_555   2_556 ......     # 2488 Check
PLAT779_ALERT_4_G Suspect or Irrelevant (Bond) Angle(s) in CIF ...      26.50 Deg.
             C34C -C39C -C15C    1_555   1_555   2_556 ......     # 2490 Check
PLAT779_ALERT_4_G Suspect or Irrelevant (Bond) Angle(s) in CIF ...      33.40 Deg.
             C75C -C39C -C76C    2_556   1_555   2_556 ......     # 2496 Check
PLAT779_ALERT_4_G Suspect or Irrelevant (Bond) Angle(s) in CIF ...      11.90 Deg.
             C40C -C39C -C76C    1_555   1_555   2_556 ......     # 2499 Check
PLAT779_ALERT_4_G Suspect or Irrelevant (Bond) Angle(s) in CIF ...      19.30 Deg.
             C76C -C40C -C41C    2_556   1_555   1_555 ......     # 2504 Check
PLAT779_ALERT_4_G Suspect or Irrelevant (Bond) Angle(s) in CIF ...       2.60 Deg.
             C70C -C40C -C77C    1_555   1_555   2_556 ......     # 2512 Check
PLAT779_ALERT_4_G Suspect or Irrelevant (Bond) Angle(s) in CIF ...      26.90 Deg.
             C75C -C40C -C39C    2_556   1_555   1_555 ......     # 2514 Check
PLAT779_ALERT_4_G Suspect or Irrelevant (Bond) Angle(s) in CIF ...      10.30 Deg.
             C76C -C40C -C73C    2_556   1_555   2_556 ......     # 2518 Check
PLAT779_ALERT_4_G Suspect or Irrelevant (Bond) Angle(s) in CIF ...      10.40 Deg.
             C41C -C40C -C73C    1_555   1_555   2_556 ......     # 2520 Check
PLAT779_ALERT_4_G Suspect or Irrelevant (Bond) Angle(s) in CIF ...      10.17 Deg.
             ND4C -C40C -ND3C    1_555   1_555   2_556 ......     # 2538 Check
PLAT779_ALERT_4_G Suspect or Irrelevant (Bond) Angle(s) in CIF ...      10.30 Deg.
             C76C -C41C -C40C    2_556   1_555   1_555 ......     # 2541 Check
PLAT779_ALERT_4_G Suspect or Irrelevant (Bond) Angle(s) in CIF ...      41.40 Deg.
             C73C -C41C -C59C    2_556   1_555   1_555 ......     # 2545 Check
PLAT779_ALERT_4_G Suspect or Irrelevant (Bond) Angle(s) in CIF ...      28.60 Deg.
             C42C -C41C -C74C    1_555   1_555   2_556 ......     # 2552 Check
PLAT779_ALERT_4_G Suspect or Irrelevant (Bond) Angle(s) in CIF ...      40.00 Deg.
             C40C -C41C -C75C    1_555   1_555   2_556 ......     # 2556 Check
PLAT779_ALERT_4_G Suspect or Irrelevant (Bond) Angle(s) in CIF ...      25.50 Deg.
             C73C -C41C -C72C    2_556   1_555   2_556 ......     # 2560 Check
PLAT779_ALERT_4_G Suspect or Irrelevant (Bond) Angle(s) in CIF ...      17.20 Deg.
             C59C -C41C -C72C    1_555   1_555   2_556 ......     # 2564 Check
PLAT779_ALERT_4_G Suspect or Irrelevant (Bond) Angle(s) in CIF ...      38.00 Deg.
             C6C  -C42C -C43C    2_556   1_555   1_555 ......     # 2579 Check
PLAT779_ALERT_4_G Suspect or Irrelevant (Bond) Angle(s) in CIF ...      26.10 Deg.
             C41C -C42C -C73C    1_555   1_555   2_556 ......     # 2583 Check
PLAT779_ALERT_4_G Suspect or Irrelevant (Bond) Angle(s) in CIF ...      25.60 Deg.
             C74C -C42C -C2C     2_556   1_555   1_555 ......     # 2585 Check
PLAT779_ALERT_4_G Suspect or Irrelevant (Bond) Angle(s) in CIF ...      15.40 Deg.
             C43C -C42C -C7C     1_555   1_555   2_556 ......     # 2593 Check
```

```
PLAT779_ALERT_4_G Suspect or Irrelevant (Bond) Angle(s) in CIF ...      31.90 Deg.
              C7C  -C43C -C38C    2_556   1_555   1_555 ......     # 2604 Check
PLAT779_ALERT_4_G Suspect or Irrelevant (Bond) Angle(s) in CIF ...      40.40 Deg.
              C6C  -C43C -C42C    2_556   1_555   1_555 ......     # 2610 Check
PLAT779_ALERT_4_G Suspect or Irrelevant (Bond) Angle(s) in CIF ...      39.00 Deg.
              C38C -C43C -C8C     1_555   1_555   2_556 ......     # 2615 Check
PLAT779_ALERT_4_G Suspect or Irrelevant (Bond) Angle(s) in CIF ...      35.80 Deg.
              C7C  -C43C -C75C    2_556   1_555   2_556 ......     # 2618 Check
PLAT779_ALERT_4_G Suspect or Irrelevant (Bond) Angle(s) in CIF ...      43.20 Deg.
              C6C  -C43C -C5C     2_556   1_555   2_556 ......     # 2625 Check
PLAT779_ALERT_4_G Suspect or Irrelevant (Bond) Angle(s) in CIF ...      33.10 Deg.
              C44C -C43C -C5C     1_555   1_555   2_556 ......     # 2627 Check
PLAT779_ALERT_4_G Suspect or Irrelevant (Bond) Angle(s) in CIF ...      29.80 Deg.
              C5C  -C44C -C45C    2_556   1_555   1_555 ......     # 2638 Check
PLAT779_ALERT_4_G Suspect or Irrelevant (Bond) Angle(s) in CIF ...      36.90 Deg.
              C43C -C44C -C6C     1_555   1_555   2_556 ......     # 2644 Check
PLAT779_ALERT_4_G Suspect or Irrelevant (Bond) Angle(s) in CIF ...       9.80 Deg.
              C71C -C44C -C9C     1_555   1_555   2_556 ......     # 2648 Check
PLAT779_ALERT_4_G Suspect or Irrelevant (Bond) Angle(s) in CIF ...      10.10 Deg.
              C43C -C44C -C7C     1_555   1_555   2_556 ......     # 2655 Check
PLAT779_ALERT_4_G Suspect or Irrelevant (Bond) Angle(s) in CIF ...      20.30 Deg.
              C1C  -C45C -C1C     2_556   1_555   1_555 ......     # 2679 Check
PLAT779_ALERT_4_G Suspect or Irrelevant (Bond) Angle(s) in CIF ...      16.90 Deg.
              C46C -C45C -C3C     1_555   1_555   2_556 ......     # 2685 Check
PLAT779_ALERT_4_G Suspect or Irrelevant (Bond) Angle(s) in CIF ...      34.00 Deg.
              C46C -C45C -C2C     1_555   1_555   2_556 ......     # 2691 Check
PLAT779_ALERT_4_G Suspect or Irrelevant (Bond) Angle(s) in CIF ...      12.00 Deg.
              C3C  -C46C -C47C    2_556   1_555   1_555 ......     # 2698 Check
PLAT779_ALERT_4_G Suspect or Irrelevant (Bond) Angle(s) in CIF ...      34.20 Deg.
              C2C  -C46C -C74C    2_556   1_555   1_555 ......     # 2702 Check
PLAT779_ALERT_4_G Suspect or Irrelevant (Bond) Angle(s) in CIF ...      44.90 Deg.
              C45C -C46C -C4C     1_555   1_555   2_556 ......     # 2707 Check
PLAT779_ALERT_4_G Suspect or Irrelevant (Bond) Angle(s) in CIF ...      33.60 Deg.
              C3C  -C46C -C58C    2_556   1_555   2_556 ......     # 2710 Check
PLAT779_ALERT_4_G Suspect or Irrelevant (Bond) Angle(s) in CIF ...      21.70 Deg.
              C47C -C46C -C58C    1_555   1_555   2_556 ......     # 2713 Check
PLAT779_ALERT_4_G Suspect or Irrelevant (Bond) Angle(s) in CIF ...      41.70 Deg.
              C45C -C46C -C1C     1_555   1_555   2_556 ......     # 2718 Check
PLAT779_ALERT_4_G Suspect or Irrelevant (Bond) Angle(s) in CIF ...      11.30 Deg.
              C58C -C47C -C72C    2_556   1_555   1_555 ......     # 2726 Check
PLAT779_ALERT_4_G Suspect or Irrelevant (Bond) Angle(s) in CIF ...       6.00 Deg.
              C3C  -C47C -C46C    2_556   1_555   1_555 ......     # 2730 Check
PLAT779_ALERT_4_G Suspect or Irrelevant (Bond) Angle(s) in CIF ...      34.60 Deg.
              C10C -C48C -C49C    2_556   1_555   1_555 ......     # 2750 Check
PLAT779_ALERT_4_G Suspect or Irrelevant (Bond) Angle(s) in CIF ...       8.70 Deg.
              C9C  -C48C -C71C    2_556   1_555   1_555 ......     # 2756 Check
PLAT779_ALERT_4_G Suspect or Irrelevant (Bond) Angle(s) in CIF ...      21.50 Deg.
              C47C -C48C -C58C    1_555   1_555   2_556 ......     # 2768 Check
PLAT779_ALERT_4_G Suspect or Irrelevant (Bond) Angle(s) in CIF ...       7.10 Deg.
              C11C -C49C -C57C    2_556   1_555   1_555 ......     # 2788 Check
PLAT779_ALERT_4_G Suspect or Irrelevant (Bond) Angle(s) in CIF ...      34.10 Deg.
              C10C -C49C -C48C    2_556   1_555   1_555 ......     # 2790 Check
PLAT779_ALERT_4_G Suspect or Irrelevant (Bond) Angle(s) in CIF ...       1.10 Deg.
              C50C -C49C -C62C    1_555   1_555   2_556 ......     # 2798 Check
PLAT779_ALERT_4_G Suspect or Irrelevant (Bond) Angle(s) in CIF ...      13.19 Deg.
              ND2C -C49C -ND1C    1_555   1_555   2_556 ......     # 2824 Check
PLAT779_ALERT_4_G Suspect or Irrelevant (Bond) Angle(s) in CIF ...      37.60 Deg.
```

```
              C64C -C50C -C55C    2_556   1_555   1_555 ......     # 2834 Check
PLAT779_ALERT_4_G Suspect or Irrelevant (Bond) Angle(s) in CIF ...      21.00 Deg.
              C62C -C50C -C61C    2_556   1_555   2_556 ......     # 2840 Check
PLAT779_ALERT_4_G Suspect or Irrelevant (Bond) Angle(s) in CIF ...      44.90 Deg.
              C51C -C50C -C61C    1_555   1_555   2_556 ......     # 2845 Check
PLAT779_ALERT_4_G Suspect or Irrelevant (Bond) Angle(s) in CIF ...      14.14 Deg.
              ND2C -C50C -ND1C    1_555   1_555   2_556 ......     # 2860 Check
PLAT779_ALERT_4_G Suspect or Irrelevant (Bond) Angle(s) in CIF ...      42.60 Deg.
              ND1C -C50C -ND3C    2_556   1_555   1_555 ......     # 2869 Check
PLAT779_ALERT_4_G Suspect or Irrelevant (Bond) Angle(s) in CIF ...      13.02 Deg.
              ND3C -C50C -ND4C    1_555   1_555   2_556 ......     # 2879 Check
PLAT779_ALERT_4_G Suspect or Irrelevant (Bond) Angle(s) in CIF ...      29.70 Deg.
              C60C -C51C -C52C    2_556   1_555   1_555 ......     # 2884 Check
PLAT779_ALERT_4_G Suspect or Irrelevant (Bond) Angle(s) in CIF ...       6.30 Deg.
              C62C -C51C -C50C    2_556   1_555   1_555 ......     # 2888 Check
PLAT779_ALERT_4_G Suspect or Irrelevant (Bond) Angle(s) in CIF ...      29.80 Deg.
              C72C -C51C -C59C    1_555   1_555   2_556 ......     # 2900 Check
PLAT779_ALERT_4_G Suspect or Irrelevant (Bond) Angle(s) in CIF ...      23.00 Deg.
              C72C -C51C -C58C    1_555   1_555   2_556 ......     # 2913 Check
PLAT779_ALERT_4_G Suspect or Irrelevant (Bond) Angle(s) in CIF ...      15.12 Deg.
              ND4C -C51C -ND3C    2_556   1_555   1_555 ......     # 2934 Check
PLAT779_ALERT_4_G Suspect or Irrelevant (Bond) Angle(s) in CIF ...       1.50 Deg.
              C77C -C52C -C70C    1_555   1_555   2_556 ......     # 2949 Check
PLAT779_ALERT_4_G Suspect or Irrelevant (Bond) Angle(s) in CIF ...      16.10 Deg.
              C53C -C52C -C67C    1_555   1_555   2_556 ......     # 2952 Check
PLAT779_ALERT_4_G Suspect or Irrelevant (Bond) Angle(s) in CIF ...      16.76 Deg.
              ND3C -C52C -ND4C    1_555   1_555   2_556 ......     # 2970 Check
PLAT779_ALERT_4_G Suspect or Irrelevant (Bond) Angle(s) in CIF ...      16.50 Deg.
              C68C -C53C -C79C    2_556   1_555   1_555 ......     # 2973 Check
PLAT779_ALERT_4_G Suspect or Irrelevant (Bond) Angle(s) in CIF ...      42.70 Deg.
              C61C -C53C -C52C    2_556   1_555   1_555 ......     # 2980 Check
PLAT779_ALERT_4_G Suspect or Irrelevant (Bond) Angle(s) in CIF ...       8.20 Deg.
              C67C -C53C -C54C    2_556   1_555   1_555 ......     # 2981 Check
PLAT779_ALERT_4_G Suspect or Irrelevant (Bond) Angle(s) in CIF ...      44.50 Deg.
              C68C -C53C -C69C    2_556   1_555   2_556 ......     # 2987 Check
PLAT779_ALERT_4_G Suspect or Irrelevant (Bond) Angle(s) in CIF ...      28.10 Deg.
              C79C -C53C -C69C    1_555   1_555   2_556 ......     # 2988 Check
PLAT779_ALERT_4_G Suspect or Irrelevant (Bond) Angle(s) in CIF ...      16.30 Deg.
              C67C -C53C -C66C    2_556   1_555   2_556 ......     # 2992 Check
PLAT779_ALERT_4_G Suspect or Irrelevant (Bond) Angle(s) in CIF ...      13.60 Deg.
              C54C -C53C -C66C    1_555   1_555   2_556 ......     # 2997 Check
PLAT779_ALERT_4_G Suspect or Irrelevant (Bond) Angle(s) in CIF ...      12.64 Deg.
              ND3C -C53C -ND4C    1_555   1_555   2_556 ......     # 3015 Check
PLAT779_ALERT_4_G Suspect or Irrelevant (Bond) Angle(s) in CIF ...      15.60 Deg.
              C66C -C54C -C80C    2_556   1_555   1_555 ......     # 3019 Check
PLAT779_ALERT_4_G Suspect or Irrelevant (Bond) Angle(s) in CIF ...       5.70 Deg.
              C67C -C54C -C53C    2_556   1_555   1_555 ......     # 3023 Check
PLAT779_ALERT_4_G Suspect or Irrelevant (Bond) Angle(s) in CIF ...      33.50 Deg.
              C66C -C54C -C25C    2_556   1_555   2_556 ......     # 3037 Check
PLAT779_ALERT_4_G Suspect or Irrelevant (Bond) Angle(s) in CIF ...      18.40 Deg.
              C80C -C54C -C25C    1_555   1_555   2_556 ......     # 3040 Check
PLAT779_ALERT_4_G Suspect or Irrelevant (Bond) Angle(s) in CIF ...      42.82 Deg.
              ND3C -C54C -ND1C    1_555   1_555   2_556 ......     # 3060 Check
PLAT779_ALERT_4_G Suspect or Irrelevant (Bond) Angle(s) in CIF ...      40.20 Deg.
              C64C -C55C -C56C    2_556   1_555   1_555 ......     # 3064 Check
PLAT779_ALERT_4_G Suspect or Irrelevant (Bond) Angle(s) in CIF ...      44.60 Deg.
              C65C -C55C -C56C    2_556   1_555   1_555 ......     # 3065 Check
```

```
PLAT779_ALERT_4_G Suspect or Irrelevant (Bond) Angle(s) in CIF ...       6.40 Deg.
              C62C -C55C -C50C    2_556   1_555   1_555 ......     # 3073 Check
PLAT779_ALERT_4_G Suspect or Irrelevant (Bond) Angle(s) in CIF ...      21.10 Deg.
              C54C -C55C -C66C    1_555   1_555   2_556 ......     # 3080 Check
PLAT779_ALERT_4_G Suspect or Irrelevant (Bond) Angle(s) in CIF ...      22.80 Deg.
              C54C -C55C -C67C    1_555   1_555   2_556 ......     # 3093 Check
PLAT779_ALERT_4_G Suspect or Irrelevant (Bond) Angle(s) in CIF ...      43.80 Deg.
              C66C -C55C -C67C    2_556   1_555   2_556 ......     # 3095 Check
PLAT779_ALERT_4_G Suspect or Irrelevant (Bond) Angle(s) in CIF ...      43.80 Deg.
              C61C -C55C -C67C    2_556   1_555   2_556 ......     # 3096 Check
PLAT779_ALERT_4_G Suspect or Irrelevant (Bond) Angle(s) in CIF ...      13.39 Deg.
              ND1C -C55C -ND2C    2_556   1_555   1_555 ......     # 3115 Check
PLAT779_ALERT_4_G Suspect or Irrelevant (Bond) Angle(s) in CIF ...      42.71 Deg.
              ND1C -C55C -ND3C    2_556   1_555   1_555 ......     # 3125 Check
PLAT779_ALERT_4_G Suspect or Irrelevant (Bond) Angle(s) in CIF ...      40.20 Deg.
              C64C -C56C -C55C    2_556   1_555   1_555 ......     # 3130 Check
PLAT779_ALERT_4_G Suspect or Irrelevant (Bond) Angle(s) in CIF ...       5.90 Deg.
              C57C -C56C -C11C    1_555   1_555   2_556 ......     # 3139 Check
PLAT779_ALERT_4_G Suspect or Irrelevant (Bond) Angle(s) in CIF ...       3.80 Deg.
              C30C -C56C -C18C    1_555   1_555   2_556 ......     # 3146 Check
PLAT779_ALERT_4_G Suspect or Irrelevant (Bond) Angle(s) in CIF ...      15.23 Deg.
              ND1C -C56C -ND2C    2_556   1_555   1_555 ......     # 3162 Check
PLAT779_ALERT_4_G Suspect or Irrelevant (Bond) Angle(s) in CIF ...      38.70 Deg.
              C64C -C57C -C56C    2_556   1_555   1_555 ......     # 3167 Check
PLAT779_ALERT_4_G Suspect or Irrelevant (Bond) Angle(s) in CIF ...      41.00 Deg.
              C11C -C57C -C49C    2_556   1_555   1_555 ......     # 3169 Check
PLAT779_ALERT_4_G Suspect or Irrelevant (Bond) Angle(s) in CIF ...      23.90 Deg.
              C12C -C57C -C36C    2_556   1_555   1_555 ......     # 3175 Check
PLAT779_ALERT_4_G Suspect or Irrelevant (Bond) Angle(s) in CIF ...       8.00 Deg.
              C11C -C57C -C10C    2_556   1_555   2_556 ......     # 3178 Check
PLAT779_ALERT_4_G Suspect or Irrelevant (Bond) Angle(s) in CIF ...      32.70 Deg.
              C49C -C57C -C10C    1_555   1_555   2_556 ......     # 3182 Check
PLAT779_ALERT_4_G Suspect or Irrelevant (Bond) Angle(s) in CIF ...      15.71 Deg.
              ND2C -C57C -ND1C    1_555   1_555   2_556 ......     # 3198 Check
PLAT779_ALERT_4_G Suspect or Irrelevant (Bond) Angle(s) in CIF ...      13.80 Deg.
              C72C -C58C -C59C    2_556   1_555   1_555 ......     # 3202 Check
PLAT779_ALERT_4_G Suspect or Irrelevant (Bond) Angle(s) in CIF ...      35.70 Deg.
              C47C -C58C -C3C     2_556   1_555   1_555 ......     # 3206 Check
PLAT779_ALERT_4_G Suspect or Irrelevant (Bond) Angle(s) in CIF ...      44.40 Deg.
              C47C -C58C -C48C    2_556   1_555   2_556 ......     # 3215 Check
PLAT779_ALERT_4_G Suspect or Irrelevant (Bond) Angle(s) in CIF ...      44.10 Deg.
              C63C -C58C -C48C    1_555   1_555   2_556 ......     # 3216 Check
PLAT779_ALERT_4_G Suspect or Irrelevant (Bond) Angle(s) in CIF ...      10.60 Deg.
              C3C  -C58C -C46C    1_555   1_555   2_556 ......     # 3224 Check
PLAT779_ALERT_4_G Suspect or Irrelevant (Bond) Angle(s) in CIF ...      42.70 Deg.
              C72C -C58C -C73C    2_556   1_555   2_556 ......     # 3227 Check
PLAT779_ALERT_4_G Suspect or Irrelevant (Bond) Angle(s) in CIF ...      32.90 Deg.
              C59C -C58C -C73C    1_555   1_555   2_556 ......     # 3230 Check
PLAT779_ALERT_4_G Suspect or Irrelevant (Bond) Angle(s) in CIF ...      25.00 Deg.
              C73C -C59C -C41C    2_556   1_555   1_555 ......     # 3248 Check
PLAT779_ALERT_4_G Suspect or Irrelevant (Bond) Angle(s) in CIF ...      13.00 Deg.
              C72C -C59C -C58C    2_556   1_555   1_555 ......     # 3250 Check
PLAT779_ALERT_4_G Suspect or Irrelevant (Bond) Angle(s) in CIF ...      30.10 Deg.
              C41C -C59C -C76C    1_555   1_555   2_556 ......     # 3262 Check
PLAT779_ALERT_4_G Suspect or Irrelevant (Bond) Angle(s) in CIF ...      10.23 Deg.
              ND4C -C59C -ND3C    1_555   1_555   2_556 ......     # 3279 Check
PLAT779_ALERT_4_G Suspect or Irrelevant (Bond) Angle(s) in CIF ...       1.20 Deg.
```

```
              C77C -C60C -C70C    2_556   1_555   1_555 ......     # 3285 Check
PLAT779_ALERT_4_G Suspect or Irrelevant (Bond) Angle(s) in CIF ...      39.90 Deg.
              C52C -C60C -C61C    2_556   1_555   1_555 ......     # 3290 Check
PLAT779_ALERT_4_G Suspect or Irrelevant (Bond) Angle(s) in CIF ...      20.70 Deg.
              C59C -C60C -C72C    1_555   1_555   2_556 ......     # 3299 Check
PLAT779_ALERT_4_G Suspect or Irrelevant (Bond) Angle(s) in CIF ...      15.82 Deg.
              ND4C -C60C -ND3C    1_555   1_555   2_556 ......     # 3324 Check
PLAT779_ALERT_4_G Suspect or Irrelevant (Bond) Angle(s) in CIF ...      24.70 Deg.
              C53C -C61C -C67C    2_556   1_555   1_555 ......     # 3333 Check
PLAT779_ALERT_4_G Suspect or Irrelevant (Bond) Angle(s) in CIF ...      26.30 Deg.
              C52C -C61C -C60C    2_556   1_555   1_555 ......     # 3335 Check
PLAT779_ALERT_4_G Suspect or Irrelevant (Bond) Angle(s) in CIF ...       2.40 Deg.
              C62C -C61C -C50C    1_555   1_555   2_556 ......     # 3343 Check
PLAT779_ALERT_4_G Suspect or Irrelevant (Bond) Angle(s) in CIF ...      27.90 Deg.
              C67C -C61C -C54C    1_555   1_555   2_556 ......     # 3350 Check
PLAT779_ALERT_4_G Suspect or Irrelevant (Bond) Angle(s) in CIF ...      44.50 Deg.
              C54C -C61C -C55C    2_556   1_555   2_556 ......     # 3360 Check
PLAT779_ALERT_4_G Suspect or Irrelevant (Bond) Angle(s) in CIF ...      15.47 Deg.
              ND3C -C61C -ND4C    2_556   1_555   1_555 ......     # 3379 Check
PLAT779_ALERT_4_G Suspect or Irrelevant (Bond) Angle(s) in CIF ...      36.00 Deg.
              C50C -C62C -C63C    2_556   1_555   1_555 ......     # 3383 Check
PLAT779_ALERT_4_G Suspect or Irrelevant (Bond) Angle(s) in CIF ...      37.80 Deg.
              C55C -C62C -C64C    2_556   1_555   1_555 ......     # 3391 Check
PLAT779_ALERT_4_G Suspect or Irrelevant (Bond) Angle(s) in CIF ...       9.00 Deg.
              C50C -C62C -C49C    2_556   1_555   2_556 ......     # 3395 Check
PLAT779_ALERT_4_G Suspect or Irrelevant (Bond) Angle(s) in CIF ...      44.30 Deg.
              C63C -C62C -C49C    1_555   1_555   2_556 ......     # 3398 Check
PLAT779_ALERT_4_G Suspect or Irrelevant (Bond) Angle(s) in CIF ...      13.93 Deg.
              ND2C -C62C -ND1C    2_556   1_555   1_555 ......     # 3423 Check
PLAT779_ALERT_4_G Suspect or Irrelevant (Bond) Angle(s) in CIF ...      42.79 Deg.
              ND3C -C62C -ND1C    2_556   1_555   1_555 ......     # 3424 Check
PLAT779_ALERT_4_G Suspect or Irrelevant (Bond) Angle(s) in CIF ...      13.19 Deg.
              ND3C -C62C -ND4C    2_556   1_555   1_555 ......     # 3433 Check
PLAT779_ALERT_4_G Suspect or Irrelevant (Bond) Angle(s) in CIF ...       4.50 Deg.
              C50C -C63C -C62C    2_556   1_555   1_555 ......     # 3442 Check
PLAT779_ALERT_4_G Suspect or Irrelevant (Bond) Angle(s) in CIF ...      38.60 Deg.
              C49C -C63C -C10C    2_556   1_555   1_555 ......     # 3445 Check
PLAT779_ALERT_4_G Suspect or Irrelevant (Bond) Angle(s) in CIF ...      37.30 Deg.
              C48C -C63C -C10C    2_556   1_555   1_555 ......     # 3447 Check
PLAT779_ALERT_4_G Suspect or Irrelevant (Bond) Angle(s) in CIF ...      28.80 Deg.
              C58C -C63C -C47C    1_555   1_555   2_556 ......     # 3453 Check
PLAT779_ALERT_4_G Suspect or Irrelevant (Bond) Angle(s) in CIF ...      21.70 Deg.
              C58C -C63C -C72C    1_555   1_555   2_556 ......     # 3466 Check
PLAT779_ALERT_4_G Suspect or Irrelevant (Bond) Angle(s) in CIF ...      11.98 Deg.
              ND2C -C63C -ND1C    2_556   1_555   1_555 ......     # 3499 Check
PLAT779_ALERT_4_G Suspect or Irrelevant (Bond) Angle(s) in CIF ...      43.40 Deg.
              C56C -C64C -C65C    2_556   1_555   1_555 ......     # 3508 Check
PLAT779_ALERT_4_G Suspect or Irrelevant (Bond) Angle(s) in CIF ...       6.60 Deg.
              C50C -C64C -C62C    2_556   1_555   1_555 ......     # 3514 Check
PLAT779_ALERT_4_G Suspect or Irrelevant (Bond) Angle(s) in CIF ...       9.10 Deg.
              C57C -C64C -C11C    2_556   1_555   1_555 ......     # 3518 Check
PLAT779_ALERT_4_G Suspect or Irrelevant (Bond) Angle(s) in CIF ...      15.59 Deg.
              ND1C -C64C -ND2C    1_555   1_555   2_556 ......     # 3545 Check
PLAT779_ALERT_4_G Suspect or Irrelevant (Bond) Angle(s) in CIF ...      38.80 Deg.
              C56C -C65C -C64C    2_556   1_555   1_555 ......     # 3552 Check
PLAT779_ALERT_4_G Suspect or Irrelevant (Bond) Angle(s) in CIF ...      39.00 Deg.
              C55C -C65C -C64C    2_556   1_555   1_555 ......     # 3553 Check
```

```
PLAT779_ALERT_4_G Suspect or Irrelevant (Bond) Angle(s) in CIF ...       9.40 Deg.
             C30C -C65C -C18C    2_556   1_555   1_555 ......     # 3558 Check
PLAT779_ALERT_4_G Suspect or Irrelevant (Bond) Angle(s) in CIF ...      22.90 Deg.
             C66C -C65C -C54C    1_555   1_555   2_556 ......     # 3564 Check
PLAT779_ALERT_4_G Suspect or Irrelevant (Bond) Angle(s) in CIF ...      26.40 Deg.
             C66C -C65C -C80C    1_555   1_555   2_556 ......     # 3577 Check
PLAT779_ALERT_4_G Suspect or Irrelevant (Bond) Angle(s) in CIF ...      11.41 Deg.
             ND1C -C65C -ND2C    1_555   1_555   2_556 ......     # 3600 Check
PLAT779_ALERT_4_G Suspect or Irrelevant (Bond) Angle(s) in CIF ...      27.10 Deg.
             C54C -C66C -C67C    2_556   1_555   1_555 ......     # 3602 Check
PLAT779_ALERT_4_G Suspect or Irrelevant (Bond) Angle(s) in CIF ...      21.20 Deg.
             C80C -C66C -C25C    2_556   1_555   1_555 ......     # 3605 Check
PLAT779_ALERT_4_G Suspect or Irrelevant (Bond) Angle(s) in CIF ...      44.20 Deg.
             C65C -C66C -C55C    1_555   1_555   2_556 ......     # 3615 Check
PLAT779_ALERT_4_G Suspect or Irrelevant (Bond) Angle(s) in CIF ...      42.60 Deg.
             C80C -C66C -C29C    2_556   1_555   2_556 ......     # 3617 Check
PLAT779_ALERT_4_G Suspect or Irrelevant (Bond) Angle(s) in CIF ...      32.90 Deg.
             C54C -C66C -C53C    2_556   1_555   2_556 ......     # 3622 Check
PLAT779_ALERT_4_G Suspect or Irrelevant (Bond) Angle(s) in CIF ...       7.00 Deg.
             C67C -C66C -C53C    1_555   1_555   2_556 ......     # 3624 Check
PLAT779_ALERT_4_G Suspect or Irrelevant (Bond) Angle(s) in CIF ...      40.94 Deg.
             ND1C -C66C -ND3C    1_555   1_555   2_556 ......     # 3645 Check
PLAT779_ALERT_4_G Suspect or Irrelevant (Bond) Angle(s) in CIF ...      19.40 Deg.
             C54C -C67C -C66C    2_556   1_555   1_555 ......     # 3648 Check
PLAT779_ALERT_4_G Suspect or Irrelevant (Bond) Angle(s) in CIF ...      41.80 Deg.
             C53C -C67C -C79C    2_556   1_555   2_556 ......     # 3656 Check
PLAT779_ALERT_4_G Suspect or Irrelevant (Bond) Angle(s) in CIF ...       9.20 Deg.
             C68C -C67C -C79C    1_555   1_555   2_556 ......     # 3659 Check
PLAT779_ALERT_4_G Suspect or Irrelevant (Bond) Angle(s) in CIF ...      39.90 Deg.
             C53C -C67C -C52C    2_556   1_555   2_556 ......     # 3661 Check
PLAT779_ALERT_4_G Suspect or Irrelevant (Bond) Angle(s) in CIF ...      34.10 Deg.
             C61C -C67C -C52C    1_555   1_555   2_556 ......     # 3665 Check
PLAT779_ALERT_4_G Suspect or Irrelevant (Bond) Angle(s) in CIF ...      38.30 Deg.
             C54C -C67C -C55C    2_556   1_555   2_556 ......     # 3668 Check
PLAT779_ALERT_4_G Suspect or Irrelevant (Bond) Angle(s) in CIF ...      38.20 Deg.
             C54C -C67C -C80C    2_556   1_555   2_556 ......     # 3675 Check
PLAT779_ALERT_4_G Suspect or Irrelevant (Bond) Angle(s) in CIF ...      19.20 Deg.
             C66C -C67C -C80C    1_555   1_555   2_556 ......     # 3676 Check
PLAT779_ALERT_4_G Suspect or Irrelevant (Bond) Angle(s) in CIF ...      10.61 Deg.
             ND3C -C67C -ND4C    2_556   1_555   1_555 ......     # 3700 Check
PLAT779_ALERT_4_G Suspect or Irrelevant (Bond) Angle(s) in CIF ...      16.80 Deg.
             C79C -C68C -C69C    2_556   1_555   1_555 ......     # 3704 Check
PLAT779_ALERT_4_G Suspect or Irrelevant (Bond) Angle(s) in CIF ...      24.20 Deg.
             C53C -C68C -C67C    2_556   1_555   1_555 ......     # 3708 Check
PLAT779_ALERT_4_G Suspect or Irrelevant (Bond) Angle(s) in CIF ...      22.60 Deg.
             C26C -C68C -C21C    1_555   1_555   2_556 ......     # 3713 Check
PLAT779_ALERT_4_G Suspect or Irrelevant (Bond) Angle(s) in CIF ...      20.40 Deg.
             C79C -C68C -C78C    2_556   1_555   2_556 ......     # 3716 Check
PLAT779_ALERT_4_G Suspect or Irrelevant (Bond) Angle(s) in CIF ...       3.60 Deg.
             C69C -C68C -C78C    1_555   1_555   2_556 ......     # 3719 Check
PLAT779_ALERT_4_G Suspect or Irrelevant (Bond) Angle(s) in CIF ...      10.39 Deg.
             ND3C -C68C -ND4C    2_556   1_555   1_555 ......     # 3736 Check
PLAT779_ALERT_4_G Suspect or Irrelevant (Bond) Angle(s) in CIF ...       7.50 Deg.
             C79C -C69C -C68C    2_556   1_555   1_555 ......     # 3739 Check
PLAT779_ALERT_4_G Suspect or Irrelevant (Bond) Angle(s) in CIF ...       2.20 Deg.
             C70C -C69C -C77C    1_555   1_555   2_556 ......     # 3751 Check
PLAT779_ALERT_4_G Suspect or Irrelevant (Bond) Angle(s) in CIF ...      23.30 Deg.
```

```
              C33C -C69C -C15C    1_555   1_555   2_556 ......      # 3755 Check
PLAT779_ALERT_4_G Suspect or Irrelevant (Bond) Angle(s) in CIF ...      42.10 Deg.
              C79C -C69C -C53C    2_556   1_555   2_556 ......      # 3759 Check
PLAT779_ALERT_4_G Suspect or Irrelevant (Bond) Angle(s) in CIF ...      34.70 Deg.
              C68C -C69C -C53C    1_555   1_555   2_556 ......      # 3760 Check
PLAT779_ALERT_4_G Suspect or Irrelevant (Bond) Angle(s) in CIF ...      14.09 Deg.
              ND3C -C69C -ND4C    2_556   1_555   1_555 ......      # 3781 Check
PLAT779_ALERT_4_G Suspect or Irrelevant (Bond) Angle(s) in CIF ...      25.00 Deg.
              C77C -C70C -C60C    2_556   1_555   1_555 ......      # 3783 Check
PLAT779_ALERT_4_G Suspect or Irrelevant (Bond) Angle(s) in CIF ...      35.00 Deg.
              C77C -C70C -C52C    2_556   1_555   2_556 ......      # 3788 Check
PLAT779_ALERT_4_G Suspect or Irrelevant (Bond) Angle(s) in CIF ...      28.30 Deg.
              C60C -C70C -C52C    1_555   1_555   2_556 ......      # 3790 Check
PLAT779_ALERT_4_G Suspect or Irrelevant (Bond) Angle(s) in CIF ...      19.10 Deg.
              C40C -C70C -C76C    1_555   1_555   2_556 ......      # 3795 Check
PLAT779_ALERT_4_G Suspect or Irrelevant (Bond) Angle(s) in CIF ...      14.30 Deg.
              C78C -C70C -C69C    2_556   1_555   1_555 ......      # 3798 Check
PLAT779_ALERT_4_G Suspect or Irrelevant (Bond) Angle(s) in CIF ...      43.00 Deg.
              C77C -C70C -ND4C    2_556   1_555   1_555 ......      # 3803 Check
PLAT779_ALERT_4_G Suspect or Irrelevant (Bond) Angle(s) in CIF ...      15.27 Deg.
              ND4C -C70C -ND3C    1_555   1_555   2_556 ......      # 3817 Check
PLAT779_ALERT_4_G Suspect or Irrelevant (Bond) Angle(s) in CIF ...      25.60 Deg.
              C8C  -C71C -C37C    2_556   1_555   1_555 ......      # 3822 Check
PLAT779_ALERT_4_G Suspect or Irrelevant (Bond) Angle(s) in CIF ...      43.50 Deg.
              C44C -C71C -C4C     1_555   1_555   2_556 ......      # 3826 Check
PLAT779_ALERT_4_G Suspect or Irrelevant (Bond) Angle(s) in CIF ...      37.00 Deg.
              C9C  -C71C -C10C    2_556   1_555   2_556 ......      # 3833 Check
PLAT779_ALERT_4_G Suspect or Irrelevant (Bond) Angle(s) in CIF ...      32.50 Deg.
              C48C -C71C -C10C    1_555   1_555   2_556 ......      # 3838 Check
PLAT779_ALERT_4_G Suspect or Irrelevant (Bond) Angle(s) in CIF ...      25.00 Deg.
              C9C  -C71C -ND2C    2_556   1_555   1_555 ......      # 3839 Check
PLAT779_ALERT_4_G Suspect or Irrelevant (Bond) Angle(s) in CIF ...      43.80 Deg.
              C59C -C72C -C73C    2_556   1_555   1_555 ......      # 3848 Check
PLAT779_ALERT_4_G Suspect or Irrelevant (Bond) Angle(s) in CIF ...      11.60 Deg.
              C58C -C72C -C47C    2_556   1_555   1_555 ......      # 3849 Check
PLAT779_ALERT_4_G Suspect or Irrelevant (Bond) Angle(s) in CIF ...      40.40 Deg.
              C59C -C72C -C60C    2_556   1_555   2_556 ......      # 3862 Check
PLAT779_ALERT_4_G Suspect or Irrelevant (Bond) Angle(s) in CIF ...      40.30 Deg.
              C51C -C72C -C60C    1_555   1_555   2_556 ......      # 3865 Check
PLAT779_ALERT_4_G Suspect or Irrelevant (Bond) Angle(s) in CIF ...      33.50 Deg.
              C59C -C72C -C41C    2_556   1_555   2_556 ......      # 3868 Check
PLAT779_ALERT_4_G Suspect or Irrelevant (Bond) Angle(s) in CIF ...      11.40 Deg.
              C73C -C72C -C41C    1_555   1_555   2_556 ......      # 3869 Check
PLAT779_ALERT_4_G Suspect or Irrelevant (Bond) Angle(s) in CIF ...      35.70 Deg.
              C58C -C72C -C3C     2_556   1_555   2_556 ......      # 3874 Check
PLAT779_ALERT_4_G Suspect or Irrelevant (Bond) Angle(s) in CIF ...      27.10 Deg.
              C47C -C72C -C3C     1_555   1_555   2_556 ......      # 3877 Check
PLAT779_ALERT_4_G Suspect or Irrelevant (Bond) Angle(s) in CIF ...      10.07 Deg.
              ND4C -C72C -ND3C    2_556   1_555   1_555 ......      # 3900 Check
PLAT779_ALERT_4_G Suspect or Irrelevant (Bond) Angle(s) in CIF ...      31.00 Deg.
              C59C -C73C -C72C    2_556   1_555   1_555 ......      # 3905 Check
PLAT779_ALERT_4_G Suspect or Irrelevant (Bond) Angle(s) in CIF ...      24.30 Deg.
              C41C -C73C -C76C    2_556   1_555   1_555 ......      # 3907 Check
PLAT779_ALERT_4_G Suspect or Irrelevant (Bond) Angle(s) in CIF ...      31.70 Deg.
              C74C -C73C -C42C    1_555   1_555   2_556 ......      # 3913 Check
PLAT779_ALERT_4_G Suspect or Irrelevant (Bond) Angle(s) in CIF ...      20.00 Deg.
              C72C -C73C -C58C    1_555   1_555   2_556 ......      # 3919 Check
```

```
PLAT779_ALERT_4_G Suspect or Irrelevant (Bond) Angle(s) in CIF ...      21.60 Deg.
              C41C -C73C -C40C    2_556   1_555   2_556 ......     # 3922 Check
PLAT779_ALERT_4_G Suspect or Irrelevant (Bond) Angle(s) in CIF ...       3.40 Deg.
              C76C -C73C -C40C    1_555   1_555   2_556 ......     # 3926 Check
PLAT779_ALERT_4_G Suspect or Irrelevant (Bond) Angle(s) in CIF ...      37.50 Deg.
              C2C  -C74C -C46C    2_556   1_555   1_555 ......     # 3941 Check
PLAT779_ALERT_4_G Suspect or Irrelevant (Bond) Angle(s) in CIF ...      37.30 Deg.
              C42C -C74C -C6C     2_556   1_555   1_555 ......     # 3943 Check
PLAT779_ALERT_4_G Suspect or Irrelevant (Bond) Angle(s) in CIF ...      22.90 Deg.
              C73C -C74C -C41C    1_555   1_555   2_556 ......     # 3949 Check
PLAT779_ALERT_4_G Suspect or Irrelevant (Bond) Angle(s) in CIF ...      13.70 Deg.
              C46C -C74C -C3C     1_555   1_555   2_556 ......     # 3955 Check
PLAT779_ALERT_4_G Suspect or Irrelevant (Bond) Angle(s) in CIF ...      20.50 Deg.
              C40C -C75C -C76C    2_556   1_555   1_555 ......     # 3960 Check
PLAT779_ALERT_4_G Suspect or Irrelevant (Bond) Angle(s) in CIF ...      36.60 Deg.
              C38C -C75C -C7C     2_556   1_555   1_555 ......     # 3967 Check
PLAT779_ALERT_4_G Suspect or Irrelevant (Bond) Angle(s) in CIF ...       9.40 Deg.
              C39C -C75C -C14C    2_556   1_555   1_555 ......     # 3968 Check
PLAT779_ALERT_4_G Suspect or Irrelevant (Bond) Angle(s) in CIF ...      44.00 Deg.
              C38C -C75C -C43C    2_556   1_555   2_556 ......     # 3976 Check
PLAT779_ALERT_4_G Suspect or Irrelevant (Bond) Angle(s) in CIF ...      11.10 Deg.
              C7C  -C75C -C43C    1_555   1_555   2_556 ......     # 3977 Check
PLAT779_ALERT_4_G Suspect or Irrelevant (Bond) Angle(s) in CIF ...      29.20 Deg.
              C76C -C75C -C41C    1_555   1_555   2_556 ......     # 3981 Check
PLAT779_ALERT_4_G Suspect or Irrelevant (Bond) Angle(s) in CIF ...      17.20 Deg.
              C39C -C75C -C34C    2_556   1_555   2_556 ......     # 3986 Check
PLAT779_ALERT_4_G Suspect or Irrelevant (Bond) Angle(s) in CIF ...       8.00 Deg.
              C14C -C75C -C34C    1_555   1_555   2_556 ......     # 3991 Check
PLAT779_ALERT_4_G Suspect or Irrelevant (Bond) Angle(s) in CIF ...      17.50 Deg.
              C41C -C76C -C73C    2_556   1_555   1_555 ......     # 4007 Check
PLAT779_ALERT_4_G Suspect or Irrelevant (Bond) Angle(s) in CIF ...       2.50 Deg.
              C77C -C76C -C70C    1_555   1_555   2_556 ......     # 4017 Check
PLAT779_ALERT_4_G Suspect or Irrelevant (Bond) Angle(s) in CIF ...      35.20 Deg.
              C73C -C76C -C59C    1_555   1_555   2_556 ......     # 4021 Check
PLAT779_ALERT_4_G Suspect or Irrelevant (Bond) Angle(s) in CIF ...      43.20 Deg.
              C40C -C76C -C39C    2_556   1_555   2_556 ......     # 4024 Check
PLAT779_ALERT_4_G Suspect or Irrelevant (Bond) Angle(s) in CIF ...      18.60 Deg.
              C75C -C76C -C39C    1_555   1_555   2_556 ......     # 4026 Check
PLAT779_ALERT_4_G Suspect or Irrelevant (Bond) Angle(s) in CIF ...      40.50 Deg.
              C77C -C76C -C60C    1_555   1_555   2_556 ......     # 4035 Check
PLAT779_ALERT_4_G Suspect or Irrelevant (Bond) Angle(s) in CIF ...      42.80 Deg.
              C70C -C76C -C60C    2_556   1_555   2_556 ......     # 4036 Check
PLAT779_ALERT_4_G Suspect or Irrelevant (Bond) Angle(s) in CIF ...      43.00 Deg.
              C59C -C76C -C60C    2_556   1_555   2_556 ......     # 4037 Check
PLAT779_ALERT_4_G Suspect or Irrelevant (Bond) Angle(s) in CIF ...       9.51 Deg.
              ND4C -C76C -ND3C    2_556   1_555   1_555 ......     # 4057 Check
PLAT779_ALERT_4_G Suspect or Irrelevant (Bond) Angle(s) in CIF ...      29.40 Deg.
              C60C -C77C -C52C    2_556   1_555   1_555 ......     # 4065 Check
PLAT779_ALERT_4_G Suspect or Irrelevant (Bond) Angle(s) in CIF ...      19.20 Deg.
              C40C -C77C -C76C    2_556   1_555   1_555 ......     # 4071 Check
PLAT779_ALERT_4_G Suspect or Irrelevant (Bond) Angle(s) in CIF ...      14.30 Deg.
              C78C -C77C -C69C    1_555   1_555   2_556 ......     # 4075 Check
PLAT779_ALERT_4_G Suspect or Irrelevant (Bond) Angle(s) in CIF ...      15.56 Deg.
              ND4C -C77C -ND3C    2_556   1_555   1_555 ......     # 4093 Check
PLAT779_ALERT_4_G Suspect or Irrelevant (Bond) Angle(s) in CIF ...       2.30 Deg.
              C70C -C78C -C77C    2_556   1_555   1_555 ......     # 4098 Check
PLAT779_ALERT_4_G Suspect or Irrelevant (Bond) Angle(s) in CIF ...       5.00 Deg.
```

```
              C69C -C78C -C79C    2_556   1_555   1_555 ......     # 4100 Check
PLAT779_ALERT_4_G Suspect or Irrelevant (Bond) Angle(s) in CIF ...      27.90 Deg.
              C33C -C78C -C15C    2_556   1_555   1_555 ......     # 4106 Check
PLAT779_ALERT_4_G Suspect or Irrelevant (Bond) Angle(s) in CIF ...      11.70 Deg.
              C69C -C78C -C68C    2_556   1_555   2_556 ......     # 4109 Check
PLAT779_ALERT_4_G Suspect or Irrelevant (Bond) Angle(s) in CIF ...       6.30 Deg.
              C79C -C78C -C68C    1_555   1_555   2_556 ......     # 4113 Check
PLAT779_ALERT_4_G Suspect or Irrelevant (Bond) Angle(s) in CIF ...      14.36 Deg.
              ND3C -C78C -ND4C    1_555   1_555   2_556 ......     # 4129 Check
PLAT779_ALERT_4_G Suspect or Irrelevant (Bond) Angle(s) in CIF ...       2.40 Deg.
              C69C -C79C -C78C    2_556   1_555   1_555 ......     # 4134 Check
PLAT779_ALERT_4_G Suspect or Irrelevant (Bond) Angle(s) in CIF ...      20.90 Deg.
              C21C -C79C -C26C    1_555   1_555   2_556 ......     # 4144 Check
PLAT779_ALERT_4_G Suspect or Irrelevant (Bond) Angle(s) in CIF ...      30.70 Deg.
              C68C -C79C -C67C    2_556   1_555   2_556 ......     # 4145 Check
PLAT779_ALERT_4_G Suspect or Irrelevant (Bond) Angle(s) in CIF ...      16.90 Deg.
              C53C -C79C -C67C    1_555   1_555   2_556 ......     # 4147 Check
PLAT779_ALERT_4_G Suspect or Irrelevant (Bond) Angle(s) in CIF ...      39.60 Deg.
              C69C -C79C -C33C    2_556   1_555   2_556 ......     # 4152 Check
PLAT779_ALERT_4_G Suspect or Irrelevant (Bond) Angle(s) in CIF ...      41.50 Deg.
              C78C -C79C -C33C    1_555   1_555   2_556 ......     # 4154 Check
PLAT779_ALERT_4_G Suspect or Irrelevant (Bond) Angle(s) in CIF ...      11.55 Deg.
              ND3C -C79C -ND4C    1_555   1_555   2_556 ......     # 4174 Check
PLAT779_ALERT_4_G Suspect or Irrelevant (Bond) Angle(s) in CIF ...       5.40 Deg.
              C25C -C80C -C22C    2_556   1_555   1_555 ......     # 4178 Check
PLAT779_ALERT_4_G Suspect or Irrelevant (Bond) Angle(s) in CIF ...      11.80 Deg.
              C66C -C80C -C54C    2_556   1_555   1_555 ......     # 4182 Check
PLAT779_ALERT_4_G Suspect or Irrelevant (Bond) Angle(s) in CIF ...      31.10 Deg.
              C25C -C80C -C26C    2_556   1_555   2_556 ......     # 4196 Check
PLAT779_ALERT_4_G Suspect or Irrelevant (Bond) Angle(s) in CIF ...      29.50 Deg.
              C22C -C80C -C26C    1_555   1_555   2_556 ......     # 4199 Check
PLAT779_ALERT_4_G Suspect or Irrelevant (Bond) Angle(s) in CIF ...      33.30 Deg.
              C66C -C80C -C67C    2_556   1_555   2_556 ......     # 4204 Check
PLAT779_ALERT_4_G Suspect or Irrelevant (Bond) Angle(s) in CIF ...      22.20 Deg.
              C54C -C80C -C67C    1_555   1_555   2_556 ......     # 4207 Check
PLAT779_ALERT_4_G Suspect or Irrelevant (Bond) Angle(s) in CIF ...      24.10 Deg.
              C81C -C81C -F9      2_556   1_555   1_555 ......     # 4212 Check
PLAT779_ALERT_4_G Suspect or Irrelevant (Bond) Angle(s) in CIF ...      19.60 Deg.
              C1C  -C81C -C1C     1_555   1_555   2_556 ......     # 4231 Check
PLAT779_ALERT_4_G Suspect or Irrelevant (Bond) Angle(s) in CIF ...      44.00 Deg.
              C81C -C81C -F8      2_556   1_555   2_556 ......     # 4232 Check
PLAT779_ALERT_4_G Suspect or Irrelevant (Bond) Angle(s) in CIF ...      36.60 Deg.
              F9   -C81C -F8      1_555   1_555   2_556 ......     # 4234 Check
PLAT779_ALERT_4_G Suspect or Irrelevant (Bond) Angle(s) in CIF ...      40.00 Deg.
              C81C -C81C -F7      2_556   1_555   2_556 ......     # 4239 Check
PLAT779_ALERT_4_G Suspect or Irrelevant (Bond) Angle(s) in CIF ...      39.30 Deg.
              F9   -C81C -F7      1_555   1_555   2_556 ......     # 4241 Check
PLAT779_ALERT_4_G Suspect or Irrelevant (Bond) Angle(s) in CIF ...      35.80 Deg.
              F9   -F7   -C81C    2_556   1_555   1_555 ......     # 4247 Check
PLAT779_ALERT_4_G Suspect or Irrelevant (Bond) Angle(s) in CIF ...      16.90 Deg.
              C81C -F7   -C81C    1_555   1_555   2_556 ......     # 4249 Check
PLAT779_ALERT_4_G Suspect or Irrelevant (Bond) Angle(s) in CIF ...      34.70 Deg.
              F9   -F8   -C81C    2_556   1_555   1_555 ......     # 4250 Check
PLAT779_ALERT_4_G Suspect or Irrelevant (Bond) Angle(s) in CIF ...      17.90 Deg.
              C81C -F8   -C81C    1_555   1_555   2_556 ......     # 4252 Check
PLAT779_ALERT_4_G Suspect or Irrelevant (Bond) Angle(s) in CIF ...      18.20 Deg.
              C81C -F9   -C81C    2_556   1_555   1_555 ......     # 4256 Check
```

```
PLAT779_ALERT_4_G Suspect or Irrelevant (Bond) Angle(s) in CIF ...      41.90 Deg.
              C81C -F9   -F9      2_556   1_555   2_556 ......     # 4259 Check
PLAT779_ALERT_4_G Suspect or Irrelevant (Bond) Angle(s) in CIF ...      23.90 Deg.
              C81C -F9   -F9      1_555   1_555   2_556 ......     # 4262 Check
PLAT779_ALERT_4_G Suspect or Irrelevant (Bond) Angle(s) in CIF ...      35.70 Deg.
              C9B  -C1B  -C2B     2_656   1_555   1_555 ......     # 4531 Check
PLAT779_ALERT_4_G Suspect or Irrelevant (Bond) Angle(s) in CIF ...      33.30 Deg.
              C2B  -C1B  -C9B     2_656   1_555   1_555 ......     # 4541 Check
PLAT779_ALERT_4_G Suspect or Irrelevant (Bond) Angle(s) in CIF ...      13.80 Deg.
              C81B -C1B  -C81B    1_555   1_555   2_656 ......     # 4554 Check
PLAT779_ALERT_4_G Suspect or Irrelevant (Bond) Angle(s) in CIF ...      18.40 Deg.
              C1B  -C1B  -C5B     2_656   1_555   2_656 ......     # 4555 Check
PLAT779_ALERT_4_G Suspect or Irrelevant (Bond) Angle(s) in CIF ...      38.40 Deg.
              C8B  -C2B  -C3B     2_656   1_555   1_555 ......     # 4565 Check
PLAT779_ALERT_4_G Suspect or Irrelevant (Bond) Angle(s) in CIF ...      17.50 Deg.
              C1B  -C2B  -C1B     1_555   1_555   2_656 ......     # 4577 Check
PLAT779_ALERT_4_G Suspect or Irrelevant (Bond) Angle(s) in CIF ...      30.50 Deg.
              C66B -C2B  -C7B     1_555   1_555   2_656 ......     # 4581 Check
PLAT779_ALERT_4_G Suspect or Irrelevant (Bond) Angle(s) in CIF ...      38.90 Deg.
              C9B  -C3B  -C2B     2_656   1_555   1_555 ......     # 4598 Check
PLAT779_ALERT_4_G Suspect or Irrelevant (Bond) Angle(s) in CIF ...       9.30 Deg.
              C13B -C3B  -C42B    2_656   1_555   1_555 ......     # 4603 Check
PLAT779_ALERT_4_G Suspect or Irrelevant (Bond) Angle(s) in CIF ...      11.60 Deg.
              C4B  -C3B  -C10B    1_555   1_555   2_656 ......     # 4609 Check
PLAT779_ALERT_4_G Suspect or Irrelevant (Bond) Angle(s) in CIF ...      30.30 Deg.
              C4B  -C3B  -C11B    1_555   1_555   2_656 ......     # 4622 Check
PLAT779_ALERT_4_G Suspect or Irrelevant (Bond) Angle(s) in CIF ...      41.00 Deg.
              C10B -C3B  -C11B    2_656   1_555   2_656 ......     # 4625 Check
PLAT779_ALERT_4_G Suspect or Irrelevant (Bond) Angle(s) in CIF ...      17.80 Deg.
              C10B -C4B  -C5B     2_656   1_555   1_555 ......     # 4630 Check
PLAT779_ALERT_4_G Suspect or Irrelevant (Bond) Angle(s) in CIF ...      14.50 Deg.
              C11B -C4B  -C45B    2_656   1_555   1_555 ......     # 4638 Check
PLAT779_ALERT_4_G Suspect or Irrelevant (Bond) Angle(s) in CIF ...      23.90 Deg.
              C10B -C4B  -C71B    2_656   1_555   2_656 ......     # 4642 Check
PLAT779_ALERT_4_G Suspect or Irrelevant (Bond) Angle(s) in CIF ...      38.40 Deg.
              C5B  -C4B  -C71B    1_555   1_555   2_656 ......     # 4645 Check
PLAT779_ALERT_4_G Suspect or Irrelevant (Bond) Angle(s) in CIF ...       7.20 Deg.
              C10B -C5B  -C4B     2_656   1_555   1_555 ......     # 4649 Check
PLAT779_ALERT_4_G Suspect or Irrelevant (Bond) Angle(s) in CIF ...      30.30 Deg.
              C71B -C5B  -C6B     2_656   1_555   1_555 ......     # 4652 Check
PLAT779_ALERT_4_G Suspect or Irrelevant (Bond) Angle(s) in CIF ...      27.00 Deg.
              C6B  -C5B  -C66B    1_555   1_555   2_656 ......     # 4666 Check
PLAT779_ALERT_4_G Suspect or Irrelevant (Bond) Angle(s) in CIF ...       5.70 Deg.
              C1B  -C5B  -C1B     1_555   1_555   2_656 ......     # 4673 Check
PLAT779_ALERT_4_G Suspect or Irrelevant (Bond) Angle(s) in CIF ...      24.70 Deg.
              C66B -C6B  -C7B     2_656   1_555   1_555 ......     # 4691 Check
PLAT779_ALERT_4_G Suspect or Irrelevant (Bond) Angle(s) in CIF ...      20.00 Deg.
              C79B -C6B  -C70B    1_555   1_555   2_656 ......     # 4697 Check
PLAT779_ALERT_4_G Suspect or Irrelevant (Bond) Angle(s) in CIF ...      28.70 Deg.
              C7B  -C6B  -C67B    1_555   1_555   2_656 ......     # 4703 Check
PLAT779_ALERT_4_G Suspect or Irrelevant (Bond) Angle(s) in CIF ...      24.90 Deg.
              C67B -C7B  -C80B    2_656   1_555   1_555 ......     # 4709 Check
PLAT779_ALERT_4_G Suspect or Irrelevant (Bond) Angle(s) in CIF ...      21.60 Deg.
              C66B -C7B  -C6B     2_656   1_555   1_555 ......     # 4711 Check
PLAT779_ALERT_4_G Suspect or Irrelevant (Bond) Angle(s) in CIF ...      39.50 Deg.
              C8B  -C7B  -C2B     1_555   1_555   2_656 ......     # 4717 Check
PLAT779_ALERT_4_G Suspect or Irrelevant (Bond) Angle(s) in CIF ...      29.80 Deg.
```

```
              C80B -C7B  -C41B    1_555   1_555   2_656 ......     # 4723 Check
PLAT779_ALERT_4_G Suspect or Irrelevant (Bond) Angle(s) in CIF ...      38.70 Deg.
              C2B  -C8B  -C9B     2_656   1_555   1_555 ......     # 4730 Check
PLAT779_ALERT_4_G Suspect or Irrelevant (Bond) Angle(s) in CIF ...       8.80 Deg.
              C13B -C8B  -C42B    1_555   1_555   2_656 ......     # 4740 Check
PLAT779_ALERT_4_G Suspect or Irrelevant (Bond) Angle(s) in CIF ...      28.10 Deg.
              C7B  -C8B  -C66B    1_555   1_555   2_656 ......     # 4743 Check
PLAT779_ALERT_4_G Suspect or Irrelevant (Bond) Angle(s) in CIF ...      38.20 Deg.
              C3B  -C9B  -C8B     2_656   1_555   1_555 ......     # 4758 Check
PLAT779_ALERT_4_G Suspect or Irrelevant (Bond) Angle(s) in CIF ...      16.70 Deg.
              C10B -C9B  -C4B     1_555   1_555   2_656 ......     # 4768 Check
PLAT779_ALERT_4_G Suspect or Irrelevant (Bond) Angle(s) in CIF ...      40.80 Deg.
              C10B -C9B  -C5B     1_555   1_555   2_656 ......     # 4773 Check
PLAT779_ALERT_4_G Suspect or Irrelevant (Bond) Angle(s) in CIF ...      15.20 Deg.
              C1B  -C9B  -C1B     2_656   1_555   1_555 ......     # 4777 Check
PLAT779_ALERT_4_G Suspect or Irrelevant (Bond) Angle(s) in CIF ...      43.00 Deg.
              C4B  -C10B -C11B    2_656   1_555   1_555 ......     # 4783 Check
PLAT779_ALERT_4_G Suspect or Irrelevant (Bond) Angle(s) in CIF ...      16.40 Deg.
              C11B -C10B -C45B    1_555   1_555   2_656 ......     # 4794 Check
PLAT779_ALERT_4_G Suspect or Irrelevant (Bond) Angle(s) in CIF ...      41.50 Deg.
              C4B  -C10B -C3B     2_656   1_555   2_656 ......     # 4797 Check
PLAT779_ALERT_4_G Suspect or Irrelevant (Bond) Angle(s) in CIF ...      41.70 Deg.
              C9B  -C10B -C3B     1_555   1_555   2_656 ......     # 4800 Check
PLAT779_ALERT_4_G Suspect or Irrelevant (Bond) Angle(s) in CIF ...      16.10 Deg.
              C4B  -C11B -C10B    2_656   1_555   1_555 ......     # 4805 Check
PLAT779_ALERT_4_G Suspect or Irrelevant (Bond) Angle(s) in CIF ...      11.60 Deg.
              C45B -C11B -C72B    2_656   1_555   1_555 ......     # 4806 Check
PLAT779_ALERT_4_G Suspect or Irrelevant (Bond) Angle(s) in CIF ...      25.40 Deg.
              C45B -C11B -C46B    2_656   1_555   2_656 ......     # 4818 Check
PLAT779_ALERT_4_G Suspect or Irrelevant (Bond) Angle(s) in CIF ...      15.20 Deg.
              C72B -C11B -C46B    1_555   1_555   2_656 ......     # 4821 Check
PLAT779_ALERT_4_G Suspect or Irrelevant (Bond) Angle(s) in CIF ...      42.60 Deg.
              C4B  -C11B -C3B     2_656   1_555   2_656 ......     # 4825 Check
PLAT779_ALERT_4_G Suspect or Irrelevant (Bond) Angle(s) in CIF ...      38.70 Deg.
              C43B -C12B -C17B    2_656   1_555   1_555 ......     # 4834 Check
PLAT779_ALERT_4_G Suspect or Irrelevant (Bond) Angle(s) in CIF ...       9.20 Deg.
              C42B -C12B -C13B    2_656   1_555   1_555 ......     # 4839 Check
PLAT779_ALERT_4_G Suspect or Irrelevant (Bond) Angle(s) in CIF ...      15.80 Deg.
              C11B -C12B -C45B    1_555   1_555   2_656 ......     # 4851 Check
PLAT779_ALERT_4_G Suspect or Irrelevant (Bond) Angle(s) in CIF ...      36.50 Deg.
              C3B  -C13B -C8B     2_656   1_555   1_555 ......     # 4869 Check
PLAT779_ALERT_4_G Suspect or Irrelevant (Bond) Angle(s) in CIF ...      22.10 Deg.
              C14B -C13B -C41B    1_555   1_555   2_656 ......     # 4880 Check
PLAT779_ALERT_4_G Suspect or Irrelevant (Bond) Angle(s) in CIF ...      31.60 Deg.
              C12B -C13B -C43B    1_555   1_555   2_656 ......     # 4886 Check
PLAT779_ALERT_4_G Suspect or Irrelevant (Bond) Angle(s) in CIF ...       7.00 Deg.
              C42B -C13B -ND2B    2_656   1_555   1_555 ......     # 4888 Check
PLAT779_ALERT_4_G Suspect or Irrelevant (Bond) Angle(s) in CIF ...      18.90 Deg.
              C41B -C14B -C80B    2_656   1_555   1_555 ......     # 4898 Check
PLAT779_ALERT_4_G Suspect or Irrelevant (Bond) Angle(s) in CIF ...       9.40 Deg.
              C42B -C14B -C13B    2_656   1_555   1_555 ......     # 4903 Check
PLAT779_ALERT_4_G Suspect or Irrelevant (Bond) Angle(s) in CIF ...      44.50 Deg.
              C40B -C14B -C15B    2_656   1_555   1_555 ......     # 4906 Check
PLAT779_ALERT_4_G Suspect or Irrelevant (Bond) Angle(s) in CIF ...       5.00 Deg.
              C41B -C14B -C67B    2_656   1_555   2_656 ......     # 4910 Check
PLAT779_ALERT_4_G Suspect or Irrelevant (Bond) Angle(s) in CIF ...      13.90 Deg.
              C80B -C14B -C67B    1_555   1_555   2_656 ......     # 4913 Check
```

```
PLAT779_ALERT_4_G Suspect or Irrelevant (Bond) Angle(s) in CIF ...      12.40 Deg.
           C16B -C15B -C38B    1_555   1_555   2_656 ......     # 4936 Check
PLAT779_ALERT_4_G Suspect or Irrelevant (Bond) Angle(s) in CIF ...      12.30 Deg.
           C14B -C15B -C41B    1_555   1_555   2_656 ......     # 4940 Check
PLAT779_ALERT_4_G Suspect or Irrelevant (Bond) Angle(s) in CIF ...      10.48 Deg.
           ND2B -C15B -ND1B    1_555   1_555   2_656 ......     # 4958 Check
PLAT779_ALERT_4_G Suspect or Irrelevant (Bond) Angle(s) in CIF ...      27.90 Deg.
           C39B -C16B -C15B    2_656   1_555   1_555 ......     # 4963 Check
PLAT779_ALERT_4_G Suspect or Irrelevant (Bond) Angle(s) in CIF ...      21.00 Deg.
           C38B -C16B -C17B    2_656   1_555   1_555 ......     # 4965 Check
PLAT779_ALERT_4_G Suspect or Irrelevant (Bond) Angle(s) in CIF ...      13.00 Deg.
           C38B -C16B -C43B    2_656   1_555   2_656 ......     # 4974 Check
PLAT779_ALERT_4_G Suspect or Irrelevant (Bond) Angle(s) in CIF ...      33.30 Deg.
           C17B -C16B -C43B    1_555   1_555   2_656 ......     # 4978 Check
PLAT779_ALERT_4_G Suspect or Irrelevant (Bond) Angle(s) in CIF ...      13.06 Deg.
           ND2B -C16B -ND1B    1_555   1_555   2_656 ......     # 4994 Check
PLAT779_ALERT_4_G Suspect or Irrelevant (Bond) Angle(s) in CIF ...      32.70 Deg.
           C43B -C17B -C12B    2_656   1_555   1_555 ......     # 4998 Check
PLAT779_ALERT_4_G Suspect or Irrelevant (Bond) Angle(s) in CIF ...       5.70 Deg.
           C38B -C17B -C16B    2_656   1_555   1_555 ......     # 5012 Check
PLAT779_ALERT_4_G Suspect or Irrelevant (Bond) Angle(s) in CIF ...       9.50 Deg.
           C65B -C17B -C18B    2_656   1_555   1_555 ......     # 5021 Check
PLAT779_ALERT_4_G Suspect or Irrelevant (Bond) Angle(s) in CIF ...      12.12 Deg.
           ND2B -C17B -ND1B    1_555   1_555   2_656 ......     # 5039 Check
PLAT779_ALERT_4_G Suspect or Irrelevant (Bond) Angle(s) in CIF ...      41.50 Deg.
           C37B -C18B -C19B    2_656   1_555   1_555 ......     # 5042 Check
PLAT779_ALERT_4_G Suspect or Irrelevant (Bond) Angle(s) in CIF ...      36.90 Deg.
           C73B -C18B -C48B    1_555   1_555   2_656 ......     # 5049 Check
PLAT779_ALERT_4_G Suspect or Irrelevant (Bond) Angle(s) in CIF ...      11.87 Deg.
           ND1B -C18B -ND2B    2_656   1_555   1_555 ......     # 5075 Check
PLAT779_ALERT_4_G Suspect or Irrelevant (Bond) Angle(s) in CIF ...      44.80 Deg.
           ND1B -C18B -ND3B    2_656   1_555   2_656 ......     # 5083 Check
PLAT779_ALERT_4_G Suspect or Irrelevant (Bond) Angle(s) in CIF ...      10.75 Deg.
           ND3B -C18B -ND4B    2_656   1_555   1_555 ......     # 5094 Check
PLAT779_ALERT_4_G Suspect or Irrelevant (Bond) Angle(s) in CIF ...      10.10 Deg.
           C24B -C19B -C64B    1_555   1_555   2_656 ......     # 5108 Check
PLAT779_ALERT_4_G Suspect or Irrelevant (Bond) Angle(s) in CIF ...       4.30 Deg.
           C18B -C19B -C65B    1_555   1_555   2_656 ......     # 5112 Check
PLAT779_ALERT_4_G Suspect or Irrelevant (Bond) Angle(s) in CIF ...      10.27 Deg.
           ND1B -C19B -ND2B    2_656   1_555   1_555 ......     # 5130 Check
PLAT779_ALERT_4_G Suspect or Irrelevant (Bond) Angle(s) in CIF ...      43.40 Deg.
           ND1B -C19B -ND3B    2_656   1_555   2_656 ......     # 5138 Check
PLAT779_ALERT_4_G Suspect or Irrelevant (Bond) Angle(s) in CIF ...      42.20 Deg.
           C36B -C20B -C19B    2_656   1_555   1_555 ......     # 5141 Check
PLAT779_ALERT_4_G Suspect or Irrelevant (Bond) Angle(s) in CIF ...      41.80 Deg.
           C37B -C20B -C19B    2_656   1_555   1_555 ......     # 5142 Check
PLAT779_ALERT_4_G Suspect or Irrelevant (Bond) Angle(s) in CIF ...      13.20 Deg.
           C21B -C20B -C35B    1_555   1_555   2_656 ......     # 5154 Check
PLAT779_ALERT_4_G Suspect or Irrelevant (Bond) Angle(s) in CIF ...      10.50 Deg.
           C16B -C20B -C38B    1_555   1_555   2_656 ......     # 5158 Check
PLAT779_ALERT_4_G Suspect or Irrelevant (Bond) Angle(s) in CIF ...      36.70 Deg.
           C21B -C20B -C34B    1_555   1_555   2_656 ......     # 5165 Check
PLAT779_ALERT_4_G Suspect or Irrelevant (Bond) Angle(s) in CIF ...      39.30 Deg.
           C16B -C20B -C39B    1_555   1_555   2_656 ......     # 5171 Check
PLAT779_ALERT_4_G Suspect or Irrelevant (Bond) Angle(s) in CIF ...      44.20 Deg.
           C34B -C20B -C39B    2_656   1_555   2_656 ......     # 5175 Check
PLAT779_ALERT_4_G Suspect or Irrelevant (Bond) Angle(s) in CIF ...      12.69 Deg.
```

```
              ND1B -C20B -ND2B    2_656   1_555   1_555 ......     # 5194 Check
PLAT779_ALERT_4_G Suspect or Irrelevant (Bond) Angle(s) in CIF ...      37.00 Deg.
              C35B -C21B -C22B    2_656   1_555   1_555 ......     # 5196 Check
PLAT779_ALERT_4_G Suspect or Irrelevant (Bond) Angle(s) in CIF ...      26.70 Deg.
              C34B -C21B -C58B    2_656   1_555   1_555 ......     # 5199 Check
PLAT779_ALERT_4_G Suspect or Irrelevant (Bond) Angle(s) in CIF ...      24.90 Deg.
              C22B -C21B -C31B    1_555   1_555   2_656 ......     # 5207 Check
PLAT779_ALERT_4_G Suspect or Irrelevant (Bond) Angle(s) in CIF ...      40.70 Deg.
              C20B -C21B -C36B    1_555   1_555   2_656 ......     # 5214 Check
PLAT779_ALERT_4_G Suspect or Irrelevant (Bond) Angle(s) in CIF ...      10.52 Deg.
              ND1B -C21B -ND2B    2_656   1_555   1_555 ......     # 5230 Check
PLAT779_ALERT_4_G Suspect or Irrelevant (Bond) Angle(s) in CIF ...      33.10 Deg.
              C31B -C22B -C60B    2_656   1_555   1_555 ......     # 5237 Check
PLAT779_ALERT_4_G Suspect or Irrelevant (Bond) Angle(s) in CIF ...      10.00 Deg.
              C35B -C22B -C21B    2_656   1_555   1_555 ......     # 5242 Check
PLAT779_ALERT_4_G Suspect or Irrelevant (Bond) Angle(s) in CIF ...      13.10 Deg.
              C31B -C22B -C32B    2_656   1_555   2_656 ......     # 5252 Check
PLAT779_ALERT_4_G Suspect or Irrelevant (Bond) Angle(s) in CIF ...      43.50 Deg.
              C60B -C22B -C32B    1_555   1_555   2_656 ......     # 5256 Check
PLAT779_ALERT_4_G Suspect or Irrelevant (Bond) Angle(s) in CIF ...      32.00 Deg.
              C63B -C23B -C28B    2_656   1_555   1_555 ......     # 5273 Check
PLAT779_ALERT_4_G Suspect or Irrelevant (Bond) Angle(s) in CIF ...       8.60 Deg.
              C64B -C23B -C24B    2_656   1_555   1_555 ......     # 5278 Check
PLAT779_ALERT_4_G Suspect or Irrelevant (Bond) Angle(s) in CIF ...      13.20 Deg.
              C22B -C23B -C31B    1_555   1_555   2_656 ......     # 5291 Check
PLAT779_ALERT_4_G Suspect or Irrelevant (Bond) Angle(s) in CIF ...      39.20 Deg.
              C19B -C24B -C36B    1_555   1_555   2_656 ......     # 5300 Check
PLAT779_ALERT_4_G Suspect or Irrelevant (Bond) Angle(s) in CIF ...      32.00 Deg.
              C64B -C24B -C23B    2_656   1_555   1_555 ......     # 5301 Check
PLAT779_ALERT_4_G Suspect or Irrelevant (Bond) Angle(s) in CIF ...      16.10 Deg.
              C49B -C24B -C25B    2_656   1_555   1_555 ......     # 5306 Check
PLAT779_ALERT_4_G Suspect or Irrelevant (Bond) Angle(s) in CIF ...      42.00 Deg.
              C64B -C24B -C63B    2_656   1_555   2_656 ......     # 5310 Check
PLAT779_ALERT_4_G Suspect or Irrelevant (Bond) Angle(s) in CIF ...      27.30 Deg.
              C23B -C24B -C63B    1_555   1_555   2_656 ......     # 5314 Check
PLAT779_ALERT_4_G Suspect or Irrelevant (Bond) Angle(s) in CIF ...      43.90 Deg.
              ND3B -C24B -ND1B    2_656   1_555   2_656 ......     # 5330 Check
PLAT779_ALERT_4_G Suspect or Irrelevant (Bond) Angle(s) in CIF ...      12.40 Deg.
              C50B -C25B -C26B    2_656   1_555   1_555 ......     # 5333 Check
PLAT779_ALERT_4_G Suspect or Irrelevant (Bond) Angle(s) in CIF ...      36.30 Deg.
              C74B -C25B -C48B    1_555   1_555   2_656 ......     # 5344 Check
PLAT779_ALERT_4_G Suspect or Irrelevant (Bond) Angle(s) in CIF ...       9.80 Deg.
              C24B -C25B -C64B    1_555   1_555   2_656 ......     # 5350 Check
PLAT779_ALERT_4_G Suspect or Irrelevant (Bond) Angle(s) in CIF ...      41.10 Deg.
              C50B -C25B -C62B    2_656   1_555   2_656 ......     # 5353 Check
PLAT779_ALERT_4_G Suspect or Irrelevant (Bond) Angle(s) in CIF ...      33.90 Deg.
              C26B -C25B -C62B    1_555   1_555   2_656 ......     # 5354 Check
PLAT779_ALERT_4_G Suspect or Irrelevant (Bond) Angle(s) in CIF ...       9.21 Deg.
              ND3B -C25B -ND4B    2_656   1_555   1_555 ......     # 5375 Check
PLAT779_ALERT_4_G Suspect or Irrelevant (Bond) Angle(s) in CIF ...      25.10 Deg.
              C62B -C26B -C27B    2_656   1_555   1_555 ......     # 5380 Check
PLAT779_ALERT_4_G Suspect or Irrelevant (Bond) Angle(s) in CIF ...      24.30 Deg.
              C50B -C26B -C25B    2_656   1_555   1_555 ......     # 5382 Check
PLAT779_ALERT_4_G Suspect or Irrelevant (Bond) Angle(s) in CIF ...      14.70 Deg.
              C51B -C26B -C76B    2_656   1_555   1_555 ......     # 5388 Check
PLAT779_ALERT_4_G Suspect or Irrelevant (Bond) Angle(s) in CIF ...      25.40 Deg.
              C50B -C26B -C49B    2_656   1_555   2_656 ......     # 5391 Check
```

```
PLAT779_ALERT_4_G Suspect or Irrelevant (Bond) Angle(s) in CIF ...       5.30 Deg.
              C25B -C26B -C49B    1_555   1_555   2_656 ......     # 5395 Check
PLAT779_ALERT_4_G Suspect or Irrelevant (Bond) Angle(s) in CIF ...      21.20 Deg.
              C27B -C26B -C61B    1_555   1_555   2_656 ......     # 5400 Check
PLAT779_ALERT_4_G Suspect or Irrelevant (Bond) Angle(s) in CIF ...       8.65 Deg.
              ND3B -C26B -ND4B    2_656   1_555   1_555 ......     # 5420 Check
PLAT779_ALERT_4_G Suspect or Irrelevant (Bond) Angle(s) in CIF ...      10.70 Deg.
              C61B -C27B -C32B    2_656   1_555   1_555 ......     # 5428 Check
PLAT779_ALERT_4_G Suspect or Irrelevant (Bond) Angle(s) in CIF ...      29.50 Deg.
              C28B -C27B -C63B    1_555   1_555   2_656 ......     # 5434 Check
PLAT779_ALERT_4_G Suspect or Irrelevant (Bond) Angle(s) in CIF ...      40.40 Deg.
              C62B -C27B -C50B    2_656   1_555   2_656 ......     # 5436 Check
PLAT779_ALERT_4_G Suspect or Irrelevant (Bond) Angle(s) in CIF ...      11.10 Deg.
              C26B -C27B -C50B    1_555   1_555   2_656 ......     # 5438 Check
PLAT779_ALERT_4_G Suspect or Irrelevant (Bond) Angle(s) in CIF ...      43.20 Deg.
              C61B -C27B -C60B    2_656   1_555   2_656 ......     # 5443 Check
PLAT779_ALERT_4_G Suspect or Irrelevant (Bond) Angle(s) in CIF ...      37.20 Deg.
              C61B -C27B -C57B    2_656   1_555   2_656 ......     # 5450 Check
PLAT779_ALERT_4_G Suspect or Irrelevant (Bond) Angle(s) in CIF ...      31.00 Deg.
              C32B -C27B -C57B    1_555   1_555   2_656 ......     # 5453 Check
PLAT779_ALERT_4_G Suspect or Irrelevant (Bond) Angle(s) in CIF ...      27.80 Deg.
              C63B -C28B -C23B    2_656   1_555   1_555 ......     # 5472 Check
PLAT779_ALERT_4_G Suspect or Irrelevant (Bond) Angle(s) in CIF ...      25.10 Deg.
              C62B -C28B -C27B    2_656   1_555   1_555 ......     # 5478 Check
PLAT779_ALERT_4_G Suspect or Irrelevant (Bond) Angle(s) in CIF ...      13.00 Deg.
              C29B -C28B -C29B    1_555   1_555   2_656 ......     # 5484 Check
PLAT779_ALERT_4_G Suspect or Irrelevant (Bond) Angle(s) in CIF ...      23.00 Deg.
              C27B -C28B -C61B    1_555   1_555   2_656 ......     # 5492 Check
PLAT779_ALERT_4_G Suspect or Irrelevant (Bond) Angle(s) in CIF ...      43.60 Deg.
              C29B -C28B -C60B    2_656   1_555   2_656 ......     # 5500 Check
PLAT779_ALERT_4_G Suspect or Irrelevant (Bond) Angle(s) in CIF ...      42.00 Deg.
              C61B -C28B -C60B    2_656   1_555   2_656 ......     # 5501 Check
PLAT779_ALERT_4_G Suspect or Irrelevant (Bond) Angle(s) in CIF ...       9.60 Deg.
              C29B -C29B -C30B    2_656   1_555   1_555 ......     # 5508 Check
PLAT779_ALERT_4_G Suspect or Irrelevant (Bond) Angle(s) in CIF ...      41.10 Deg.
              C30B -C29B -C28B    1_555   1_555   2_656 ......     # 5516 Check
PLAT779_ALERT_4_G Suspect or Irrelevant (Bond) Angle(s) in CIF ...      40.70 Deg.
              C29B -C29B -C60B    2_656   1_555   2_656 ......     # 5517 Check
PLAT779_ALERT_4_G Suspect or Irrelevant (Bond) Angle(s) in CIF ...      42.80 Deg.
              C30B -C29B -C60B    1_555   1_555   2_656 ......     # 5521 Check
PLAT779_ALERT_4_G Suspect or Irrelevant (Bond) Angle(s) in CIF ...      37.60 Deg.
              C28B -C30B -C63B    2_656   1_555   1_555 ......     # 5534 Check
PLAT779_ALERT_4_G Suspect or Irrelevant (Bond) Angle(s) in CIF ...      31.80 Deg.
              C23B -C30B -C63B    2_656   1_555   1_555 ......     # 5536 Check
PLAT779_ALERT_4_G Suspect or Irrelevant (Bond) Angle(s) in CIF ...       4.10 Deg.
              C29B -C30B -C29B    2_656   1_555   1_555 ......     # 5538 Check
PLAT779_ALERT_4_G Suspect or Irrelevant (Bond) Angle(s) in CIF ...      39.40 Deg.
              C60B -C30B -C31B    2_656   1_555   1_555 ......     # 5546 Check
PLAT779_ALERT_4_G Suspect or Irrelevant (Bond) Angle(s) in CIF ...      26.50 Deg.
              C22B -C30B -C31B    2_656   1_555   1_555 ......     # 5548 Check
PLAT779_ALERT_4_G Suspect or Irrelevant (Bond) Angle(s) in CIF ...      11.90 Deg.
              C35B -C31B -C21B    1_555   1_555   2_656 ......     # 5564 Check
PLAT779_ALERT_4_G Suspect or Irrelevant (Bond) Angle(s) in CIF ...      19.20 Deg.
              C32B -C31B -C61B    1_555   1_555   2_656 ......     # 5568 Check
PLAT779_ALERT_4_G Suspect or Irrelevant (Bond) Angle(s) in CIF ...      28.50 Deg.
              C22B -C31B -C23B    2_656   1_555   2_656 ......     # 5572 Check
PLAT779_ALERT_4_G Suspect or Irrelevant (Bond) Angle(s) in CIF ...      44.20 Deg.
```

```
              C30B -C31B -C23B    1_555   1_555   2_656 ......     # 5576 Check
PLAT779_ALERT_4_G Suspect or Irrelevant (Bond) Angle(s) in CIF ...      39.20 Deg.
              C31B -C32B -C60B    1_555   1_555   2_656 ......     # 5592 Check
PLAT779_ALERT_4_G Suspect or Irrelevant (Bond) Angle(s) in CIF ...      25.40 Deg.
              C57B -C32B -C33B    2_656   1_555   1_555 ......     # 5594 Check
PLAT779_ALERT_4_G Suspect or Irrelevant (Bond) Angle(s) in CIF ...      13.50 Deg.
              C61B -C32B -C27B    2_656   1_555   1_555 ......     # 5597 Check
PLAT779_ALERT_4_G Suspect or Irrelevant (Bond) Angle(s) in CIF ...      25.10 Deg.
              C33B -C32B -C58B    1_555   1_555   2_656 ......     # 5606 Check
PLAT779_ALERT_4_G Suspect or Irrelevant (Bond) Angle(s) in CIF ...       6.20 Deg.
              C31B -C32B -C22B    1_555   1_555   2_656 ......     # 5610 Check
PLAT779_ALERT_4_G Suspect or Irrelevant (Bond) Angle(s) in CIF ...      44.30 Deg.
              C60B -C32B -C22B    2_656   1_555   2_656 ......     # 5611 Check
PLAT779_ALERT_4_G Suspect or Irrelevant (Bond) Angle(s) in CIF ...      42.80 Deg.
              C57B -C33B -C32B    2_656   1_555   1_555 ......     # 5618 Check
PLAT779_ALERT_4_G Suspect or Irrelevant (Bond) Angle(s) in CIF ...      12.40 Deg.
              C58B -C33B -C34B    2_656   1_555   1_555 ......     # 5622 Check
PLAT779_ALERT_4_G Suspect or Irrelevant (Bond) Angle(s) in CIF ...      19.80 Deg.
              C77B -C33B -C56B    1_555   1_555   2_656 ......     # 5627 Check
PLAT779_ALERT_4_G Suspect or Irrelevant (Bond) Angle(s) in CIF ...      27.50 Deg.
              C77B -C33B -C55B    1_555   1_555   2_656 ......     # 5638 Check
PLAT779_ALERT_4_G Suspect or Irrelevant (Bond) Angle(s) in CIF ...      29.80 Deg.
              C57B -C33B -C61B    2_656   1_555   2_656 ......     # 5643 Check
PLAT779_ALERT_4_G Suspect or Irrelevant (Bond) Angle(s) in CIF ...      15.00 Deg.
              C32B -C33B -C61B    1_555   1_555   2_656 ......     # 5646 Check
PLAT779_ALERT_4_G Suspect or Irrelevant (Bond) Angle(s) in CIF ...      15.30 Deg.
              C58B -C34B -C33B    2_656   1_555   1_555 ......     # 5666 Check
PLAT779_ALERT_4_G Suspect or Irrelevant (Bond) Angle(s) in CIF ...       0.20 Deg.
              C21B -C34B -C35B    2_656   1_555   1_555 ......     # 5671 Check
PLAT779_ALERT_4_G Suspect or Irrelevant (Bond) Angle(s) in CIF ...      12.61 Deg.
              ND1B -C34B -ND2B    1_555   1_555   2_656 ......     # 5695 Check
PLAT779_ALERT_4_G Suspect or Irrelevant (Bond) Angle(s) in CIF ...      26.90 Deg.
              C22B -C35B -C31B    2_656   1_555   1_555 ......     # 5698 Check
PLAT779_ALERT_4_G Suspect or Irrelevant (Bond) Angle(s) in CIF ...       0.00 Deg.
              C21B -C35B -C34B    2_656   1_555   1_555 ......     # 5699 Check
PLAT779_ALERT_4_G Suspect or Irrelevant (Bond) Angle(s) in CIF ...      44.00 Deg.
              C36B -C35B -C20B    1_555   1_555   2_656 ......     # 5710 Check
PLAT779_ALERT_4_G Suspect or Irrelevant (Bond) Angle(s) in CIF ...      22.00 Deg.
              C21B -C35B -C58B    2_656   1_555   2_656 ......     # 5711 Check
PLAT779_ALERT_4_G Suspect or Irrelevant (Bond) Angle(s) in CIF ...      21.70 Deg.
              C34B -C35B -C58B    1_555   1_555   2_656 ......     # 5714 Check
PLAT779_ALERT_4_G Suspect or Irrelevant (Bond) Angle(s) in CIF ...       9.33 Deg.
              ND1B -C35B -ND2B    1_555   1_555   2_656 ......     # 5731 Check
PLAT779_ALERT_4_G Suspect or Irrelevant (Bond) Angle(s) in CIF ...      13.10 Deg.
              C24B -C36B -C64B    2_656   1_555   1_555 ......     # 5737 Check
PLAT779_ALERT_4_G Suspect or Irrelevant (Bond) Angle(s) in CIF ...      38.00 Deg.
              C19B -C36B -C37B    2_656   1_555   1_555 ......     # 5742 Check
PLAT779_ALERT_4_G Suspect or Irrelevant (Bond) Angle(s) in CIF ...      10.00 Deg.
              C35B -C36B -C21B    1_555   1_555   2_656 ......     # 5751 Check
PLAT779_ALERT_4_G Suspect or Irrelevant (Bond) Angle(s) in CIF ...      39.10 Deg.
              C64B -C36B -C23B    1_555   1_555   2_656 ......     # 5756 Check
PLAT779_ALERT_4_G Suspect or Irrelevant (Bond) Angle(s) in CIF ...      36.90 Deg.
              C35B -C36B -C22B    1_555   1_555   2_656 ......     # 5764 Check
PLAT779_ALERT_4_G Suspect or Irrelevant (Bond) Angle(s) in CIF ...      42.50 Deg.
              C23B -C36B -C22B    2_656   1_555   2_656 ......     # 5767 Check
PLAT779_ALERT_4_G Suspect or Irrelevant (Bond) Angle(s) in CIF ...       8.83 Deg.
              ND1B -C36B -ND2B    1_555   1_555   2_656 ......     # 5786 Check
```

```
PLAT779_ALERT_4_G Suspect or Irrelevant (Bond) Angle(s) in CIF ...       7.80 Deg.
               C18B -C37B -C65B    2_656   1_555   1_555 ......     # 5792 Check
PLAT779_ALERT_4_G Suspect or Irrelevant (Bond) Angle(s) in CIF ...      38.30 Deg.
               C19B -C37B -C36B    2_656   1_555   1_555 ......     # 5802 Check
PLAT779_ALERT_4_G Suspect or Irrelevant (Bond) Angle(s) in CIF ...      12.80 Deg.
               C38B -C37B -C16B    1_555   1_555   2_656 ......     # 5812 Check
PLAT779_ALERT_4_G Suspect or Irrelevant (Bond) Angle(s) in CIF ...      12.64 Deg.
               ND1B -C37B -ND2B    1_555   1_555   2_656 ......     # 5831 Check
PLAT779_ALERT_4_G Suspect or Irrelevant (Bond) Angle(s) in CIF ...      42.00 Deg.
               C17B -C38B -C43B    2_656   1_555   1_555 ......     # 5834 Check
PLAT779_ALERT_4_G Suspect or Irrelevant (Bond) Angle(s) in CIF ...      26.20 Deg.
               C39B -C38B -C15B    1_555   1_555   2_656 ......     # 5845 Check
PLAT779_ALERT_4_G Suspect or Irrelevant (Bond) Angle(s) in CIF ...      42.70 Deg.
               C37B -C38B -C20B    1_555   1_555   2_656 ......     # 5851 Check
PLAT779_ALERT_4_G Suspect or Irrelevant (Bond) Angle(s) in CIF ...      13.27 Deg.
               ND2B -C38B -ND1B    2_656   1_555   1_555 ......     # 5867 Check
PLAT779_ALERT_4_G Suspect or Irrelevant (Bond) Angle(s) in CIF ...      11.50 Deg.
               C16B -C39B -C38B    2_656   1_555   1_555 ......     # 5872 Check
PLAT779_ALERT_4_G Suspect or Irrelevant (Bond) Angle(s) in CIF ...      40.20 Deg.
               C15B -C39B -C40B    2_656   1_555   1_555 ......     # 5878 Check
PLAT779_ALERT_4_G Suspect or Irrelevant (Bond) Angle(s) in CIF ...      14.60 Deg.
               C34B -C39B -C58B    1_555   1_555   2_656 ......     # 5893 Check
PLAT779_ALERT_4_G Suspect or Irrelevant (Bond) Angle(s) in CIF ...      12.92 Deg.
               ND2B -C39B -ND1B    2_656   1_555   1_555 ......     # 5912 Check
PLAT779_ALERT_4_G Suspect or Irrelevant (Bond) Angle(s) in CIF ...      24.00 Deg.
               C14B -C40B -C41B    2_656   1_555   1_555 ......     # 5917 Check
PLAT779_ALERT_4_G Suspect or Irrelevant (Bond) Angle(s) in CIF ...      25.20 Deg.
               C15B -C40B -C39B    2_656   1_555   1_555 ......     # 5919 Check
PLAT779_ALERT_4_G Suspect or Irrelevant (Bond) Angle(s) in CIF ...      31.90 Deg.
               C41B -C40B -C80B    1_555   1_555   2_656 ......     # 5931 Check
PLAT779_ALERT_4_G Suspect or Irrelevant (Bond) Angle(s) in CIF ...      17.70 Deg.
               C78B -C40B -C55B    1_555   1_555   2_656 ......     # 5939 Check
PLAT779_ALERT_4_G Suspect or Irrelevant (Bond) Angle(s) in CIF ...      28.30 Deg.
               C78B -C40B -C54B    1_555   1_555   2_656 ......     # 5946 Check
PLAT779_ALERT_4_G Suspect or Irrelevant (Bond) Angle(s) in CIF ...       8.56 Deg.
               ND2B -C40B -ND1B    2_656   1_555   1_555 ......     # 5967 Check
PLAT779_ALERT_4_G Suspect or Irrelevant (Bond) Angle(s) in CIF ...      23.90 Deg.
               C80B -C41B -C67B    2_656   1_555   1_555 ......     # 5972 Check
PLAT779_ALERT_4_G Suspect or Irrelevant (Bond) Angle(s) in CIF ...       8.90 Deg.
               C42B -C41B -C13B    1_555   1_555   2_656 ......     # 5982 Check
PLAT779_ALERT_4_G Suspect or Irrelevant (Bond) Angle(s) in CIF ...      29.30 Deg.
               C67B -C41B -C7B     1_555   1_555   2_656 ......     # 5986 Check
PLAT779_ALERT_4_G Suspect or Irrelevant (Bond) Angle(s) in CIF ...      32.60 Deg.
               C14B -C41B -C15B    2_656   1_555   2_656 ......     # 5989 Check
PLAT779_ALERT_4_G Suspect or Irrelevant (Bond) Angle(s) in CIF ...      32.50 Deg.
               C40B -C41B -C15B    1_555   1_555   2_656 ......     # 5991 Check
PLAT779_ALERT_4_G Suspect or Irrelevant (Bond) Angle(s) in CIF ...      43.50 Deg.
               C7B  -C41B -C8B     2_656   1_555   2_656 ......     # 6002 Check
PLAT779_ALERT_4_G Suspect or Irrelevant (Bond) Angle(s) in CIF ...      34.20 Deg.
               C12B -C42B -C43B    2_656   1_555   1_555 ......     # 6018 Check
PLAT779_ALERT_4_G Suspect or Irrelevant (Bond) Angle(s) in CIF ...      23.10 Deg.
               C14B -C42B -C41B    2_656   1_555   1_555 ......     # 6024 Check
PLAT779_ALERT_4_G Suspect or Irrelevant (Bond) Angle(s) in CIF ...      34.30 Deg.
               C3B  -C42B -C8B     1_555   1_555   2_656 ......     # 6032 Check
PLAT779_ALERT_4_G Suspect or Irrelevant (Bond) Angle(s) in CIF ...       7.60 Deg.
               C42B -C43B -C13B    1_555   1_555   2_656 ......     # 6054 Check
PLAT779_ALERT_4_G Suspect or Irrelevant (Bond) Angle(s) in CIF ...       3.10 Deg.
```

```
             C38B -C43B -C16B    1_555   1_555   2_656 ......     # 6058 Check
PLAT779_ALERT_4_G Suspect or Irrelevant (Bond) Angle(s) in CIF ...       9.97 Deg.
             ND2B -C43B -ND1B    2_656   1_555   1_555 ......     # 6076 Check
PLAT779_ALERT_4_G Suspect or Irrelevant (Bond) Angle(s) in CIF ...      35.20 Deg.
             C12B -C44B -C43B    2_656   1_555   1_555 ......     # 6083 Check
PLAT779_ALERT_4_G Suspect or Irrelevant (Bond) Angle(s) in CIF ...      41.60 Deg.
             C17B -C44B -C43B    2_656   1_555   1_555 ......     # 6084 Check
PLAT779_ALERT_4_G Suspect or Irrelevant (Bond) Angle(s) in CIF ...      21.00 Deg.
             C45B -C44B -C11B    1_555   1_555   2_656 ......     # 6089 Check
PLAT779_ALERT_4_G Suspect or Irrelevant (Bond) Angle(s) in CIF ...       8.10 Deg.
             C65B -C44B -C18B    1_555   1_555   2_656 ......     # 6095 Check
PLAT779_ALERT_4_G Suspect or Irrelevant (Bond) Angle(s) in CIF ...      28.90 Deg.
             C45B -C44B -C72B    1_555   1_555   2_656 ......     # 6100 Check
PLAT779_ALERT_4_G Suspect or Irrelevant (Bond) Angle(s) in CIF ...      10.80 Deg.
             ND2B -C44B -ND1B    2_656   1_555   1_555 ......     # 6131 Check
PLAT779_ALERT_4_G Suspect or Irrelevant (Bond) Angle(s) in CIF ...      29.00 Deg.
             C11B -C45B -C4B     2_656   1_555   1_555 ......     # 6135 Check
PLAT779_ALERT_4_G Suspect or Irrelevant (Bond) Angle(s) in CIF ...      18.20 Deg.
             C72B -C45B -C46B    2_656   1_555   1_555 ......     # 6139 Check
PLAT779_ALERT_4_G Suspect or Irrelevant (Bond) Angle(s) in CIF ...      43.20 Deg.
             C11B -C45B -C10B    2_656   1_555   2_656 ......     # 6142 Check
PLAT779_ALERT_4_G Suspect or Irrelevant (Bond) Angle(s) in CIF ...      14.30 Deg.
             C4B  -C45B -C10B    1_555   1_555   2_656 ......     # 6145 Check
PLAT779_ALERT_4_G Suspect or Irrelevant (Bond) Angle(s) in CIF ...      40.70 Deg.
             C44B -C45B -C12B    1_555   1_555   2_656 ......     # 6149 Check
PLAT779_ALERT_4_G Suspect or Irrelevant (Bond) Angle(s) in CIF ...      42.60 Deg.
             C72B -C45B -C70B    2_656   1_555   2_656 ......     # 6154 Check
PLAT779_ALERT_4_G Suspect or Irrelevant (Bond) Angle(s) in CIF ...      24.80 Deg.
             C46B -C45B -C70B    1_555   1_555   2_656 ......     # 6157 Check
PLAT779_ALERT_4_G Suspect or Irrelevant (Bond) Angle(s) in CIF ...      40.00 Deg.
             C72B -C45B -C73B    2_656   1_555   2_656 ......     # 6161 Check
PLAT779_ALERT_4_G Suspect or Irrelevant (Bond) Angle(s) in CIF ...      12.10 Deg.
             C70B -C46B -C79B    2_656   1_555   1_555 ......     # 6179 Check
PLAT779_ALERT_4_G Suspect or Irrelevant (Bond) Angle(s) in CIF ...      22.80 Deg.
             C72B -C46B -C45B    2_656   1_555   1_555 ......     # 6183 Check
PLAT779_ALERT_4_G Suspect or Irrelevant (Bond) Angle(s) in CIF ...      44.60 Deg.
             C47B -C46B -C73B    1_555   1_555   2_656 ......     # 6190 Check
PLAT779_ALERT_4_G Suspect or Irrelevant (Bond) Angle(s) in CIF ...      39.40 Deg.
             C79B -C46B -C69B    1_555   1_555   2_656 ......     # 6194 Check
PLAT779_ALERT_4_G Suspect or Irrelevant (Bond) Angle(s) in CIF ...      31.30 Deg.
             C72B -C46B -C11B    2_656   1_555   2_656 ......     # 6198 Check
PLAT779_ALERT_4_G Suspect or Irrelevant (Bond) Angle(s) in CIF ...       9.00 Deg.
             C45B -C46B -C11B    1_555   1_555   2_656 ......     # 6202 Check
PLAT779_ALERT_4_G Suspect or Irrelevant (Bond) Angle(s) in CIF ...      30.90 Deg.
             C70B -C46B -C71B    2_656   1_555   2_656 ......     # 6206 Check
PLAT779_ALERT_4_G Suspect or Irrelevant (Bond) Angle(s) in CIF ...      43.00 Deg.
             C79B -C46B -C71B    1_555   1_555   2_656 ......     # 6207 Check
PLAT779_ALERT_4_G Suspect or Irrelevant (Bond) Angle(s) in CIF ...       8.66 Deg.
             ND4B -C46B -ND3B    2_656   1_555   1_555 ......     # 6231 Check
PLAT779_ALERT_4_G Suspect or Irrelevant (Bond) Angle(s) in CIF ...      42.30 Deg.
             C74B -C47B -C48B    2_656   1_555   1_555 ......     # 6238 Check
PLAT779_ALERT_4_G Suspect or Irrelevant (Bond) Angle(s) in CIF ...      39.00 Deg.
             C73B -C47B -C48B    2_656   1_555   1_555 ......     # 6239 Check
PLAT779_ALERT_4_G Suspect or Irrelevant (Bond) Angle(s) in CIF ...      10.70 Deg.
             C52B -C47B -C75B    1_555   1_555   2_656 ......     # 6244 Check
PLAT779_ALERT_4_G Suspect or Irrelevant (Bond) Angle(s) in CIF ...      24.20 Deg.
             C46B -C47B -C72B    1_555   1_555   2_656 ......     # 6250 Check
```

```
PLAT779_ALERT_4_G Suspect or Irrelevant (Bond) Angle(s) in CIF ...      42.40 Deg.
              C52B -C47B -C69B    1_555   1_555   2_656 ......      # 6255 Check
PLAT779_ALERT_4_G Suspect or Irrelevant (Bond) Angle(s) in CIF ...      21.60 Deg.
              C46B -C47B -C70B    1_555   1_555   2_656 ......      # 6263 Check
PLAT779_ALERT_4_G Suspect or Irrelevant (Bond) Angle(s) in CIF ...      43.80 Deg.
              C72B -C47B -C70B    2_656   1_555   2_656 ......      # 6266 Check
PLAT779_ALERT_4_G Suspect or Irrelevant (Bond) Angle(s) in CIF ...      43.60 Deg.
              C69B -C47B -C70B    2_656   1_555   2_656 ......      # 6267 Check
PLAT779_ALERT_4_G Suspect or Irrelevant (Bond) Angle(s) in CIF ...      12.78 Deg.
              ND4B -C47B -ND3B    2_656   1_555   1_555 ......      # 6286 Check
PLAT779_ALERT_4_G Suspect or Irrelevant (Bond) Angle(s) in CIF ...      41.90 Deg.
              C74B -C48B -C47B    2_656   1_555   1_555 ......      # 6291 Check
PLAT779_ALERT_4_G Suspect or Irrelevant (Bond) Angle(s) in CIF ...       9.50 Deg.
              C18B -C48B -C65B    2_656   1_555   1_555 ......      # 6301 Check
PLAT779_ALERT_4_G Suspect or Irrelevant (Bond) Angle(s) in CIF ...      16.00 Deg.
              C49B -C48B -C25B    1_555   1_555   2_656 ......      # 6304 Check
PLAT779_ALERT_4_G Suspect or Irrelevant (Bond) Angle(s) in CIF ...      12.41 Deg.
              ND3B -C48B -ND4B    1_555   1_555   2_656 ......      # 6322 Check
PLAT779_ALERT_4_G Suspect or Irrelevant (Bond) Angle(s) in CIF ...      10.70 Deg.
              C24B -C49B -C64B    2_656   1_555   1_555 ......      # 6325 Check
PLAT779_ALERT_4_G Suspect or Irrelevant (Bond) Angle(s) in CIF ...      16.00 Deg.
              C25B -C49B -C50B    2_656   1_555   1_555 ......      # 6326 Check
PLAT779_ALERT_4_G Suspect or Irrelevant (Bond) Angle(s) in CIF ...      38.00 Deg.
              C48B -C49B -C74B    1_555   1_555   2_656 ......      # 6337 Check
PLAT779_ALERT_4_G Suspect or Irrelevant (Bond) Angle(s) in CIF ...      16.30 Deg.
              C25B -C49B -C26B    2_656   1_555   2_656 ......      # 6338 Check
PLAT779_ALERT_4_G Suspect or Irrelevant (Bond) Angle(s) in CIF ...       8.90 Deg.
              C50B -C49B -C26B    1_555   1_555   2_656 ......      # 6341 Check
PLAT779_ALERT_4_G Suspect or Irrelevant (Bond) Angle(s) in CIF ...       8.54 Deg.
              ND3B -C49B -ND4B    1_555   1_555   2_656 ......      # 6358 Check
PLAT779_ALERT_4_G Suspect or Irrelevant (Bond) Angle(s) in CIF ...       7.50 Deg.
              C25B -C50B -C49B    2_656   1_555   1_555 ......      # 6363 Check
PLAT779_ALERT_4_G Suspect or Irrelevant (Bond) Angle(s) in CIF ...      11.50 Deg.
              C51B -C50B -C76B    1_555   1_555   2_656 ......      # 6373 Check
PLAT779_ALERT_4_G Suspect or Irrelevant (Bond) Angle(s) in CIF ...      32.20 Deg.
              C26B -C50B -C27B    2_656   1_555   2_656 ......      # 6374 Check
PLAT779_ALERT_4_G Suspect or Irrelevant (Bond) Angle(s) in CIF ...      16.50 Deg.
              C62B -C50B -C27B    1_555   1_555   2_656 ......      # 6376 Check
PLAT779_ALERT_4_G Suspect or Irrelevant (Bond) Angle(s) in CIF ...       8.72 Deg.
              ND3B -C50B -ND4B    1_555   1_555   2_656 ......      # 6403 Check
PLAT779_ALERT_4_G Suspect or Irrelevant (Bond) Angle(s) in CIF ...       2.00 Deg.
              C76B -C51B -C56B    2_656   1_555   1_555 ......      # 6407 Check
PLAT779_ALERT_4_G Suspect or Irrelevant (Bond) Angle(s) in CIF ...      20.70 Deg.
              C26B -C51B -C50B    2_656   1_555   1_555 ......      # 6412 Check
PLAT779_ALERT_4_G Suspect or Irrelevant (Bond) Angle(s) in CIF ...       4.20 Deg.
              C75B -C51B -C52B    2_656   1_555   1_555 ......      # 6415 Check
PLAT779_ALERT_4_G Suspect or Irrelevant (Bond) Angle(s) in CIF ...      12.00 Deg.
              C76B -C51B -C77B    2_656   1_555   2_656 ......      # 6419 Check
PLAT779_ALERT_4_G Suspect or Irrelevant (Bond) Angle(s) in CIF ...      13.30 Deg.
              C56B -C51B -C77B    1_555   1_555   2_656 ......      # 6422 Check
PLAT779_ALERT_4_G Suspect or Irrelevant (Bond) Angle(s) in CIF ...      12.26 Deg.
              ND3B -C51B -ND4B    1_555   1_555   2_656 ......      # 6448 Check
PLAT779_ALERT_4_G Suspect or Irrelevant (Bond) Angle(s) in CIF ...      19.60 Deg.
              C69B -C52B -C53B    2_656   1_555   1_555 ......      # 6453 Check
PLAT779_ALERT_4_G Suspect or Irrelevant (Bond) Angle(s) in CIF ...      18.00 Deg.
              C75B -C52B -C51B    2_656   1_555   1_555 ......      # 6455 Check
PLAT779_ALERT_4_G Suspect or Irrelevant (Bond) Angle(s) in CIF ...      37.70 Deg.
```

```
              C47B -C52B -C74B    1_555   1_555   2_656 ......     # 6461 Check
PLAT779_ALERT_4_G Suspect or Irrelevant (Bond) Angle(s) in CIF ...      28.00 Deg.
              C75B -C52B -C76B    2_656   1_555   2_656 ......     # 6464 Check
PLAT779_ALERT_4_G Suspect or Irrelevant (Bond) Angle(s) in CIF ...      12.10 Deg.
              C51B -C52B -C76B    1_555   1_555   2_656 ......     # 6468 Check
PLAT779_ALERT_4_G Suspect or Irrelevant (Bond) Angle(s) in CIF ...      12.47 Deg.
              ND4B -C52B -ND3B    2_656   1_555   1_555 ......     # 6484 Check
PLAT779_ALERT_4_G Suspect or Irrelevant (Bond) Angle(s) in CIF ...      20.80 Deg.
              C68B -C53B -C54B    2_656   1_555   1_555 ......     # 6492 Check
PLAT779_ALERT_4_G Suspect or Irrelevant (Bond) Angle(s) in CIF ...       7.80 Deg.
              C52B -C53B -C75B    1_555   1_555   2_656 ......     # 6497 Check
PLAT779_ALERT_4_G Suspect or Irrelevant (Bond) Angle(s) in CIF ...      30.30 Deg.
              C69B -C53B -C70B    2_656   1_555   2_656 ......     # 6500 Check
PLAT779_ALERT_4_G Suspect or Irrelevant (Bond) Angle(s) in CIF ...      16.20 Deg.
              C79B -C53B -C70B    1_555   1_555   2_656 ......     # 6503 Check
PLAT779_ALERT_4_G Suspect or Irrelevant (Bond) Angle(s) in CIF ...       8.24 Deg.
              ND4B -C53B -ND3B    2_656   1_555   1_555 ......     # 6520 Check
PLAT779_ALERT_4_G Suspect or Irrelevant (Bond) Angle(s) in CIF ...       8.90 Deg.
              C78B -C54B -C55B    2_656   1_555   1_555 ......     # 6523 Check
PLAT779_ALERT_4_G Suspect or Irrelevant (Bond) Angle(s) in CIF ...      37.30 Deg.
              C68B -C54B -C53B    2_656   1_555   1_555 ......     # 6527 Check
PLAT779_ALERT_4_G Suspect or Irrelevant (Bond) Angle(s) in CIF ...      26.50 Deg.
              C80B -C54B -C67B    1_555   1_555   2_656 ......     # 6534 Check
PLAT779_ALERT_4_G Suspect or Irrelevant (Bond) Angle(s) in CIF ...      34.90 Deg.
              C68B -C54B -C69B    2_656   1_555   2_656 ......     # 6536 Check
PLAT779_ALERT_4_G Suspect or Irrelevant (Bond) Angle(s) in CIF ...      10.00 Deg.
              C53B -C54B -C69B    1_555   1_555   2_656 ......     # 6540 Check
PLAT779_ALERT_4_G Suspect or Irrelevant (Bond) Angle(s) in CIF ...      15.50 Deg.
              C78B -C55B -C54B    2_656   1_555   1_555 ......     # 6552 Check
PLAT779_ALERT_4_G Suspect or Irrelevant (Bond) Angle(s) in CIF ...      11.60 Deg.
              C77B -C55B -C56B    2_656   1_555   1_555 ......     # 6556 Check
PLAT779_ALERT_4_G Suspect or Irrelevant (Bond) Angle(s) in CIF ...      41.60 Deg.
              C77B -C55B -C33B    2_656   1_555   2_656 ......     # 6565 Check
PLAT779_ALERT_4_G Suspect or Irrelevant (Bond) Angle(s) in CIF ...      21.60 Deg.
              C78B -C55B -C68B    2_656   1_555   2_656 ......     # 6570 Check
PLAT779_ALERT_4_G Suspect or Irrelevant (Bond) Angle(s) in CIF ...       8.80 Deg.
              C54B -C55B -C68B    1_555   1_555   2_656 ......     # 6573 Check
PLAT779_ALERT_4_G Suspect or Irrelevant (Bond) Angle(s) in CIF ...      21.70 Deg.
              C77B -C56B -C55B    2_656   1_555   1_555 ......     # 6580 Check
PLAT779_ALERT_4_G Suspect or Irrelevant (Bond) Angle(s) in CIF ...       0.70 Deg.
              C76B -C56B -C51B    2_656   1_555   1_555 ......     # 6584 Check
PLAT779_ALERT_4_G Suspect or Irrelevant (Bond) Angle(s) in CIF ...      25.60 Deg.
              C57B -C56B -C33B    1_555   1_555   2_656 ......     # 6589 Check
PLAT779_ALERT_4_G Suspect or Irrelevant (Bond) Angle(s) in CIF ...      27.10 Deg.
              C77B -C56B -C78B    2_656   1_555   2_656 ......     # 6592 Check
PLAT779_ALERT_4_G Suspect or Irrelevant (Bond) Angle(s) in CIF ...      12.10 Deg.
              C55B -C56B -C78B    1_555   1_555   2_656 ......     # 6595 Check
PLAT779_ALERT_4_G Suspect or Irrelevant (Bond) Angle(s) in CIF ...      19.20 Deg.
              C33B -C57B -C58B    2_656   1_555   1_555 ......     # 6608 Check
PLAT779_ALERT_4_G Suspect or Irrelevant (Bond) Angle(s) in CIF ...      27.00 Deg.
              C32B -C57B -C61B    2_656   1_555   1_555 ......     # 6612 Check
PLAT779_ALERT_4_G Suspect or Irrelevant (Bond) Angle(s) in CIF ...      20.70 Deg.
              C56B -C57B -C77B    1_555   1_555   2_656 ......     # 6617 Check
PLAT779_ALERT_4_G Suspect or Irrelevant (Bond) Angle(s) in CIF ...      21.20 Deg.
              C61B -C57B -C27B    1_555   1_555   2_656 ......     # 6624 Check
PLAT779_ALERT_4_G Suspect or Irrelevant (Bond) Angle(s) in CIF ...      15.50 Deg.
              C33B -C58B -C57B    2_656   1_555   1_555 ......     # 6630 Check
```

```
PLAT779_ALERT_4_G Suspect or Irrelevant (Bond) Angle(s) in CIF ...       5.10 Deg.
              C21B -C58B -C35B    1_555   1_555   2_656 ......     # 6640 Check
PLAT779_ALERT_4_G Suspect or Irrelevant (Bond) Angle(s) in CIF ...      34.50 Deg.
              C57B -C58B -C32B    1_555   1_555   2_656 ......     # 6644 Check
PLAT779_ALERT_4_G Suspect or Irrelevant (Bond) Angle(s) in CIF ...      33.10 Deg.
              C34B -C58B -C39B    2_656   1_555   2_656 ......     # 6647 Check
PLAT779_ALERT_4_G Suspect or Irrelevant (Bond) Angle(s) in CIF ...      40.50 Deg.
              C59B -C58B -C39B    1_555   1_555   2_656 ......     # 6649 Check
PLAT779_ALERT_4_G Suspect or Irrelevant (Bond) Angle(s) in CIF ...      32.10 Deg.
              C33B -C58B -C77B    2_656   1_555   2_656 ......     # 6655 Check
PLAT779_ALERT_4_G Suspect or Irrelevant (Bond) Angle(s) in CIF ...      11.21 Deg.
              ND1B -C58B -ND2B    2_656   1_555   1_555 ......     # 6680 Check
PLAT779_ALERT_4_G Suspect or Irrelevant (Bond) Angle(s) in CIF ...      27.80 Deg.
              C34B -C59B -C58B    2_656   1_555   1_555 ......     # 6686 Check
PLAT779_ALERT_4_G Suspect or Irrelevant (Bond) Angle(s) in CIF ...      27.60 Deg.
              C39B -C59B -C15B    2_656   1_555   1_555 ......     # 6691 Check
PLAT779_ALERT_4_G Suspect or Irrelevant (Bond) Angle(s) in CIF ...      43.60 Deg.
              C40B -C59B -C15B    2_656   1_555   1_555 ......     # 6692 Check
PLAT779_ALERT_4_G Suspect or Irrelevant (Bond) Angle(s) in CIF ...      18.20 Deg.
              C55B -C59B -C78B    1_555   1_555   2_656 ......     # 6700 Check
PLAT779_ALERT_4_G Suspect or Irrelevant (Bond) Angle(s) in CIF ...      29.40 Deg.
              C58B -C59B -C33B    1_555   1_555   2_656 ......     # 6705 Check
PLAT779_ALERT_4_G Suspect or Irrelevant (Bond) Angle(s) in CIF ...      31.50 Deg.
              C55B -C59B -C77B    1_555   1_555   2_656 ......     # 6713 Check
PLAT779_ALERT_4_G Suspect or Irrelevant (Bond) Angle(s) in CIF ...      11.05 Deg.
              ND2B -C59B -ND1B    1_555   1_555   2_656 ......     # 6735 Check
PLAT779_ALERT_4_G Suspect or Irrelevant (Bond) Angle(s) in CIF ...      44.90 Deg.
              C30B -C60B -C29B    2_656   1_555   1_555 ......     # 6740 Check
PLAT779_ALERT_4_G Suspect or Irrelevant (Bond) Angle(s) in CIF ...      22.70 Deg.
              C31B -C60B -C22B    2_656   1_555   1_555 ......     # 6742 Check
PLAT779_ALERT_4_G Suspect or Irrelevant (Bond) Angle(s) in CIF ...      27.60 Deg.
              C32B -C60B -C61B    2_656   1_555   1_555 ......     # 6748 Check
PLAT779_ALERT_4_G Suspect or Irrelevant (Bond) Angle(s) in CIF ...      11.70 Deg.
              C29B -C60B -C29B    1_555   1_555   2_656 ......     # 6754 Check
PLAT779_ALERT_4_G Suspect or Irrelevant (Bond) Angle(s) in CIF ...      23.90 Deg.
              C61B -C60B -C27B    1_555   1_555   2_656 ......     # 6762 Check
PLAT779_ALERT_4_G Suspect or Irrelevant (Bond) Angle(s) in CIF ...      43.10 Deg.
              C29B -C60B -C28B    2_656   1_555   2_656 ......     # 6770 Check
PLAT779_ALERT_4_G Suspect or Irrelevant (Bond) Angle(s) in CIF ...      42.40 Deg.
              C27B -C60B -C28B    2_656   1_555   2_656 ......     # 6771 Check
PLAT779_ALERT_4_G Suspect or Irrelevant (Bond) Angle(s) in CIF ...      44.90 Deg.
              C32B -C61B -C57B    2_656   1_555   1_555 ......     # 6773 Check
PLAT779_ALERT_4_G Suspect or Irrelevant (Bond) Angle(s) in CIF ...       4.30 Deg.
              C27B -C61B -C62B    2_656   1_555   1_555 ......     # 6779 Check
PLAT779_ALERT_4_G Suspect or Irrelevant (Bond) Angle(s) in CIF ...      42.70 Deg.
              C32B -C61B -C31B    2_656   1_555   2_656 ......     # 6782 Check
PLAT779_ALERT_4_G Suspect or Irrelevant (Bond) Angle(s) in CIF ...      30.80 Deg.
              C60B -C61B -C31B    1_555   1_555   2_656 ......     # 6785 Check
PLAT779_ALERT_4_G Suspect or Irrelevant (Bond) Angle(s) in CIF ...      33.30 Deg.
              C32B -C61B -C33B    2_656   1_555   2_656 ......     # 6787 Check
PLAT779_ALERT_4_G Suspect or Irrelevant (Bond) Angle(s) in CIF ...      13.50 Deg.
              C57B -C61B -C33B    1_555   1_555   2_656 ......     # 6789 Check
PLAT779_ALERT_4_G Suspect or Irrelevant (Bond) Angle(s) in CIF ...      41.40 Deg.
              C27B -C61B -C28B    2_656   1_555   2_656 ......     # 6794 Check
PLAT779_ALERT_4_G Suspect or Irrelevant (Bond) Angle(s) in CIF ...      36.90 Deg.
              C27B -C61B -C26B    2_656   1_555   2_656 ......     # 6801 Check
PLAT779_ALERT_4_G Suspect or Irrelevant (Bond) Angle(s) in CIF ...      34.00 Deg.
```

```
              C62B -C61B -C26B    1_555   1_555   2_656 ......     # 6804 Check
PLAT779_ALERT_4_G Suspect or Irrelevant (Bond) Angle(s) in CIF ...      36.30 Deg.
              C28B -C62B -C63B    2_656   1_555   1_555 ......     # 6813 Check
PLAT779_ALERT_4_G Suspect or Irrelevant (Bond) Angle(s) in CIF ...      19.80 Deg.
              C26B -C62B -C50B    2_656   1_555   1_555 ......     # 6815 Check
PLAT779_ALERT_4_G Suspect or Irrelevant (Bond) Angle(s) in CIF ...       6.00 Deg.
              C27B -C62B -C61B    2_656   1_555   1_555 ......     # 6818 Check
PLAT779_ALERT_4_G Suspect or Irrelevant (Bond) Angle(s) in CIF ...      44.70 Deg.
              C26B -C62B -C25B    2_656   1_555   2_656 ......     # 6824 Check
PLAT779_ALERT_4_G Suspect or Irrelevant (Bond) Angle(s) in CIF ...      27.40 Deg.
              C50B -C62B -C25B    1_555   1_555   2_656 ......     # 6827 Check
PLAT779_ALERT_4_G Suspect or Irrelevant (Bond) Angle(s) in CIF ...       8.80 Deg.
              C64B -C63B -C24B    1_555   1_555   2_656 ......     # 6850 Check
PLAT779_ALERT_4_G Suspect or Irrelevant (Bond) Angle(s) in CIF ...      18.40 Deg.
              C62B -C63B -C27B    1_555   1_555   2_656 ......     # 6853 Check
PLAT779_ALERT_4_G Suspect or Irrelevant (Bond) Angle(s) in CIF ...      42.00 Deg.
              C24B -C64B -C49B    2_656   1_555   1_555 ......     # 6858 Check
PLAT779_ALERT_4_G Suspect or Irrelevant (Bond) Angle(s) in CIF ...      32.10 Deg.
              C23B -C64B -C63B    2_656   1_555   1_555 ......     # 6861 Check
PLAT779_ALERT_4_G Suspect or Irrelevant (Bond) Angle(s) in CIF ...      36.10 Deg.
              C36B -C64B -C19B    1_555   1_555   2_656 ......     # 6871 Check
PLAT779_ALERT_4_G Suspect or Irrelevant (Bond) Angle(s) in CIF ...      14.80 Deg.
              C49B -C64B -C25B    1_555   1_555   2_656 ......     # 6874 Check
PLAT779_ALERT_4_G Suspect or Irrelevant (Bond) Angle(s) in CIF ...      44.00 Deg.
              C24B -C64B -ND3B    2_656   1_555   1_555 ......     # 6878 Check
PLAT779_ALERT_4_G Suspect or Irrelevant (Bond) Angle(s) in CIF ...      41.03 Deg.
              ND3B -C64B -ND1B    1_555   1_555   1_555 ......     # 6892 Check
PLAT779_ALERT_4_G Suspect or Irrelevant (Bond) Angle(s) in CIF ...      44.00 Deg.
              C18B -C65B -C37B    2_656   1_555   1_555 ......     # 6896 Check
PLAT779_ALERT_4_G Suspect or Irrelevant (Bond) Angle(s) in CIF ...      37.30 Deg.
              C73B -C65B -C48B    2_656   1_555   1_555 ......     # 6904 Check
PLAT779_ALERT_4_G Suspect or Irrelevant (Bond) Angle(s) in CIF ...      25.00 Deg.
              C18B -C65B -C19B    2_656   1_555   2_656 ......     # 6908 Check
PLAT779_ALERT_4_G Suspect or Irrelevant (Bond) Angle(s) in CIF ...      35.70 Deg.
              C37B -C65B -C19B    1_555   1_555   2_656 ......     # 6911 Check
PLAT779_ALERT_4_G Suspect or Irrelevant (Bond) Angle(s) in CIF ...      38.00 Deg.
              C18B -C65B -ND1B    2_656   1_555   1_555 ......     # 6914 Check
PLAT779_ALERT_4_G Suspect or Irrelevant (Bond) Angle(s) in CIF ...      42.20 Deg.
              ND1B -C65B -ND3B    1_555   1_555   1_555 ......     # 6928 Check
PLAT779_ALERT_4_G Suspect or Irrelevant (Bond) Angle(s) in CIF ...      11.27 Deg.
              ND1B -C65B -ND2B    1_555   1_555   2_656 ......     # 6936 Check
PLAT779_ALERT_4_G Suspect or Irrelevant (Bond) Angle(s) in CIF ...      10.47 Deg.
              ND3B -C65B -ND4B    1_555   1_555   2_656 ......     # 6946 Check
PLAT779_ALERT_4_G Suspect or Irrelevant (Bond) Angle(s) in CIF ...      19.00 Deg.
              C6B  -C66B -C71B    2_656   1_555   1_555 ......     # 6949 Check
PLAT779_ALERT_4_G Suspect or Irrelevant (Bond) Angle(s) in CIF ...      32.10 Deg.
              C7B  -C66B -C67B    2_656   1_555   1_555 ......     # 6952 Check
PLAT779_ALERT_4_G Suspect or Irrelevant (Bond) Angle(s) in CIF ...      42.80 Deg.
              C71B -C66B -C5B     1_555   1_555   2_656 ......     # 6960 Check
PLAT779_ALERT_4_G Suspect or Irrelevant (Bond) Angle(s) in CIF ...      36.90 Deg.
              C2B  -C66B -C8B     1_555   1_555   2_656 ......     # 6967 Check
PLAT779_ALERT_4_G Suspect or Irrelevant (Bond) Angle(s) in CIF ...      29.70 Deg.
              C80B -C67B -C41B    2_656   1_555   1_555 ......     # 6972 Check
PLAT779_ALERT_4_G Suspect or Irrelevant (Bond) Angle(s) in CIF ...      31.10 Deg.
              C7B  -C67B -C66B    2_656   1_555   1_555 ......     # 6976 Check
PLAT779_ALERT_4_G Suspect or Irrelevant (Bond) Angle(s) in CIF ...      21.20 Deg.
              C68B -C67B -C54B    1_555   1_555   2_656 ......     # 6981 Check
```

```
PLAT779_ALERT_4_G Suspect or Irrelevant (Bond) Angle(s) in CIF ...      23.60 Deg.
              C66B -C67B -C6B     1_555   1_555   2_656 ......     # 6988 Check
PLAT779_ALERT_4_G Suspect or Irrelevant (Bond) Angle(s) in CIF ...      27.70 Deg.
              C80B -C67B -C14B    2_656   1_555   2_656 ......     # 6990 Check
PLAT779_ALERT_4_G Suspect or Irrelevant (Bond) Angle(s) in CIF ...       2.00 Deg.
              C41B -C67B -C14B    1_555   1_555   2_656 ......     # 6993 Check
PLAT779_ALERT_4_G Suspect or Irrelevant (Bond) Angle(s) in CIF ...      15.90 Deg.
              C53B -C68B -C69B    2_656   1_555   1_555 ......     # 7001 Check
PLAT779_ALERT_4_G Suspect or Irrelevant (Bond) Angle(s) in CIF ...      29.30 Deg.
              C67B -C68B -C80B    1_555   1_555   2_656 ......     # 7005 Check
PLAT779_ALERT_4_G Suspect or Irrelevant (Bond) Angle(s) in CIF ...      15.40 Deg.
              C54B -C68B -C78B    2_656   1_555   1_555 ......     # 7007 Check
PLAT779_ALERT_4_G Suspect or Irrelevant (Bond) Angle(s) in CIF ...      37.00 Deg.
              C69B -C68B -C79B    1_555   1_555   2_656 ......     # 7015 Check
PLAT779_ALERT_4_G Suspect or Irrelevant (Bond) Angle(s) in CIF ...      20.80 Deg.
              C54B -C68B -C55B    2_656   1_555   2_656 ......     # 7018 Check
PLAT779_ALERT_4_G Suspect or Irrelevant (Bond) Angle(s) in CIF ...       7.60 Deg.
              C78B -C68B -C55B    1_555   1_555   2_656 ......     # 7023 Check
PLAT779_ALERT_4_G Suspect or Irrelevant (Bond) Angle(s) in CIF ...      36.30 Deg.
              C53B -C69B -C68B    2_656   1_555   1_555 ......     # 7036 Check
PLAT779_ALERT_4_G Suspect or Irrelevant (Bond) Angle(s) in CIF ...      24.00 Deg.
              C79B -C69B -C70B    2_656   1_555   1_555 ......     # 7040 Check
PLAT779_ALERT_4_G Suspect or Irrelevant (Bond) Angle(s) in CIF ...       7.90 Deg.
              C52B -C69B -C75B    2_656   1_555   1_555 ......     # 7045 Check
PLAT779_ALERT_4_G Suspect or Irrelevant (Bond) Angle(s) in CIF ...      26.80 Deg.
              C70B -C69B -C46B    1_555   1_555   2_656 ......     # 7052 Check
PLAT779_ALERT_4_G Suspect or Irrelevant (Bond) Angle(s) in CIF ...      32.00 Deg.
              C53B -C69B -C54B    2_656   1_555   2_656 ......     # 7054 Check
PLAT779_ALERT_4_G Suspect or Irrelevant (Bond) Angle(s) in CIF ...      14.60 Deg.
              C68B -C69B -C54B    1_555   1_555   2_656 ......     # 7057 Check
PLAT779_ALERT_4_G Suspect or Irrelevant (Bond) Angle(s) in CIF ...       8.87 Deg.
              ND4B -C69B -ND3B    1_555   1_555   2_656 ......     # 7087 Check
PLAT779_ALERT_4_G Suspect or Irrelevant (Bond) Angle(s) in CIF ...      22.80 Deg.
              C46B -C70B -C72B    2_656   1_555   1_555 ......     # 7092 Check
PLAT779_ALERT_4_G Suspect or Irrelevant (Bond) Angle(s) in CIF ...      24.00 Deg.
              C71B -C70B -C6B     1_555   1_555   2_656 ......     # 7100 Check
PLAT779_ALERT_4_G Suspect or Irrelevant (Bond) Angle(s) in CIF ...      41.30 Deg.
              C79B -C70B -C53B    2_656   1_555   2_656 ......     # 7103 Check
PLAT779_ALERT_4_G Suspect or Irrelevant (Bond) Angle(s) in CIF ...       9.70 Deg.
              C69B -C70B -C53B    1_555   1_555   2_656 ......     # 7107 Check
PLAT779_ALERT_4_G Suspect or Irrelevant (Bond) Angle(s) in CIF ...      25.00 Deg.
              C72B -C70B -C45B    1_555   1_555   2_656 ......     # 7112 Check
PLAT779_ALERT_4_G Suspect or Irrelevant (Bond) Angle(s) in CIF ...      38.40 Deg.
              C46B -C70B -C47B    2_656   1_555   2_656 ......     # 7117 Check
PLAT779_ALERT_4_G Suspect or Irrelevant (Bond) Angle(s) in CIF ...      25.70 Deg.
              C70B -C71B -C79B    1_555   1_555   2_656 ......     # 7138 Check
PLAT779_ALERT_4_G Suspect or Irrelevant (Bond) Angle(s) in CIF ...      19.60 Deg.
              C6B  -C71B -C66B    2_656   1_555   1_555 ......     # 7139 Check
PLAT779_ALERT_4_G Suspect or Irrelevant (Bond) Angle(s) in CIF ...      44.90 Deg.
              C5B  -C71B -C10B    2_656   1_555   1_555 ......     # 7144 Check
PLAT779_ALERT_4_G Suspect or Irrelevant (Bond) Angle(s) in CIF ...       6.80 Deg.
              C10B -C71B -C4B     1_555   1_555   2_656 ......     # 7153 Check
PLAT779_ALERT_4_G Suspect or Irrelevant (Bond) Angle(s) in CIF ...      18.60 Deg.
              C70B -C71B -C46B    1_555   1_555   2_656 ......     # 7156 Check
PLAT779_ALERT_4_G Suspect or Irrelevant (Bond) Angle(s) in CIF ...      44.40 Deg.
              C79B -C71B -C46B    2_656   1_555   2_656 ......     # 7157 Check
PLAT779_ALERT_4_G Suspect or Irrelevant (Bond) Angle(s) in CIF ...       7.10 Deg.
```

```
              C45B -C72B -C11B    2_656   1_555   1_555 ......     # 7163 Check
PLAT779_ALERT_4_G Suspect or Irrelevant (Bond) Angle(s) in CIF ...      27.70 Deg.
              C46B -C72B -C70B    2_656   1_555   1_555 ......     # 7164 Check
PLAT779_ALERT_4_G Suspect or Irrelevant (Bond) Angle(s) in CIF ...      26.30 Deg.
              C46B -C72B -C79B    2_656   1_555   2_656 ......     # 7182 Check
PLAT779_ALERT_4_G Suspect or Irrelevant (Bond) Angle(s) in CIF ...       3.00 Deg.
              C70B -C72B -C79B    1_555   1_555   2_656 ......     # 7185 Check
PLAT779_ALERT_4_G Suspect or Irrelevant (Bond) Angle(s) in CIF ...       9.32 Deg.
              ND4B -C72B -ND3B    1_555   1_555   2_656 ......     # 7205 Check
PLAT779_ALERT_4_G Suspect or Irrelevant (Bond) Angle(s) in CIF ...       9.80 Deg.
              C65B -C73B -C18B    2_656   1_555   1_555 ......     # 7211 Check
PLAT779_ALERT_4_G Suspect or Irrelevant (Bond) Angle(s) in CIF ...      42.10 Deg.
              C48B -C73B -C74B    2_656   1_555   1_555 ......     # 7212 Check
PLAT779_ALERT_4_G Suspect or Irrelevant (Bond) Angle(s) in CIF ...      41.60 Deg.
              C47B -C73B -C74B    2_656   1_555   1_555 ......     # 7213 Check
PLAT779_ALERT_4_G Suspect or Irrelevant (Bond) Angle(s) in CIF ...      23.20 Deg.
              C72B -C73B -C46B    1_555   1_555   2_656 ......     # 7226 Check
PLAT779_ALERT_4_G Suspect or Irrelevant (Bond) Angle(s) in CIF ...      22.50 Deg.
              C72B -C73B -C45B    1_555   1_555   2_656 ......     # 7239 Check
PLAT779_ALERT_4_G Suspect or Irrelevant (Bond) Angle(s) in CIF ...      42.40 Deg.
              C44B -C73B -C45B    2_656   1_555   2_656 ......     # 7241 Check
PLAT779_ALERT_4_G Suspect or Irrelevant (Bond) Angle(s) in CIF ...      12.75 Deg.
              ND4B -C73B -ND3B    1_555   1_555   2_656 ......     # 7260 Check
PLAT779_ALERT_4_G Suspect or Irrelevant (Bond) Angle(s) in CIF ...      38.90 Deg.
              C48B -C74B -C73B    2_656   1_555   1_555 ......     # 7263 Check
PLAT779_ALERT_4_G Suspect or Irrelevant (Bond) Angle(s) in CIF ...      17.70 Deg.
              C25B -C74B -C49B    1_555   1_555   2_656 ......     # 7274 Check
PLAT779_ALERT_4_G Suspect or Irrelevant (Bond) Angle(s) in CIF ...      10.10 Deg.
              C75B -C74B -C52B    1_555   1_555   2_656 ......     # 7280 Check
PLAT779_ALERT_4_G Suspect or Irrelevant (Bond) Angle(s) in CIF ...      28.60 Deg.
              C25B -C74B -C50B    1_555   1_555   2_656 ......     # 7285 Check
PLAT779_ALERT_4_G Suspect or Irrelevant (Bond) Angle(s) in CIF ...      36.30 Deg.
              C75B -C74B -C51B    1_555   1_555   2_656 ......     # 7293 Check
PLAT779_ALERT_4_G Suspect or Irrelevant (Bond) Angle(s) in CIF ...      42.90 Deg.
              C50B -C74B -C51B    2_656   1_555   2_656 ......     # 7296 Check
PLAT779_ALERT_4_G Suspect or Irrelevant (Bond) Angle(s) in CIF ...      14.05 Deg.
              ND3B -C74B -ND4B    2_656   1_555   1_555 ......     # 7315 Check
PLAT779_ALERT_4_G Suspect or Irrelevant (Bond) Angle(s) in CIF ...      13.80 Deg.
              C51B -C75B -C76B    2_656   1_555   1_555 ......     # 7318 Check
PLAT779_ALERT_4_G Suspect or Irrelevant (Bond) Angle(s) in CIF ...      38.00 Deg.
              C52B -C75B -C69B    2_656   1_555   1_555 ......     # 7322 Check
PLAT779_ALERT_4_G Suspect or Irrelevant (Bond) Angle(s) in CIF ...      38.20 Deg.
              C74B -C75B -C47B    1_555   1_555   2_656 ......     # 7329 Check
PLAT779_ALERT_4_G Suspect or Irrelevant (Bond) Angle(s) in CIF ...      43.00 Deg.
              C52B -C75B -C53B    2_656   1_555   2_656 ......     # 7331 Check
PLAT779_ALERT_4_G Suspect or Irrelevant (Bond) Angle(s) in CIF ...      17.20 Deg.
              C69B -C75B -C53B    1_555   1_555   2_656 ......     # 7335 Check
PLAT779_ALERT_4_G Suspect or Irrelevant (Bond) Angle(s) in CIF ...      13.36 Deg.
              ND4B -C75B -ND3B    1_555   1_555   2_656 ......     # 7351 Check
PLAT779_ALERT_4_G Suspect or Irrelevant (Bond) Angle(s) in CIF ...      17.00 Deg.
              C56B -C76B -C77B    2_656   1_555   1_555 ......     # 7356 Check
PLAT779_ALERT_4_G Suspect or Irrelevant (Bond) Angle(s) in CIF ...       5.30 Deg.
              C75B -C76B -C52B    1_555   1_555   2_656 ......     # 7364 Check
PLAT779_ALERT_4_G Suspect or Irrelevant (Bond) Angle(s) in CIF ...      17.40 Deg.
              C26B -C76B -C50B    1_555   1_555   2_656 ......     # 7371 Check
PLAT779_ALERT_4_G Suspect or Irrelevant (Bond) Angle(s) in CIF ...      11.92 Deg.
              ND3B -C76B -ND4B    2_656   1_555   1_555 ......     # 7387 Check
```

```
PLAT779_ALERT_4_G Suspect or Irrelevant (Bond) Angle(s) in CIF ...      15.60 Deg.
              C55B -C77B -C78B    2_656   1_555   1_555 ......     # 7392 Check
PLAT779_ALERT_4_G Suspect or Irrelevant (Bond) Angle(s) in CIF ...      36.00 Deg.
              C56B -C77B -C76B    2_656   1_555   1_555 ......     # 7394 Check
PLAT779_ALERT_4_G Suspect or Irrelevant (Bond) Angle(s) in CIF ...      26.50 Deg.
              C33B -C77B -C57B    1_555   1_555   2_656 ......     # 7400 Check
PLAT779_ALERT_4_G Suspect or Irrelevant (Bond) Angle(s) in CIF ...      39.00 Deg.
              C56B -C77B -C51B    2_656   1_555   2_656 ......     # 7403 Check
PLAT779_ALERT_4_G Suspect or Irrelevant (Bond) Angle(s) in CIF ...       3.30 Deg.
              C76B -C77B -C51B    1_555   1_555   2_656 ......     # 7407 Check
PLAT779_ALERT_4_G Suspect or Irrelevant (Bond) Angle(s) in CIF ...      18.30 Deg.
              C33B -C77B -C58B    1_555   1_555   2_656 ......     # 7418 Check
PLAT779_ALERT_4_G Suspect or Irrelevant (Bond) Angle(s) in CIF ...      44.90 Deg.
              C57B -C77B -C58B    2_656   1_555   2_656 ......     # 7421 Check
PLAT779_ALERT_4_G Suspect or Irrelevant (Bond) Angle(s) in CIF ...      42.70 Deg.
              C59B -C77B -C58B    2_656   1_555   2_656 ......     # 7423 Check
PLAT779_ALERT_4_G Suspect or Irrelevant (Bond) Angle(s) in CIF ...      10.31 Deg.
              ND4B -C77B -ND3B    1_555   1_555   2_656 ......     # 7442 Check
PLAT779_ALERT_4_G Suspect or Irrelevant (Bond) Angle(s) in CIF ...      29.10 Deg.
              C55B -C78B -C77B    2_656   1_555   1_555 ......     # 7444 Check
PLAT779_ALERT_4_G Suspect or Irrelevant (Bond) Angle(s) in CIF ...      10.10 Deg.
              C54B -C78B -C68B    2_656   1_555   1_555 ......     # 7447 Check
PLAT779_ALERT_4_G Suspect or Irrelevant (Bond) Angle(s) in CIF ...      34.30 Deg.
              C55B -C78B -C56B    2_656   1_555   2_656 ......     # 7458 Check
PLAT779_ALERT_4_G Suspect or Irrelevant (Bond) Angle(s) in CIF ...       9.90 Deg.
              C77B -C78B -C56B    1_555   1_555   2_656 ......     # 7460 Check
PLAT779_ALERT_4_G Suspect or Irrelevant (Bond) Angle(s) in CIF ...      44.30 Deg.
              C54B -C78B -C80B    2_656   1_555   2_656 ......     # 7465 Check
PLAT779_ALERT_4_G Suspect or Irrelevant (Bond) Angle(s) in CIF ...      16.90 Deg.
              C70B -C79B -C46B    2_656   1_555   1_555 ......     # 7474 Check
PLAT779_ALERT_4_G Suspect or Irrelevant (Bond) Angle(s) in CIF ...      17.40 Deg.
              C69B -C79B -C53B    2_656   1_555   1_555 ......     # 7478 Check
PLAT779_ALERT_4_G Suspect or Irrelevant (Bond) Angle(s) in CIF ...      29.70 Deg.
              C71B -C79B -C6B     2_656   1_555   1_555 ......     # 7483 Check
PLAT779_ALERT_4_G Suspect or Irrelevant (Bond) Angle(s) in CIF ...      32.40 Deg.
              C53B -C79B -C68B    1_555   1_555   2_656 ......     # 7490 Check
PLAT779_ALERT_4_G Suspect or Irrelevant (Bond) Angle(s) in CIF ...       6.90 Deg.
              C70B -C79B -C72B    2_656   1_555   2_656 ......     # 7492 Check
PLAT779_ALERT_4_G Suspect or Irrelevant (Bond) Angle(s) in CIF ...      12.80 Deg.
              C46B -C79B -C72B    1_555   1_555   2_656 ......     # 7495 Check
PLAT779_ALERT_4_G Suspect or Irrelevant (Bond) Angle(s) in CIF ...      12.00 Deg.
              C41B -C80B -C14B    2_656   1_555   1_555 ......     # 7509 Check
PLAT779_ALERT_4_G Suspect or Irrelevant (Bond) Angle(s) in CIF ...      30.50 Deg.
              C67B -C80B -C7B     2_656   1_555   1_555 ......     # 7510 Check
PLAT779_ALERT_4_G Suspect or Irrelevant (Bond) Angle(s) in CIF ...      24.10 Deg.
              C54B -C80B -C68B    1_555   1_555   2_656 ......     # 7521 Check
PLAT779_ALERT_4_G Suspect or Irrelevant (Bond) Angle(s) in CIF ...      26.40 Deg.
              C54B -C80B -C78B    1_555   1_555   2_656 ......     # 7532 Check
PLAT779_ALERT_4_G Suspect or Irrelevant (Bond) Angle(s) in CIF ...      29.00 Deg.
              C81B -C81B -F6      2_656   1_555   1_555 ......     # 7538 Check
PLAT779_ALERT_4_G Suspect or Irrelevant (Bond) Angle(s) in CIF ...      42.00 Deg.
              F6   -C81B -F4      1_555   1_555   2_656 ......     # 7548 Check
PLAT779_ALERT_4_G Suspect or Irrelevant (Bond) Angle(s) in CIF ...      32.00 Deg.
              C81B -C81B -F5      2_656   1_555   2_656 ......     # 7556 Check
PLAT779_ALERT_4_G Suspect or Irrelevant (Bond) Angle(s) in CIF ...      16.60 Deg.
              C1B  -C81B -C1B     1_555   1_555   2_656 ......     # 7569 Check
PLAT779_ALERT_4_G Suspect or Irrelevant (Bond) Angle(s) in CIF ...      15.40 Deg.
```

```
              C81B -F4   -C81B    1_555   1_555   2_656 ......     # 7573 Check
PLAT779_ALERT_4_G Suspect or Irrelevant (Bond) Angle(s) in CIF ...       9.00 Deg.
              C81B -F5   -C81B    1_555   1_555   2_656 ......     # 7579 Check
PLAT779_ALERT_4_G Suspect or Irrelevant (Bond) Angle(s) in CIF ...      11.00 Deg.
              C81B -F6   -C81B    2_656   1_555   1_555 ......     # 7587 Check
PLAT780_ALERT_1_G Coordinates do not Form a Properly Connected Set     Please Do !
PLAT793_ALERT_4_G Model has Chirality at C1B         (Centro SPGR)         R Verify
PLAT793_ALERT_4_G Model has Chirality at C1C         (Centro SPGR)         S Verify
PLAT811_ALERT_5_G No ADDSYM Analysis: Too Many Excluded Atoms ....         ! Info
PLAT860_ALERT_3_G Number of Least-Squares Restraints ............       8477 Note
PLAT883_ALERT_1_G No Info/Value for _atom_sites_solution_primary .     Please Do !
PLAT912_ALERT_4_G Missing # of FCF Reflections Above STh/L=  0.600       6930 Note
PLAT952_ALERT_5_G Calculated (ThMax) and CIF-Reported Lmax Differ.         2 Units
PLAT958_ALERT_1_G Calculated (ThMax) and Actual (FCF) Lmax Differ.         2 Units
PLAT965_ALERT_2_G The SHELXL WEIGHT Optimisation has not Converged     Please Check
PLAT978_ALERT_2_G Number C-C Bonds with Positive Residual Density.         6 Info
PLAT984_ALERT_1_G The  C-f'=    0.0033 Deviates from the B&C-Value     0.0043 Check
PLAT984_ALERT_1_G The  F-f'=    0.0186 Deviates from the B&C-Value     0.0225 Check
PLAT984_ALERT_1_G The  N-f'=    0.0063 Deviates from the B&C-Value     0.0082 Check
PLAT984_ALERT_1_G The Nd-f'=   -0.4357 Deviates from the B&C-Value    -0.0517 Check
PLAT984_ALERT_1_G The Ni-f'=    0.2535 Deviates from the B&C-Value     0.3143 Check
PLAT985_ALERT_1_G The Nd-f"=    3.7052 Deviates from the B&C-Value     3.7170 Check
PLAT985_ALERT_1_G The Ni-f"=    1.3759 Deviates from the B&C-Value     1.3773 Check
```

---

8 **ALERT level A** = Most likely a serious problem - resolve or explain
3 **ALERT level B** = A potentially serious problem, consider carefully
89 **ALERT level C** = Check. Ensure it is not caused by an omission or oversight
2389 **ALERT level G** = General information/check it is not something unexpected

68 ALERT type 1 CIF construction/syntax error, inconsistent or missing data
1299 ALERT type 2 Indicator that the structure model may be wrong or deficient
9 ALERT type 3 Indicator that the structure quality may be low
1111 ALERT type 4 Improvement, methodology, query or suggestion
2 ALERT type 5 Informative message, check

It is advisable to attempt to resolve as many as possible of the alerts in all categories. Often the minor alerts point to easily fixed oversights, errors and omissions in your CIF or refinement strategy, so attention to these fine details can be worthwhile. In order to resolve some of the more serious problems it may be necessary to carry out additional measurements or structure refinements. However, the purpose of your study may justify the reported deviations and the more serious of these should normally be commented upon in the discussion or experimental section of a paper or in the "special_details" fields of the CIF. checkCIF was carefully designed to identify outliers and unusual parameters, but every test has its limitations and alerts that are not important in a particular case may appear. Conversely, the absence of alerts does not guarantee there are no aspects of the results needing attention. It is up to the individual to critically assess their own results and, if necessary, seek expert advice.

Publication of your CIF in IUCr journals

A basic structural check has been run on your CIF. These basic checks will be run on all CIFs submitted for publication in IUCr journals (*Acta Crystallographica*, *Journal of Applied Crystallography*, *Journal of Synchrotron Radiation*); however, if you intend to submit to *Acta Crystallographica Section C* or *E* or *IUCrData*, you should make sure that full publication checks are run on the final version of your CIF prior to submission.

Publication of your CIF in other journals

Please refer to the *Notes for Authors* of the relevant journal for any special instructions relating to CIF submission.

PLATON version of 20/01/2022; check.def file version of 19/01/2022

Datablock nd2@c80-cf3-i - ellipsoid plot

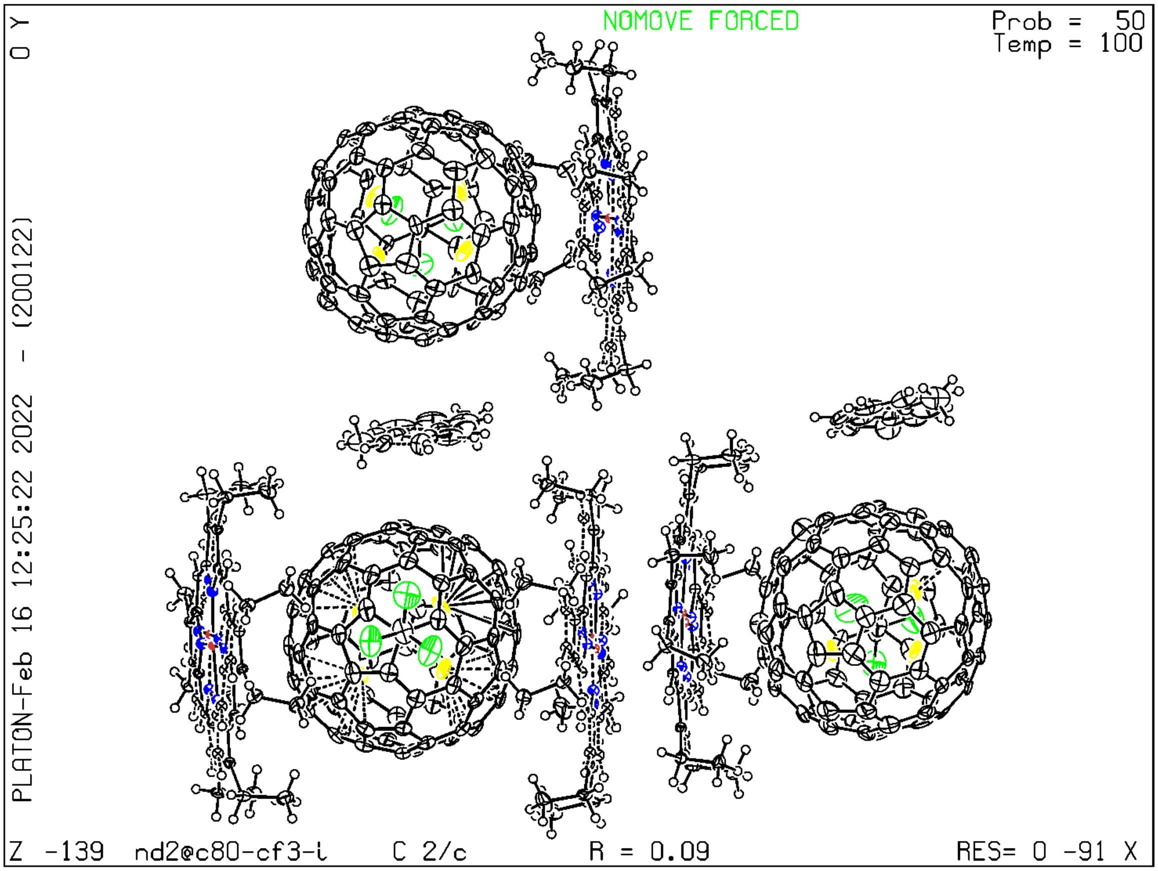

Supplement: Supplementary file 2 — Supporting Information [file ADVS-11-2305190-s002.zip › advs202305190-sup-0002-cif/nd2@c80-cf3-i_submission_checkcif.pdf]
